# Proteome Dynamics: Tissue Variation in the Kinetics of Proteostasis in Intact Animals* [Fn FN2]

**DOI:** 10.1074/mcp.M115.053488

**Published:** 2016-02-01

**Authors:** Dean E. Hammond, Amy J. Claydon, Deborah M. Simpson, Dominic Edward, Paula Stockley, Jane L. Hurst, Robert J. Beynon

**Affiliations:** From the ‡Centre for Proteome Research, Institute of Integrative Biology, University of Liverpool, Crown Street, L697ZB, UK;; §Mammalian Behaviour and Evolution Group, Institute of Integrative Biology, University of Liverpool, Leahurst Campus, Neston, CH64 7TE, UK

## Abstract

Understanding the role of protein turnover in the maintenance of proteostasis requires accurate measurements of the rates of replacement of proteins in complex systems, such as intact animals. Moreover, any investigation of allometric scaling of protein turnover is likely to include species for which fully annotated proteomes are not available. We have used dietary administration of stable isotope labeled lysine to assess protein turnover rates for proteins from four tissues in the bank vole, *Myodes glareolus*. The annotated genome for this species is not available, so protein identification was attained through cross-species matching to the mouse. For proteins for which confident identifications were derived, the pattern of lysine incorporation over 40 days was used to define the rate of synthesis of individual proteins in the four tissues. The data were heavily filtered to retain a very high quality dataset of turnover rates for 1088 proteins. Comparative analysis of the four tissues revealed different median rates of degradation (kidney: 0.099 days^−1^; liver 0.136 days^−1^; heart, 0.054 days^−1^, and skeletal muscle, 0.035 days^−1^). These data were compared with protein degradation rates from other studies on intact animals or from cells in culture and indicate that both cell type and analytical methodology may contribute to variance in turnover data between different studies. These differences were not only due to tissue-specific proteins but were reflected in gene products common to all tissues. All data are available via ProteomeXchange with identifier PXD002054.

Proteostasis balances the opposing contributions of protein synthesis and protein degradation in the maintenance or adjustment of the intracellular abundance of a protein. Accurate determination of the net contribution of these two processes requires accurate determination of at least two of the three parameters of synthesis rate, degradation rate, and protein pool size. Moreover, these parameters need to be recoverable at proteome scales, extending to many proteins in a parallel analysis within a single experiment. Because synthesis and degradation can still occur even when the protein concentration is unchanging, it is necessary to monitor the flux through the protein pool by a tracer, and in a mass spectrometry-driven proteomics context, this involves the incorporation of a stable isotope label. The tracer can be administered as a metabolic precursor (1, 2), an amino acid (3–5), a microbially sourced diet uniformly labeled with ^15^N, or by administration of [^2^H_2_]O in drinking water (6–9).

Many studies of proteome dynamics have been conducted in mammalian cells in culture (for reviews see (10, 11)). The experimental convenience of effecting rapid switching between a labeled and a subsequent unlabeled precursor pool by medium exchange is, however, offset by the fact that rapidly dividing cells are able to “solve” the problem of protein level adjustment by dilution into progeny cells. This dilution of the isotopically labeled pool not only restricts the scope to monitor the labeled pool but also may be focusing turnover studies on cells that bear little resemblance in their protein turnover to the same or similar cells in a tissue (11, 12). There is therefore a requirement to determine protein turnover in intact animals, which imposes considerable complexity in experimental design and data analysis (13–16). In tissues, protein turnover is substantially slower than in cells in culture (12, 17), and it is necessary to administer label for extended periods, rendering oral (food or drinking water) administration as the only feasible option.

Measurement of turnover in rapidly growing cells in culture suffers from the rapid loss or gain of label as a consequence of growth and, thus, dilution of the labeled protein pool. By contrast, measurement of turnover in animal tissues, particularly in nongrowing individuals, requires a different strategy (13, 14, 16, 18–20). It is challenging and prohibitively expensive to label animals fully over multiple generations and subsequently monitor the loss of label over time. Further, a strategy that measures the transition between fully labeled and fully unlabeled proteins requires specialist, completely labeled diets that differ substantially from normal laboratory diets. To circumvent such difficulties, we have developed a strategy based on the addition of a single stable isotope labeled amino acid to a laboratory diet, such that the isotopic enrichment of the total amino acid in the diet would be ∼0.5. The precise degree of dietary labeling is not critical, as this is revealed during analysis. Animals are acclimated to the modified diet containing nonlabeled amino acid before being transferred to the same composition, labeled diet. Subsequently, incorporation of label into the body precursor pool can be monitored noninvasively by measurement of the relative isotope abundance (RIA)^1^ of secreted proteins, particularly those released in urine (16). We have previously used the essential amino acid, valine, to measure protein turnover in the house mouse, *Mus musculus domesticus* (16). The valine was administered by supplementation of a standard laboratory diet by incorporation of crystalline [^2^H_8_]valine to the same level as originally present in the diet (in protein bound or free form). Thus, the dietary RIA for the valine was set to a nominal value of 0.5. This approach worked very well, apart from an additional (but surmountable, (16)) complication due to partial transamination of the valine that led to loss of the alpha carbon deuteron to form a mixture of [^2^H_8_] and [^2^H_7_]valine. In this study, to simplify the strategy for determination of protein turnover rates, we have substituted [^13^C_6_]lysine for the [^2^H_8_]valine used previously. Lysine is also an essential amino acid, and so the RIA of the precursor pool cannot be reduced by biosynthesis *de novo*. Moreover, the labeling at the six carbon atoms precludes complications due to metabolic loss of specific atom centers. Finally, approximately half of the tryptic peptides (those that are lysine-terminated or which contain an internal LysPro sequence) should yield informative turnover data, although all tryptic peptides (both lysine and arginine terminated) can of course be used for protein identification.

We are interested in the allometric scaling of proteome turnover, which will require approaches that recover high-quality turnover rates from species for which fully annotated proteomes do not exist. To test the feasibility of this approach, we selected as our experimental system a rodent of similar body mass to the house mouse, the bank vole *Myodes glareoulus*. The annotated genome sequence of this rodent is not available, and thus, the measurement of protein turnover in this species brings two challenges: that of cross-species identification and also the recovery of species-specific turnover parameters. Finally, we have provided a robust analytical approach to the determination of protein turnover rates using the statistical package R that is thus amenable to use by all groups working in the field.

## EXPERIMENTAL PROCEDURES

### 

#### 

##### Experimental Animals

Adult male bank voles (*Myodes glareolus*) at least two months of age at the start of the experiment were from a colony derived from local populations in Cheshire that had been outbred for two generations in captivity. They were housed individually in polypropylene cages (48 × 15 × 13 cm, North Kent Plastic Cages Ltd., UK) containing substrate (Corn Cob Absorb 10/14 substrate) and paper wool nest material. Food and water were provided *ad libitum* (LabDiet 5002 Certified Rodent Diet, Purina Mills, St. Louis, MO). Animals were maintained on a reversed photo-period (light, 17 h; dark, 7 h; lights on at 16:30 h) and at 21 ± 1 °C. All bank voles were acclimated to a reconstituted semisynthetic diet (prepared in-house), based on the 5002 Certified Rodent Diet supplemented with [^12^C_6_]lysine at a quantity equal to the natural lysine content of the diet (1.18% (w/w)), for 7 days prior to the start of the experiment. This “light diet” was then replaced with a semisynthetic diet, identical apart from the substitution of the crystalline amino acid by [^13^C_6_]lysine at a relative isotope abundance (RIA) of 0.5. The dietary pellets were dissociated with water containing the dissolved [^13^C_6_]lysine to form a thick paste and mixed extensively. When fully homogeneous, the paste was then extruded into strips ∼1 cm across and dried in a commercial foodstuff drying oven, at 40 °C. This is referred to as “heavy diet.” At the indicated times (1, 5, 12, 25, and 40 days after the start of the dietary labeling period), pairs of animals were humanely killed, weighed, and dissected to recover the heart, kidneys, liver, and pooled hind leg skeletal muscle from each animal. All resected tissues were frozen at −20 °C for further analysis (see below). For analysis of urinary proteins, individuals were placed on a grid over a clean cage and the voided urine was collected for analysis. Because we were interested in the ratio of labeled to unlabeled peptides, each sample is self-referential and the quantity recovered is not relevant.

##### Sample Preparation

A sample (100–150 mg) of each tissue was homogenized for 30 s using a Polytron homogenizer (setting 3) in nine volumes (skeletal muscle, 19 volumes) using ice-cold 25 mm NH_4_HCO_3_ containing Roche complete-mini protease inhibitors (+EDTA) (Roche Diagnostics, West Sussex, UK) to a final concentration of 0.05% (w/v). Tissue samples were homogenized in labeling order from day 1 to day 40; the homogenizer was thoroughly rinsed between samples. Homogenates were immediately transferred to 2 ml microfuge tubes and centrifuged at 16,000 × *g* for 15 min at 4 °C. Cleared supernatants were transferred to clean low-bind tubes, and the protein concentration determined by dye-binding protein assay. A fixed amount of protein from each tissue (100 μg) was initially incubated at 80 °C for 10 min to aid RapiGest^TM^ (Waters, Manchester, UK)-mediated protein denaturation, after which time the sample was reduced (addition of 10 μl 60 mm dithiothreitol, 60 °C for 10 min) and alkylated (10 μl 180 mm iodoacetamide, room temperature, 30 min in the dark). Reduced and alkylated proteins were then digested using sequencing grade trypsin (lysine or arginine in P1 but not if P1′ contains proline) (Promega) at an enzyme:protein ratio of 1:50 overnight at 37 °C. After proteolysis, the digest was acidified by the addition of trifluoroacetic acid (final concentration of 1% (v/v)) and incubated at 37 °C for 45 min to allow precipitation of the acid labile surfactant. The digests were clarified at 16,000 × *g* for 30 min, the supernatant fraction transferred to fresh tubes and centrifuged again (16,000 × *g*, 30 min) before LC-MS/MS analysis of the final supernatant fraction.

##### Liquid Chromatography-Tandem Mass Spectrometry (LC-MS/MS)

Digests (2 μl) from each tissue taken at each time point, with duplicate animals per time point, were loaded onto a trap column (Acclaim PepMap 100, 2 cm × 75 μm inner diameter, C_18_, 3 μm, 100 Å) at 5 μl min^−1^ with an aqueous solution containing 0.1%(v/v) TFA and 2%(v/v) acetonitrile. After 3 min, the trap column was set in-line with an analytical column (Easy-Spray PepMap® RSLC 15 cm × 50cm inner diameter, C_18_, 2 μm, 100 Å) (Dionex). Peptides were loaded in 0.1%(v/v) formic acid and eluted with a linear gradient of 3.8–40% buffer B (HPLC grade acetonitrile 80%(v/v) with 0.1%(v/v) formic acid) over 95 min at 300 nl min^−1^, followed by a washing step (5 min at 99% solvent B) and an equilibration step (15 min at 3.8% solvent). All peptide separations were carried out using an Ultimate 3000 nano system (Dionex/Thermo Fisher Scientific). The column was operated at a constant temperature of 35 °C and the LC system coupled to a Q-Exactive mass spectrometer (Thermo Fisher Scientific). The Q-Exactive was operated in data-dependent mode with survey scans acquired at a resolution of 70,000 at *m/z* 200. Up to the top 10 most abundant isotope patterns with charge states +2, +3, and/or +4 from the survey scan were selected with an isolation window of 2.0 Th for fragmentation by higher energy collisional dissociation with normalized collision energies of 30. The maximum ion injection times for the survey scan and the MS/MS scans were 250 and 100 ms, respectively, and the ion target value was set to 1E6 for survey scans and 1E5 for the MS/MS scans. Repetitive sequencing of peptides was minimized through dynamic exclusion of the sequenced peptides for 20 s.

##### Mass Isotopomer Extraction, Protein Identification, and Quantification

Thermo raw MS data files from each tissue and duplicate animals over the labeling trajectory were analyzed using the Progenesis-QI software package (v2.0, Waters, Newcastle-upon-Tyne, UK). For each tissue, a total of 10 raw data files (two animals, five sampling times) were grouped and imported. MS “features” in Progenesis-QI, defined as peptide signals mapped in retention time *versus m/z* space, were warped and aligned to ensure that “like-for-like” peptide features were compared. Aligned, detected peptide features in each raw data file across the sampling time points were quantified using an area-under-the-curve method and converted into peak lists. Each peak list, on a tissue-by-tissue basis (*n* = 10 in each dataset) per experiment, were merged and searched using Mascot (version 2.4.1, Matrix Science, UK) against a UniProt *Mus* genus database (dated December 13, 2010) that contained 16,367 reviewed entries. The search parameters allowed for a single trypsin missed cleavage and carbamidomethylation of cysteine residues as a fixed modification. Oxidation of methionine and [^13^C_6_]lysine (from the time course of metabolic labeling) were allowed as variable modifications. A peptide tolerance of 10 ppm was set, with an MS/MS tolerance of 0.01 Da. Protein identifications were based on both arginine- and lysine-terminated tryptic peptide matches, but only lysine-terminated peptides carried the dynamic stable isotope labeling pattern for turnover measurement. To improve the quality of the search results and allow determination of false discovery rate (FDR), automatic decoy database searches were performed in Mascot. An FDR cutoff of 1% at the peptide level was applied to the database searches; only those lysine-containing peptide matches that satisfied these criteria were imported into Progenesis-QI to match feature-derived peptide quantitation(s) with identification. All other (lower quality) peptide data were ignored. The mass spectrometry proteomics data have been deposited to the ProteomeXchange Consortium (21) *via* the PRoteomics IDEntifications (PRIDE) partner repository with the dataset identifier PXD002054 and 10.6019/PXD002054.

To enable the feature-level quantitation performed by Progenesis-QI to be used in turnover calculations, we used Proteolabels, a development version of the Progenesis postprocessor tool (22), with an improved algorithm and graphical user interface. Proteolabels searches the Progenesis-QI feature table to recover cognate pairs of heavy (H) and light (L) features that coexist in the retention time dimension of aligned 2D LC-MS/MS maps, separated by the mass shift in *m/z* space due to incorporation of one or more [^13^C_6_]lysine residues. The output from Proteolabels is a .csv file containing feature identification information, protein identity, peptide sequence, and abundance information, together with computed isotope ratio data, specifically the relative isotope abundance (H/(H+L)); see below.

##### Calculation of Precursor RIA and Calculation of Turnover Rates

The relative isotope abundance of the amino acid precursor pool (RIA_p_) is a critical parameter in any turnover study, and experimental acquisition of this parameter should be included in the experimental workflow. In whole animal models, the initial value of RIA_p_ is zero, label is then introduced in the diet, and precursor incorporation into new protein during translation is monitored over time (16, 23). Assessing the distribution of heavy label in peptides containing two possible instances of the stable isotope labeled residue, arising either from incompletely labeled precursors, by dilution of the precursor pool by endogenous degradation-derived amino acids, or arising from biosynthesis *de novo*, is an effective method to calculate the RIA of heavy label in the amino acid precursor pool (11). For any di-lysine peptide, there are three possible MS peak envelopes visible on a mass spectrum: one corresponding to peptide species containing two light lysine residues (LL), one corresponding to peptides containing one light and one heavy lysine (LH and HL), and one representative of peptides containing two heavy lysine residues (HH). If the RIA of lysine is *r*, then the RIA of the light variant is (1 – *r*). So, the probability of a HH di-lysine peptide is *r*^2^ and an LL peptide would be (1 – *r*)^2^. The abundance (*i.e.* intensity/area-under-the-curve) of the HL peptide is therefore represented by 2(*r* × (1 – *r*)) (the multiplier of two allows for the two positional variants). Thus, the ratio of the abundances of the HH (*A*_HH_) and HL (*A*_HL_) peptides is

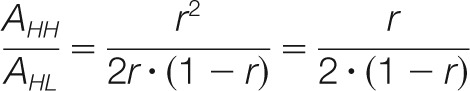


Knowing the abundance ratio of HH and HL (*x*) permits *r* (RIA_p_) to be calculated:

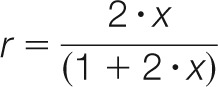


For each tissue, di-lysine peptides assigned to Progenesis-QI features were extracted from the output tables and analyzed to determine the RIA_p_ of each tissue (Supplemental Table S1). In this labeling system, the RIA at *t* = 0 is zero and increases as the labeled food is metabolized. A first order curve was fitted to the di-lysine peptide data recorded over time (RIA_t_) to obtain the maximum RIA plateau value (RIA_inf_) by nonlinear curve fitting. More specifically, only the reliable RIA_t_ data from late time points (day 12, day 25, and day 40) were used as they produce less variance about the mean. The data were fitted using a self-seeding nonlinear model in R (using nls(), *SSAsympOrig*). The horizontal asymptote given by the self-start nls function (RIA_inf_) is the best estimate of RIA_p_ in that tissue.

Once a value for RIA_p_ was established, the rate of turnover for individual proteins was calculated from RIA measurements of mono-lysine peptides at each sampling time point (*t*) within the experiment, again using nonlinear modeling (fixing RIA_0_ at 0 and RIA_inf_ at the predetermined maximum RIA_p_). The abundances of the labeled (*A*_H_) and unlabeled (*A*_L_) peptides were used to calculate the RIA_t_ = *A*_H_/(*A*_H_ + *A*_L_). The temporal change permits the calculation of the first-order rate constant for turnover (*k*_deg_):

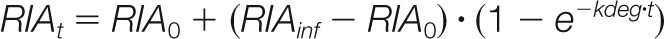
 where RIA_0_ = 0, RIA_inf_ is set as a fixed value for each tissue and *k* is the degradation rate constant (*k*_deg_) defining the rate of replacement (turnover) of the peptide/protein (Supplemental Fig. S2).

##### Turnover Data Processing

In-house generated scripts were created for postprocessing of Proteolabels output files. The scripts, written to separate mono- and multilabeled peptides, parse peptide feature data, and aggregate to protein-level, were written in Perl (v5.16.1). Scripts for mathematical modeling and generation of graphics were written in R (v3.0.3). RIA_t_ data for peptides that passed a 1% FDR threshold in the Mascot search were further filtered on the basis of two additional criteria; a) if any RIA_t_ value was > the mean ± three standard deviations of the RIA at *t* = 40 d, then the peptide was rejected, and b) RIA_t_ data for at least one of the biological replicate animals had to be evident at all time points of the labeling trajectory. If the criteria were unmet, the peptide was not used. Modified peptides were treated as separate entities to their unmodified counterparts throughout because the recovery of RIA_t_ is independent of the peptide abundance. To obtain protein-level turnover data, RIA_t_ values for peptides belonging to the same protein were grouped together and fitted using the nls() function in R. The resultant kinetic profiles were plotted with 95% confidence intervals, using ggplot2 (v1.0.0).

## RESULTS

We are interested in the allometric scaling of protein turnover (24). In particular, we wish to examine the profile of protein turnover in the tissues of animals of widely differing body masses, as it has long been established that protein metabolism, in common with other metabolic process, is considerably higher in animals of low body mass (25, 26). However, such studies also require analysis of protein turnover in nonmodel species for which full, annotated proteomes, or even genomes, are not yet available. Accordingly, in the first instance, we measured rates of protein turnover in four tissues of the bank vole (*Myodes glareolus)*, a rodent of similar body mass to the house mouse (*Mus domesticus*) that has been the subject of several global proteome turnover studies (8, 14, 16, 27, 28).

**Fig. 1.**
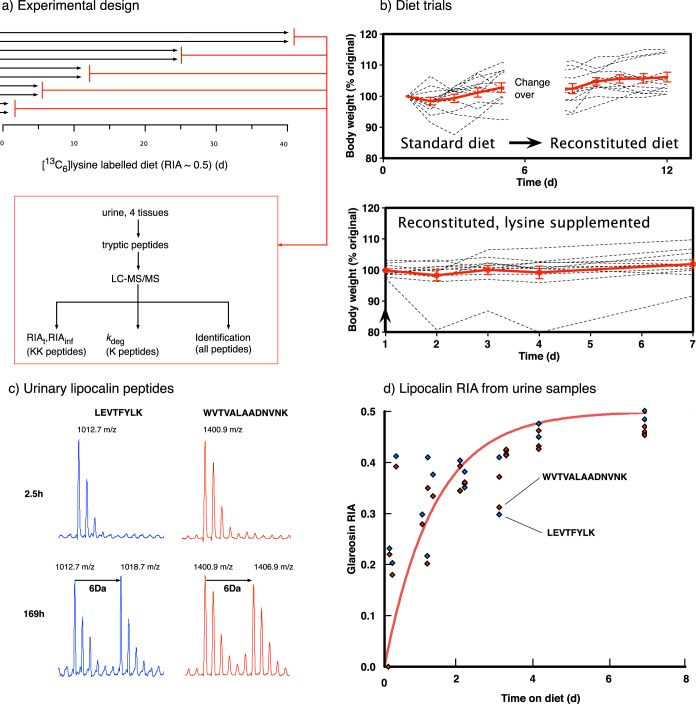
**Overall design and analytical structure of the study.** Bank voles (*Myodes glareolus*) were fed a diet containing 50% of the lysine in [^13^C_6_] labeled form for up to 40 days (*panel A*). Throughout that period, four tissues were recovered from two animals at specified time points, processed, and treated independently (true biological replicates). In addition, urine sampling was used to monitor the progress of labeling of urinary protein, predominantly the urinary lipocalins. The shift to the unsupplemented diet did not greatly influence body weight (*panel B*, top, individual data with mean weight ± S.E., red line) nor did the shift to the diet supplemented with [^12^C_6_]lysine (Fig. 1*B*, bottom panel). Evidence for uptake and incorporation of labeled lysine was obtained by analysis of urinary protein output, using two lysine-terminated peptides from a urinary lipocalin (glareosin, *panel C*, see text) and was used to obtain a preliminary assessment of the tissue (liver) precursor RIA (*panel D*).

Our experimental design (Fig. 1*A*) was developed from previous studies and is based on supplementation of standard laboratory diets with a stable isotope labeled amino acid to achieve an enrichment of ∼50% (11, 13). We conducted preliminary experiments to establish the palatability of the modified diet. First, the transition from standard laboratory diet to the diet used in this study was without effect. Second, bank voles (initial body weight between 18.4 g and 28.3 g) were placed on unlabeled, [^12^C_6_]lysine supplemented diet, and the animal weights were recorded for 7 days. During that time, there was no change in body weight (mean ± S.E., day 1: 24.3 ± 0.97 g, day 5: 24.7 ± 0.95 g, matched pair *t* test), so the modified, lysine supplemented, diet did not impair the ability of the animals to feed and maintain body mass (Fig. 1*B*). One individual exhibited a substantial weight loss in the acclimation study, but in the labeling experiment, there was no weight loss in any subject. When the bank voles were transferred to the heavy diet, weight was maintained with a small increase in body mass in all individuals. Evidence of uptake of [^13^C_6_]lysine and incorporation into protein was obtained by analysis of lysine-terminated peptides from a urinary protein recovered throughout the labeling period. Urine recovery is noninvasive and permits tracking of the isotope flux in tissues that contribute the urinary protein. For this study, we monitored a urinary lipocalin that we have characterized (named glareosin, J. Unsworth, *unpublished data*) (Fig. 1*C*). From the urine samples, we were able to establish noninvasively that urinary proteins were labeled and that the precursor pool had reached the anticipated RIA (Fig. 1*D*).

For tissue turnover studies, at times throughout the labeling study, pairs of animals were culled, and dissected tissues were stored frozen until all time points could be analyzed (Fig. 1*A*). This approach generates 10 independent measurements at five discrete time points that define the shape of the labeling curve and that can also be independently analyzed for protein identification.

### 

#### 

##### Working with an Organism Lacking a Well-Annotated Proteome

This study was conducted with the bank vole, a species for which the genome sequence has yet to be completed or fully annotated. A further complication could therefore have arisen because protein identifications could only be derived by reference to a well-characterized rodent proteome, in this instance, the mouse *Mus musculus domesticus*. To enhance the putative identification of bank vole proteins, we included in the search terms the additional variable modification of stable isotope labeling of lysine residues and searched with data collected throughout the labeling period. This gives added confidence to the requirement for C-terminal or internal lysine residues in mono or di-lysine peptides. We surmised that matching to this variable modification increased the likelihood of a meaningful identification and that a recurrent match over an entire labeling trajectory would also add confidence to the identification. The database searches gave 9,745, 9,756, 12,872, and 13,379 tryptic peptide spectral matches (PSMs) at a 1% FDR cutoff for heart, kidney, liver, and skeletal muscle, respectively. Of these, 48% (heart, 4,715 PSMs), 50% (kidney, 4,917 PSMs), 50% (liver, 6,391 PSMs), and 46% (skeletal muscle, 6,113 PSMs) were lysine-terminated and hence able to yield turnover information. An additional 4,036 (heart), 3,904 (kidney), 5,326 (liver), and 4,952 (skeletal muscle) high-scoring arginine-terminated tryptic PSMs (at 1% FDR) gave a very high degree of confidence in assignment of *k*_deg_ values to individual protein identifications, as did an extra 994 (heart), 935 (kidney), 1,155 (liver), and 2,314 (skeletal muscle) dilabeled, miscleaved peptides (including peptides containing uncleaved LysPro sequences) identified (at 1% FDR). These additional, high-scoring dilabeled and arginine-terminated tryptic peptides contributed to the assignment of protein identity.

To assess the feasibility of using the mouse database to gain identification of bank vole proteins, we compared Mascot searches of muscle preparations from either bank vole or the house mouse (*Mus musculus domesticus*). Several analyses of these comparative searches were reassuring. The distributions of protein and peptide scores were comparable, and the same peptides generated similar scores from either species (Supplemental Fig. S1*A*). Clearly, the same proteins from both tissues were similarly identified, perhaps unsurprising given the evolutionary proximity of the bank vole and the house mouse, which diverged ∼30 million years ago (29–31). Moreover, “housekeeping” proteins, such as those present in skeletal muscle, do not evolve quickly, rendering cross-species matching more feasible (32). The distribution of Mascot protein or PSM scores were also rather similar. Additionally, PSMs that were below a 1% FDR were similar for either source tissue, indicating no major difference in performance of the Mascot algorithm with the “correct” (mouse source:mouse database) pairing or the “incorrect” (bank vole source:mouse database) comparison. We are highly confident that all proteins have been correctly identified by reference to their house mouse cognates. The cross-species matches obtained for all four tissues were of high quality, and the sequence coverage was essentially the same for each tissue in the vole, (median coverage—heart: 18%, kidney: 16%, liver: 19%, and skeletal muscle: 16%) and, in turn, very comparable to the coverage obtained for the mouse skeletal muscle analysis (median coverage: 19%) (kernel density plots for all five analyses in Supplemental Fig. S1*B*). Furthermore, the repeated observation of unlabeled and [^13^C_6_]lysine-labeled peptides across a labeling curve gave added reassurance of the consistency of the identifications. We were therefore confident about assigning the peptides to proteins and, from the temporal pattern of labeling, in the recovery of the turnover rate of the protein. The growth in the experimental subjects during the labeling period was small (less than 2% increase in body mass over the experiment), and we have not corrected for the very minor pool expansion during this time.

##### Measurement of Protein Turnover Rates in Different Tissues

**Fig. 2.**
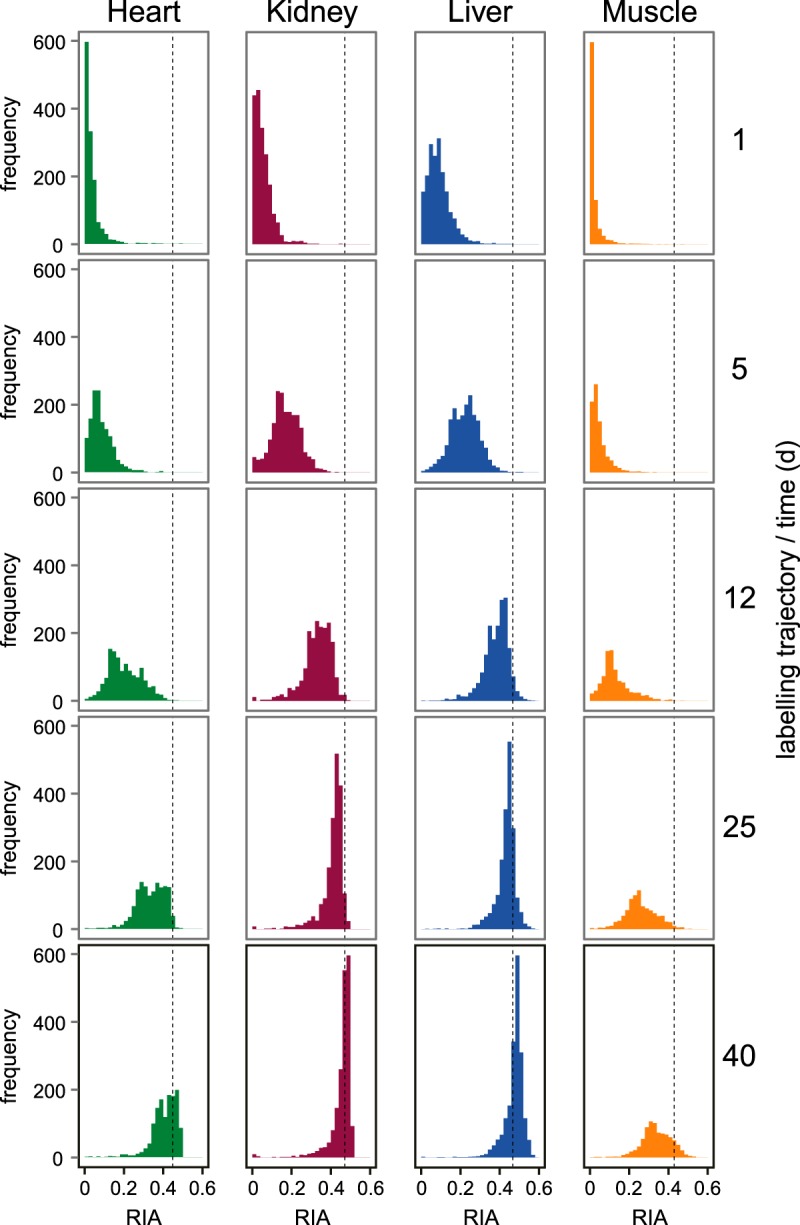
**Tissue-dependent changes in relative isotope abundance of peptides.** For all high-quality lysine-labeled peptides in each of the four tissues at different labeling times, *t*, the relative isotope abundance (RIA_t_) was calculated as the H/(H+L) ratio. The overall changes in RIA_t_ profile have been plotted as a distribution for each of the tissues over the 40 days of the labeling experiment, demonstrating the transition from largely unlabeled to extensively labeled peptides as a consequence of protein turnover. The dotted lines refer to the RIAp for each tissue.

To assess the turnover rate of individual proteins over a 40-day labeling period, four tissues (liver, kidney, heart, and skeletal muscle) were recovered from 10 individuals. Protein preparations from each tissue were subject to trypsin digestion, and the peptides were analyzed by LC-MS/MS from two animals at each of five different times (days 1, 5, 12, 25, and 40) during the labeling experiment. Although many proteins were identified, only those containing one or more mono-lysine peptide(s), readily analyzable across the whole time course of the experiment, were included in this analysis (example in Supplemental Fig. S2). The number of confidently identified, individual, single label-containing peptides that could be mapped to LC-MS features across all time points in the study and thus were able to allow turnover rate calculations were 654 (heart), 609 (kidney), 1,068 (liver), and 907 (skeletal muscle). When grouped into proteins, this equated to very high confidence *k*_deg_ measurements for 300 [125] proteins in the heart, 347 [124] in kidney, 448 [209] in liver, and 358 [152] in skeletal muscle; numbers in brackets represent protein *k*_deg_ measurements that are based on RIA_t_ values from multiple temporally separated measures of a single peptide. While these are small in number compared with the global tissue proteomes, we have set very high threshold criteria to ensure high-quality data, as examination of the supplementary data will attest. We appreciate that we cannot extrapolate to the entire proteome for each tissue, although we have derived high-quality turnover data for the highly abundant proteome in this study in numbers that are not radically different from other proteome studies. The labeling of proteins in each tissue was progressive and variable across tissues; the RIA shift in liver and kidney peptides was relatively rapid, and many peptides had reached maximal RIA_inf_ labeling at the end of the experiment (all protein data are included in Supplemental Fig. S3). By contrast, the increase in RIA_t_ for heart and skeletal muscle peptides was notably slower, and relatively few peptides had attained RIA_inf_ at 40 days, consistent with an overall lower rate of turnover (Fig. 2).

For all four tissues, the rate at which some proteins acquired heavy label was sufficiently high to allow the trajectory of RIA_p_ to be accurately assessed over the entire labeling period; by analysis of di-lysine peptides from multiple proteins and peptides (Supplemental Fig. S4 and Supplemental Table S1). In all four tissues, multiple di-lysine peptides were identified, either due to an uncleavable LysPro sequence or tryptic miscleaves. From these peptides, we were able to determine the RIA_p_ of each tissue from 12 (heart), 10 (kidney), 20 (liver), and 14 (skeletal muscle) dilabeled peptides. Because the RIA calculation is made only by internal self-reference between labeled and unlabeled variants, it is independent of the quantity of the di-lysine peptide, and if the di-lysine peptide is due to a miscleavage, even the extent of miscleavage does not have to be reproducible. The rise to the RIA_inf_ plateau is consistent with an efficient utilization of both free and protein-bound amino acids in the ingested food and an effective delivery of lysine from the gut lumen into the tissues. The slightly higher values for liver and kidney may reflect the proximity to the portal vein input from the gut and the relatively high metabolic rate of these tissues.

**Fig. 3.**
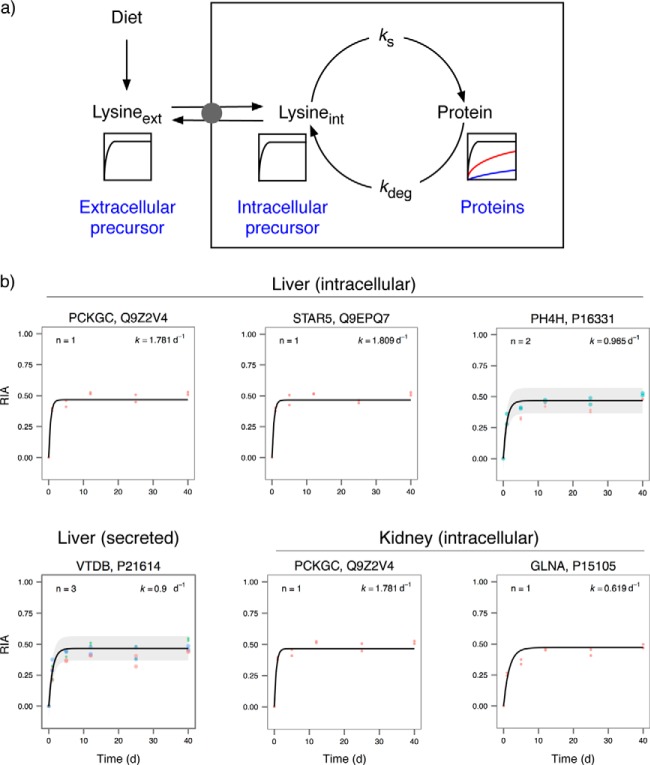
**Equilibration of lysine within tissue pools.** Dietary lysine crosses the intestine and enters the circulation, where it equilibrates with tissue free amino acid pools (*panel A*). Synthesis of lysine is not possible in mammals, and degradation of lysine occurs via the saccharopine pathway to yield the ketogenic product glutaryl CoA. The isotope enrichment (relative isotope abundance, RIA) of the lysine is obtained by sampling peptides that contain more than a single instance of the amino acid (see text). This allows tracking of precursor RIA. Further, high turnover proteins are most effective at sampling the RIA of the precursor pool and thus, report on the 'worst case' isotope enrichment profile (*panel B*). These can be secreted proteins for which no intracellular pool exists to dilute/delay acquisition of label (vitamin D binding protein, VTDB) or proteins that are intrinsically high turnover, such as phosphoenolpyruvate carboxykinase (PCKGC), StAR-related lipid transfer protein 5 (STAR5), or glucokinase (GLNA). The very high rate of acquisition of plateau RIA values (RIA_inf_ ∼0.5) is proof of the suitability of the simple two-pool model of amino acid cycling through the protein pool.

Further evidence for the speed of equilibration of the dietary amino acid with the precursor pool can be derived from examination of the behavior of secreted proteins or intracellular proteins that have very high turnover rates (Fig. 3). For phosphoenolpyruvate carboxykinase (PGKGC, accession Q9Z2V4), a high turnover protein, labeling was extremely fast, reaching a plateau RIA of 0.5 within the earliest time points. Similar data were obtained for secreted proteins, such as vitamin D binding protein (VTDB, P21614). In the case of secreted proteins that are immediately secreted from the cell, there is no intracellular protein pool to dilute the RIA of the newly synthesized proteins. The trajectory of labeling of this protein is also extremely rapid. We use the behavior of these proteins to establish that the dietary lysine equilibrates rapidly with the intracellular pools, obviating the addition of inaccessible intermediate pools in a multicompartment model that would not alter the kinetics of label incorporation.

Once the value of RIA_inf_ was derived, the pattern of labeling of mono-lysine peptides was analyzed to acquire the rate of turnover of each protein. Raw LC-MS chromatographic features corresponding to the same peptide on each aligned chromatogram were analyzed at each time point and for each of the duplicate animals. For all corresponding proteins in the different tissues, the lysine RIA_t_ would increase from 0 to RIA_inf_ at a rate dependent on turnover (synonymous with replacement since the animals are not growing and there is negligible pool expansion). High turnover proteins would therefore label rapidly, whereas for low turnover proteins RIA_t_ would increase more slowly. For any protein, each lysine-terminated peptide would be independently informative about the speed of labeling, and where possible, data were combined from multiple peptides to yield a true protein turnover rate. As a further check of the reliability of extraction of the entire peptide (H and L) at each time point, we compared the H+L intensity (duplicates averaged) over the time course of the experiment. The total of H+L remained constant throughout the study, irrespective of the shift between the H and L pools, consistent with efficient quantification of the labeled and unlabeled peptides. Moreover, the correlations of the H+L protein pool sizes (expressed as summed intensity from label-free quantification) were remarkably consistent throughout the study (Supplemental Fig. S5). Thus, the analytical approach was effective at capturing the entire protein pool (labeled and unlabeled) throughout the time course of the experiment.

The degree of labeling of mono-lysine peptides, taken as RIA_t_ = H/(H + L), was plotted against time and analyzed by nonlinear curve fitting to derive the first-order rate constant for degradation, *k*_deg_. The second parameter in the nonlinear equation/model, the value of RIA at plateau (RIA_inf_), was set as a constant value corresponding to the tissue-specific plateau RIA_inf_ calculated using a self-starter nonlinear function for the tissue precursor pool (RIA_p_). For heart, this value was 0.448 ± 0.023 (plateau value ± standard error), for kidney it was 0.471 ± 0.018, for liver, 0.467 ± 0.022, and for skeletal muscle, 0.428 ± 0.043 (Supplemental Fig. S4).

Very stringent criteria were applied to restrict the dataset to high-quality turnover measurements, defined as a distribution of points over the entire labeling window that tracked a monoexponential rise to plateau. Such behavior was obtained for a total of 3,239 peptides over the four tissues (Supplemental Table S2, 1 sheet per tissue). After grouping multiple peptides from the same protein, the dataset reduced to 808 proteins (Supplemental Table S3; 1 sheet per tissue, Supplemental Table S4). The variance of the *k*_deg_ values (S.E./*k*_deg_ for each protein × 100) were 7.4% (heart), 3.4% (kidney), 4.5% (liver), and 9.6% (skeletal muscle). The labeling curves for every protein are included in supplementary information (Supplemental Fig. S3), discriminating between those proteins for which turnover rates were acquired by multiple peptides and those where *k*_deg_ was obtained from a single peptide. Note that this does not mean that the *identification* was based on a single peptide. Using such stringent acceptance criteria, single peptides were equally capable of yielding high-quality degradation rates, although it should be noted that virtually all proteins were identified by more than three peptides, even if only a single lysine-terminated peptide was used for turnover analysis. No turnover data have been presented in this paper for identifications based on a single peptide, although such values exist in the dataset. However, we include turnover data based on single lysine-terminated peptides where additional peptides were used for identification.

##### Comparison of Turnover between Four Tissues

**Fig. 4.**
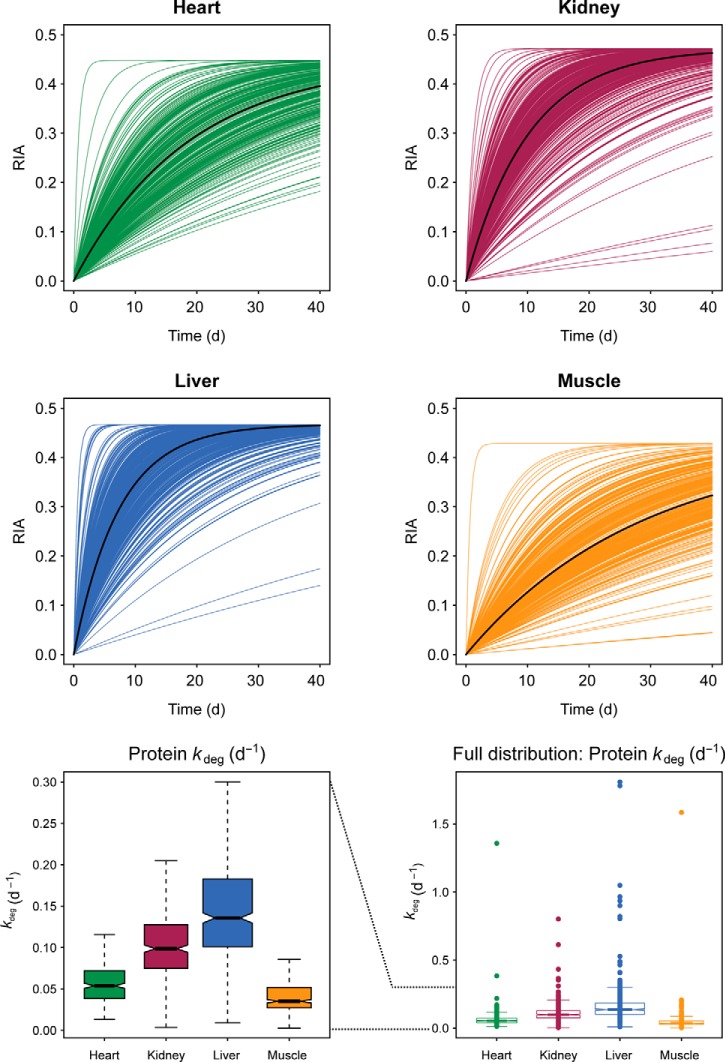
**Overall tissue turnover profiles.** For each tissue, and each protein, first-order curve fitting was used to derive the degradation rate constant (*k*_deg_). For each protein, the turnover curve was reduced to a smooth first-order labeling curve—for ease of interpretation data points were omitted (see Supplemental Fig. S3 for individual curves and Supplemental Table S2 for raw (RIA, t) data) and the aggregated tissue data were plotted (*top* four panels). The median turnover behavior is illustrated with a solid black line. The degradation rate constants were not normally distributed, and the distribution of degradation rates (all in days^−1^) is presented as a box and whiskers plot in the *lower left panel* (heart: min = 0.013, lower quartile = 0.038, median = 0.054, upper quartile = 0.072, max = 0.116; kidney: min = 0.003, lower quartile = 0.075, median = 0.099, upper quartile = 0.128, max = 0.205; liver: min = 0.0089, lower quartile = 0.1010, median = 0.1357, upper quartile = 0.1829, max = 0.300; muscle: min = 0.003, lower quartile = 0.027, median = 0.035, upper quartile = 0.052, max = 0.086). Additional extreme *k*_deg_ values are presented in the *lower-right panel*.

The rate of replacement of individual proteins can be aggregated into a family of labeling curves that summarizes the turnover behavior in each tissue (Fig. 4, for clarity, data points have been removed, but all individual curves including data points are presented in Supplemental Fig. S3). The turnover profile for each tissue was expressed by plotting the theoretical curve for each protein based on the fitted parameters of *k*_deg_ and RIA_inf_. As we first observed (17), the distribution of *k*_deg_ does not pass normality tests (Shapiro–Wilk test, *p* < 6 × 10^−11^ in all four tissues), and a nonparametric display of the turnover values emphasized the tissue differences (Fig. 4). From these profiles, it is immediately evident that kidney and liver (median *k*_deg_ of 0.099 days^−1^ and 0.136 days^−1^, respectively) have a higher rate of protein turnover than cardiac muscle (median *k*_deg_ = 0.054 days^−1^), which in turn has a higher rate of turnover than skeletal muscle (median *k*_deg_ = 0.035 days^−1^). The range of *k*_deg_ values that can be measured are largely restricted by the ability to obtain a first-order curve that is well defined by the time points. Thus, a protein that reaches 95% of RIA_inf_ within the first time point (1 day) would effectively be completely labeled, suggesting an upper limit of quantifiable *k*_deg_ = 3.0 days^−1^ (half-life approximately 0.25 days). Similarly, a protein that had achieved only 5% of RIA_inf_ at 40 days would have a *k*_deg_ = 0.0014 days^−1^ (half-life approximately 500 days, close to the lifespan of the animal). However, values at those *extrema* would have very high errors and have not been included here. This relationship between the four tissues is in broad agreement with previous work from a small mammal of similar body mass, the house mouse (16).

**Fig. 5.**
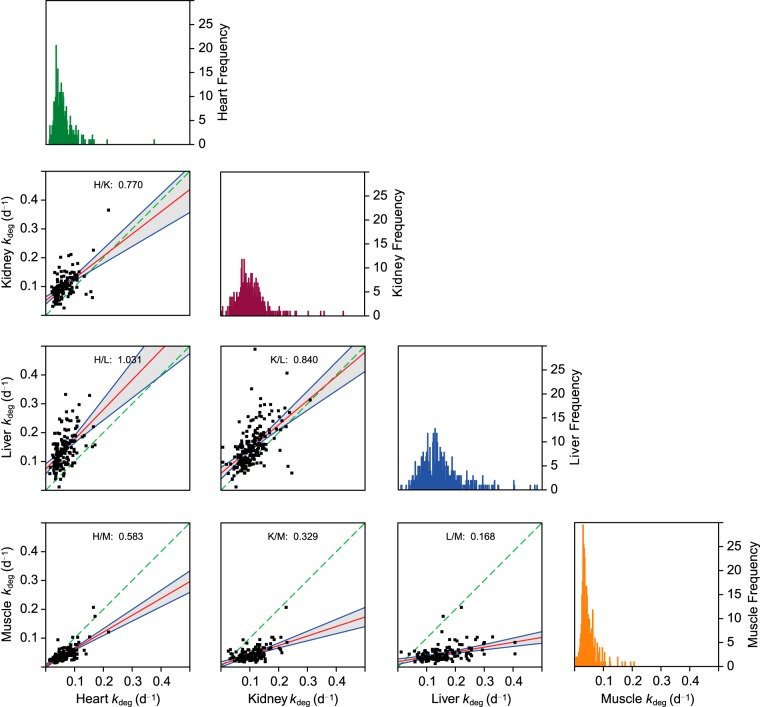
**Protein-specific comparison of degradation rates between tissues.** For the subset of proteins that were expressed in more than one tissue, it was possible to construct scatter plots to compare the overall degradation rate differences between tissues, using a restricted range of 0 to 0.5 days^−1^ that contained the majority of proteins. The red line is the linear regression of the relationship, and the shaded area defines the 95% confidence limits for the regression line. The green dashed line is the line of equivalence, upon which the degradation rates for one tissue would be equivalent to the second. *Inset* on the diagonal are the distributions of degradation rate for each tissue. Within each panel the ratio provided is the slope of the linear fit line.

The intrinsic differences in tissue-specific median *k*_deg_ could be due to changes in the activity of the degradative machinery between tissues, affecting all proteins equally. Alternatively, proteins might have evolved to have an intrinsically altered rate of entry into the degradative process. Given the differences in median degradation rate in different tissues, it was instructive to examine the degradation rate for the same gene product expressed in more than one tissue. For a limited subset of proteins it was possible to explore this relationship. Pairwise linear regression analyses between the tissues (six analyses in total, Fig. 5) revealed the consistency of tissue differences, with heart and skeletal muscle forming a cluster and kidney and liver forming a separate cluster. Thus, the differences between tissues are not solely attributable to the different protein profiles; the same proteins in different tissues also reflected the overall tissue behavior. Indeed, for the restricted subset of 67 proteins for which we calculated *k*_deg_ for all four tissues, the rank order of turnover rate for each protein was sustained across four tissues (Kendall's W = 0.69, *n* = 4, *p* < 0.0005). Such data lead to the conclusion that the primary determinant of the overall rate of degradation is the tissue-specific degradative environment. This is likely to reflect the commitment step that directs a protein to rapid degradation. It is less clear that the proteolytic completion step would need to be similarly adjusted to produce tissue-specific degradation rates—this would be unnecessary if the commitment stage is rate-limiting.

Within the entire dataset, some patterns emerge. First, as no tissues were perfused to remove blood and thus contained blood proteins (alpha and beta chains of hemoglobin, serum albumin, serotransferrin, fibrinogen, etc.), reassurance of the relevance of the tissue-specific differences in turnover derives from the labeling profiles for hemoglobin: In all four tissues and for each subunit, the rate constants (in days^−1^) for the hemoglobin alpha chain were very similar (0.033, 0.039, 0.039, 0.044; for heart, kidney, liver, and skeletal muscle, respectively). The average of these values is 0.039 days^−1^, which, because of the lifetime kinetics of the erythrocyte and the fact that hemoglobin is not degraded in the red blood cell, indicates the replacement of approximately 4% of the erythrocytes per day, giving a lifespan of 25 days for these cells—a value that is somewhat lower than some (33) but similar to other (34) reported values for the mouse red blood cell. Turnover data for serotransferrin were obtained from three tissues—as expected a similar value was obtained in all three analyses (0.170, 0.157, 0.176 days^−1^ for heart, liver, and skeletal muscle, respectively). This gives a half-life of approximately 4 days, longer than the reported value of 1.4 days for radio-iodinated, exogenously added transferrin (35); this might, however, reflect a differential stability of the modified protein. Interestingly, the apparent replacement rate of albumin varied to a greater extent from tissue to tissue. The turnover rate for heart was lower (0.165 days^−1^) than the rates for kidney, liver, and skeletal muscle (0.226 days^−1^, 0.220 days^−1^, and 0.208 days^−1^, respectively). The implication is, first, that albumin has a half-life in bank vole plasma of approximately 3 days, slightly longer than reported values for the mouse (36) and, secondly, that the lower rate of replacement of the cardiac albumin pool might reflect a slower exchange of interstitial albumin with the circulatory pool.

##### Comparison with Turnover Data from the Mouse

**Fig. 6.**
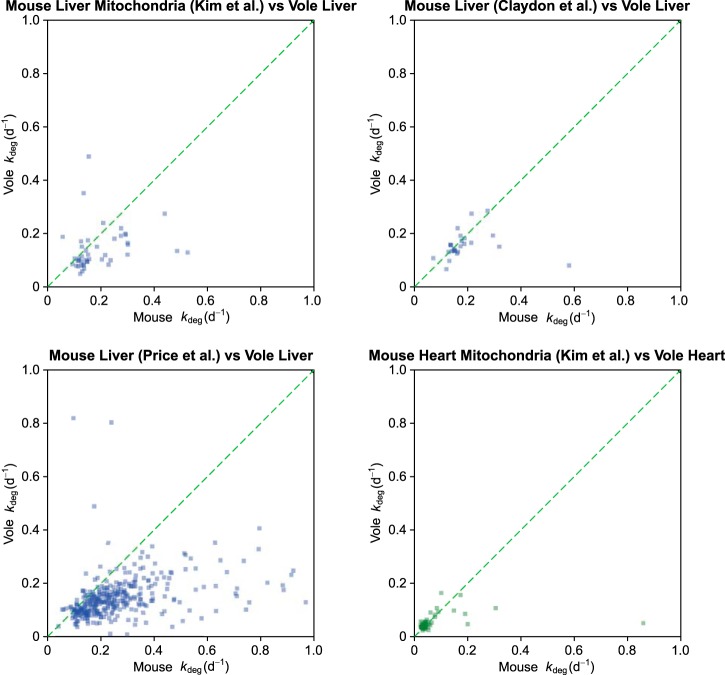
**Comparison of turnover measurements from different studies.** The bank vole degradation profile was compared for liver proteins derived from three additional studies (see text) that used different labeling protocols and analytical approaches. For each study, the calculated *k*_deg_ values were restricted to a common scale between 0 and 1 day^−1^ (half-life up to 0.7 days). The diagonal dotted line is the line along which the *k*_deg_ values would be equivalent.

The bank vole is a small rodent similar in body mass to the house mouse and with a similar metabolic rate (37). It was therefore reasonable to compare the overall degradation profile with datasets derived from the house mouse. The first of these datasets was based on dietary administration of the essential amino acid [^2^H_8_]valine as a label ((16), heart, kidney, liver, skeletal muscle), the second on an algal-derived universally ^15^N labeled diet ((14), brain, liver), and the third an administration of [^2^H_2_]O supplemented drinking water ((8), heart and liver mitochondria). The only tissue common to all four studies was liver, and so the comparison was restricted to this tissue (although we were also able to compare turnover of heart proteins with the Kim *et al.* study), given the notable variation in tissue-specific overall degradation profile of proteins (see this and previous studies). In addition, we have restricted the comparison to proteins with reported *k*_deg_ values of the same order as the restricted range we have allowed in our analysis (Fig. 6).

For example, in the study of Price *et al.* (14) *k*_deg_ values as high as 65 days^−1^ were reported. However, according to the experimental design of that study, the first sampling time was 0.4 days. From turnover kinetics, a protein with such a high degradation rate would have been fully labeled (>99%) at the very first time point, and thus, estimation of an accurate value for *k*_deg_ would be impossible. This protein, accession number P11588, is in fact a major urinary protein that is synthesized in the liver but immediately secreted, and as a consequence, there is no unlabeled pool to replace; the degree of labeling would be substantial, even at the first sampling point (see our argument about precursor labeling above). Indeed, we have used the major urinary proteins for noninvasive sampling of the precursor labeling behavior in liver, including this study, and have revealed a strong diurnal effect in synthesis (16). The labeling time for the Price *et al.* study was from 0 to 32 days, similar in scope to our investigation, and it is likely that only the values in the range that we have reported would be reliable. For the Kim *et al.* study, there were virtually no reported *k*_deg_ values that would be compromised by the sampling regimen (which extended from 0.5 days to 90 days). However, by restricting the range of all studies to a *k*_deg_ range from 0 to 1.0 days^−1^, we still include 49% of our protein data, 31% of the liver data from Price *et al.*, and 11% of the liver data from Kim *et al.* (also 20% of the heart data from the Kim *et al.* study).

The correlation diagrams for the four studies indicate a degree of concordance; high turnover proteins tend to have high turnover rates in all studies. However, the correlation is noisy, and in the case of the Price *et al.* study, there is evidence of a systematic bias in the relationship between the two tissues; proteins are reported as turning over about twice the rate as in this bank vole study, whereas for the other comparisons, the rates of degradation are comparable. Given the different labeling regimens and the analytical approaches being used, it is possible that systematic biases might pervade turnover studies. Not all study comparisons yielded the same bias in measurement, which makes the explanation of a species-related difference less probable.

##### Analysis of Tissue-Specific Protein Flux

**Fig. 7.**
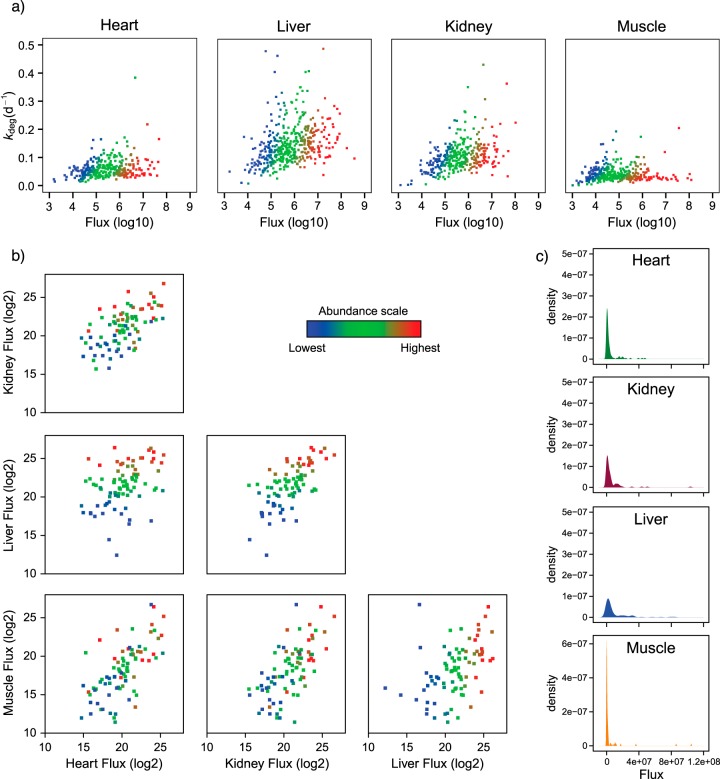
**Relationship between the flux of protein molecules through the protein pool and their abundance (H+L label-free measurement).** A surrogate measurement of flux through the protein pool was determined by calculating *k*_deg_.[P], the product of the mean label-free abundance of each protein (using lysine terminated peptides only) and the measured *k*_deg_ value. The *top panels* (*A*) describe the relationship between flux and *k*_deg_, with symbols color coded from low abundance proteins (blue) to high abundance proteins (red). The distribution of flux values is presented in (*B*). For each of the 68 proteins common to all four tissues, flux calculations were compared for all tissues, again color coded by label-free abundance (*C*).

While *k*_deg_ gives a measure of the commitment of protein to the degradative process, it does not *per se* give a measure of the flux of molecules through the protein pool. The flux through a protein pool of magnitude [P] is expressed as *k*_deg_.[P] with an additional growth term for pool expansion if the system is actively increasing biomass, whereas *k*_deg_ gives a different picture of the dynamics of protein turnover within any tissue. While we lacked absolute abundances of individual proteins, we used label-free quantification values as a measure of abundance; these values were remarkably consistent across the time course, irrespective of the degree of labeling (Supplemental Fig. S5). We used the average of 10 independent samples for each abundance measure. The consistency of the recovered abundances (a sum of the labeled and unlabeled pools) was also an independent reassurance of the ability of our analytical and data processing workflow to capture all of the peptide intensity, irrespective of whether it has incorporated label. The product of this abundance measure and the first-order rate constant (= *k*_deg_.[P]) yielded a surrogate measurement of flux through each protein pool (Fig. 7).

For each tissue, the flux measurements spanned five to six orders of magnitude, whereas the turnover rates spanned a range of no more than sixfold. The implication of this is that the major contributor to flux is the abundance of the protein pool, not the intrinsic rate of turnover, although close examination of Fig. 7*A* indicates that there are some abundant proteins that are among the highest turnover proteins in each tissue while some low abundance proteins also are very long lived. Within each tissue, the behavior was notably different, reflecting the activity of the degradative machinery in each tissue, although the general trend was similar. In heart and skeletal muscle, the rate of turnover was largely decoupled from flux, but in liver and kidney, the highest flux proteins also exhibited the highest turnover rates, although individual protein variability was noticeable. For the proteins that were common to two tissues, the flux values were in broad agreement, although the relationship was not as strongly linked to protein abundance (Fig. 7*B*), reflecting differences in protein abundance between tissues and the major contribution of pool size in the flux calculation. The overall distribution of flux through the protein pool was substantially lower in muscle and heart compared with kidney and liver (Fig. 7C), commensurate with the overall activity of the degradative apparatus in the different tissues.

##### Relationship between Turnover Rates Derived from Cell Culture and Intact Animals

Several proteome turnover studies have been conducted in cells grown in culture (reviewed in (11)). Accordingly, we were also able to address the relationship between tissue turnover and that measured in isolated cells that are delivered optimal nutrients and that can divide very quickly. In our own studies with *Saccharomyces cerevisiae* and mammalian cells, we noted that degradation rates for many proteins were extremely low, consistent with a rapidly expanding protein pool linked to high growth rates as a consequence of a high nutritional plane from rich media, supplemented in some instances by serum-derived growth factors (4, 17). Indeed, many proteins exhibited very little degradation relative to the rate of pool expansion caused by culture growth—these proteins are effectively “immortal.”

**Fig. 8.**
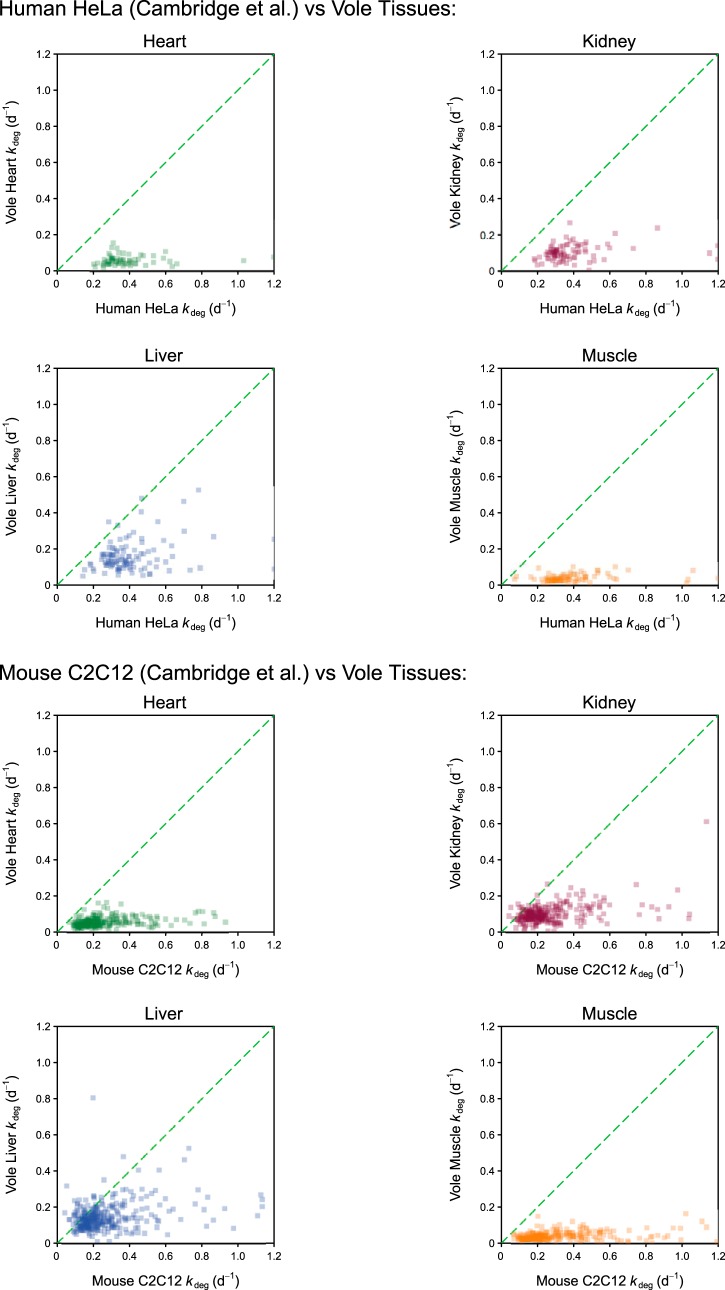
**Comparison of degradation rates for proteins in tissues and cultured cells.** The *k*_deg_ values from vole tissues were compared with published data from two cell culture studies. The diagonal dotted line defines the equivalence of *k*_deg_ values. Note that it was necessary to convert the published values from h^−1^ to days^−1^ for comparison.

One of the larger datasets for mammalian protein turnover derived from the analysis of arrested HeLa or mouse C2C12 cells (38). In both of these cell types, rapid cell division was stopped, and thus, turnover measurements would be acquired in the absence of growth. Surprisingly, the turnover rates for proteins derived from these cells were substantially higher than those from tissues (Fig. 8). Although protein turnover in humans is considered to be much lower than in smaller mammals (26), the rates of protein turnover for specific proteins in HeLa cells are much higher than their cognates in vole intact tissues. The same behavior was evident in a comparison with the second dataset from mouse C2C12 cells where the proteins were turned over at remarkably high rates compared with the intact animal. Why such arrested cells should activate protein degradation to this extent is unclear, although the possibility exists of an enhanced hypercatabolic response, perhaps associated with the need for protein complement remodeling in response to the change in nutrient availability. We suggest that while such cell-culture-based studies can generate samples that are suitable for comparison of relative degradation rate, caution should be applied in ascribing significance to the absolute values for protein replacement. It is possible that the normal proteostatic processes that operate in a tissue are differently regulated to allow for the rapid response of the protein pool in culture conditions. In this regard, there may be a fundamental difference between simple organisms such as *S. cerevisiae*, where protein degradation appears to be almost completely suppressed during rapid growth, and mammalian cells, a discrepancy that warrants closer attention.

## DISCUSSION

This study has confirmed that simple dietary supplementation with an essential stable isotope labeled amino acid can be used to recover turnover rates from many proteins and in different tissues. In this study, we used [^13^C_6_]lysine, but there is no reason why this could not be made more affordable by using a deuterated amino acid (such as [^2^H_7_]lysine, avoiding the alpha carbon deuteron that is metabolically labile through transamination). The labeling patterns obtained with a single, essential amino acid are extremely simple and amenable to analysis using readily deployed tools, yielding high-quality measurements of turnover rate.

Although the annotated proteome for the bank vole is not yet available, we believe that the extent of cross-species matching to other *Rodentia* is sufficiently high that, for many proteins, it is acceptable to infer the identity on the basis of multiple matched peptides. We have recently demonstrated the surprising robustness of cross-species matching, using sperm proteins and relating protein identifiability with evolutionary rate (39).

The stringency of the database match is one factor in recovery of high-quality turnover rates. The second is the challenge of mapping kinetic data (RIA as a function of time) across the labeling period. In this study, we have set a high threshold, requiring that we have peptide RIA data for every time point in the labeling protocol. It is undoubtedly possible to relax this requirement, but the ability to reduce the number of data points is context dependent and has to be related to the intrinsic turnover rate of the protein. We are confident that the highly robust filtering of the measured turnover values adds to the quality of our dataset. Lastly, although this is the first study of proteome dynamics in the adult bank vole, the previously published variation in correlation between different studies on bank vole or mouse requires attention. This may be in part an outcome of the data analysis strategy, but it is also possible that the different labeling protocols are yielding systematically different measures of intracellular stability. Should this be the case, it follows that there is a growing need for a critical analysis that compares and converges labeling protocols and bioinformatics. Without a full understanding of the sources of variance in these analyses, more exhaustive comparative analyses may prove infeasible. In particular, it is possible that the rapid growth of cultured cells results in an effective decoupling from the normal, tissue-specific processes of proteostasis. There are many studies that have intimated that the intrinsic rate of degradation of a protein may also be highly species specific (26); larger animals have slower turnover systems. It is thus not possible to cite a single value for intracellular stability of any one protein across multiple systems. The true values are of course those obtained from the intact animal or plant, the most inaccessible in terms of labeling protocols. This is exacerbated by the application of mass spectrometry-based measurement of isotope incorporation. Early studies used radioisotope labeling in which the specific radioactivity of the precursor (usually [^14^C], [^35^S] or [^3^H]) was so high that vanishingly small degrees of isotope incorporation could be detected by scintillation counting. Stable isotopes, as used in the context of proteomics derived turnover studies, require the detection of a confidently measurable incorporation of label compared with the cognate peptides, which typically requires a level of incorporation of at least 5% of the signal from the unlabeled material. Inevitably, this demands extended labeling protocols for all but the highest turnover proteins, increasing the complexity and indeed, the cost of the studies.

Acquiring high-quality turnover data is nontrivial. There is a need for a central repository of turnover data (both raw data and processed values) that can be explored and built into systems models. Moreover, in many studies that use plant or animal tissues, there is every possibility that residual, dynamically labeled tissue remains—tissue that could be readily shared with other groups with similar interests. In the case of animal studies, this also resonates well with the requirement to reduce the number of animals used in research, as well as ameliorate the cost of expensive labeling protocols. We would be more than willing to discuss sharing of such material with other research groups.

## Supplementary Material

Supplemental Data

## Footnotes

^1^ The abbreviations used are:

RIA: relative isotope abundance
RIA_t_: relative isotope abundance at time t
RIA_inf_: relative isotope abundance at plateau (“infinity”)
RIA_p_: relative isotope abundance of precursor pool
LC-MS/MS: liquid chromatography–tandem mass spectrometry.

## References

[1] Cargile B. J., Bundy J. L., Grunden A. M., and Stephenson J. L. Jr. (2004) Synthesis/degradation ratio mass spectrometry for measuring relative dynamic protein turnover. Anal. Chem. 76, 86–97 14697036 10.1021/ac034841a

[2] Vogt J. A., Hunzinger C., Schroer K., Hölzer K., Bauer A., Schrattenholz A., Cahill M. A., Schillo S., Schwall G., Stegmann W., and Albuszies G. (2005) Determination of fractional synthesis rates of mouse hepatic proteins via metabolic ^13^C-labeling, maldi-tof ms and analysis of relative isotopologue abundances using average masses. Anal Chem 77, 2034–2042 15801735 10.1021/ac048722m

[3] Papageorgopoulos C., Caldwell K., Shackleton C., Schweingrubber H., and Hellerstein M. K. (1999) Measuring protein synthesis by mass isotopomer distribution analysis (mida). Anal. Biochem. 267, 1–16 9918649 10.1006/abio.1998.2958

[4] Pratt J. M., Petty J., Riba-Garcia I., Robertson D. H., Gaskell S. J., Oliver S. G., and Beynon R. J. (2002) Dynamics of protein turnover, a missing dimension in proteomics. Mol. Cell. Proteomics 1, 579–591 12376573 10.1074/mcp.m200046-mcp200

[5] Doherty M. K., and Beynon R. J. (2006) Protein turnover on the scale of the proteome. Expert Rev. Proteomics 3, 97–110 16445354 10.1586/14789450.3.1.97

[6] Busch R., Kim Y. K., Neese R. A., Schade-Serin V., Collins M., Awada M., Gardner J. L., Beysen C., Marino M. E., Misell L. M., and Hellerstein M. K. (2006) Measurement of protein turnover rates by heavy water labeling of nonessential amino acids. Biochim. Biophys. Acta 1760, 730–744 16567052 10.1016/j.bbagen.2005.12.023

[7] Rachdaoui N., Austin L., Kramer E., Previs M. J., Anderson V. E., Kasumov T., and Previs S. F. (2009) Measuring proteome dynamics in vivo: As easy as adding water? Mol. Cell. Proteomics 8, 2653–2663 19724074 10.1074/mcp.M900026-MCP200 PMC2816015

[8] Kim T. Y., Wang D., Kim A. K., Lau E., Lin A. J., Liem D. A., Zhang J., Zong N. C., Lam M. P., and Ping P. (2012) Metabolic labeling reveals proteome dynamics of mouse mitochondria. Mol. Cell. Proteomics 11, 1586–1594 22915825 10.1074/mcp.M112.021162 PMC3518123

[9] Chan X. C., Black C. M., Lin A. J., Ping P., and Lau E. (2015) Mitochondrial protein turnover: Methods to measure turnover rates on a large scale. J. Mol. Cell. Cardiol. 78, 54–61 25451168 10.1016/j.yjmcc.2014.10.012 PMC4746024

[10] Beynon R. J., and Pratt J. M. (2005) Metabolic labeling of proteins for proteomics. Mol. Cell. Proteomics 4, 857–872 15849272 10.1074/mcp.R400010-MCP200

[11] Claydon A. J., and Beynon R. (2012) Proteome dynamics: Revisiting turnover with a global perspective. Mol. Cell. Proteomics 11, 1551–1565 23125033 10.1074/mcp.O112.022186 PMC3518130

[12] Yee J. C., Jacob N. M., Jayapal K. P., Kok Y. J., Philp R., Griffin T. J., and Hu W. S. (2010) Global assessment of protein turnover in recombinant antibody producing myeloma cells. J. Biotechnol. 148, 182–193 20540971 10.1016/j.jbiotec.2010.06.005

[13] Doherty M. K., Whitehead C., McCormack H., Gaskell S. J., and Beynon R. J. (2005) Proteome dynamics in complex organisms: Using stable isotopes to monitor individual protein turnover rates. Proteomics 5, 522–533 15627957 10.1002/pmic.200400959

[14] Price J. C., Guan S., Burlingame A., Prusiner S. B., and Ghaemmaghami S. (2010) Analysis of proteome dynamics in the mouse brain. Proc. Natl. Acad. Sci. U.S.A. 107, 14508–14513 20699386 10.1073/pnas.1006551107 PMC2922600

[15] Doherty M. K., Brownridge P., Owen M. A., Davies S. J., Young I. S., and Whitfield P. D. (2012) A proteomics strategy for determining the synthesis and degradation rates of individual proteins in fish. J. Proteomics 75, 4471–4477 22484057 10.1016/j.jprot.2012.03.025

[16] Claydon A. J., Thom M. D., Hurst J. L., and Beynon R. J. (2012) Protein turnover: Measurement of proteome dynamics by whole animal metabolic labelling with stable isotope labelled amino acids. Proteomics 12, 1194–1206 22577021 10.1002/pmic.201100556

[17] Doherty M. K., Hammond D. E., Clague M. J., Gaskell S. J., and Beynon R. J. (2009) Turnover of the human proteome: Determination of protein intracellular stability by dynamic silac. J. Proteome Res. 8, 104–112 18954100 10.1021/pr800641v

[18] Beynon R. J., and Pratt J. M. (2006) Strategies for measuring dynamics: The temporal component of proteomics. Methods Biochem. Anal. 49, 15–25 16929670 10.1002/0471973165.ch2

[19] Claydon A. J., Ramm S. A., Pennington A., Hurst J. L., Stockley P., and Beynon R. (2012) Heterogenous turnover of sperm and seminal vesicle proteins in the mouse revealed by dynamic metabolic labeling. Mol. Cell. Proteomics 11, M111.014993 22331477 10.1074/mcp.M111.014993 PMC3433909

[20] McClatchy D. B., Dong M. Q., Wu C. C., Venable J. D., and Yates J. R. 3rd (2007) ^15^N metabolic labeling of mammalian tissue with slow protein turnover. J. Proteome Res. 6, 2005–2010 17375949 10.1021/pr060599n PMC2527585

[21] Vizcaíno J. A., Deutsch E. W., Wang R., Csordas A., Reisinger F., Ríos D., Dianes J. A., Sun Z., Farrah T., Bandeira N., Binz P. A., Xenarios I., Eisenacher M., Mayer G., Gatto L., Campos A., Chalkley R. J., Kraus H. J., Albar J. P., Martinez-Bartolomé S., Apweiler R., Omenn G. S., Martens L., Jones A. R., and Hermjakob H. (2014) Proteomexchange provides globally coordinated proteomics data submission and dissemination. Nat. Biotechnol. 32, 223–226 24727771 10.1038/nbt.2839 PMC3986813

[22] Qi D., Brownridge P., Xia D., Mackay K., Gonzalez-Galarza F. F., Kenyani J., Harman V., Beynon R. J., and Jones A. R. (2012) A software toolkit and interface for performing stable isotope labeling and TOP3 quantification using progenesis LC-MS. OMICS 16, 489–495 22888986 10.1089/omi.2012.0042 PMC3437041

[23] Doherty M. K., McClean L., Edwards I., McCormack H., McTeir L., Whitehead C., Gaskell S. J., and Beynon R. J. (2004) Protein turnover in chicken skeletal muscle: Understanding protein dynamics on a proteome-wide scale. Br. Poult. Sci. 45, S27–28 15222351 10.1080/00071660410001698092

[24] Weibel E. R., Bacigalupe L. D., Schmitt B., and Hoppeler H. (2004) Allometric scaling of maximal metabolic rate in mammals: Muscle aerobic capacity as determinant factor. Respir. Physiol. Neurobiol. 140, 115–132 15134660 10.1016/j.resp.2004.01.006

[25] Schöch G., Topp H., Held A., Heller-Schöch G., Ballauff A., Manz F., and Sander G. (1990) Interrelation between whole-body turnover rates of RNA and protein. Eur. J. Clin. Nutr. 44, 647–658 1702054

[26] Waterlow J. C., Garlick P. J., and Millward D. J. (1978) Protein Tturnover in Mammalian Tissues and in the Whole Body, Elsevier/North-Holland Biomedical Press, Amsterdam, The Netherlands

[27] Hsieh E. J., Shulman N. J., Dai D. F., Vincow E. S., Karunadharma P. P., Pallanck L., Rabinovitch P. S., and MacCoss M. J. (2012) Topograph, a software platform for precursor enrichment corrected global protein turnover measurements. Mol. Cell. Proteomics 11, 1468–1474 22865922 10.1074/mcp.O112.017699 PMC3494182

[28] Guan S., Price J. C., Prusiner S. B., Ghaemmaghami S., and Burlingame A. L. (2011) A data processing pipeline for mammalian proteome dynamics studies using stable isotope metabolic labeling. Mol. Cell. Proteomics 10, M111.010728 21937731 10.1074/mcp.M111.010728 PMC3237081

[29] Horner D. S., Lefkimmiatis K., Reyes A., Gissi C., Saccone C., and Pesole G. (2007) Phylogenetic analyses of complete mitochondrial genome sequences suggest a basal divergence of the enigmatic rodent anomalurus. BMC Evol. Biol. 7, 16 17288612 10.1186/1471-2148-7-16 PMC1802082

[30] Honeycutt R. (2009) The Timetree of Life, Hedges S. B., and Kumar S., eds., Oxford University Press, Oxford, pp. 490–494

[31] Babik W., Stuglik M., Qi W., Kuenzli M., Kuduk K., Koteja P., and Radwan J. (2010) Heart transcriptome of the bank vole (*Myodes glareolus*): Towards understanding the evolutionary variation in metabolic rate. BMC Genomics 11, 390 20565972 10.1186/1471-2164-11-390 PMC2996923

[32] Ramm S. A., McDonald L., Hurst J. L., Beynon R. J., and Stockley P. (2009) Comparative proteomics reveals evidence for evolutionary diversification of rodent seminal fluid and its functional significance in sperm competition. Mol. Biol. Evol. 26, 189–198 18931385 10.1093/molbev/msn237

[33] Walker W. S., Singer J. A., Morrison M., and Jackson C. W. (1984) Preferential phagocytosis of in vivo aged murine red blood cells by a macrophage-like cell line. Br. J. Haematol. 58, 259–266 6477835 10.1111/j.1365-2141.1984.tb06084.x

[34] Fraser S. T., Midwinter R. G., Coupland L. A., Kong S., Berger B. S., Yeo J. H., Andrade O. C., Cromer D., Suarna C., Lam M., Maghzal G. J., Chong B. H., Parish C. R., and Stocker R. (2015) Heme oxygenase-1 deficiency alters erythroblastic island formation, steady-state erythropoiesis and red blood cell lifespan in mice. Haematologica 100, 601–610 25682599 10.3324/haematol.2014.116368 PMC4420209

[35] Chaudhury C., Kim J., Mehnaz S., Wani M. A., Oberyszyn T. M., Bronson C. L., Mohanty S., Hayton W. L., Robinson J. M., and Anderson C. L. (2006) Accelerated transferrin degradation in hfe-deficient mice is associated with increased transferrin saturation. J. Nutr. 136, 2993–2998 17116709 10.1093/jn/136.12.2993

[36] Nolte H., Hölper S., Selbach M., Braun T., and Krüger M. (2014) Assessment of serum protein dynamics by native silac flooding (silflood). Anal. Chem. 86, 11033–11037 25347402 10.1021/ac502883p

[37] Boratynski Z., and Koteja V. (2009) The association between body mass, metabolic rates and survival of bank voles. Functional Ecol. 23, 330–339

[38] Cambridge S. B., Gnad F., Nguyen C., Bermejo J. L., Krüger M., and Mann M. (2011) Systems-wide proteomic analysis in mammalian cells reveals conserved, functional protein turnover. J. Proteome Res. 10, 5275–5284 22050367 10.1021/pr101183k

[39] Bayram H. L., Claydon A. J., Brownridge P. J., Hurst J. L., Mileham A., Stockley P., Beynon R. J., and Hammond D. E. (2016) Cross-species proteomics in analysis of mammalian sperm proteins. J. Proteomics (in press) 10.1016/j.jprot.2015.12.027 26768581

