# Supplementary material for: Proteome Dynamics: Tissue Variation in the Kinetics of Proteostasis in Intact Animals
Source: Mol Cell Proteomics. 2016 Feb 1;15(4):1204–19. doi: 10.1074/mcp.M115.053488 (PMC4824850; doi:10.1074/mcp.M115.053488)
Supplement: Supplemental Data [file supp_15_4_1204__index.html]

Proteome dynamics: tissue variation in the kinetics of proteostasis in intact animals — Proteome Dynamics: Tissue Variation in the Kinetics of Proteostasis in Intact Animals — Rodent Tissue Protein Turnover — Supplemental Data 

# Proteome Dynamics: Tissue Variation in the Kinetics of Proteostasis in Intact Animals

## Supplemental Data

- Supp FS1 (.docx, 15 KB) - Index to Supp Figures and Tables
- Supp FS2 (.pdf, 1.0 MB)
- Supp FS2 (.pdf, 479 KB)
- Supp FS3 (.pdf, 15.1 MB)
- Supp FS4 (.pdf, 19 KB)
- Supp FS5 (.pdf, 11.9 MB)
- Supp FT1 (.xlsx, 29 KB)
- Supp FT2 (.xlsx, 784 KB)
- Supp FT3 (.xlsx, 223 KB)
