# Supplementary material for: Proteome Dynamics: Tissue Variation in the Kinetics of Proteostasis in Intact Animals
Source: Mol Cell Proteomics. 2016 Feb 1;15(4):1204–19. doi: 10.1074/mcp.M115.053488 (PMC4824850; doi:10.1074/mcp.M115.053488)
Supplement: Supplemental Data [file 10.1074_M115.053488_mcp.M115.053488-1.docx]

**Supplementary Figures and Tables.**

**Supplemental Figure S1.** Cross species matching of peptide database searches. Panel a) Mascot searches against a fully annotated UniProt mouse protein database, of muscle preparations from either bank vole or mouse, showed that common proteins from both tissues were similarly identified with comparable Mascot scores. The distribution of high quality (1% FDR) Mascot protein (top) or peptide spectral match (PSM, middle) scores were very similar. In addition, PSMs that were below a 1% FDR threshold were similar for either source tissue (bottom), indicating no major difference in performance of the Mascot search algorithm between the ‘correct’ mouse-mouse database paring, or the ‘incorrect’ bank vole-mouse database comparison. Panel b) Comparison of the sequence coverage for all database searches of vole samples against the mouse database. The vertical line defines the median coverage. In the bottom panel, we include the mouse muscle against mouse database profile for comparison. The matching of vole proteins to mouse proteome is robust and useable. We are highly confident that all bank vole proteins have been correctly identified according to the equivalent house mouse protein.

**Supplemental Figure S2.** Example MS spectra and labelling curves for the protein O70250 (PGAM2), which shows a differential rate of turnover in liver (high turnover) and muscle (low turnover).

**Supplemental Figure S3.** Labelling curves for all proteins in all four tissues. RIA, t, data for mono-lysine peptides, grouped on a per protein basis, were applied to a nonlinear model in R (see Materials and Methods). The number of peptides (n) and the predicted *k*_deg_ are shown within each panel. Point colours denote different peptide identities (where n > 1) and point size is indicative of Mascot peptide score, with smaller the points having higher Mascot peptide score (see notes in Supplemental Table S2 for details).

**Supplemental Figure S4.** Calculation of RIA infinity, for each tissue. (RIA, t) data for di-lysine peptides were applied to a self-seeding nonlinear model in R in order to calculate RIA infinity (see Materials and Methods). The parameter ‘Asym’ in the model summary text, contained within each plot panel, represents the horizontal asymptote and is the predicted value RIA infinity used in our determination of *k*_deg_ for mono-lysine peptides.

**Supplemental Figure S5.** Consistency of measurements of label-free abundances, expressed as the sum of the labelled and unlabelled pools, across the time course of labelling, for each tissue (see Supplemental Table S5). The matrix plots show how well the proteomes of each tissue correlate in pairwise comparisons. For comparison, on the last page, scatterplots are provided for the label free quantification based on either lysine-terminated peptides or lysine- and arginine-terminated peptides –the correlation is high and the abundance values are essentially identical.

**Supplemental Table S1.** RIA, t, data for di-lysine peptides, applied to a self-seeding nonlinear model in R in order to calculate RIA infinity (one sheet per tissue).

**Supplemental Table S2.** Proteolabels output of all peptide RIA, t, data (one sheet per tissue).

**Supplemental Table S3.** All protein *k*_deg_ data, obtained by applying all RIA, t, data for mono-lysine peptides, grouped on a per protein basis, to a nonlinear model in R (one sheet per tissue).

**Supplemental Table S4.** *k*_deg_ data for all tissues in pivot table form, for easy cross-comparison.

**Supplemental Table S5.** Abundance data (including *k*_deg_), as tab delimited file (one sheet per tissue).

**Supplemental Table S6.** Flux data (including *k*_deg_), as tab delimited file (one sheet per tissue).
