# Supplementary figures and images for: Proteome Dynamics: Tissue Variation in the Kinetics of Proteostasis in Intact Animals
Source: Mol Cell Proteomics. 2016 Feb 1;15(4):1204–19. doi: 10.1074/mcp.M115.053488 (PMC4824850; doi:10.1074/mcp.M115.053488)

a)

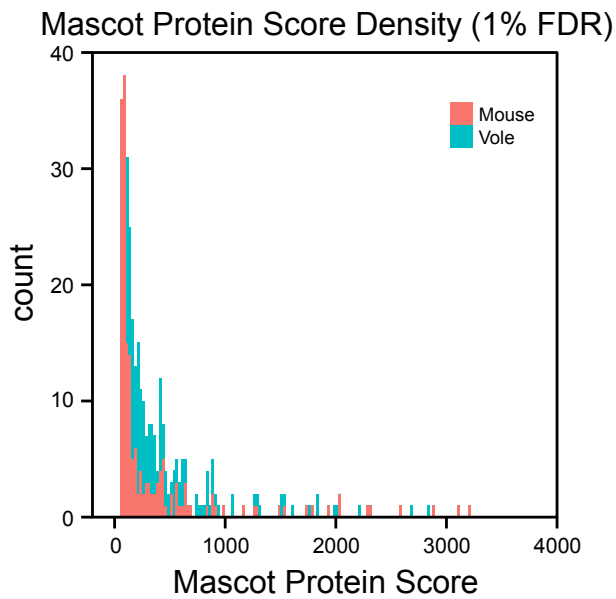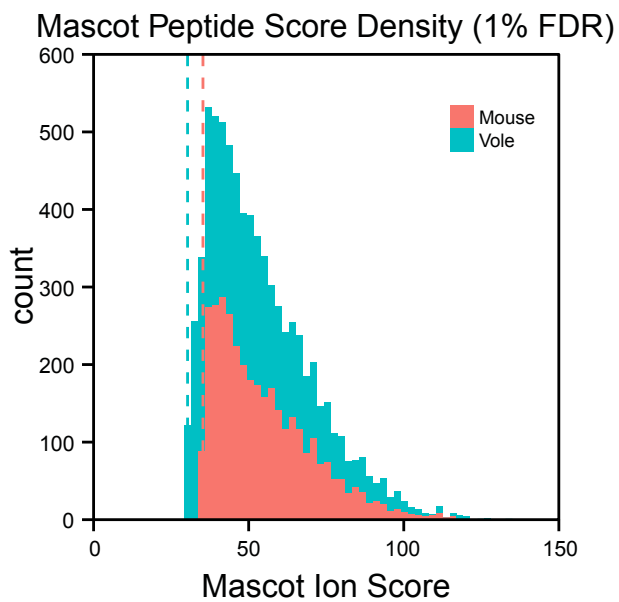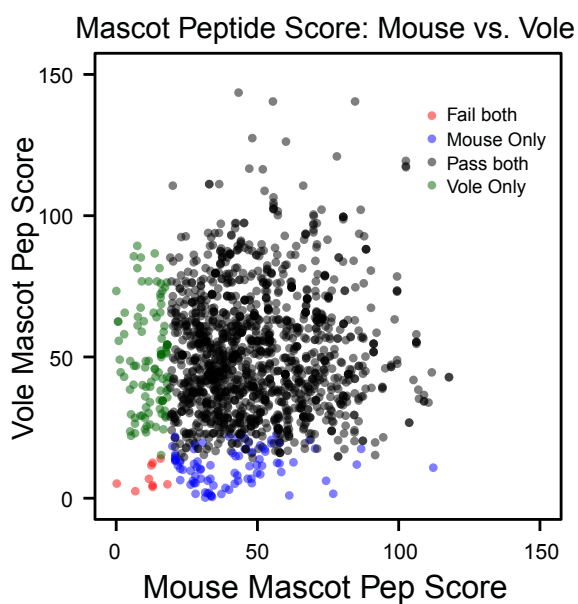

b)

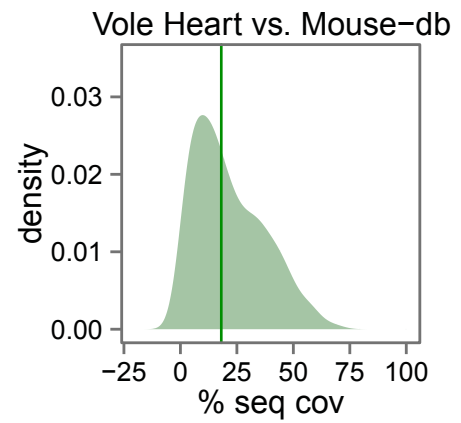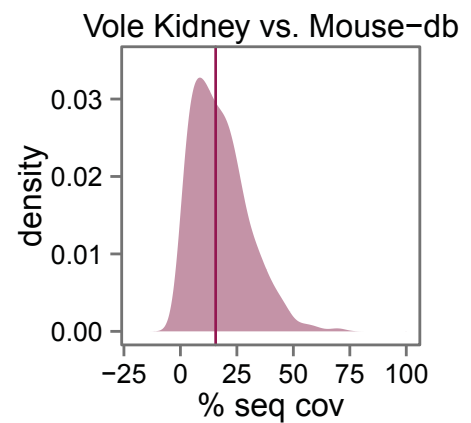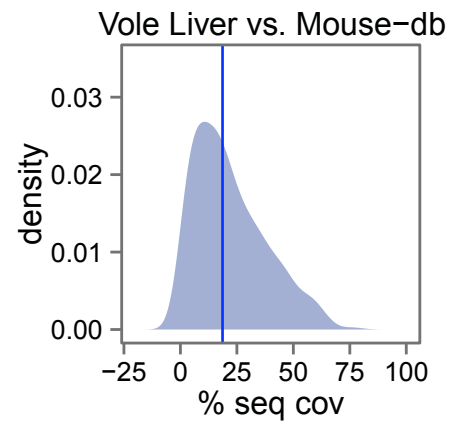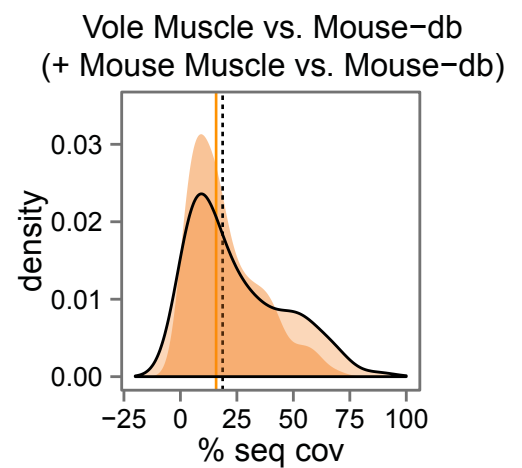

Supplement: Supplemental Data [file 10.1074_M115.053488_mcp.M115.053488-2.pdf]

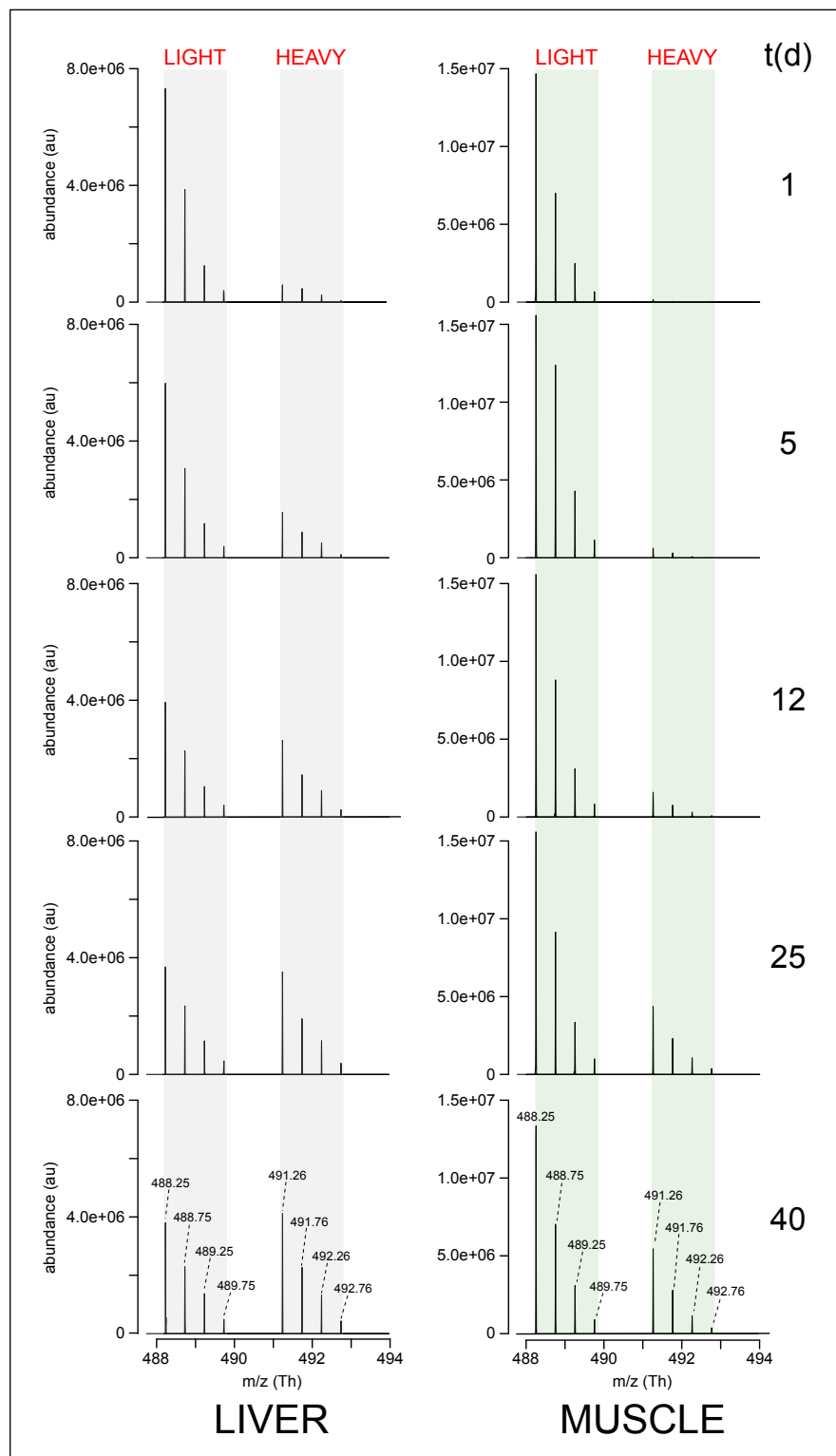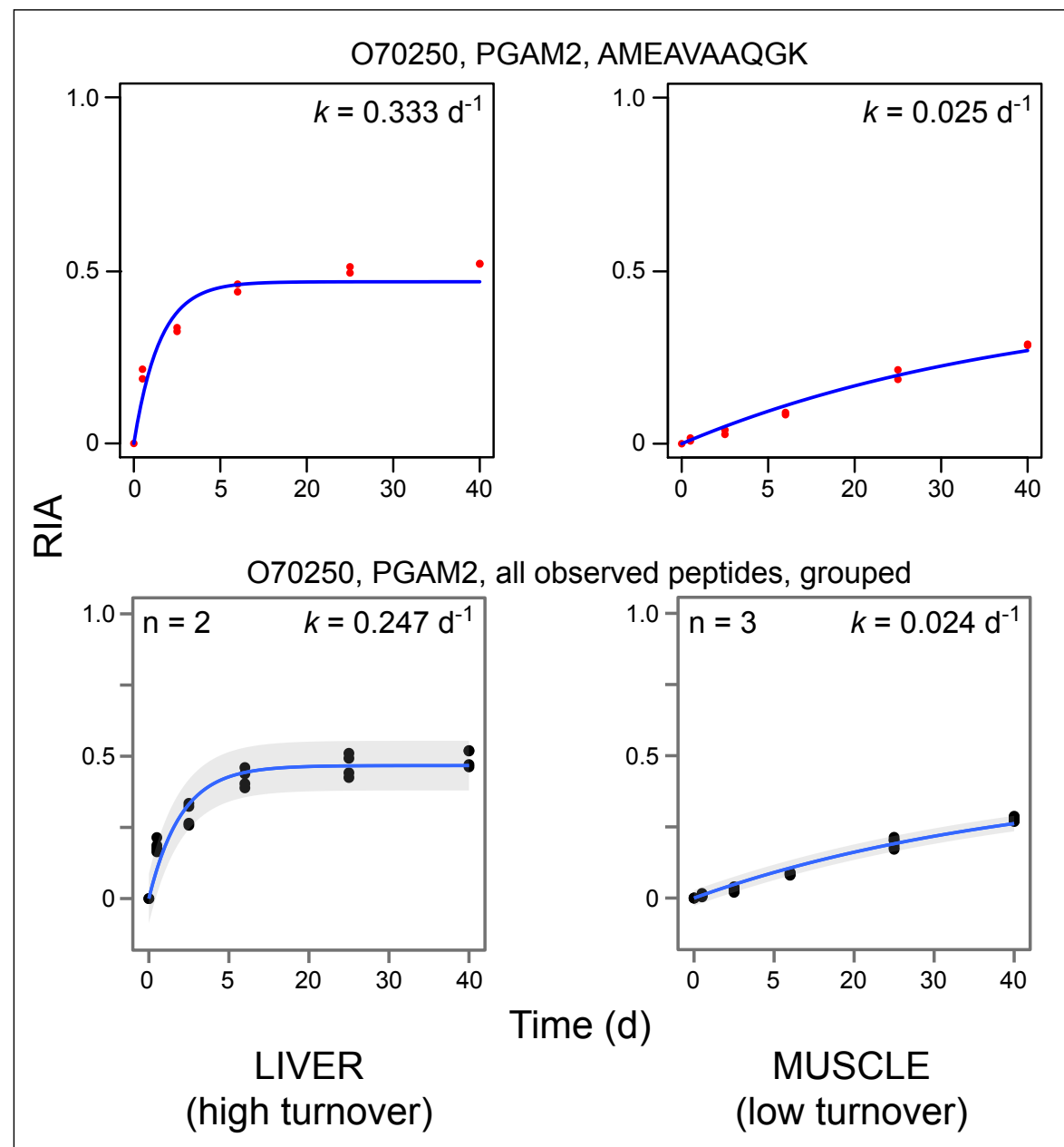

Supplement: Supplemental Data [file 10.1074_M115.053488_mcp.M115.053488-3.pdf]

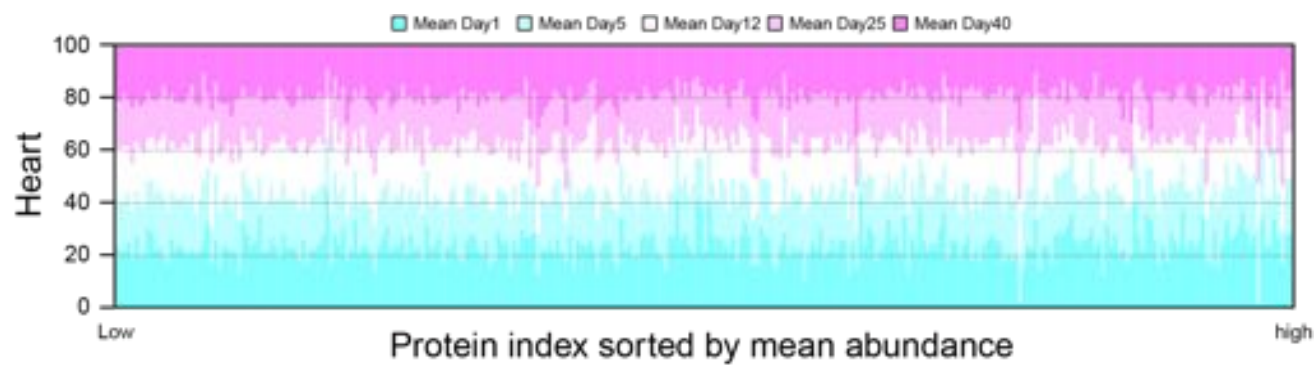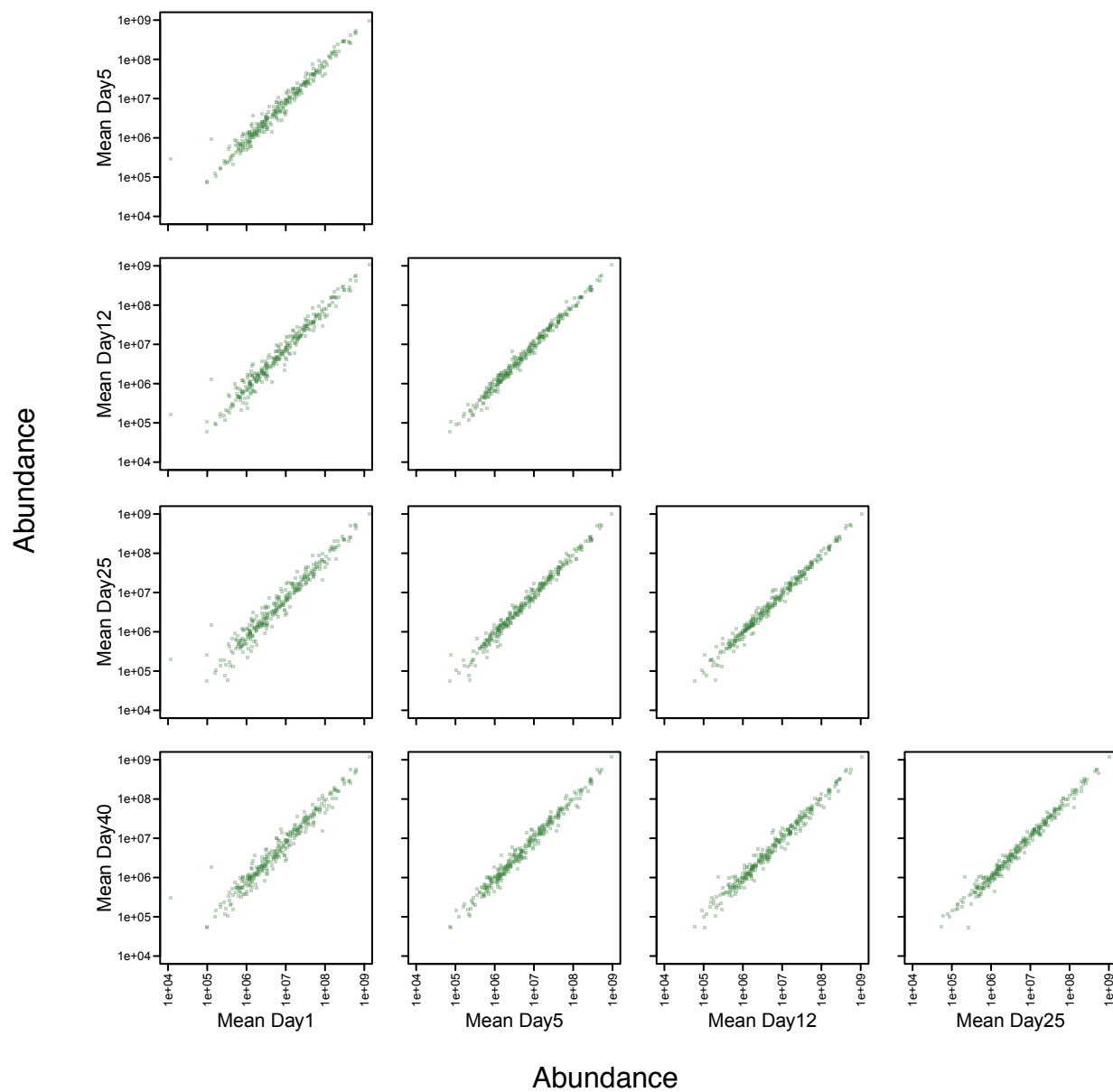

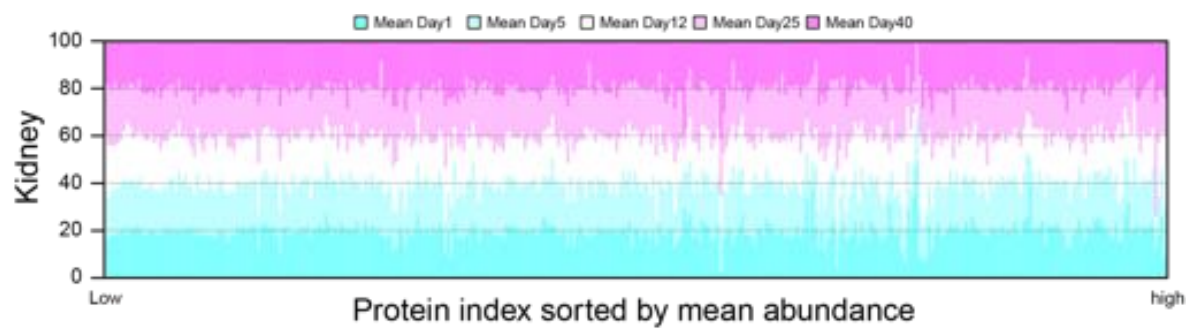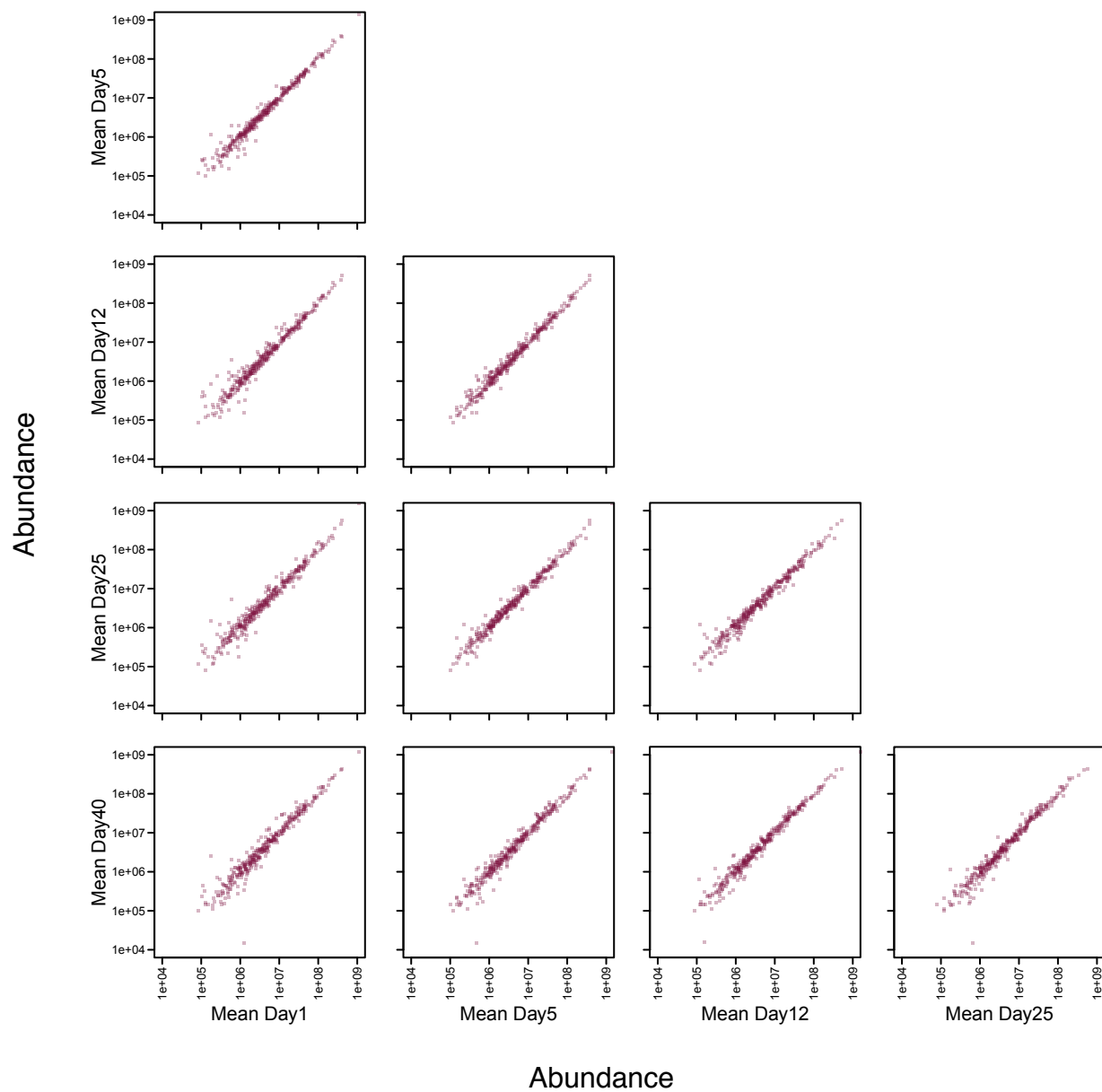

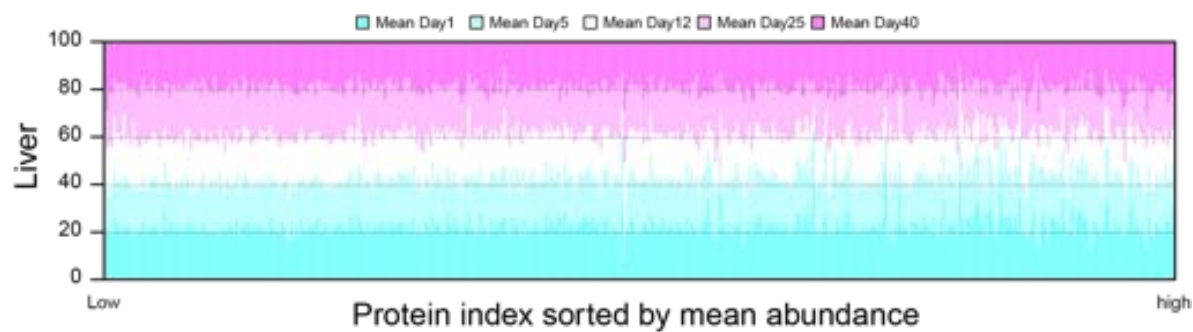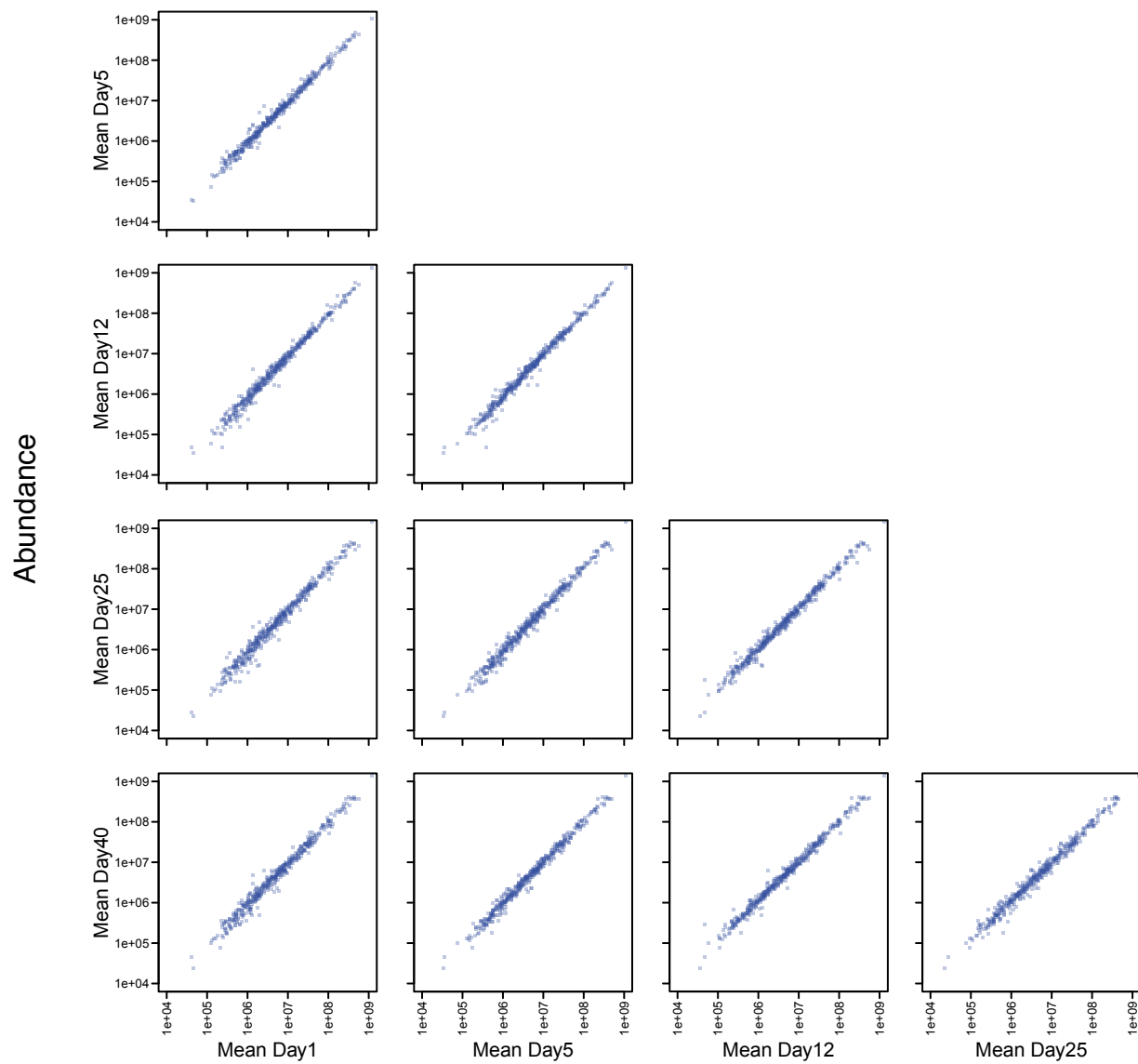

Abundance

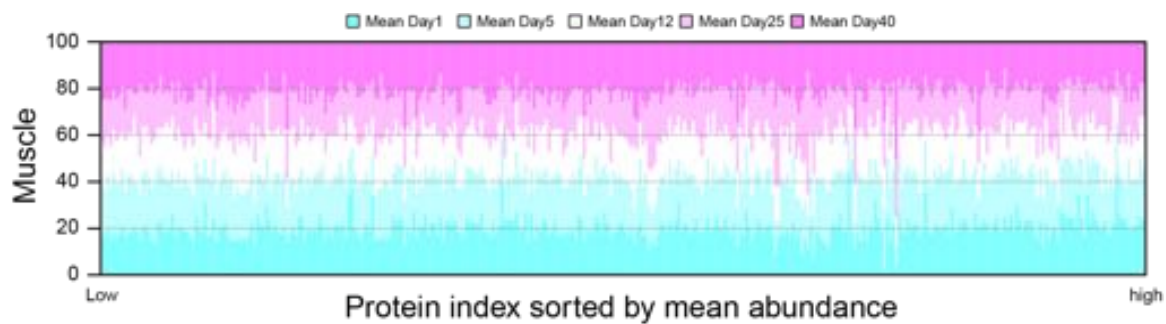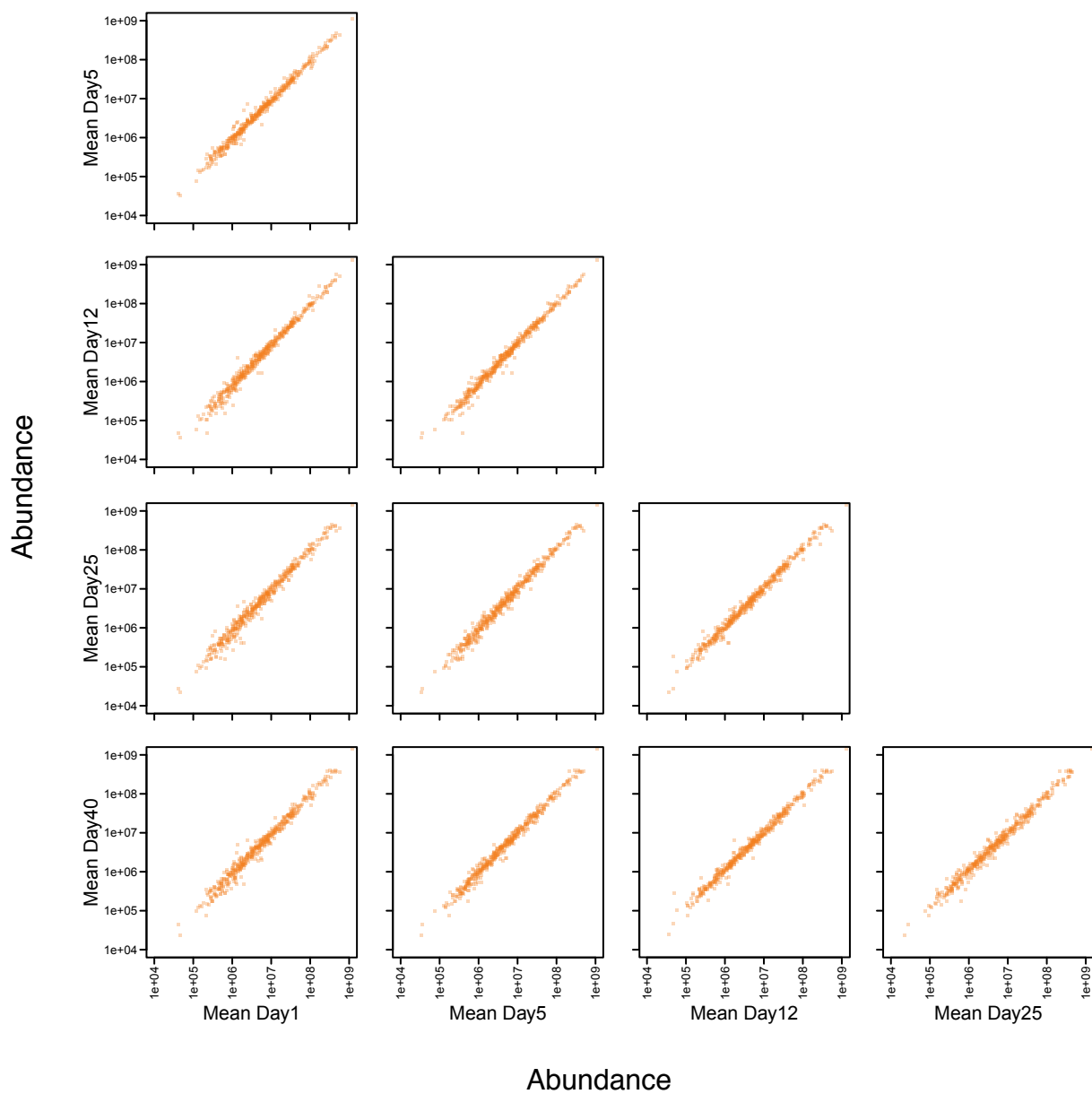

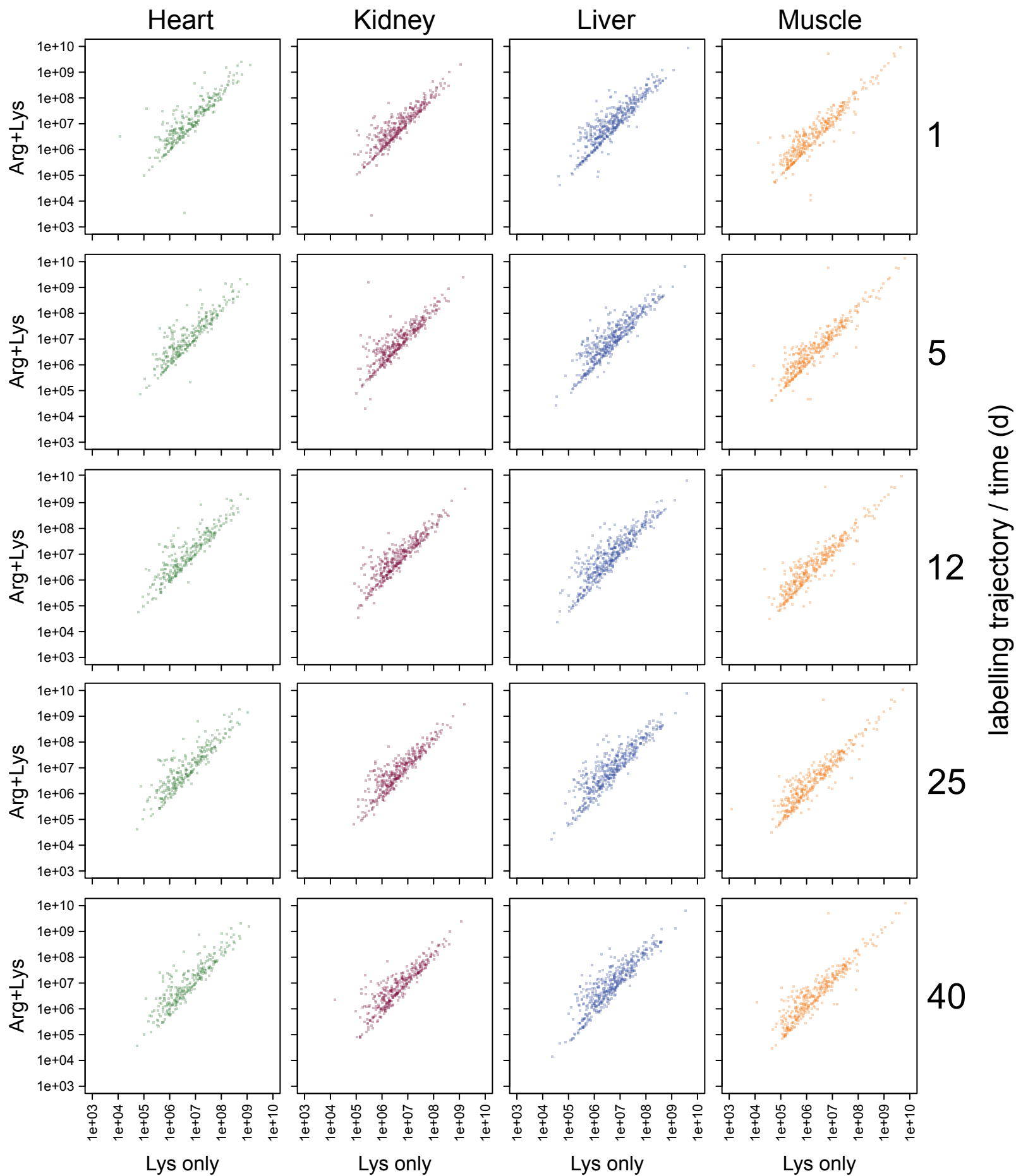

Supplement: Supplemental Data [file 10.1074_M115.053488_mcp.M115.053488-6.pdf]
