# Supplementary material for: Proteome Dynamics: Tissue Variation in the Kinetics of Proteostasis in Intact Animals
Source: Mol Cell Proteomics. 2016 Feb 1;15(4):1204–19. doi: 10.1074/mcp.M115.053488 (PMC4824850; doi:10.1074/mcp.M115.053488)
Supplement: Supplemental Data [file 10.1074_M115.053488_mcp.M115.053488-4.pdf]

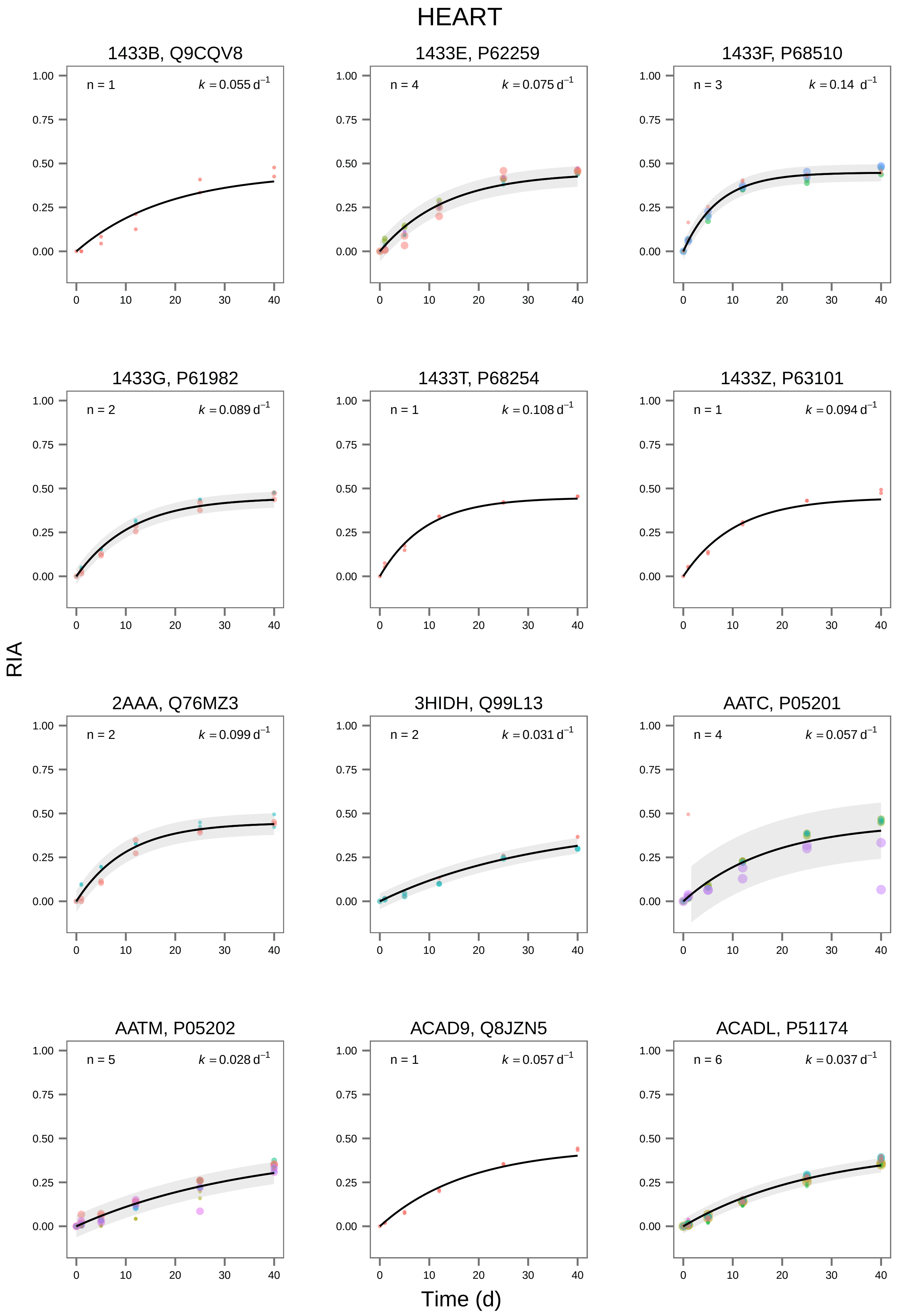
HEART
1433B, Q9CQV8
n = 1
k = 0.055 d⁻¹
1433E, P62259
n = 4
k = 0.075 d⁻¹
1433F, P68510
n = 3
k = 0.14 d⁻¹
1433G, P61982
n = 2
k = 0.089 d⁻¹
1433T, P68254
n = 1
k = 0.108 d⁻¹
1433Z, P63101
n = 1
k = 0.094 d⁻¹
2AAA, Q76MZ3
n = 2
k = 0.099 d⁻¹
3HIDH, Q99L13
n = 2
k = 0.031 d⁻¹
AATC, P05201
n = 4
k = 0.057 d⁻¹
AATM, P05202
n = 5
k = 0.028 d⁻¹
ACAD9, Q8JZN5
n = 1
k = 0.057 d⁻¹
ACADL, P51174
n = 6
k = 0.037 d⁻¹
1.00
0.75
0.50
0.25
0.00
0
10
20
30
40
RIA
Time (d)

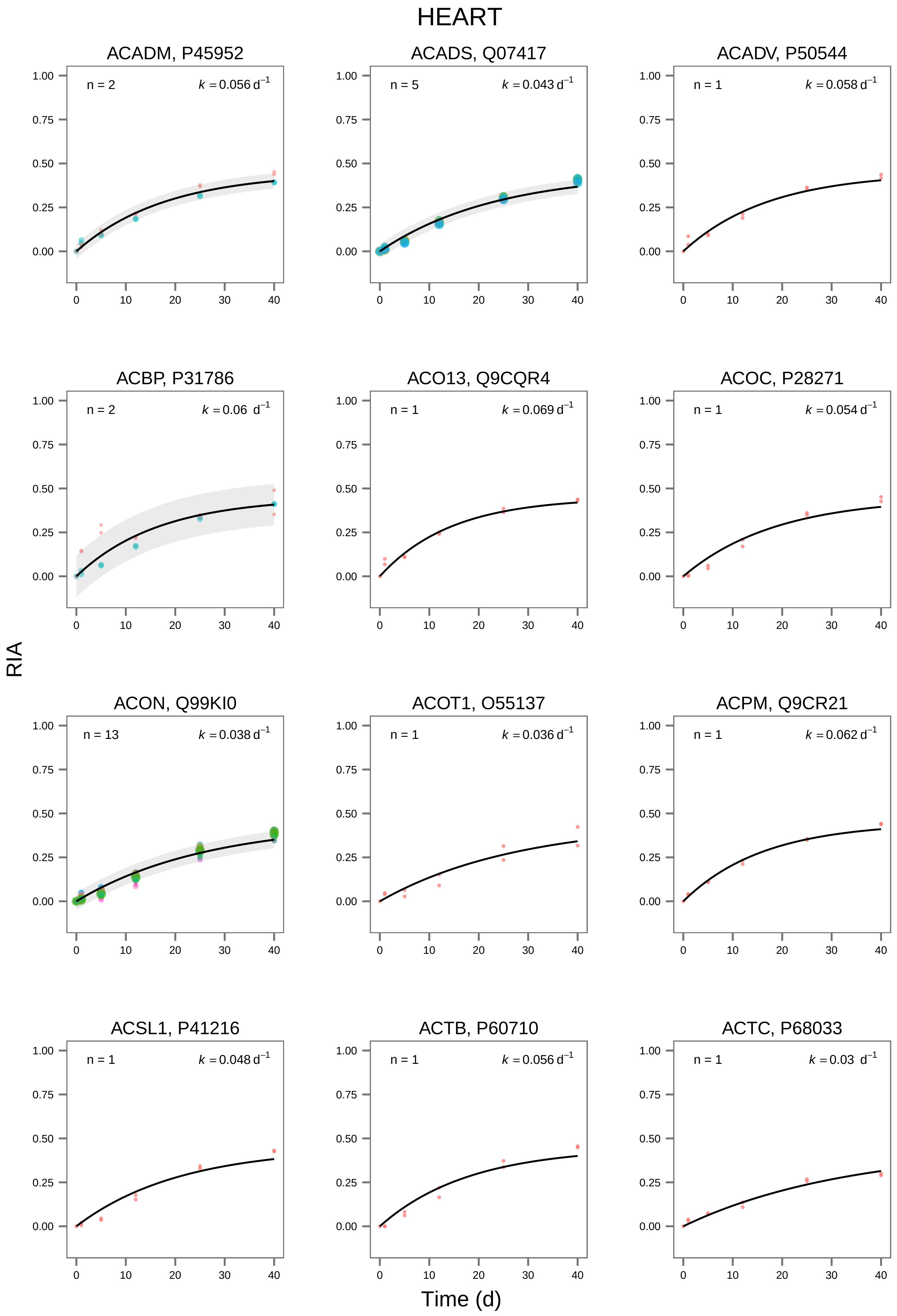
HEART
ACADM, P45952
n = 2
k = 0.056 d−1
ACADS, Q07417
n = 5
k = 0.043 d−1
ACADV, P50544
n = 1
k = 0.058 d−1
ACBP, P31786
n = 2
k = 0.06 d−1
ACO13, Q9CQR4
n = 1
k = 0.069 d−1
ACOC, P28271
n = 1
k = 0.054 d−1
ACON, Q99KI0
n = 13
k = 0.038 d−1
ACOT1, O55137
n = 1
k = 0.036 d−1
ACPM, Q9CR21
n = 1
k = 0.062 d−1
ACSL1, P41216
n = 1
k = 0.048 d−1
ACTB, P60710
n = 1
k = 0.056 d−1
ACTC, P68033
n = 1
k = 0.03 d−1
RIA
Time (d)
0.00
0.25
0.50
0.75
1.00
0
10
20
30
40

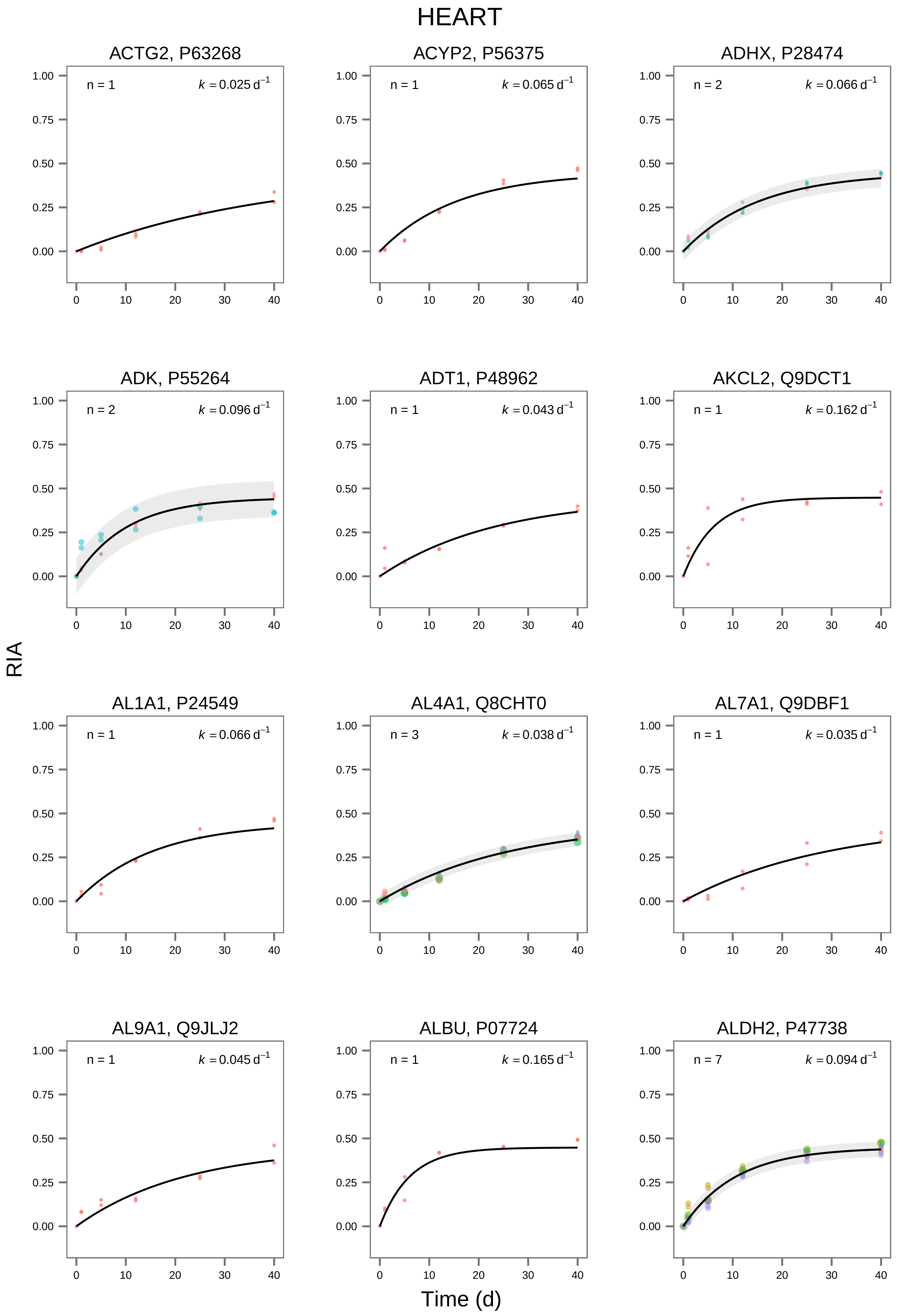
HEART
ACTG2, P63268
n = 1
k = 0.025 d−1
ACYP2, P56375
n = 1
k = 0.065 d−1
ADHX, P28474
n = 2
k = 0.066 d−1
ADK, P55264
n = 2
k = 0.096 d−1
ADT1, P48962
n = 1
k = 0.043 d−1
AKCL2, Q9DCT1
n = 1
k = 0.162 d−1
AL1A1, P24549
n = 1
k = 0.066 d−1
AL4A1, Q8CHT0
n = 3
k = 0.038 d−1
AL7A1, Q9DBF1
n = 1
k = 0.035 d−1
AL9A1, Q9JLJ2
n = 1
k = 0.045 d−1
ALBU, P07724
n = 1
k = 0.165 d−1
ALDH2, P47738
n = 7
k = 0.094 d−1
0.00
0.25
0.50
0.75
1.00
0
10
20
30
40
RIA
Time (d)

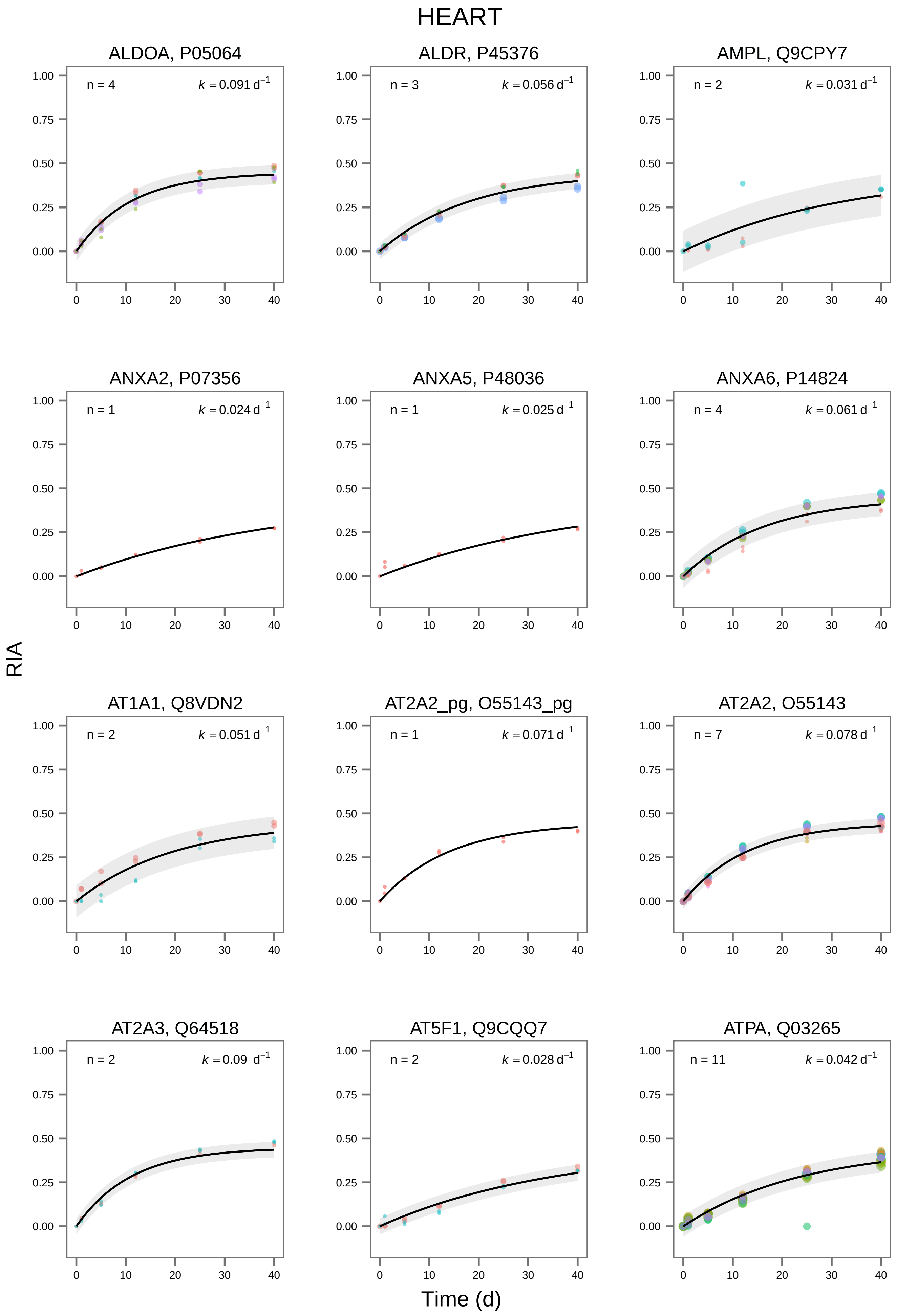
HEART
ALDOA, P05064
n = 4
k = 0.091 d^-1
ALDR, P45376
n = 3
k = 0.056 d^-1
AMPL, Q9CPY7
n = 2
k = 0.031 d^-1
ANXA2, P07356
n = 1
k = 0.024 d^-1
ANXA5, P48036
n = 1
k = 0.025 d^-1
ANXA6, P14824
n = 4
k = 0.061 d^-1
AT1A1, Q8VDN2
n = 2
k = 0.051 d^-1
AT2A2_pg, O55143_pg
n = 1
k = 0.071 d^-1
AT2A2, O55143
n = 7
k = 0.078 d^-1
AT2A3, Q64518
n = 2
k = 0.09 d^-1
AT5F1, Q9CQQ7
n = 2
k = 0.028 d^-1
ATPA, Q03265
n = 11
k = 0.042 d^-1
RIA
Time (d)
0.00
0.25
0.50
0.75
1.00
0
10
20
30
40

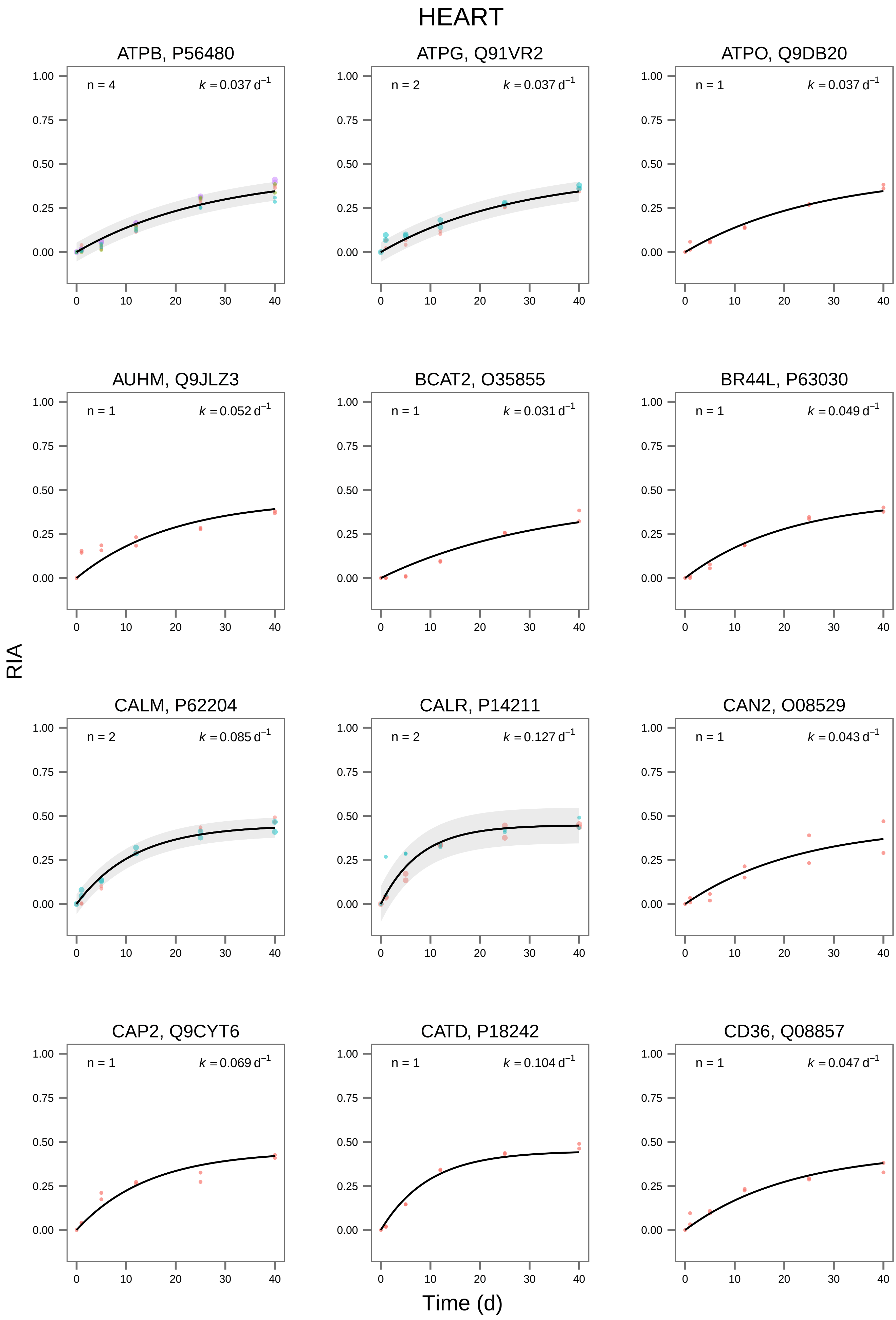
HEART
ATPB, P56480
n = 4
k = 0.037 d−1
ATPG, Q91VR2
n = 2
k = 0.037 d−1
ATPO, Q9DB20
n = 1
k = 0.037 d−1
AUHM, Q9JLZ3
n = 1
k = 0.052 d−1
BCAT2, O35855
n = 1
k = 0.031 d−1
BR44L, P63030
n = 1
k = 0.049 d−1
CALM, P62204
n = 2
k = 0.085 d−1
CALR, P14211
n = 2
k = 0.127 d−1
CAN2, O08529
n = 1
k = 0.043 d−1
CAP2, Q9CYT6
n = 1
k = 0.069 d−1
CATD, P18242
n = 1
k = 0.104 d−1
CD36, Q08857
n = 1
k = 0.047 d−1
RIA
Time (d)
0.00
0.25
0.50
0.75
1.00
0
10
20
30
40

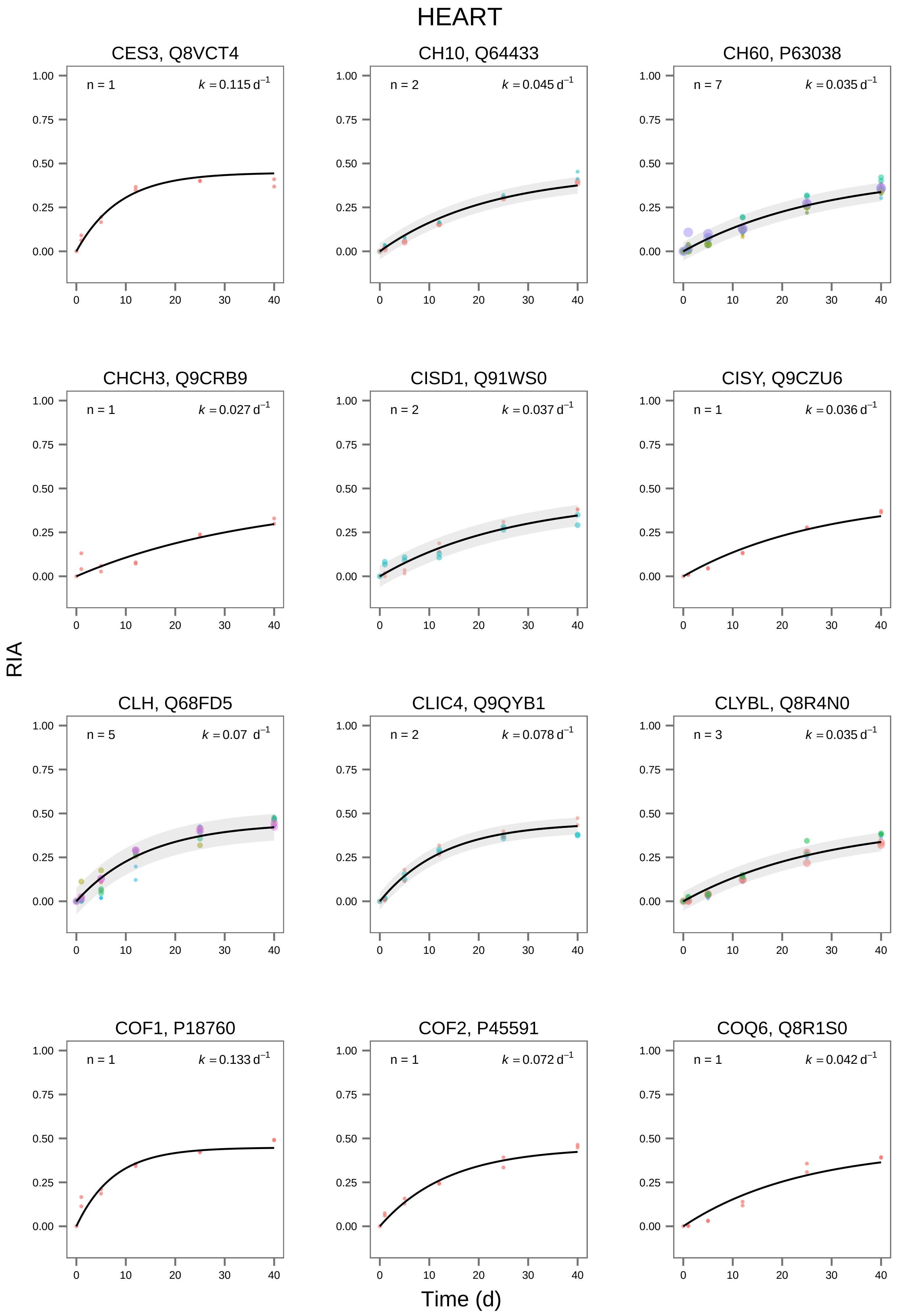
HEART
CES3, Q8VCT4
n = 1
k = 0.115 d⁻¹
CH10, Q64433
n = 2
k = 0.045 d⁻¹
CH60, P63038
n = 7
k = 0.035 d⁻¹
CHCH3, Q9CRB9
n = 1
k = 0.027 d⁻¹
CISD1, Q91WS0
n = 2
k = 0.037 d⁻¹
CISY, Q9CZU6
n = 1
k = 0.036 d⁻¹
CLH, Q68FD5
n = 5
k = 0.07 d⁻¹
CLIC4, Q9QYB1
n = 2
k = 0.078 d⁻¹
CLYBL, Q8R4N0
n = 3
k = 0.035 d⁻¹
COF1, P18760
n = 1
k = 0.133 d⁻¹
COF2, P45591
n = 1
k = 0.072 d⁻¹
COQ6, Q8R1S0
n = 1
k = 0.042 d⁻¹
RIA
Time (d)
0.00
0.25
0.50
0.75
1.00
0
10
20
30
40

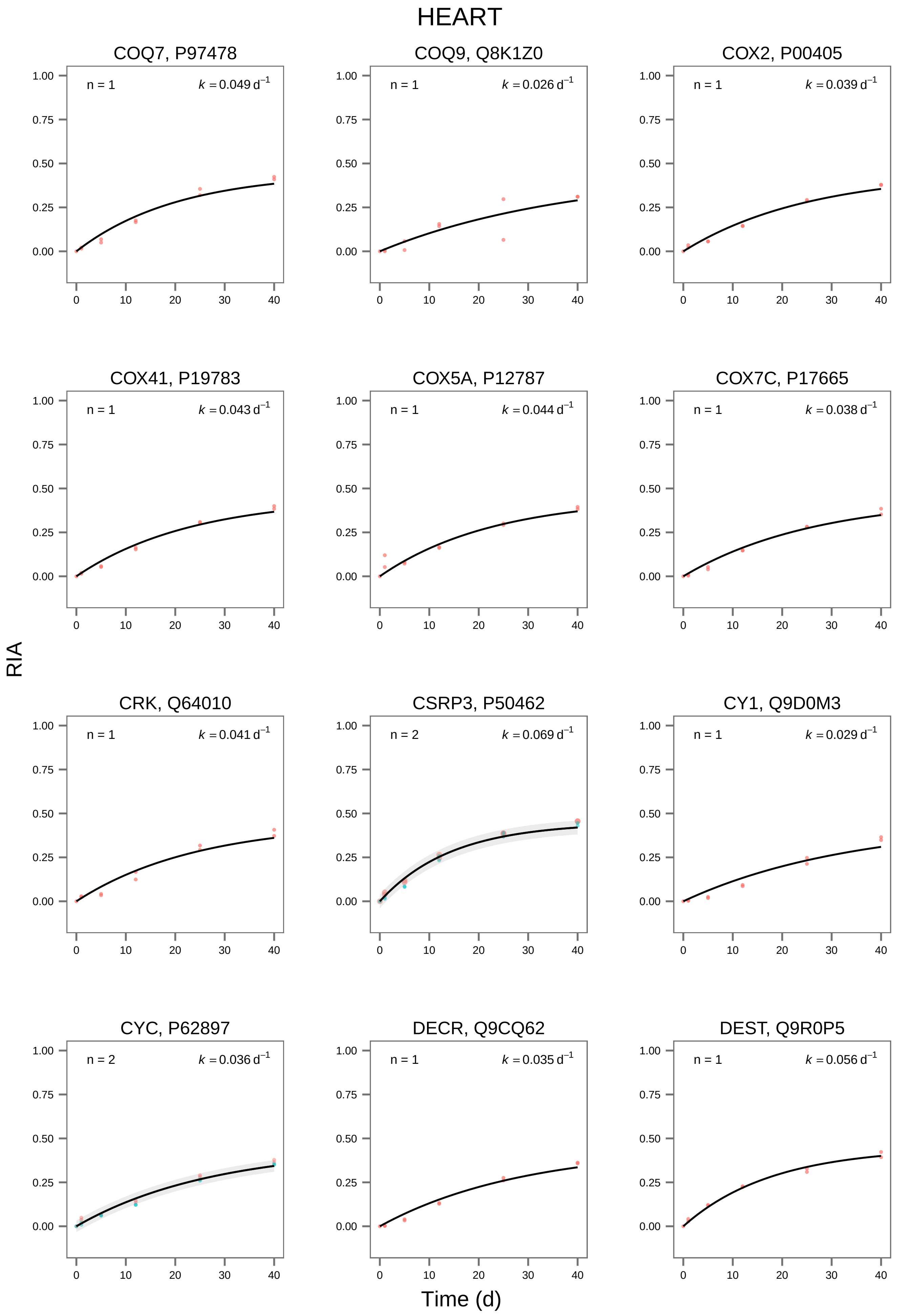
HEART
COQ7, P97478
n = 1
k = 0.049 d⁻¹
COQ9, Q8K1Z0
n = 1
k = 0.026 d⁻¹
COX2, P00405
n = 1
k = 0.039 d⁻¹
COX41, P19783
n = 1
k = 0.043 d⁻¹
COX5A, P12787
n = 1
k = 0.044 d⁻¹
COX7C, P17665
n = 1
k = 0.038 d⁻¹
CRK, Q64010
n = 1
k = 0.041 d⁻¹
CSRP3, P50462
n = 2
k = 0.069 d⁻¹
CY1, Q9D0M3
n = 1
k = 0.029 d⁻¹
CYC, P62897
n = 2
k = 0.036 d⁻¹
DECR, Q9CQ62
n = 1
k = 0.035 d⁻¹
DEST, Q9R0P5
n = 1
k = 0.056 d⁻¹
RIA
Time (d)
0.00 0.25 0.50 0.75 1.00
0 10 20 30 40

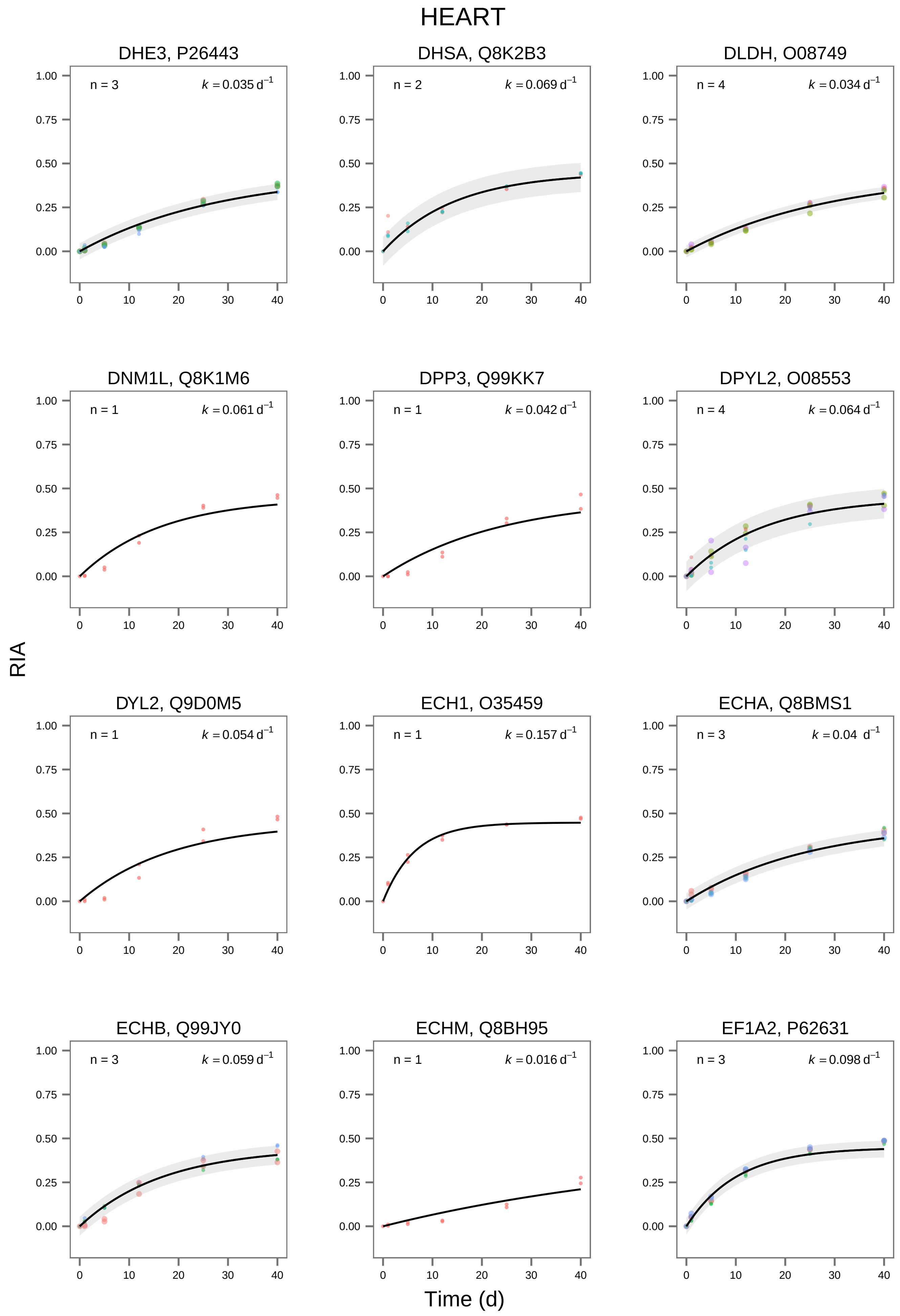
HEART
DHE3, P26443
n = 3
k = 0.035 d⁻¹
DHSA, Q8K2B3
n = 2
k = 0.069 d⁻¹
DLDH, O08749
n = 4
k = 0.034 d⁻¹
DNM1L, Q8K1M6
n = 1
k = 0.061 d⁻¹
DPP3, Q99KK7
n = 1
k = 0.042 d⁻¹
DPYL2, O08553
n = 4
k = 0.064 d⁻¹
DYL2, Q9D0M5
n = 1
k = 0.054 d⁻¹
ECH1, O35459
n = 1
k = 0.157 d⁻¹
ECHA, Q8BMS1
n = 3
k = 0.04 d⁻¹
ECHB, Q99JY0
n = 3
k = 0.059 d⁻¹
ECHM, Q8BH95
n = 1
k = 0.016 d⁻¹
EF1A2, P62631
n = 3
k = 0.098 d⁻¹
RIA
Time (d)
1.00
0.75
0.50
0.25
0.00
0
10
20
30
40

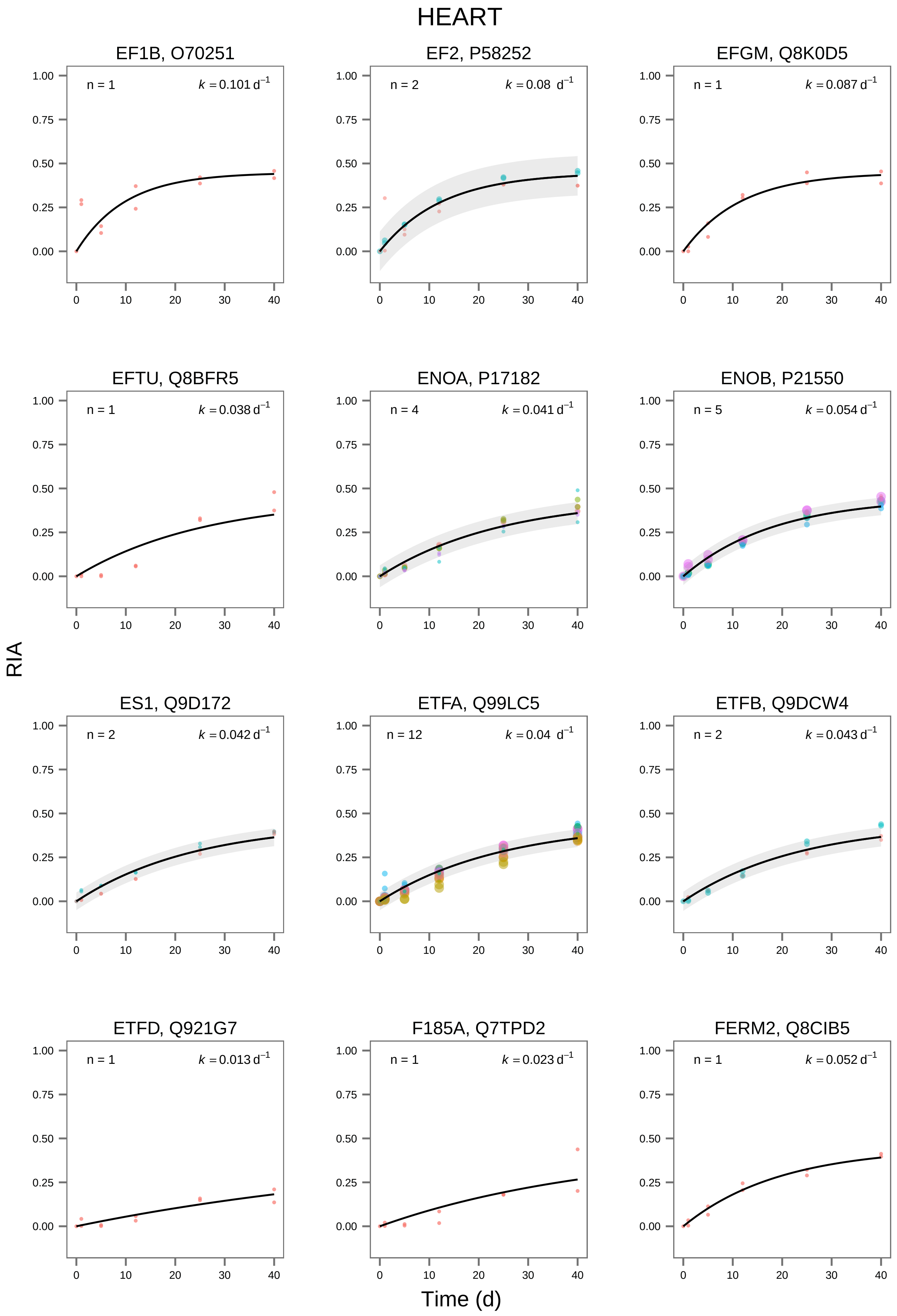
HEART
EF1B, O70251
n = 1
k = 0.101 d⁻¹
EF2, P58252
n = 2
k = 0.08 d⁻¹
EFGM, Q8K0D5
n = 1
k = 0.087 d⁻¹
EFTU, Q8BFR5
n = 1
k = 0.038 d⁻¹
ENOA, P17182
n = 4
k = 0.041 d⁻¹
ENOB, P21550
n = 5
k = 0.054 d⁻¹
ES1, Q9D172
n = 2
k = 0.042 d⁻¹
ETFA, Q99LC5
n = 12
k = 0.04 d⁻¹
ETFB, Q9DCW4
n = 2
k = 0.043 d⁻¹
ETFD, Q921G7
n = 1
k = 0.013 d⁻¹
F185A, Q7TPD2
n = 1
k = 0.023 d⁻¹
FERM2, Q8CIB5
n = 1
k = 0.052 d⁻¹
RIA
Time (d)
0.00
0.25
0.50
0.75
1.00
0
10
20
30
40

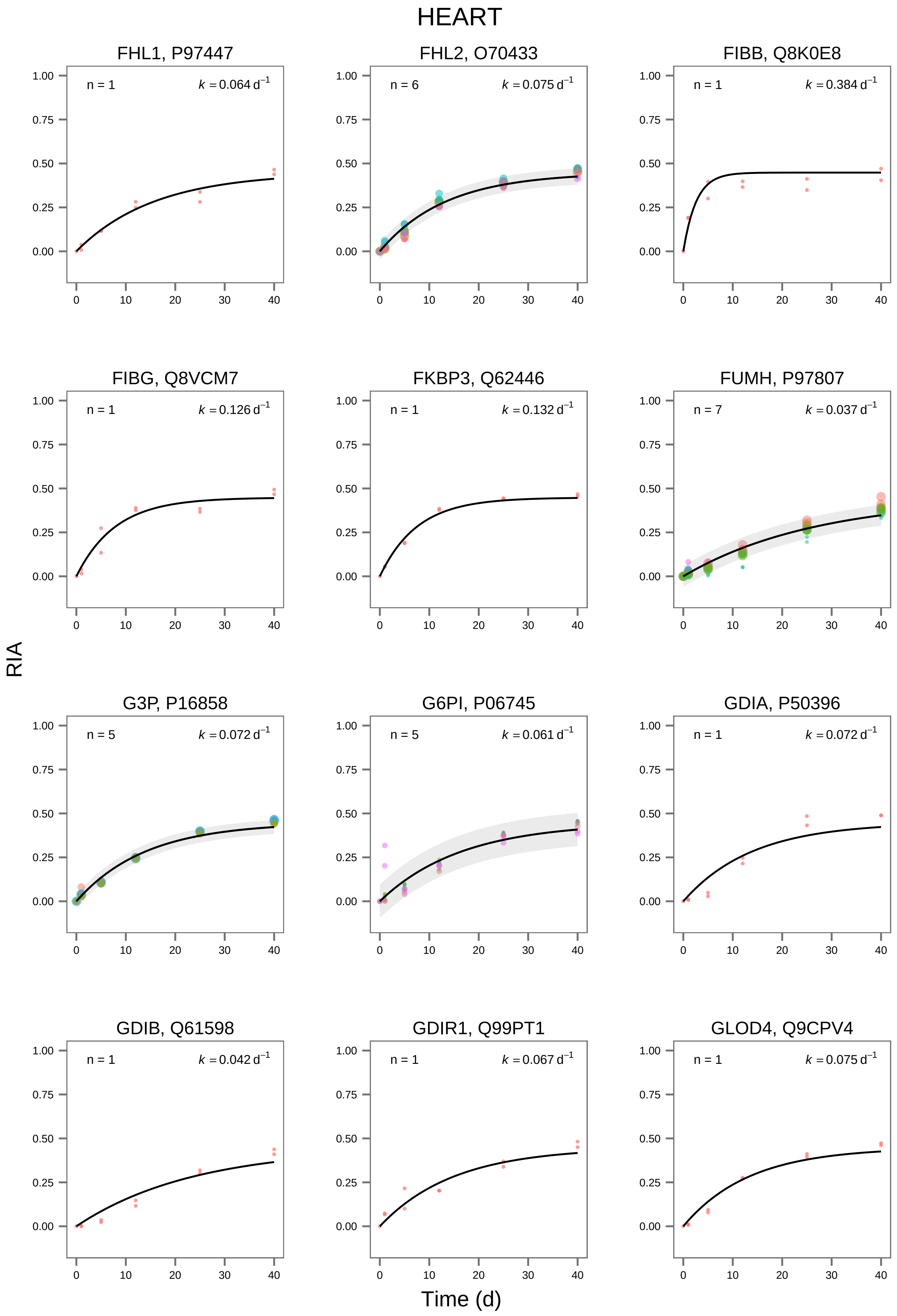
HEART
FHL1, P97447
n = 1
k = 0.064 d−1
FHL2, O70433
n = 6
k = 0.075 d−1
FIBB, Q8K0E8
n = 1
k = 0.384 d−1
FIBG, Q8VCM7
n = 1
k = 0.126 d−1
FKBP3, Q62446
n = 1
k = 0.132 d−1
FUMH, P97807
n = 7
k = 0.037 d−1
G3P, P16858
n = 5
k = 0.072 d−1
G6PI, P06745
n = 5
k = 0.061 d−1
GDIA, P50396
n = 1
k = 0.072 d−1
GDIB, Q61598
n = 1
k = 0.042 d−1
GDIR1, Q99PT1
n = 1
k = 0.067 d−1
GLOD4, Q9CPV4
n = 1
k = 0.075 d−1
0.00
0.25
0.50
0.75
1.00
0
10
20
30
40
RIA
Time (d)

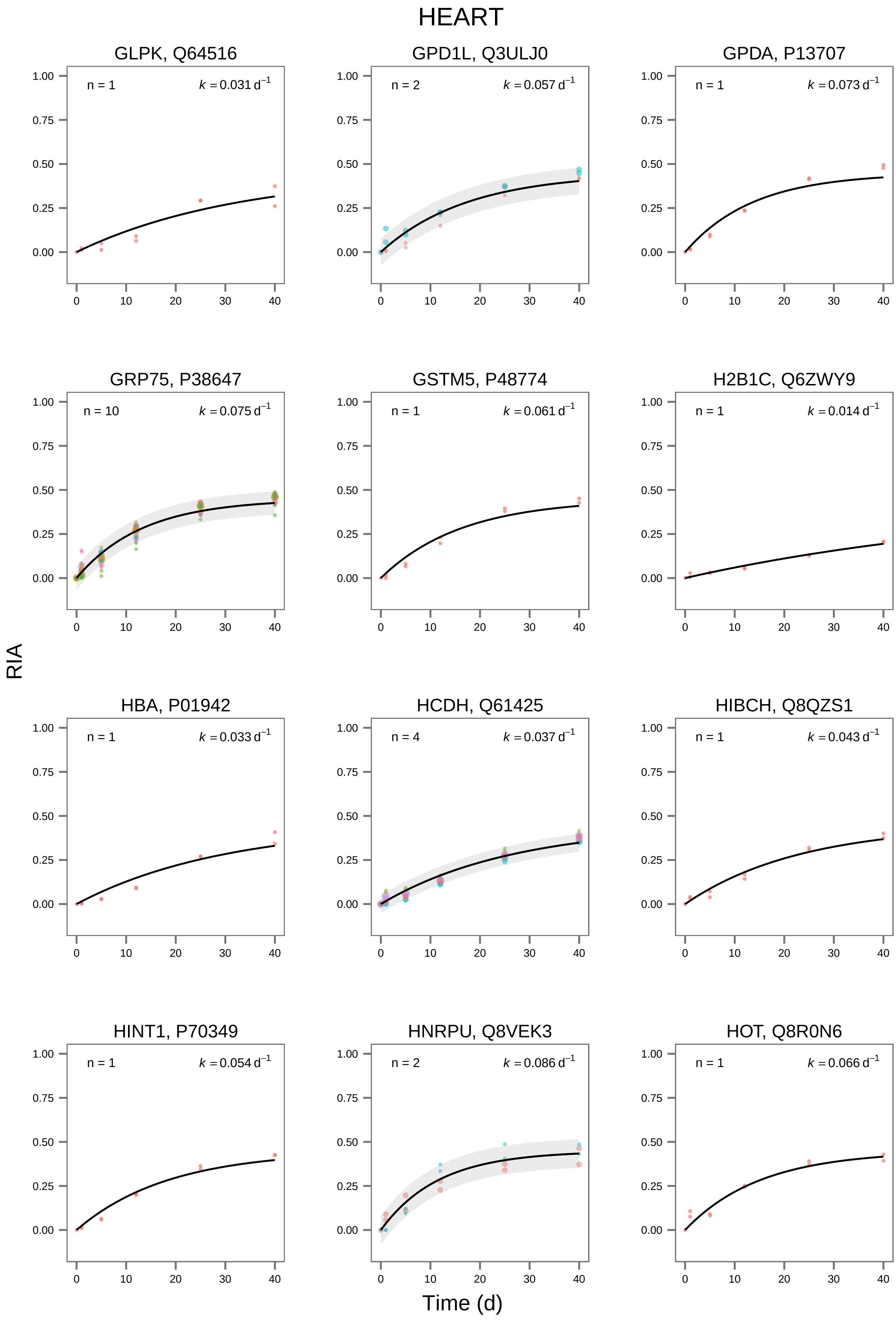
HEART
GLPK, Q64516
n = 1
k = 0.031 d−1
GPD1L, Q3ULJ0
n = 2
k = 0.057 d−1
GPDA, P13707
n = 1
k = 0.073 d−1
GRP75, P38647
n = 10
k = 0.075 d−1
GSTM5, P48774
n = 1
k = 0.061 d−1
H2B1C, Q6ZWY9
n = 1
k = 0.014 d−1
HBA, P01942
n = 1
k = 0.033 d−1
HCDH, Q61425
n = 4
k = 0.037 d−1
HIBCH, Q8QZS1
n = 1
k = 0.043 d−1
HINT1, P70349
n = 1
k = 0.054 d−1
HNRPU, Q8VEK3
n = 2
k = 0.086 d−1
HOT, Q8R0N6
n = 1
k = 0.066 d−1
RIA
Time (d)

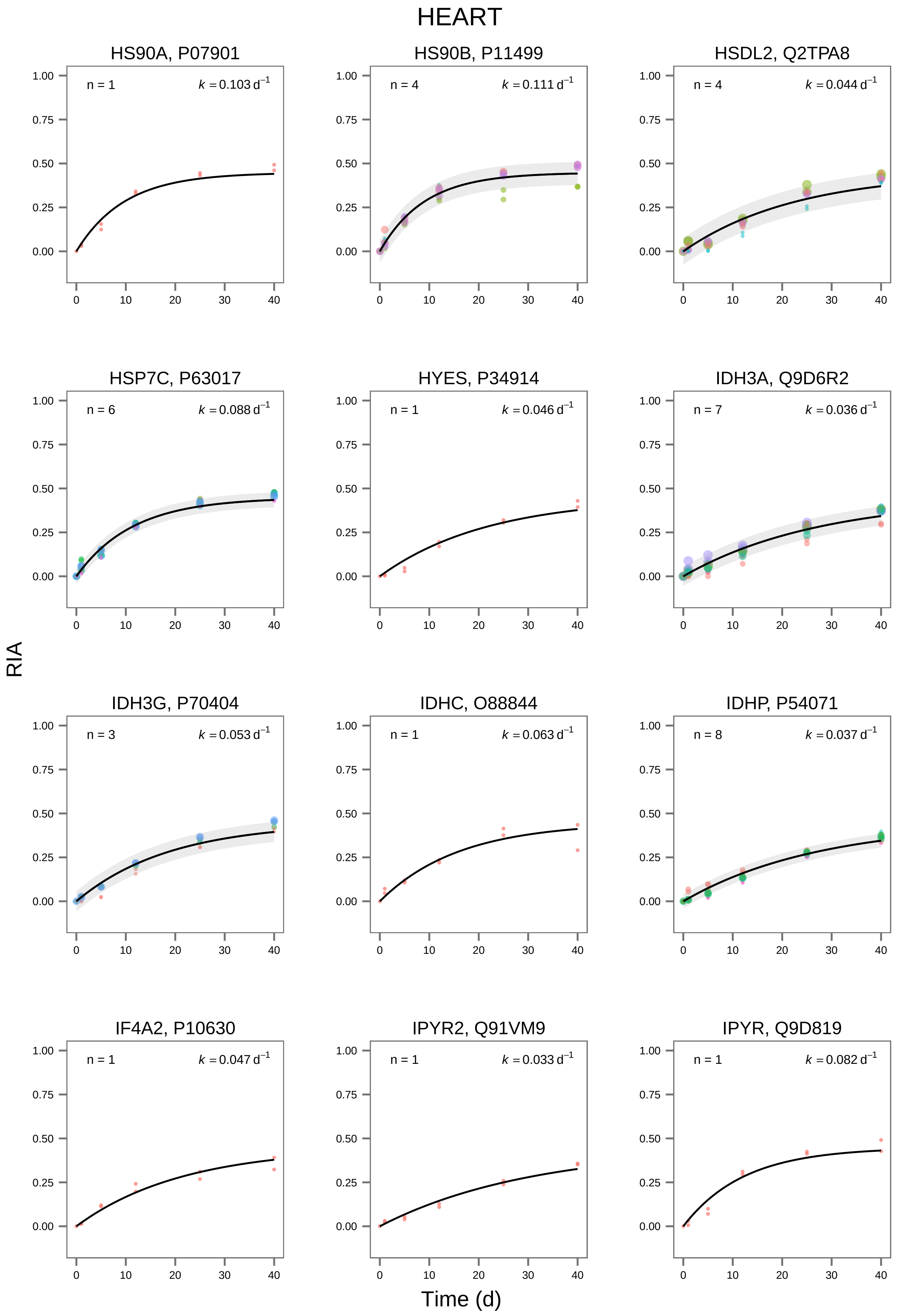
HEART
HS90A, P07901
n = 1
k = 0.103 d⁻¹
HS90B, P11499
n = 4
k = 0.111 d⁻¹
HSDL2, Q2TPA8
n = 4
k = 0.044 d⁻¹
HSP7C, P63017
n = 6
k = 0.088 d⁻¹
HYES, P34914
n = 1
k = 0.046 d⁻¹
IDH3A, Q9D6R2
n = 7
k = 0.036 d⁻¹
IDH3G, P70404
n = 3
k = 0.053 d⁻¹
IDHC, O88844
n = 1
k = 0.063 d⁻¹
IDHP, P54071
n = 8
k = 0.037 d⁻¹
IF4A2, P10630
n = 1
k = 0.047 d⁻¹
IPYR2, Q91VM9
n = 1
k = 0.033 d⁻¹
IPYR, Q9D819
n = 1
k = 0.082 d⁻¹
0.00
0.25
0.50
0.75
1.00
0
10
20
30
40
RIA
Time (d)

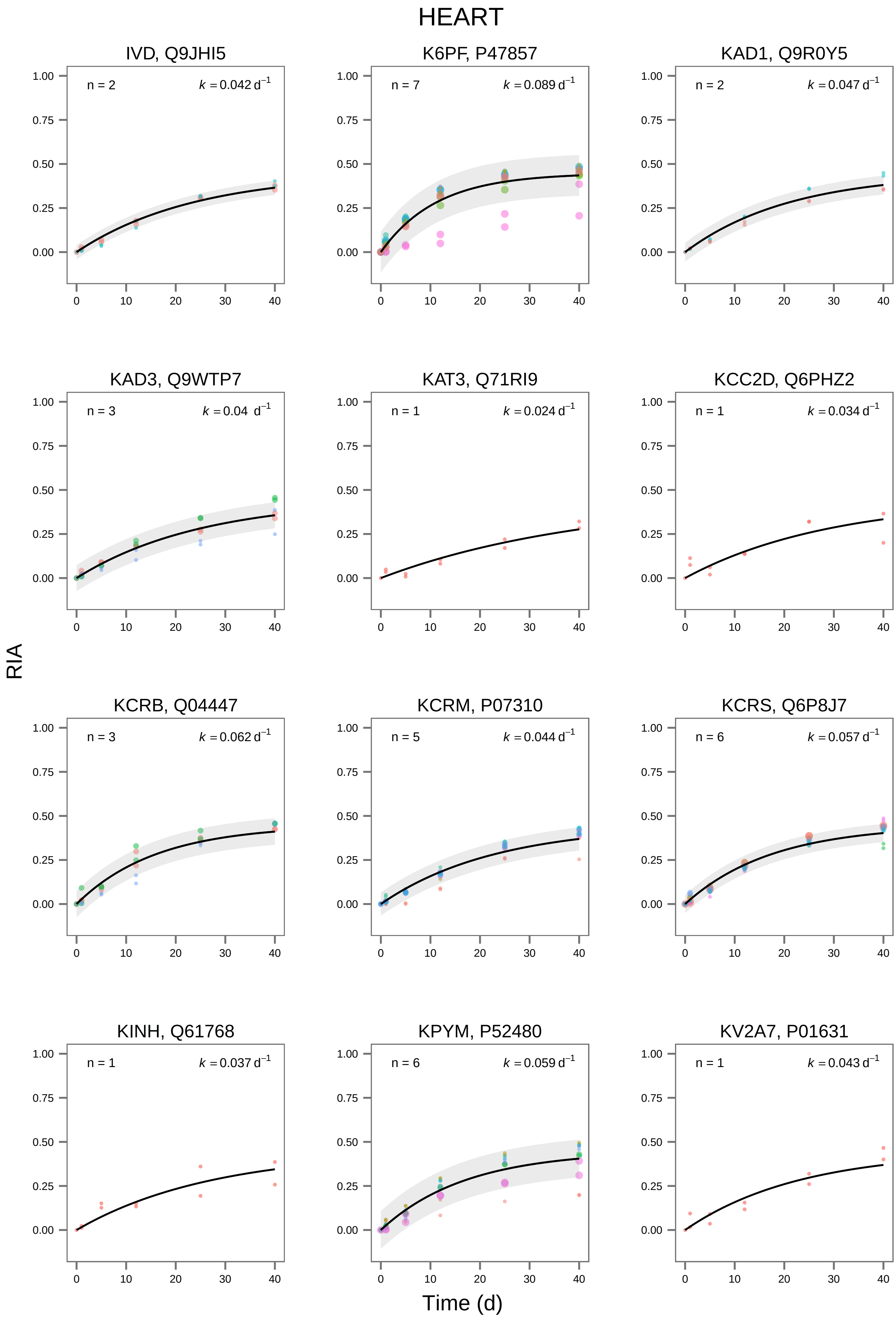
HEART
IVD, Q9JHI5
n = 2
k = 0.042 d−1
K6PF, P47857
n = 7
k = 0.089 d−1
KAD1, Q9R0Y5
n = 2
k = 0.047 d−1
KAD3, Q9WTP7
n = 3
k = 0.04 d−1
KAT3, Q71RI9
n = 1
k = 0.024 d−1
KCC2D, Q6PHZ2
n = 1
k = 0.034 d−1
KCRB, Q04447
n = 3
k = 0.062 d−1
KCRM, P07310
n = 5
k = 0.044 d−1
KCRS, Q6P8J7
n = 6
k = 0.057 d−1
KINH, Q61768
n = 1
k = 0.037 d−1
KPYM, P52480
n = 6
k = 0.059 d−1
KV2A7, P01631
n = 1
k = 0.043 d−1
RIA
Time (d)

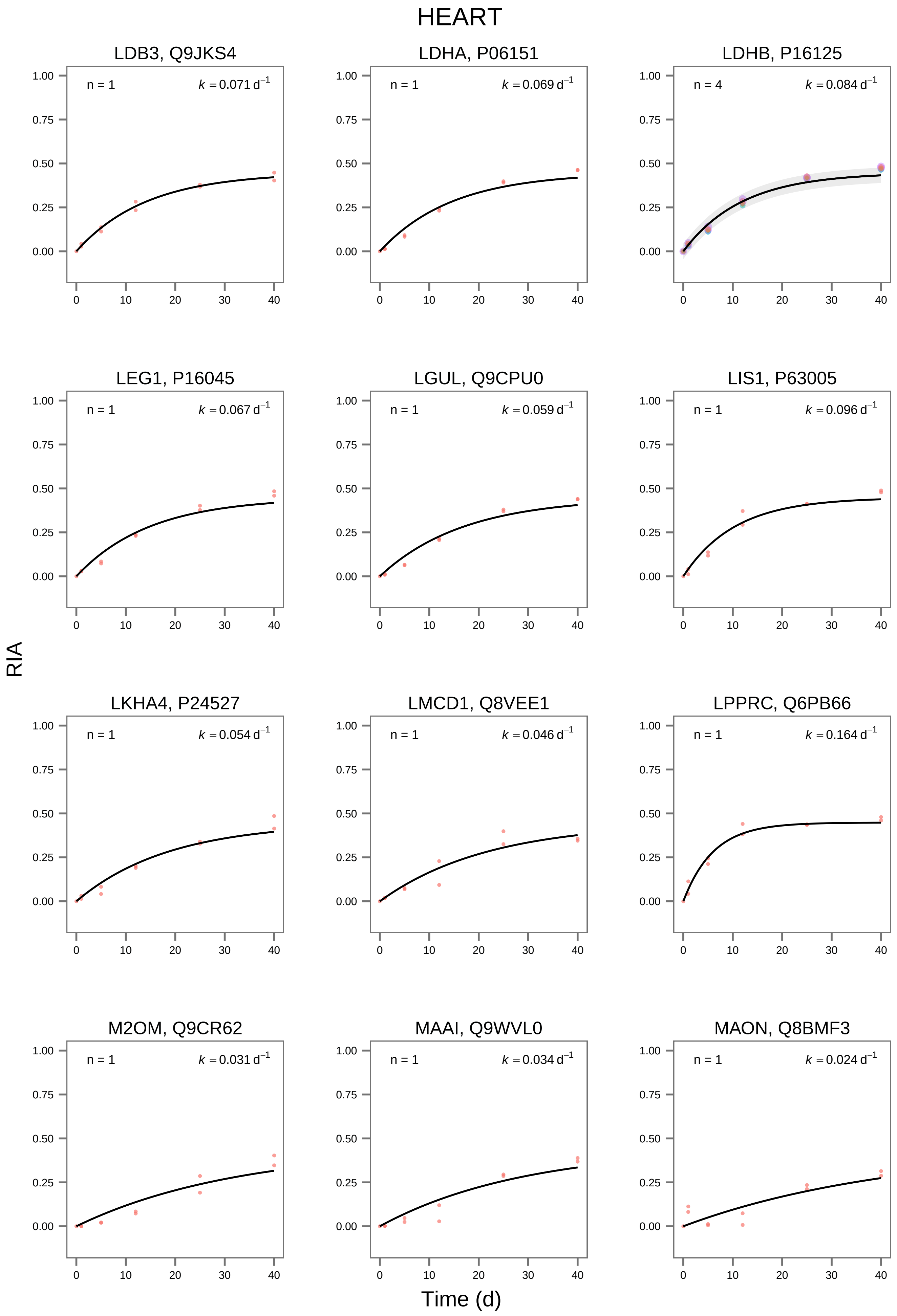
HEART
LDB3, Q9JKS4
n = 1
k = 0.071 d⁻¹
LDHA, P06151
n = 1
k = 0.069 d⁻¹
LDHB, P16125
n = 4
k = 0.084 d⁻¹
LEG1, P16045
n = 1
k = 0.067 d⁻¹
LGUL, Q9CPU0
n = 1
k = 0.059 d⁻¹
LIS1, P63005
n = 1
k = 0.096 d⁻¹
LKHA4, P24527
n = 1
k = 0.054 d⁻¹
LMCD1, Q8VEE1
n = 1
k = 0.046 d⁻¹
LPPRC, Q6PB66
n = 1
k = 0.164 d⁻¹
M2OM, Q9CR62
n = 1
k = 0.031 d⁻¹
MAAI, Q9WVL0
n = 1
k = 0.034 d⁻¹
MAON, Q8BMF3
n = 1
k = 0.024 d⁻¹
RIA
Time (d)
1.00
0.75
0.50
0.25
0.00
0
10
20
30
40

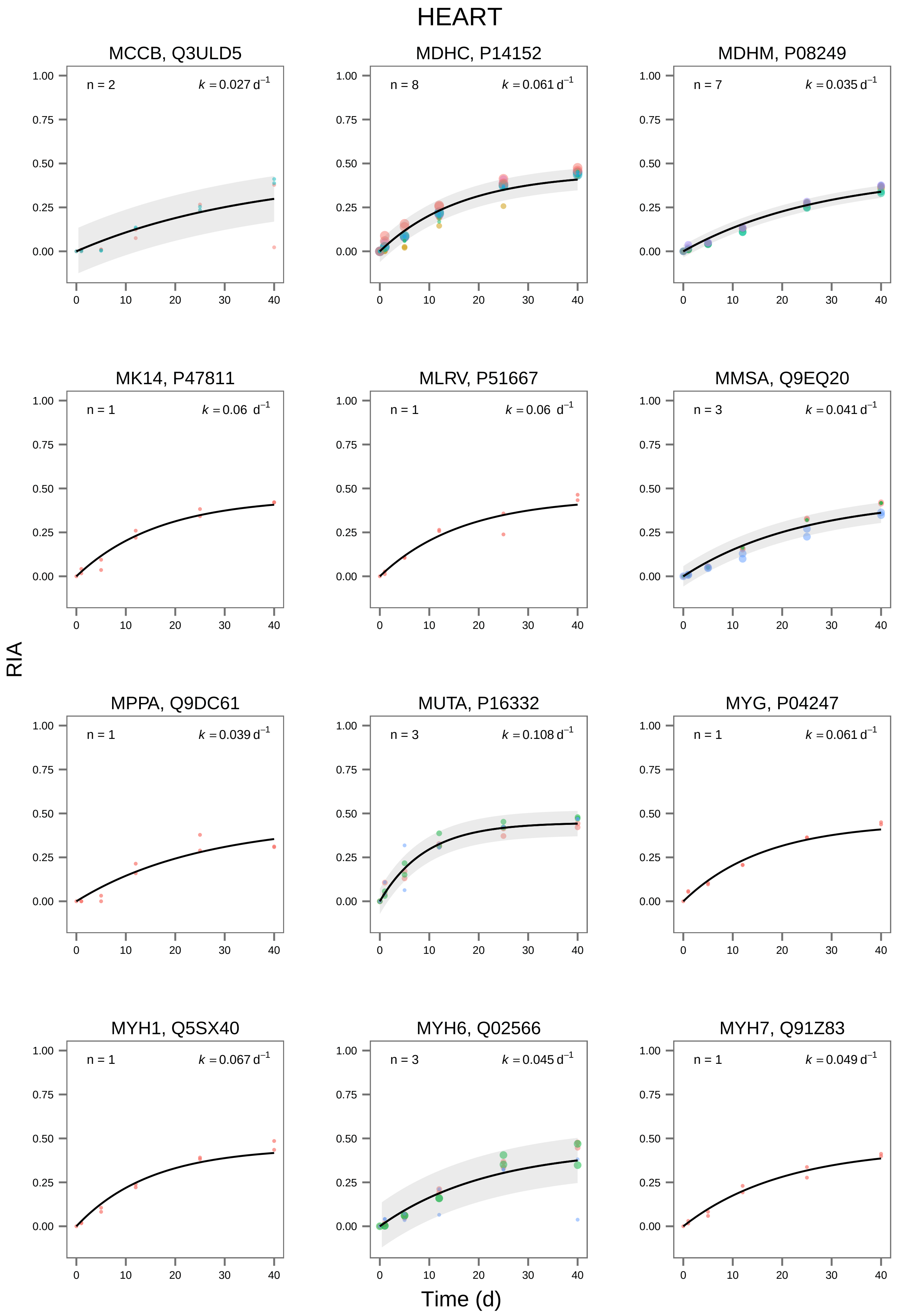
HEART
MCCB, Q3ULD5
n = 2
k = 0.027 d⁻¹
MDHC, P14152
n = 8
k = 0.061 d⁻¹
MDHM, P08249
n = 7
k = 0.035 d⁻¹
MK14, P47811
n = 1
k = 0.06 d⁻¹
MLRV, P51667
n = 1
k = 0.06 d⁻¹
MMSA, Q9EQ20
n = 3
k = 0.041 d⁻¹
MPPA, Q9DC61
n = 1
k = 0.039 d⁻¹
MUTA, P16332
n = 3
k = 0.108 d⁻¹
MYG, P04247
n = 1
k = 0.061 d⁻¹
MYH1, Q5SX40
n = 1
k = 0.067 d⁻¹
MYH6, Q02566
n = 3
k = 0.045 d⁻¹
MYH7, Q91Z83
n = 1
k = 0.049 d⁻¹
RIA
Time (d)

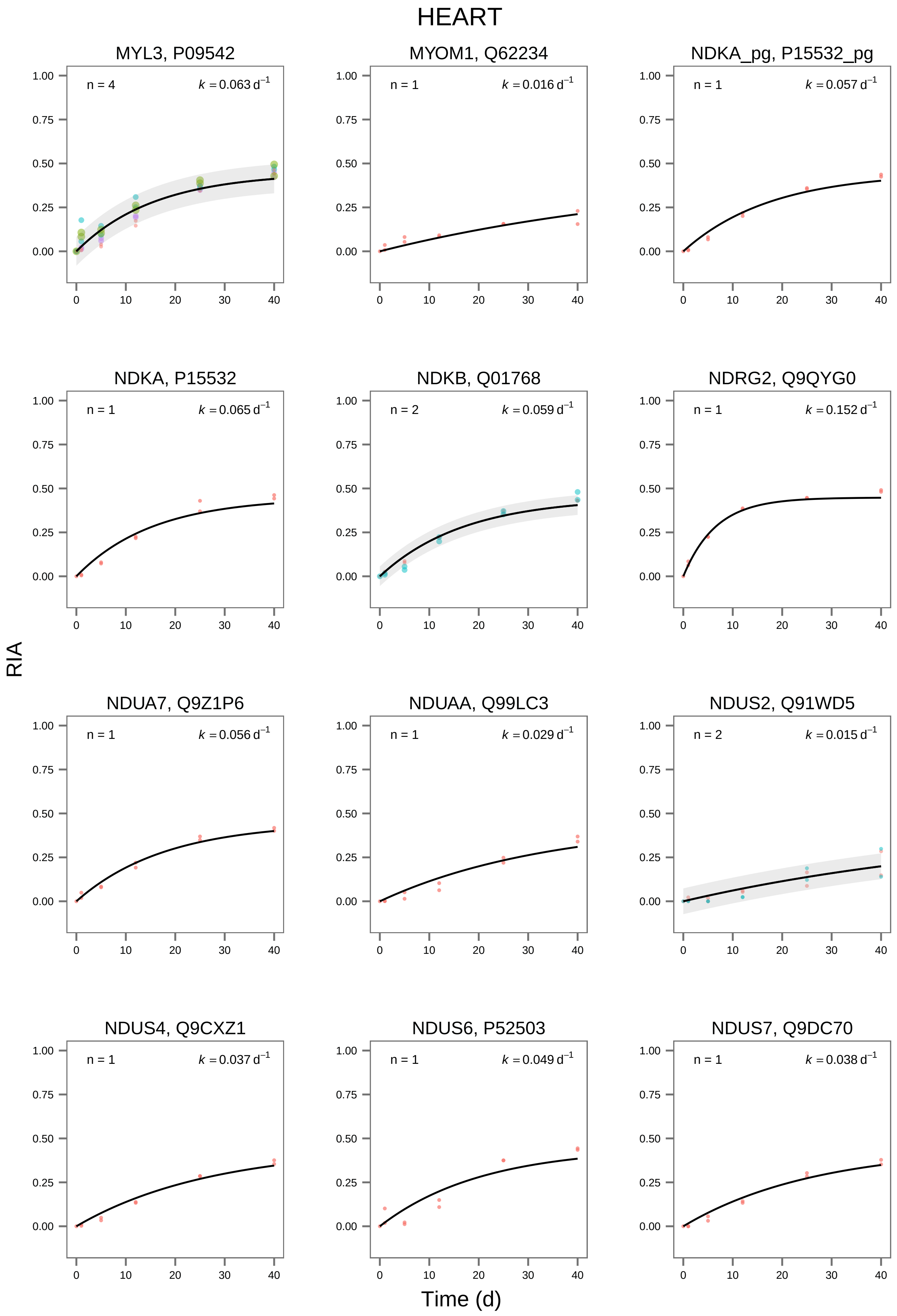
HEART
MYL3, P09542
n = 4
k = 0.063 d^-1
MYOM1, Q62234
n = 1
k = 0.016 d^-1
NDKA_pg, P15532_pg
n = 1
k = 0.057 d^-1
NDKA, P15532
n = 1
k = 0.065 d^-1
NDKB, Q01768
n = 2
k = 0.059 d^-1
NDRG2, Q9QYG0
n = 1
k = 0.152 d^-1
NDUA7, Q9Z1P6
n = 1
k = 0.056 d^-1
NDUAA, Q99LC3
n = 1
k = 0.029 d^-1
NDUS2, Q91WD5
n = 2
k = 0.015 d^-1
NDUS4, Q9CXZ1
n = 1
k = 0.037 d^-1
NDUS6, P52503
n = 1
k = 0.049 d^-1
NDUS7, Q9DC70
n = 1
k = 0.038 d^-1
RIA
Time (d)

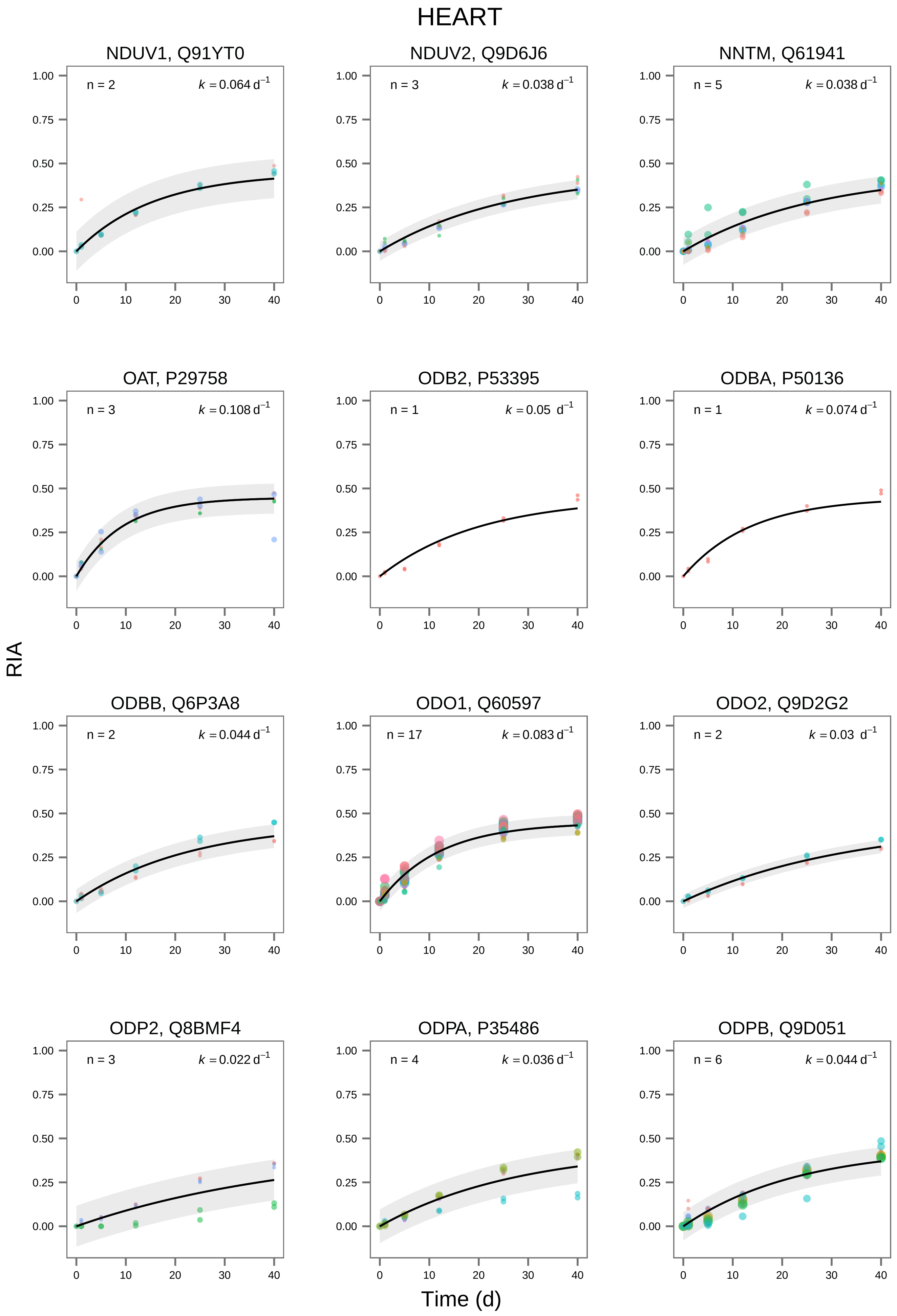
HEART
NDUV1, Q91YT0
n = 2
k = 0.064 d⁻¹
NDUV2, Q9D6J6
n = 3
k = 0.038 d⁻¹
NNTM, Q61941
n = 5
k = 0.038 d⁻¹
OAT, P29758
n = 3
k = 0.108 d⁻¹
ODB2, P53395
n = 1
k = 0.05 d⁻¹
ODBA, P50136
n = 1
k = 0.074 d⁻¹
ODBB, Q6P3A8
n = 2
k = 0.044 d⁻¹
ODO1, Q60597
n = 17
k = 0.083 d⁻¹
ODO2, Q9D2G2
n = 2
k = 0.03 d⁻¹
ODP2, Q8BMF4
n = 3
k = 0.022 d⁻¹
ODPA, P35486
n = 4
k = 0.036 d⁻¹
ODPB, Q9D051
n = 6
k = 0.044 d⁻¹
RIA
Time (d)
0.00
0.25
0.50
0.75
1.00
0
10
20
30
40

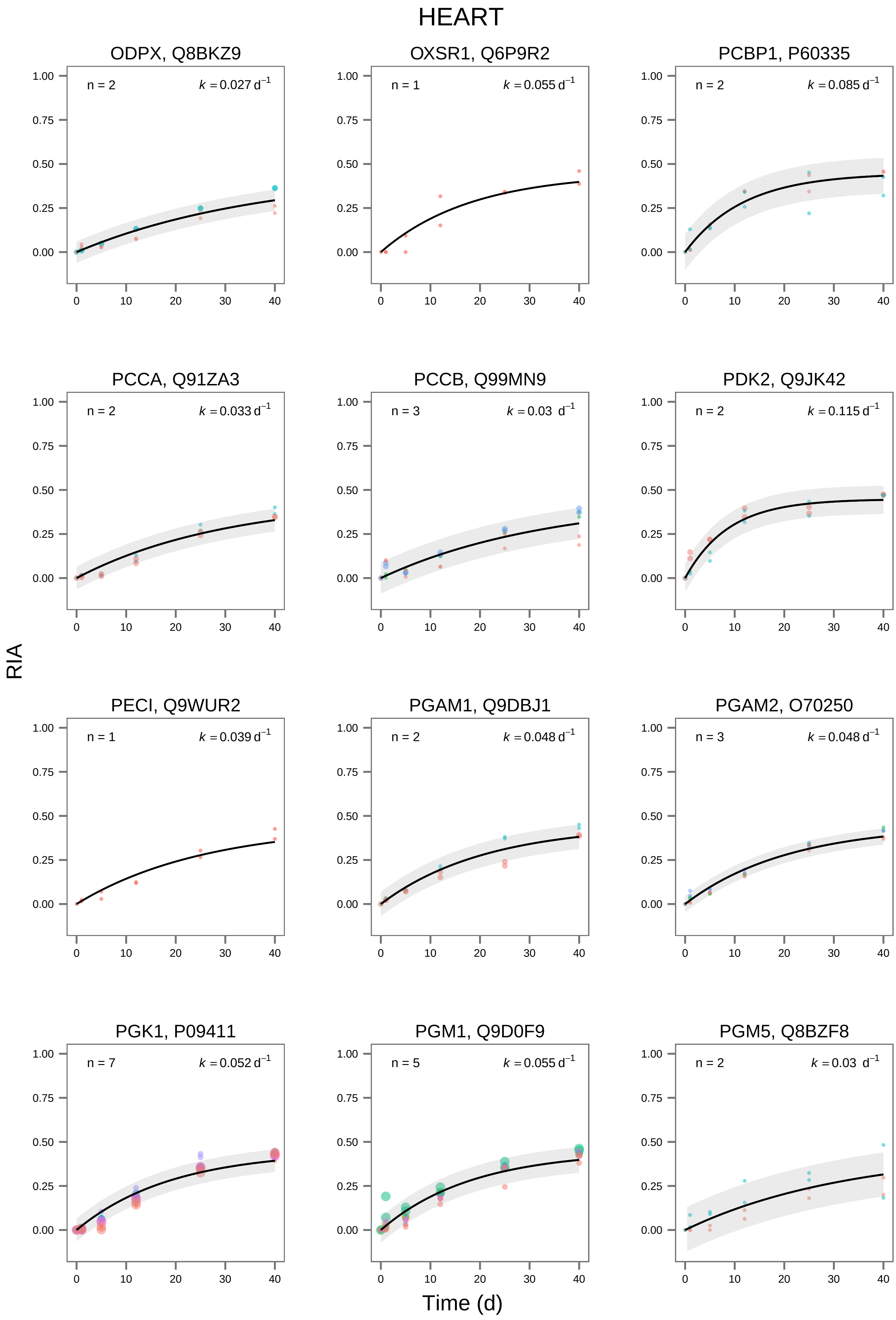
HEART
ODPX, Q8BKZ9
n = 2
k = 0.027 d⁻¹
OXSR1, Q6P9R2
n = 1
k = 0.055 d⁻¹
PCBP1, P60335
n = 2
k = 0.085 d⁻¹
PCCA, Q91ZA3
n = 2
k = 0.033 d⁻¹
PCCB, Q99MN9
n = 3
k = 0.03 d⁻¹
PDK2, Q9JK42
n = 2
k = 0.115 d⁻¹
PECI, Q9WUR2
n = 1
k = 0.039 d⁻¹
PGAM1, Q9DBJ1
n = 2
k = 0.048 d⁻¹
PGAM2, O70250
n = 3
k = 0.048 d⁻¹
PGK1, P09411
n = 7
k = 0.052 d⁻¹
PGM1, Q9D0F9
n = 5
k = 0.055 d⁻¹
PGM5, Q8BZF8
n = 2
k = 0.03 d⁻¹
RIA
Time (d)
0.00
0.25
0.50
0.75
1.00
0
10
20
30
40

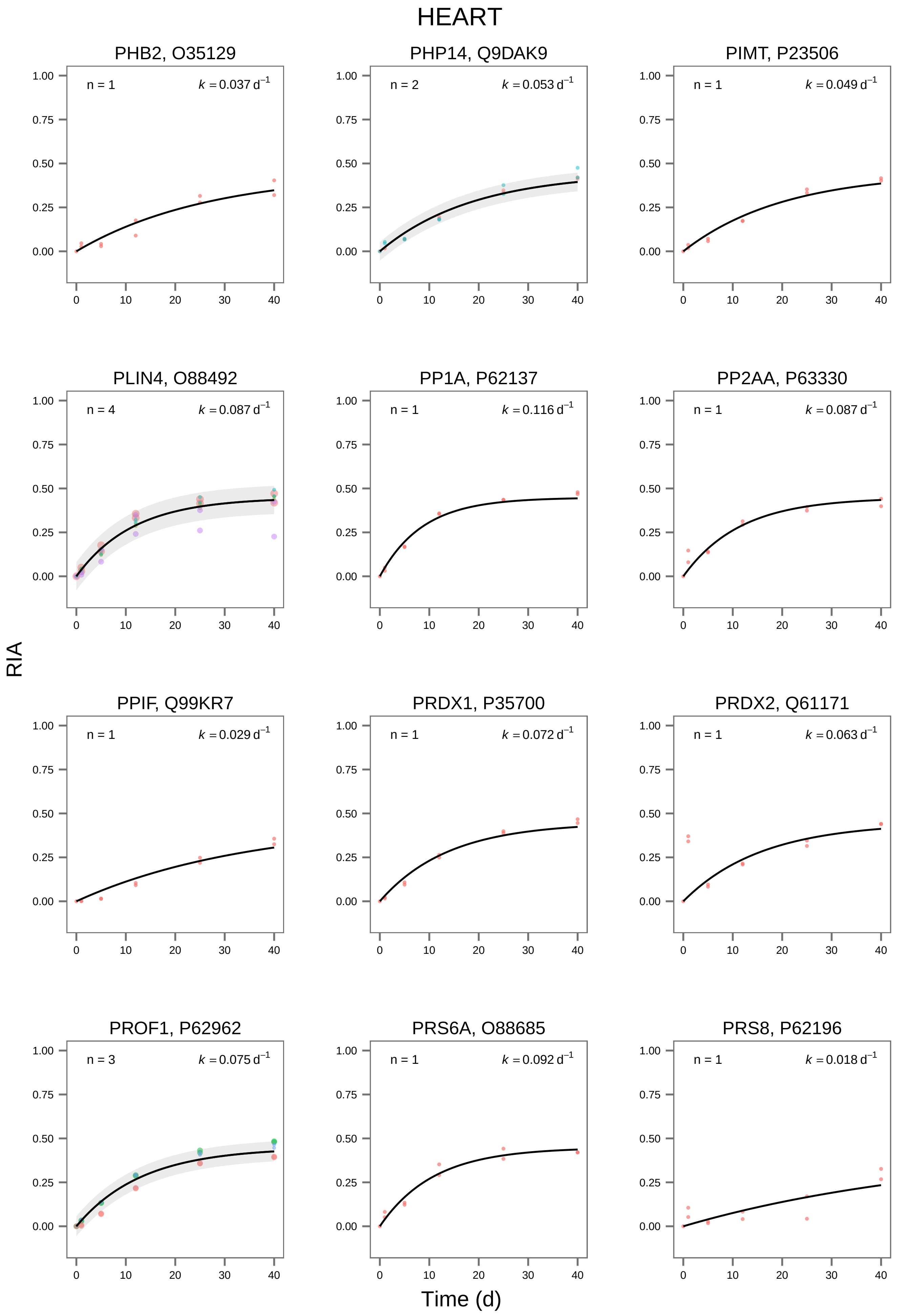
HEART
PHB2, O35129
n = 1
$k = 0.037\ d^{-1}$
PHP14, Q9DAK9
n = 2
$k = 0.053\ d^{-1}$
PIMT, P23506
n = 1
$k = 0.049\ d^{-1}$
PLIN4, O88492
n = 4
$k = 0.087\ d^{-1}$
PP1A, P62137
n = 1
$k = 0.116\ d^{-1}$
PP2AA, P63330
n = 1
$k = 0.087\ d^{-1}$
PPIF, Q99KR7
n = 1
$k = 0.029\ d^{-1}$
PRDX1, P35700
n = 1
$k = 0.072\ d^{-1}$
PRDX2, Q61171
n = 1
$k = 0.063\ d^{-1}$
PROF1, P62962
n = 3
$k = 0.075\ d^{-1}$
PRS6A, O88685
n = 1
$k = 0.092\ d^{-1}$
PRS8, P62196
n = 1
$k = 0.018\ d^{-1}$
RIA
Time (d)

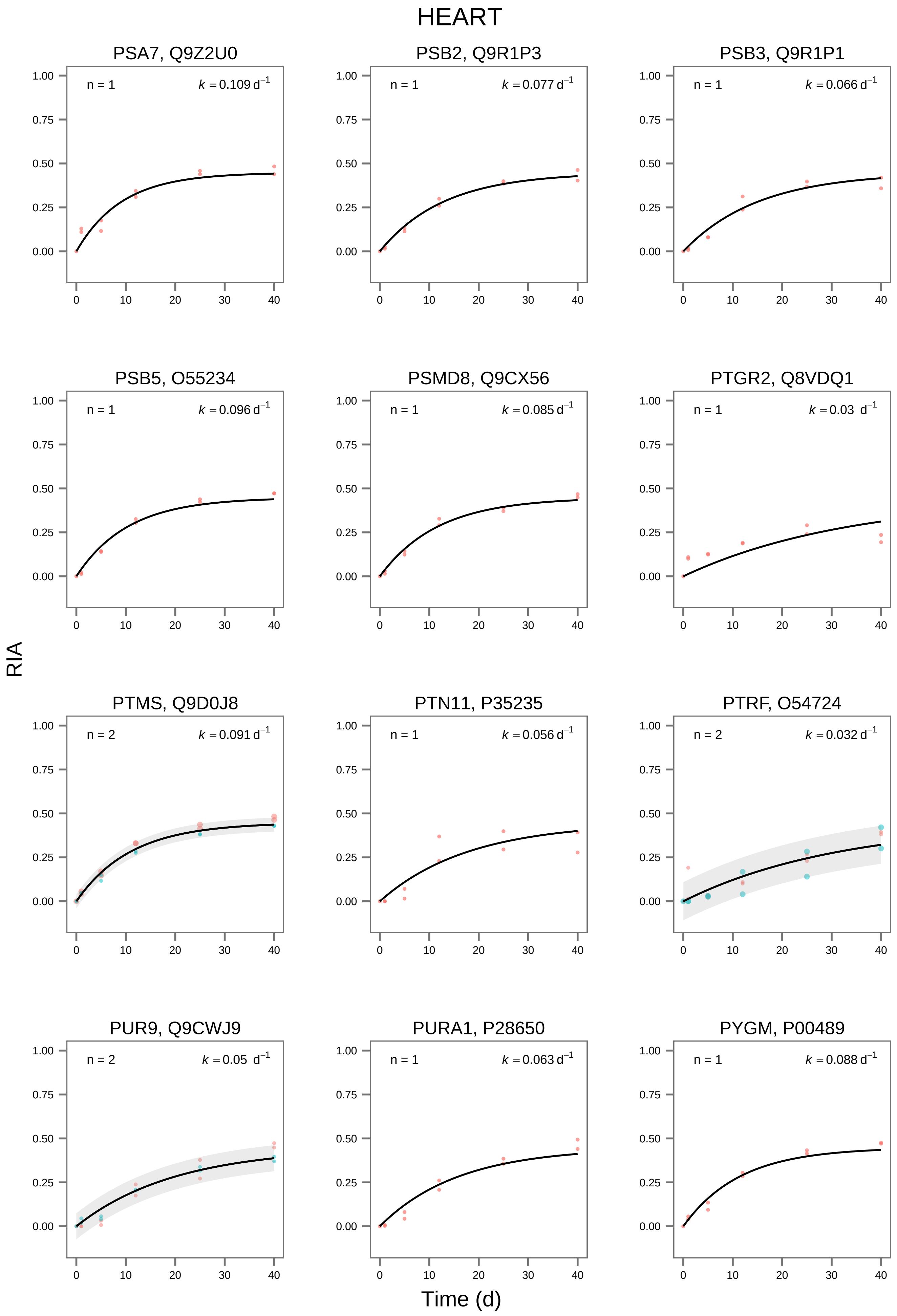
HEART
PSA7, Q9Z2U0
n = 1
k = 0.109 d^-1
PSB2, Q9R1P3
n = 1
k = 0.077 d^-1
PSB3, Q9R1P1
n = 1
k = 0.066 d^-1
PSB5, O55234
n = 1
k = 0.096 d^-1
PSMD8, Q9CX56
n = 1
k = 0.085 d^-1
PTGR2, Q8VDQ1
n = 1
k = 0.03 d^-1
PTMS, Q9D0J8
n = 2
k = 0.091 d^-1
PTN11, P35235
n = 1
k = 0.056 d^-1
PTRF, O54724
n = 2
k = 0.032 d^-1
PUR9, Q9CWJ9
n = 2
k = 0.05 d^-1
PURA1, P28650
n = 1
k = 0.063 d^-1
PYGM, P00489
n = 1
k = 0.088 d^-1
RIA
Time (d)
0.00
0.25
0.50
0.75
1.00
0
10
20
30
40

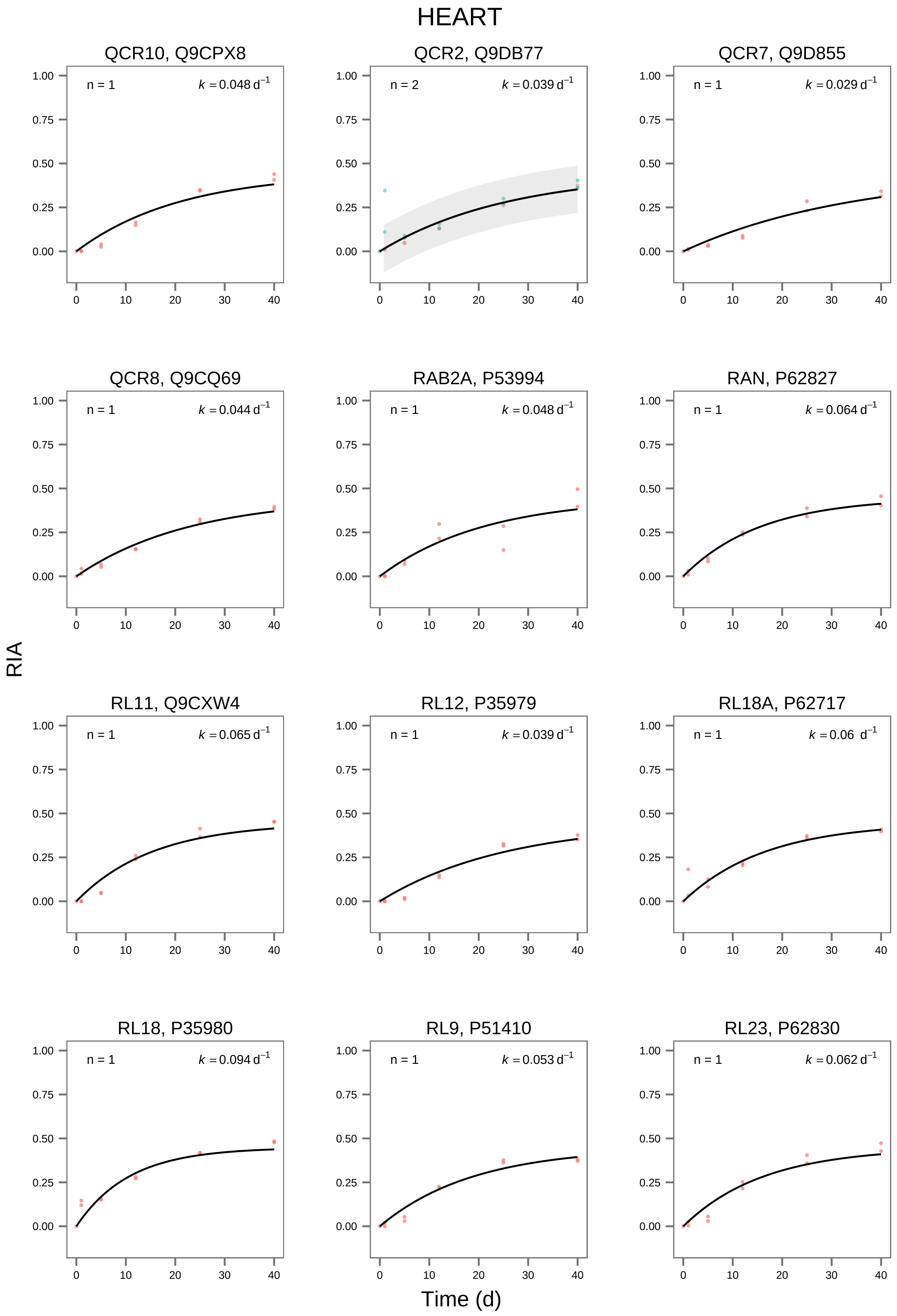
HEART
QCR10, Q9CPX8
n = 1
k = 0.048 d−1
QCR2, Q9DB77
n = 2
k = 0.039 d−1
QCR7, Q9D855
n = 1
k = 0.029 d−1
QCR8, Q9CQ69
n = 1
k = 0.044 d−1
RAB2A, P53994
n = 1
k = 0.048 d−1
RAN, P62827
n = 1
k = 0.064 d−1
RL11, Q9CXW4
n = 1
k = 0.065 d−1
RL12, P35979
n = 1
k = 0.039 d−1
RL18A, P62717
n = 1
k = 0.06 d−1
RL18, P35980
n = 1
k = 0.094 d−1
RL9, P51410
n = 1
k = 0.053 d−1
RL23, P62830
n = 1
k = 0.062 d−1
RIA
Time (d)
1.00
0.75
0.50
0.25
0.00
0
10
20
30
40

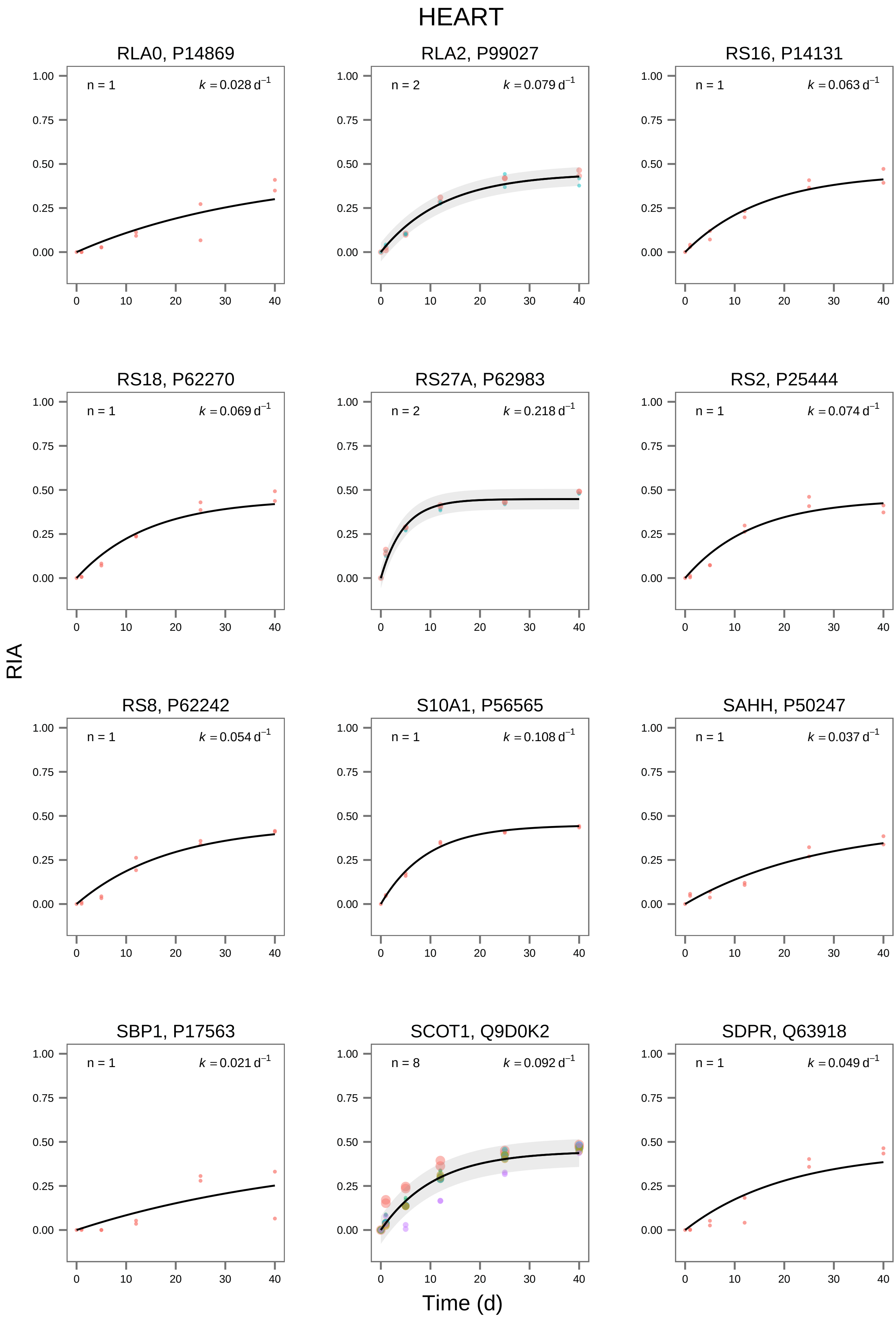
HEART
RLA0, P14869
n = 1
k = 0.028 d^−1
RLA2, P99027
n = 2
k = 0.079 d^−1
RS16, P14131
n = 1
k = 0.063 d^−1
RS18, P62270
n = 1
k = 0.069 d^−1
RS27A, P62983
n = 2
k = 0.218 d^−1
RS2, P25444
n = 1
k = 0.074 d^−1
RS8, P62242
n = 1
k = 0.054 d^−1
S10A1, P56565
n = 1
k = 0.108 d^−1
SAHH, P50247
n = 1
k = 0.037 d^−1
SBP1, P17563
n = 1
k = 0.021 d^−1
SCOT1, Q9D0K2
n = 8
k = 0.092 d^−1
SDPR, Q63918
n = 1
k = 0.049 d^−1
RIA
Time (d)
1.00
0.75
0.50
0.25
0.00
0
10
20
30
40

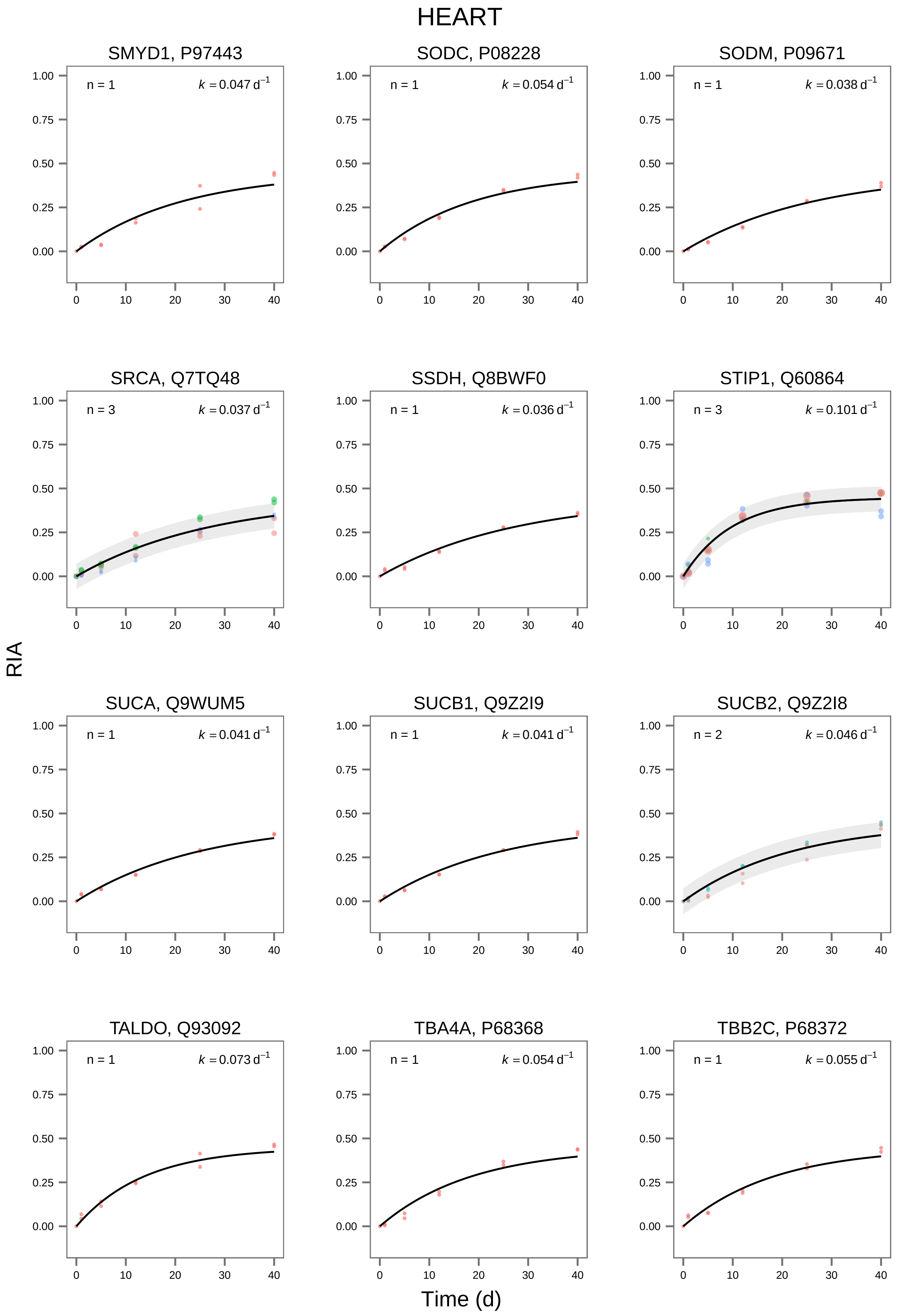

HEART
SMYD1, P97443
n = 1
k = 0.047 d⁻¹
SODC, P08228
n = 1
k = 0.054 d⁻¹
SODM, P09671
n = 1
k = 0.038 d⁻¹
SRCA, Q7TQ48
n = 3
k = 0.037 d⁻¹
SSDH, Q8BWF0
n = 1
k = 0.036 d⁻¹
STIP1, Q60864
n = 3
k = 0.101 d⁻¹
SUCA, Q9WUM5
n = 1
k = 0.041 d⁻¹
SUCB1, Q9Z2I9
n = 1
k = 0.041 d⁻¹
SUCB2, Q9Z2I8
n = 2
k = 0.046 d⁻¹
TALDO, Q93092
n = 1
k = 0.073 d⁻¹
TBA4A, P68368
n = 1
k = 0.054 d⁻¹
TBB2C, P68372
n = 1
k = 0.055 d⁻¹
RIA
Time (d)
0.00
0.25
0.50
0.75
1.00
0
10
20
30
40

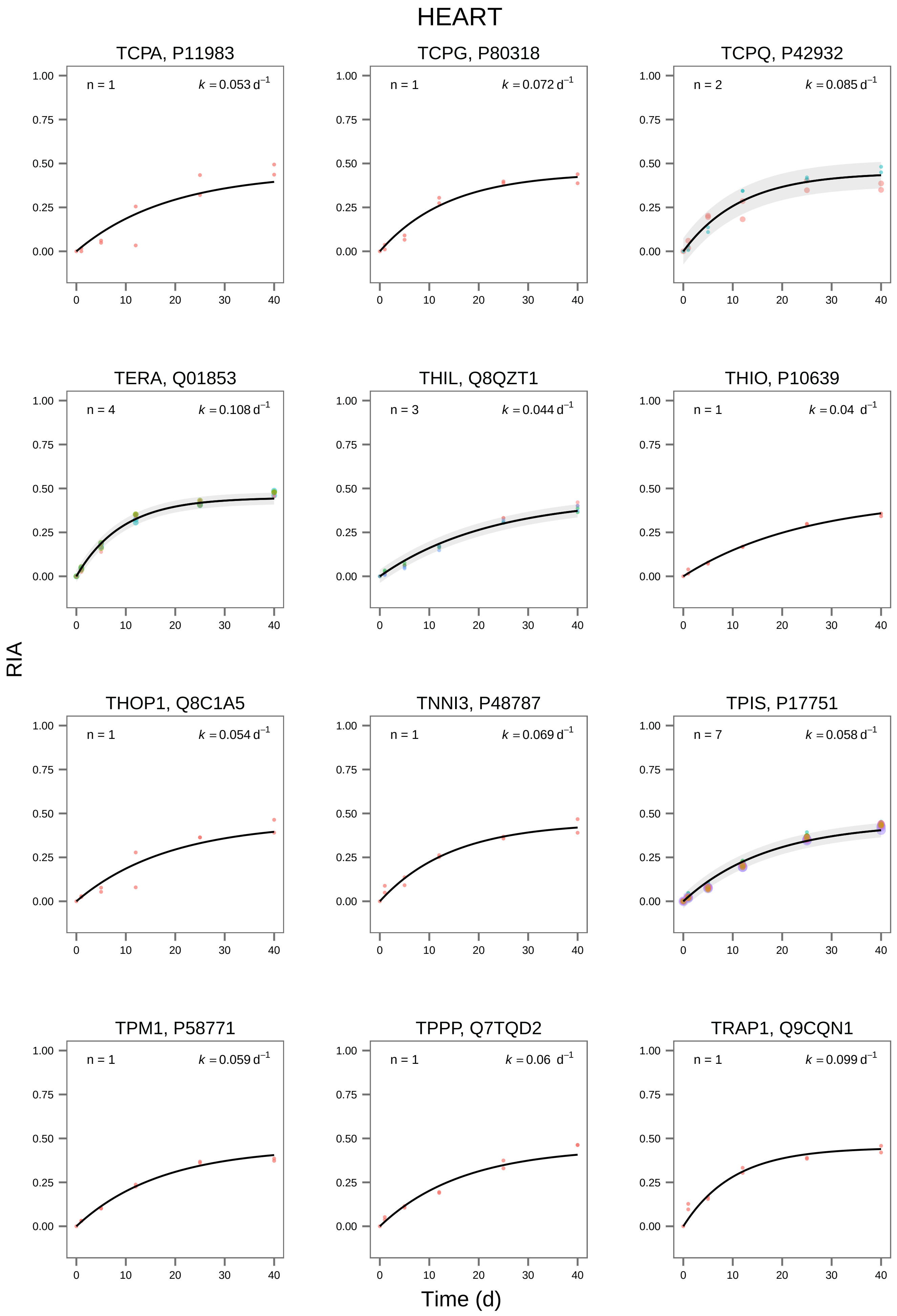
HEART
TCPA, P11983
n = 1
k = 0.053 d⁻¹
TCPG, P80318
n = 1
k = 0.072 d⁻¹
TCPQ, P42932
n = 2
k = 0.085 d⁻¹
TERA, Q01853
n = 4
k = 0.108 d⁻¹
THIL, Q8QZT1
n = 3
k = 0.044 d⁻¹
THIO, P10639
n = 1
k = 0.04 d⁻¹
THOP1, Q8C1A5
n = 1
k = 0.054 d⁻¹
TNNI3, P48787
n = 1
k = 0.069 d⁻¹
TPIS, P17751
n = 7
k = 0.058 d⁻¹
TPM1, P58771
n = 1
k = 0.059 d⁻¹
TPPP, Q7TQD2
n = 1
k = 0.06 d⁻¹
TRAP1, Q9CQN1
n = 1
k = 0.099 d⁻¹
RIA
Time (d)

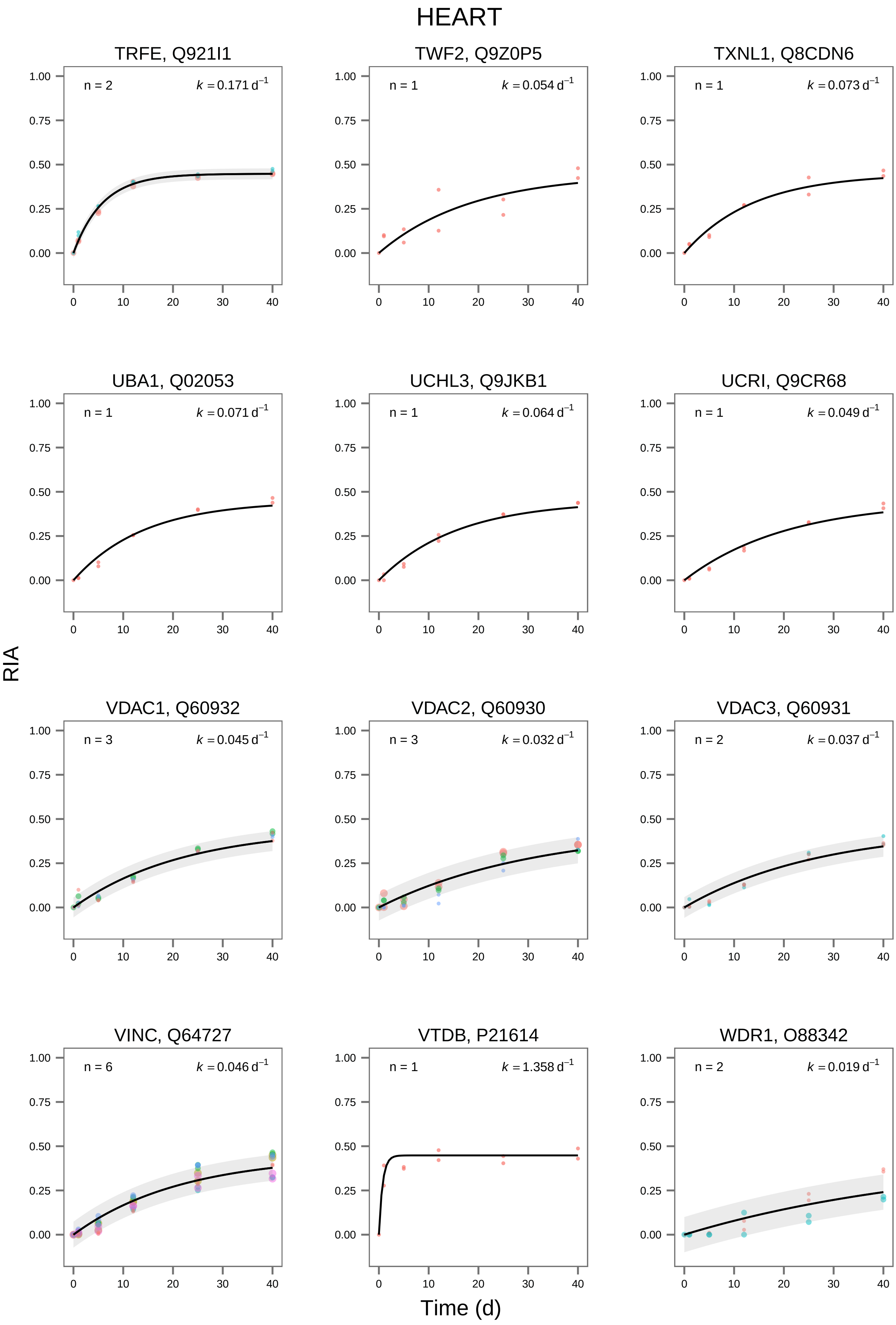
HEARTH
TRFE, Q921I1
n = 2
k = 0.171 d−1
TWF2, Q9Z0P5
n = 1
k = 0.054 d−1
TXNL1, Q8CDN6
n = 1
k = 0.073 d−1
UBA1, Q02053
n = 1
k = 0.071 d−1
UCHL3, Q9JKB1
n = 1
k = 0.064 d−1
UCRI, Q9CR68
n = 1
k = 0.049 d−1
VDAC1, Q60932
n = 3
k = 0.045 d−1
VDAC2, Q60930
n = 3
k = 0.032 d−1
VDAC3, Q60931
n = 2
k = 0.037 d−1
VINC, Q64727
n = 6
k = 0.046 d−1
VTDB, P21614
n = 1
k = 1.358 d−1
WDR1, O88342
n = 2
k = 0.019 d−1
RIA
Time (d)

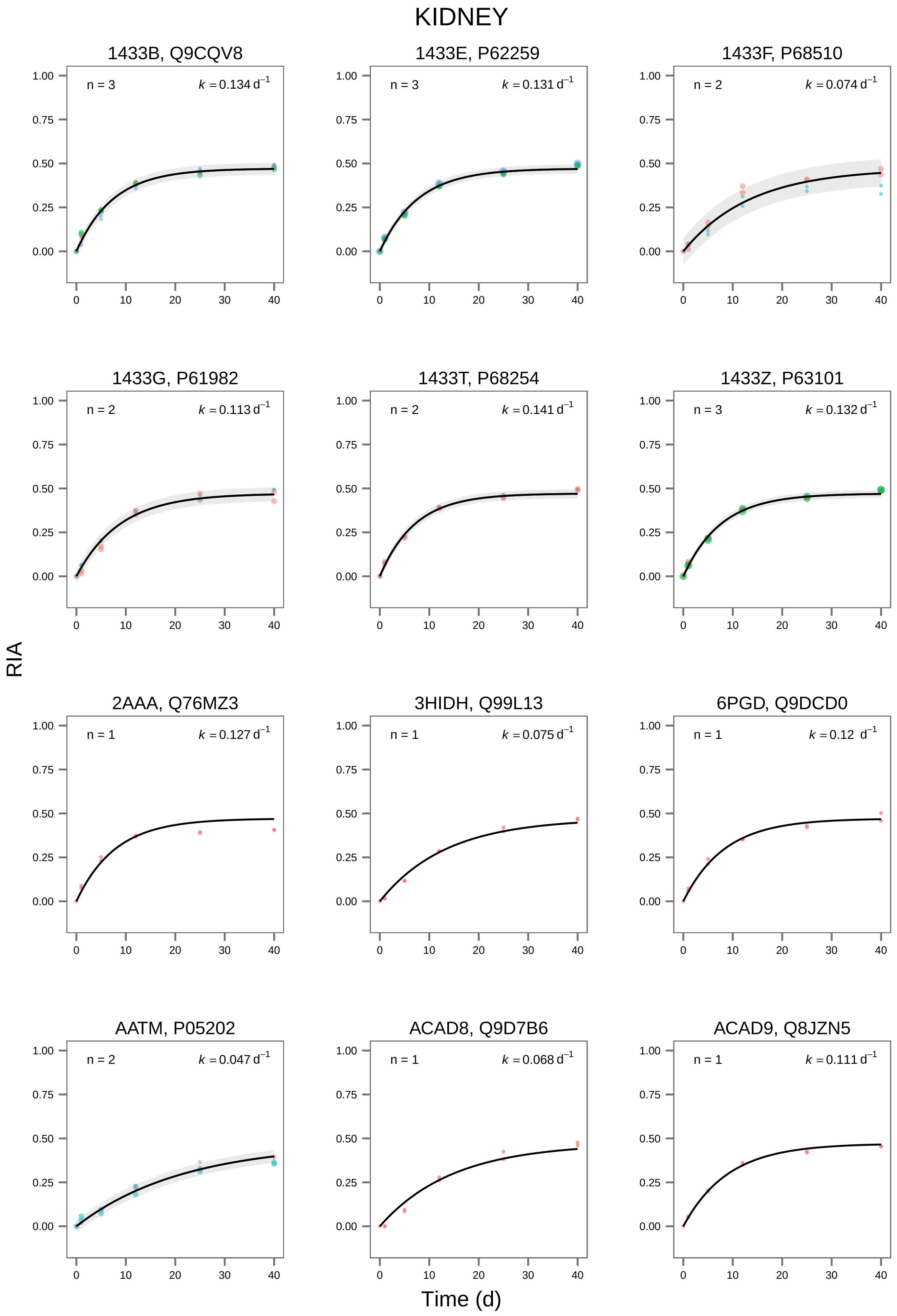
KIDNEY
1433B, Q9CQV8
n = 3
k = 0.134 d⁻¹
1433E, P62259
n = 3
k = 0.131 d⁻¹
1433F, P68510
n = 2
k = 0.074 d⁻¹
1433G, P61982
n = 2
k = 0.113 d⁻¹
1433T, P68254
n = 2
k = 0.141 d⁻¹
1433Z, P63101
n = 3
k = 0.132 d⁻¹
2AAA, Q76MZ3
n = 1
k = 0.127 d⁻¹
3HIDH, Q99L13
n = 1
k = 0.075 d⁻¹
6PGD, Q9DCD0
n = 1
k = 0.12 d⁻¹
AATM, P05202
n = 2
k = 0.047 d⁻¹
ACAD8, Q9D7B6
n = 1
k = 0.068 d⁻¹
ACAD9, Q8JZN5
n = 1
k = 0.111 d⁻¹
RIA
Time (d)

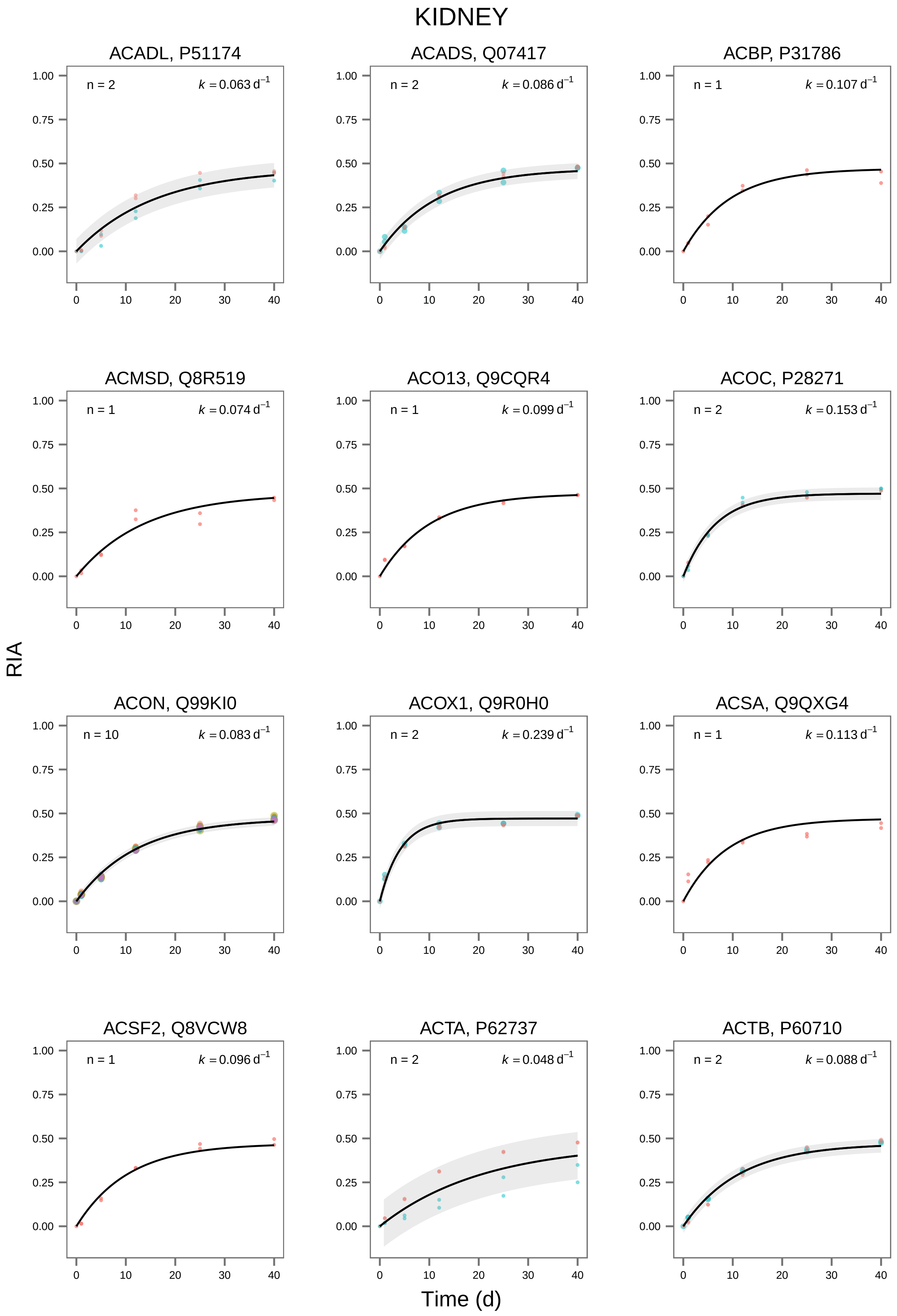
KIDNEY
ACADL, P51174
n = 2
k = 0.063 d^-1
ACADS, Q07417
n = 2
k = 0.086 d^-1
ACBP, P31786
n = 1
k = 0.107 d^-1
ACMSD, Q8R519
n = 1
k = 0.074 d^-1
ACO13, Q9CQR4
n = 1
k = 0.099 d^-1
ACOC, P28271
n = 2
k = 0.153 d^-1
ACON, Q99KI0
n = 10
k = 0.083 d^-1
ACOX1, Q9R0H0
n = 2
k = 0.239 d^-1
ACSA, Q9QXG4
n = 1
k = 0.113 d^-1
ACSF2, Q8VCW8
n = 1
k = 0.096 d^-1
ACTA, P62737
n = 2
k = 0.048 d^-1
ACTB, P60710
n = 2
k = 0.088 d^-1
RIA
Time (d)

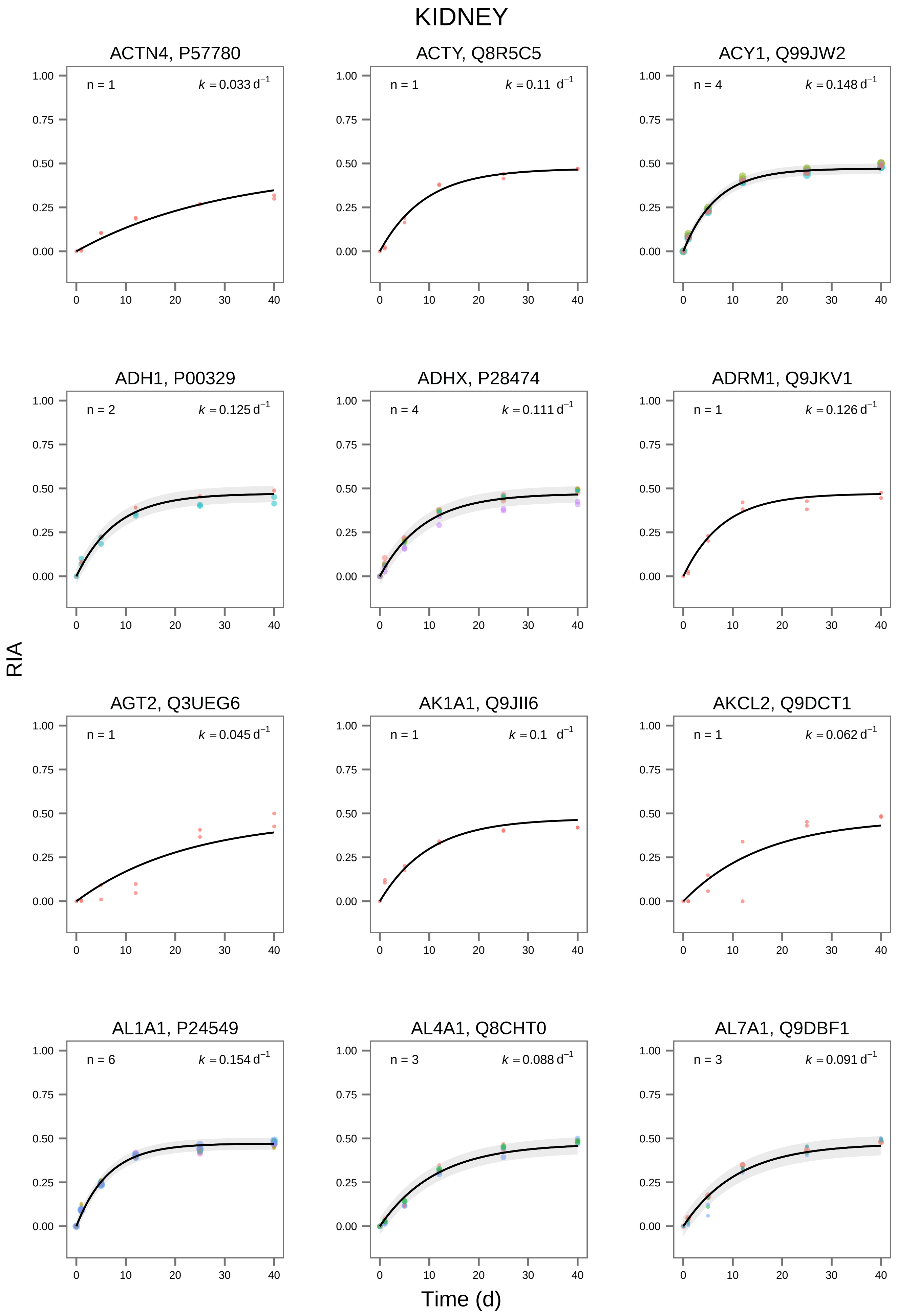
KIDNEY
ACTN4, P57780
n = 1
k = 0.033 d−1
ACTY, Q8R5C5
n = 1
k = 0.11 d−1
ACY1, Q99JW2
n = 4
k = 0.148 d−1
ADH1, P00329
n = 2
k = 0.125 d−1
ADHX, P28474
n = 4
k = 0.111 d−1
ADRM1, Q9JKV1
n = 1
k = 0.126 d−1
AGT2, Q3UEG6
n = 1
k = 0.045 d−1
AK1A1, Q9JII6
n = 1
k = 0.1 d−1
AKCL2, Q9DCT1
n = 1
k = 0.062 d−1
AL1A1, P24549
n = 6
k = 0.154 d−1
AL4A1, Q8CHT0
n = 3
k = 0.088 d−1
AL7A1, Q9DBF1
n = 3
k = 0.091 d−1
RIA
Time (d)

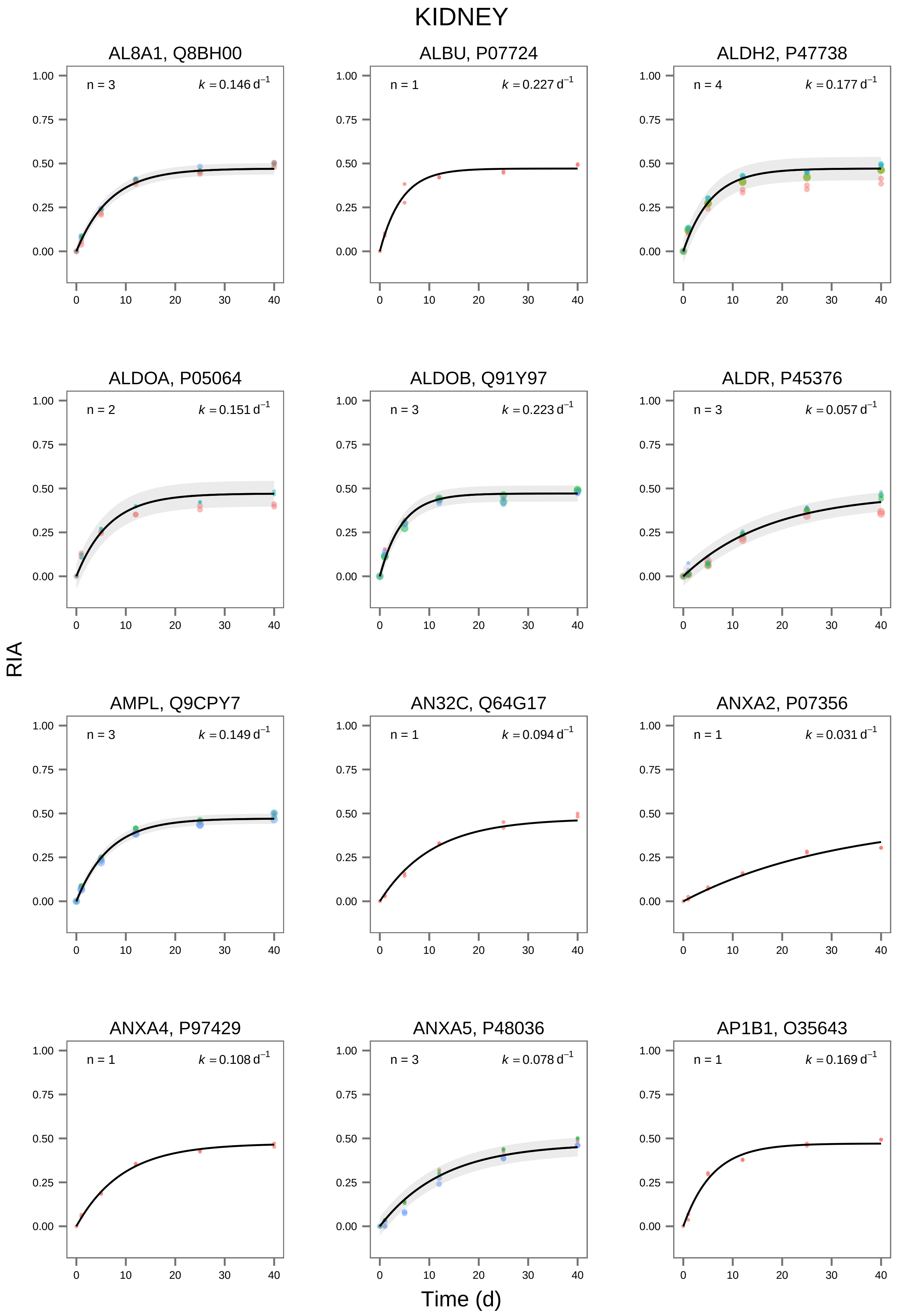
KIDNEY
AL8A1, Q8BH00
n = 3
k = 0.146 d−1
ALBU, P07724
n = 1
k = 0.227 d−1
ALDH2, P47738
n = 4
k = 0.177 d−1
ALDOA, P05064
n = 2
k = 0.151 d−1
ALDOB, Q91Y97
n = 3
k = 0.223 d−1
ALDR, P45376
n = 3
k = 0.057 d−1
AMPL, Q9CPY7
n = 3
k = 0.149 d−1
AN32C, Q64G17
n = 1
k = 0.094 d−1
ANXA2, P07356
n = 1
k = 0.031 d−1
ANXA4, P97429
n = 1
k = 0.108 d−1
ANXA5, P48036
n = 3
k = 0.078 d−1
AP1B1, O35643
n = 1
k = 0.169 d−1
RIA
Time (d)
0.00
0.25
0.50
0.75
1.00
0
10
20
30
40

KIDNEY

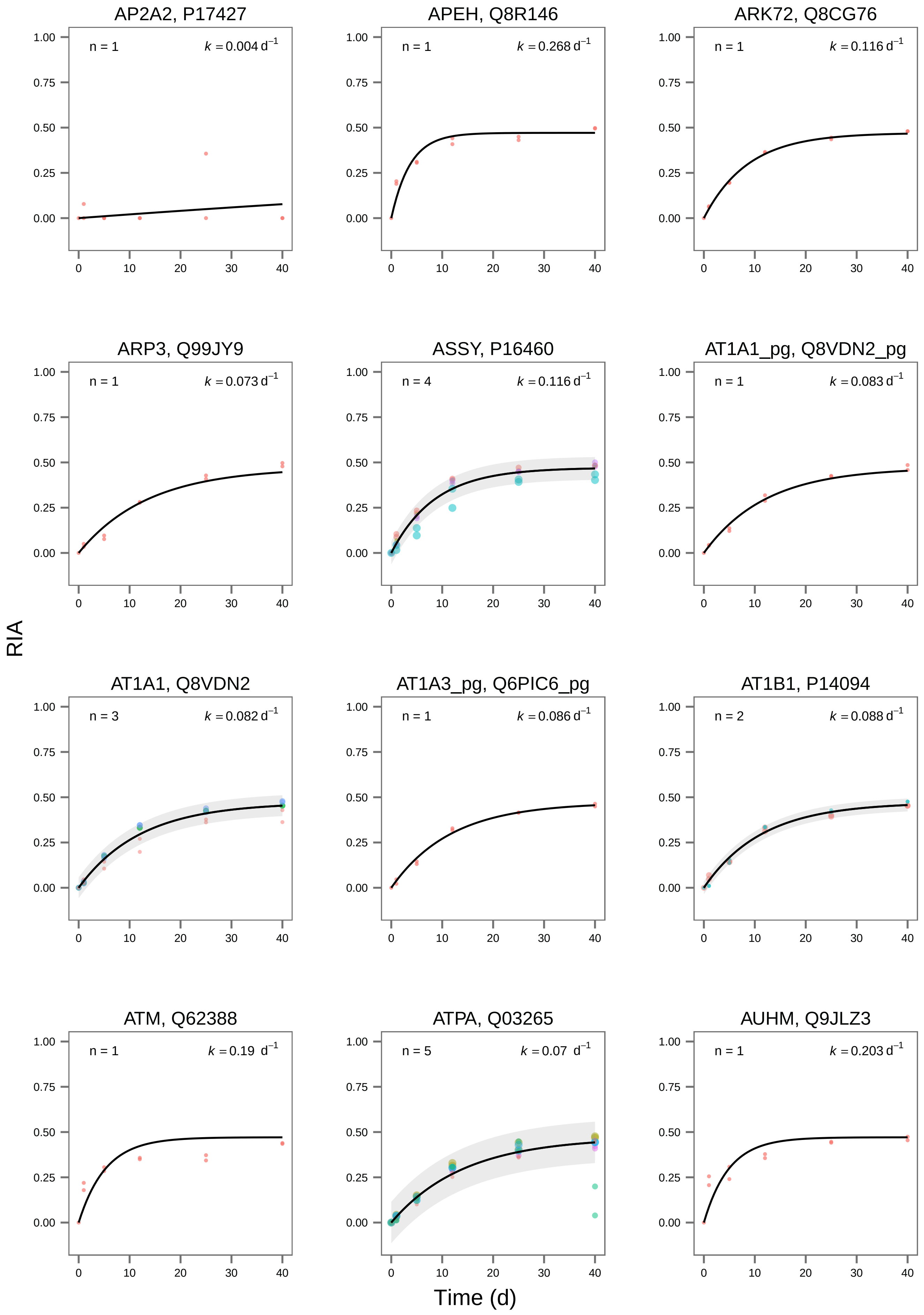
AP2A2, P17427
n = 1
k = 0.004 d−1
APEH, Q8R146
n = 1
k = 0.268 d−1
ARK72, Q8CG76
n = 1
k = 0.116 d−1
ARP3, Q99JY9
n = 1
k = 0.073 d−1
ASSY, P16460
n = 4
k = 0.116 d−1
AT1A1_pg, Q8VDN2_pg
n = 1
k = 0.083 d−1
AT1A1, Q8VDN2
n = 3
k = 0.082 d−1
AT1A3_pg, Q6PIC6_pg
n = 1
k = 0.086 d−1
AT1B1, P14094
n = 2
k = 0.088 d−1
ATM, Q62388
n = 1
k = 0.19 d−1
ATPA, Q03265
n = 5
k = 0.07 d−1
AUHM, Q9JLZ3
n = 1
k = 0.203 d−1
RIA
Time (d)
0.00
0.25
0.50
0.75
1.00
0
10
20
30
40

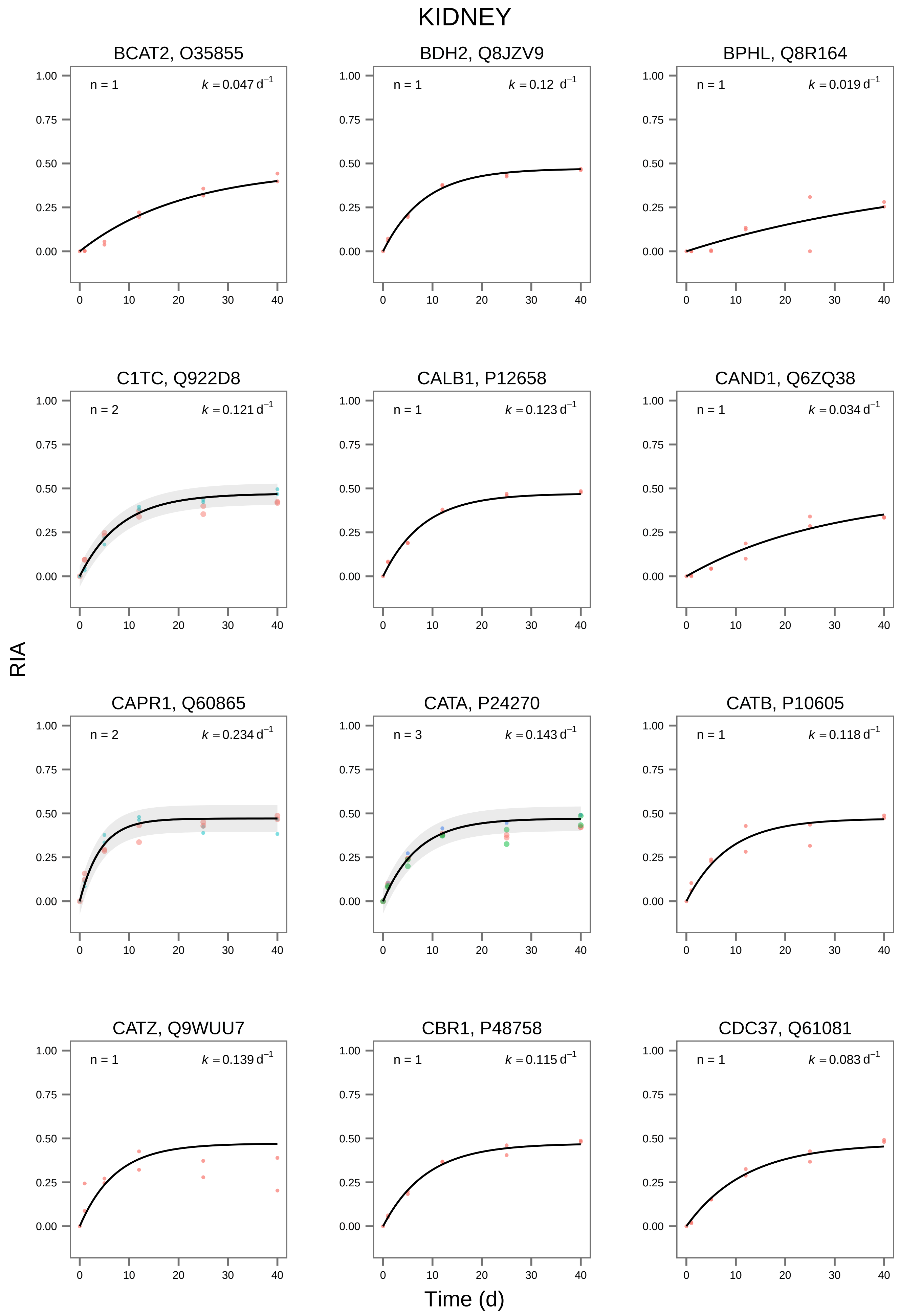
KIDNEY
BCAT2, O35855
n = 1
k = 0.047 d⁻¹
BDH2, Q8JZV9
n = 1
k = 0.12 d⁻¹
BPHL, Q8R164
n = 1
k = 0.019 d⁻¹
C1TC, Q922D8
n = 2
k = 0.121 d⁻¹
CALB1, P12658
n = 1
k = 0.123 d⁻¹
CAND1, Q6ZQ38
n = 1
k = 0.034 d⁻¹
CAPR1, Q60865
n = 2
k = 0.234 d⁻¹
CATA, P24270
n = 3
k = 0.143 d⁻¹
CATB, P10605
n = 1
k = 0.118 d⁻¹
CATZ, Q9WUU7
n = 1
k = 0.139 d⁻¹
CBR1, P48758
n = 1
k = 0.115 d⁻¹
CDC37, Q61081
n = 1
k = 0.083 d⁻¹
RIA
Time (d)

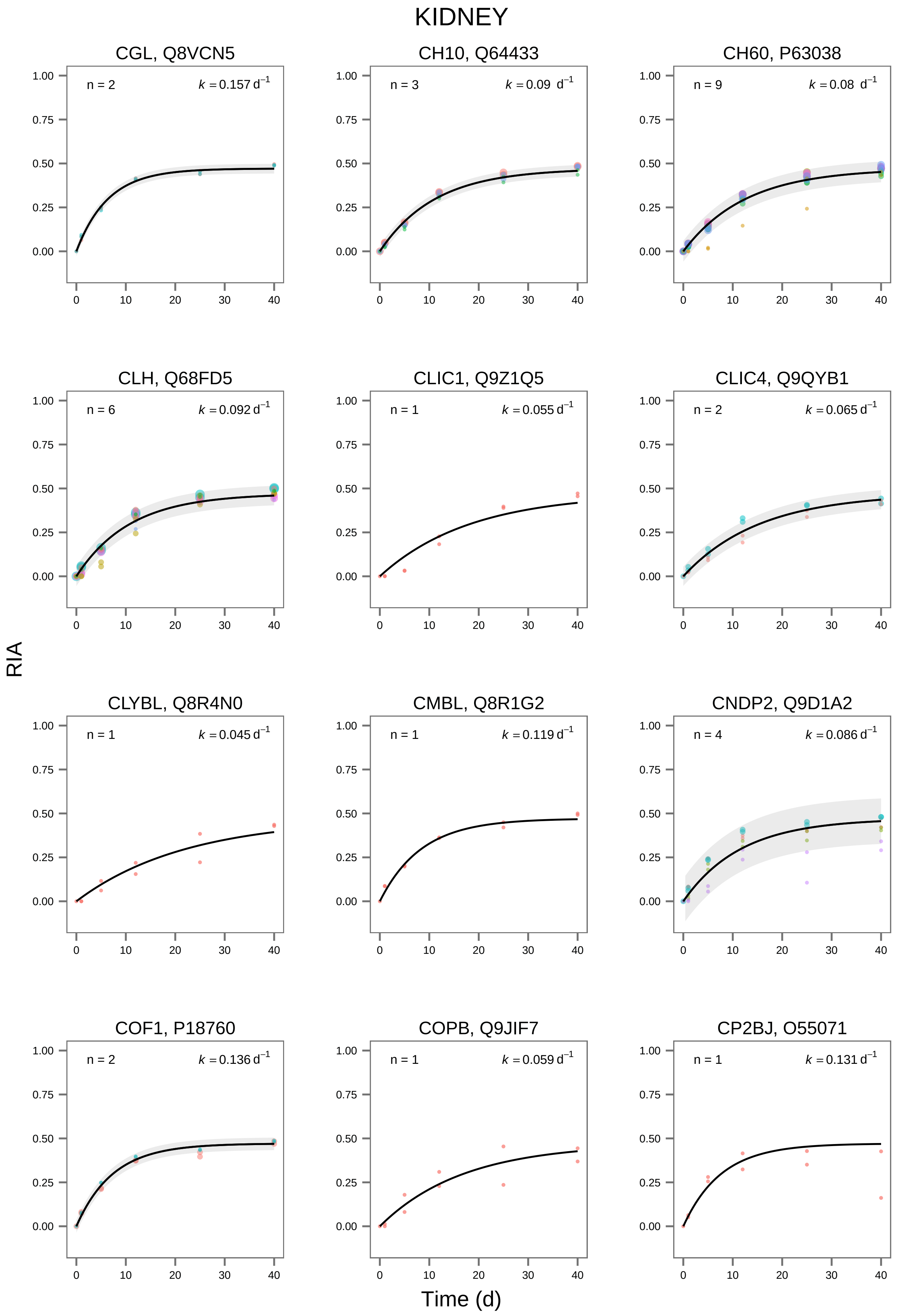
KIDNEY
CGL, Q8VCN5
n = 2
k = 0.157 d−1
CH10, Q64433
n = 3
k = 0.09 d−1
CH60, P63038
n = 9
k = 0.08 d−1
CLH, Q68FD5
n = 6
k = 0.092 d−1
CLIC1, Q9Z1Q5
n = 1
k = 0.055 d−1
CLIC4, Q9QYB1
n = 2
k = 0.065 d−1
CLYBL, Q8R4N0
n = 1
k = 0.045 d−1
CMBL, Q8R1G2
n = 1
k = 0.119 d−1
CNDP2, Q9D1A2
n = 4
k = 0.086 d−1
COF1, P18760
n = 2
k = 0.136 d−1
COPB, Q9JIF7
n = 1
k = 0.059 d−1
CP2BJ, O55071
n = 1
k = 0.131 d−1
RIA
Time (d)
0.00
0.25
0.50
0.75
1.00
0
10
20
30
40

KIDNEY

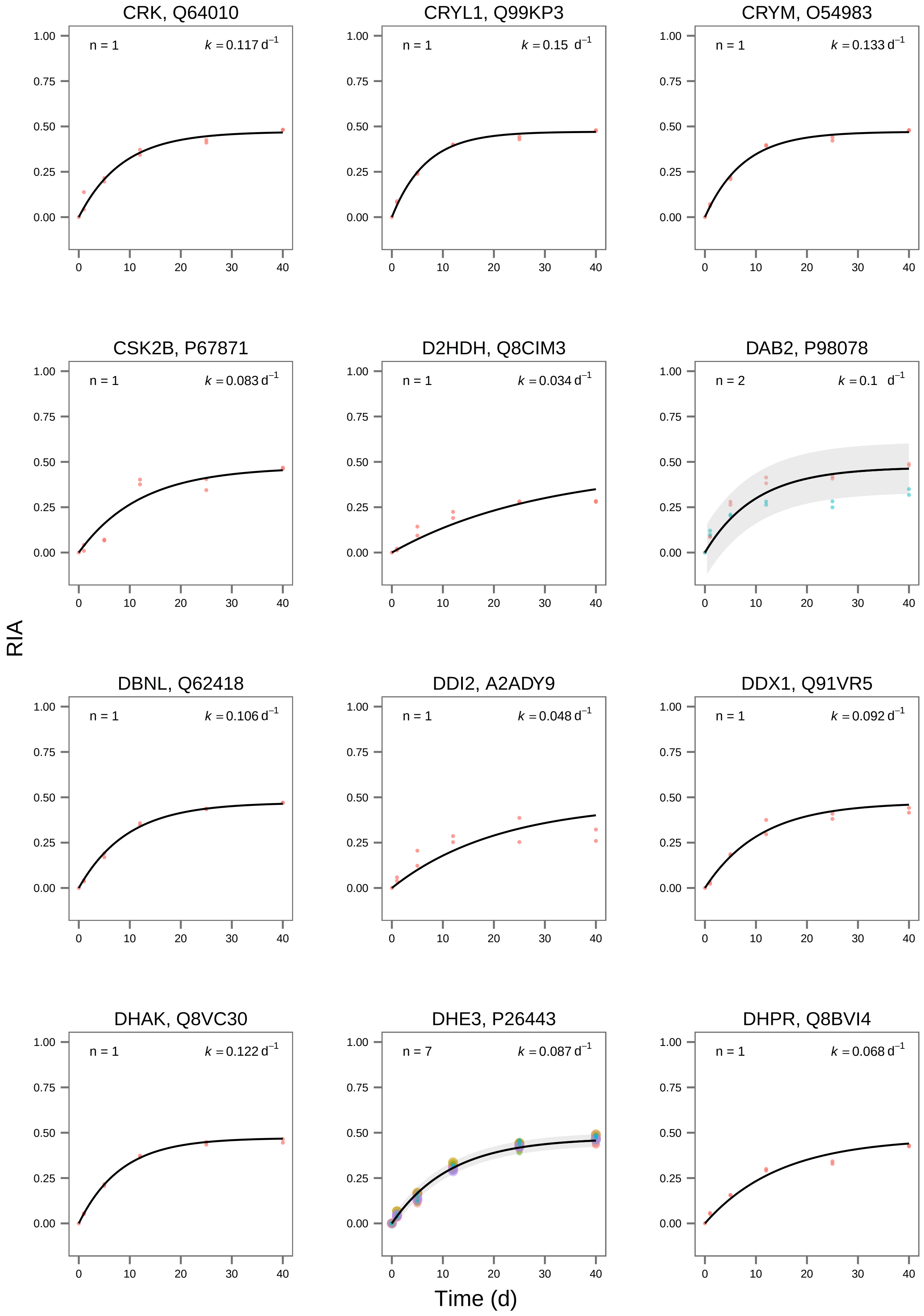

CRK, Q64010
n = 1
k = 0.117 d⁻¹
CRYL1, Q99KP3
n = 1
k = 0.15 d⁻¹
CRYM, O54983
n = 1
k = 0.133 d⁻¹
CSK2B, P67871
n = 1
k = 0.083 d⁻¹
D2HDH, Q8CIM3
n = 1
k = 0.034 d⁻¹
DAB2, P98078
n = 2
k = 0.1 d⁻¹
DBNL, Q62418
n = 1
k = 0.106 d⁻¹
DDI2, A2ADY9
n = 1
k = 0.048 d⁻¹
DDX1, Q91VR5
n = 1
k = 0.092 d⁻¹
DHAK, Q8VC30
n = 1
k = 0.122 d⁻¹
DHE3, P26443
n = 7
k = 0.087 d⁻¹
DHPR, Q8BVI4
n = 1
k = 0.068 d⁻¹
RIA
Time (d)

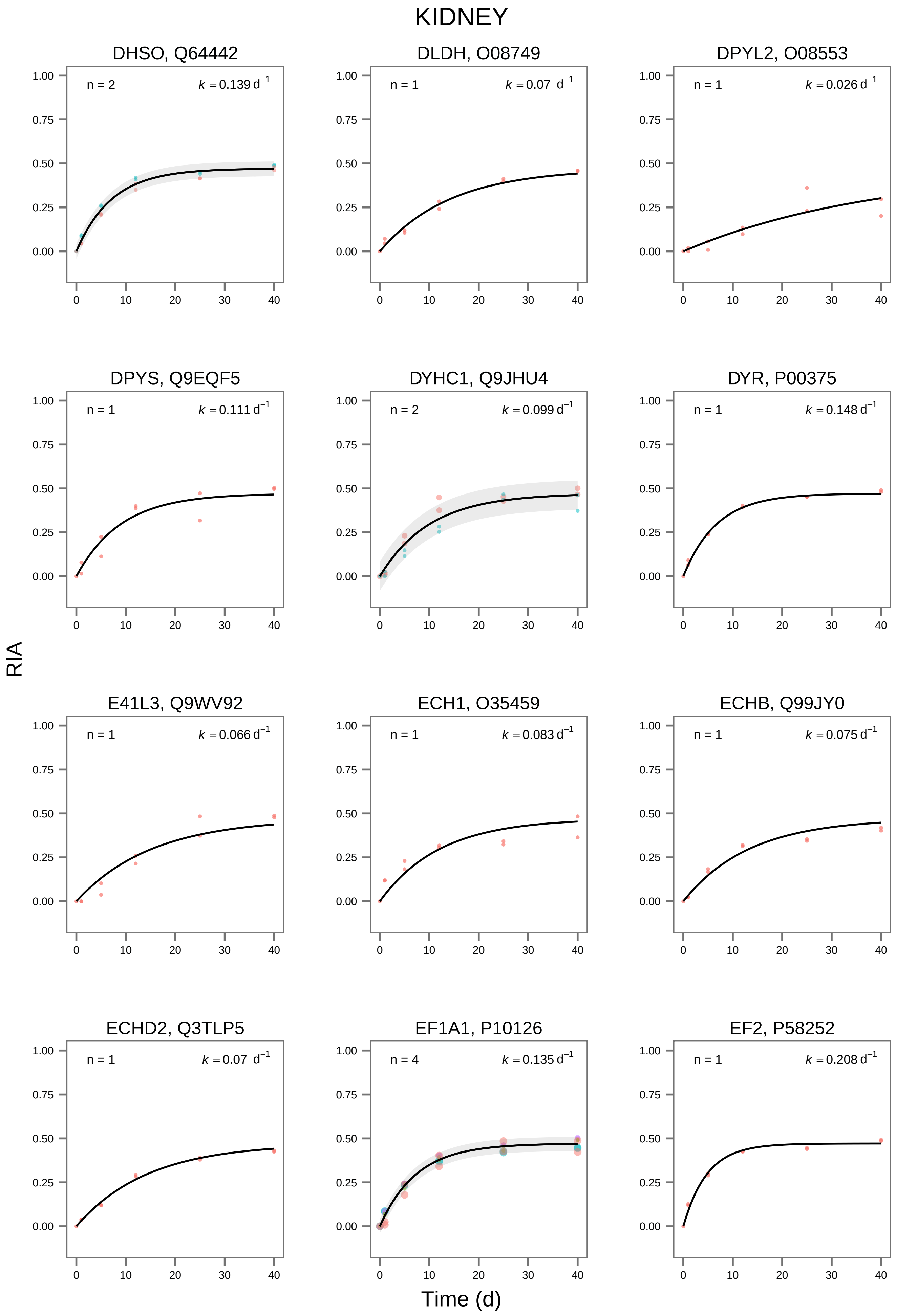
KIDNEY
DHSO, Q64442
n = 2
k = 0.139 d⁻¹
DLDH, O08749
n = 1
k = 0.07 d⁻¹
DPYL2, O08553
n = 1
k = 0.026 d⁻¹
DPYS, Q9EQF5
n = 1
k = 0.111 d⁻¹
DYHC1, Q9JHU4
n = 2
k = 0.099 d⁻¹
DYR, P00375
n = 1
k = 0.148 d⁻¹
E41L3, Q9WV92
n = 1
k = 0.066 d⁻¹
ECH1, O35459
n = 1
k = 0.083 d⁻¹
ECHB, Q99JY0
n = 1
k = 0.075 d⁻¹
ECHD2, Q3TLP5
n = 1
k = 0.07 d⁻¹
EF1A1, P10126
n = 4
k = 0.135 d⁻¹
EF2, P58252
n = 1
k = 0.208 d⁻¹
RIA
Time (d)
1.00
0.75
0.50
0.25
0.00
0
10
20
30
40

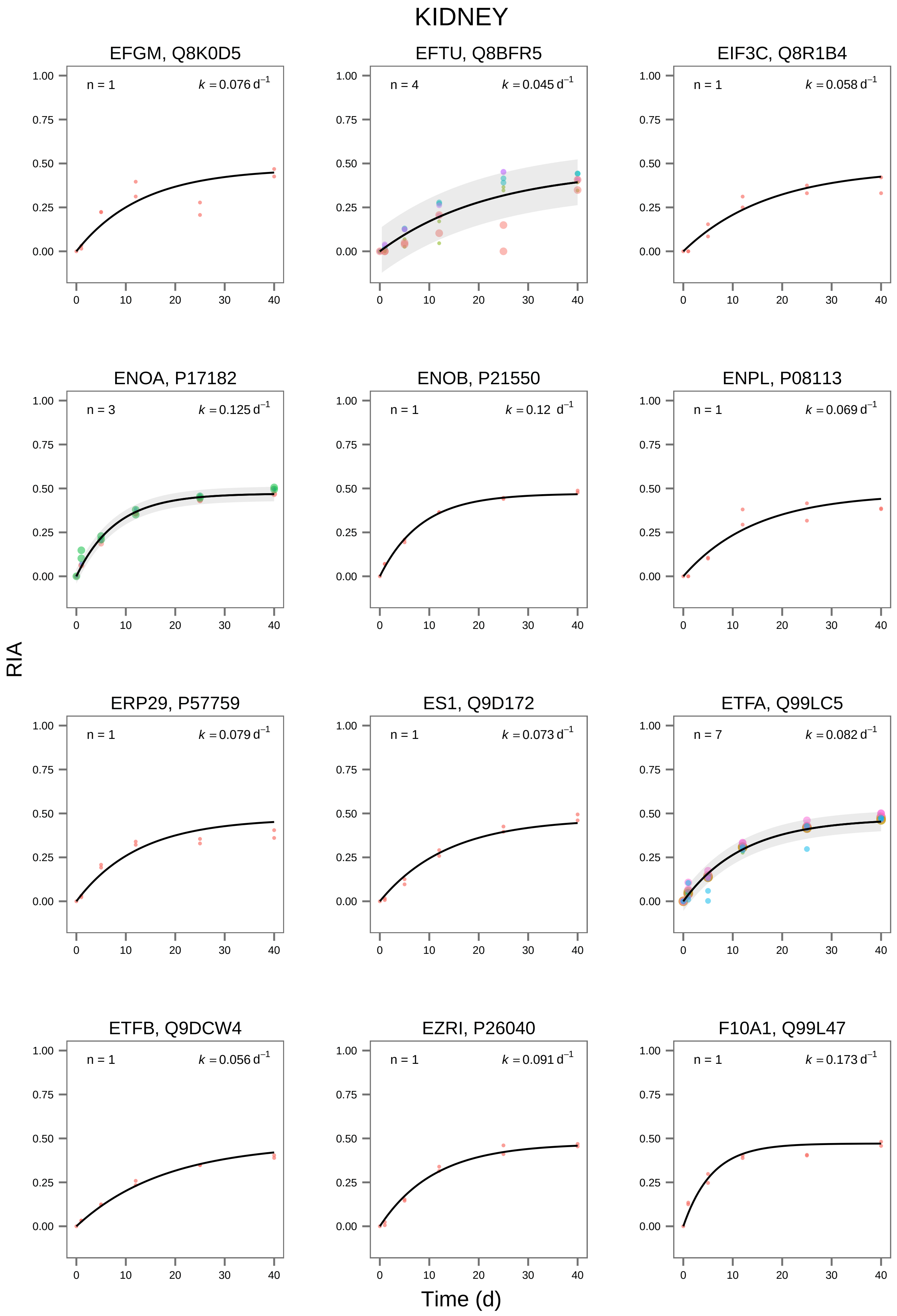

KIDNEY
EFGM, Q8K0D5
n = 1
k = 0.076 d−1
EFTU, Q8BFR5
n = 4
k = 0.045 d−1
EIF3C, Q8R1B4
n = 1
k = 0.058 d−1
ENOA, P17182
n = 3
k = 0.125 d−1
ENOB, P21550
n = 1
k = 0.12 d−1
ENPL, P08113
n = 1
k = 0.069 d−1
ERP29, P57759
n = 1
k = 0.079 d−1
ES1, Q9D172
n = 1
k = 0.073 d−1
ETFA, Q99LC5
n = 7
k = 0.082 d−1
ETFB, Q9DCW4
n = 1
k = 0.056 d−1
EZRI, P26040
n = 1
k = 0.091 d−1
F10A1, Q99L47
n = 1
k = 0.173 d−1
RIA
Time (d)

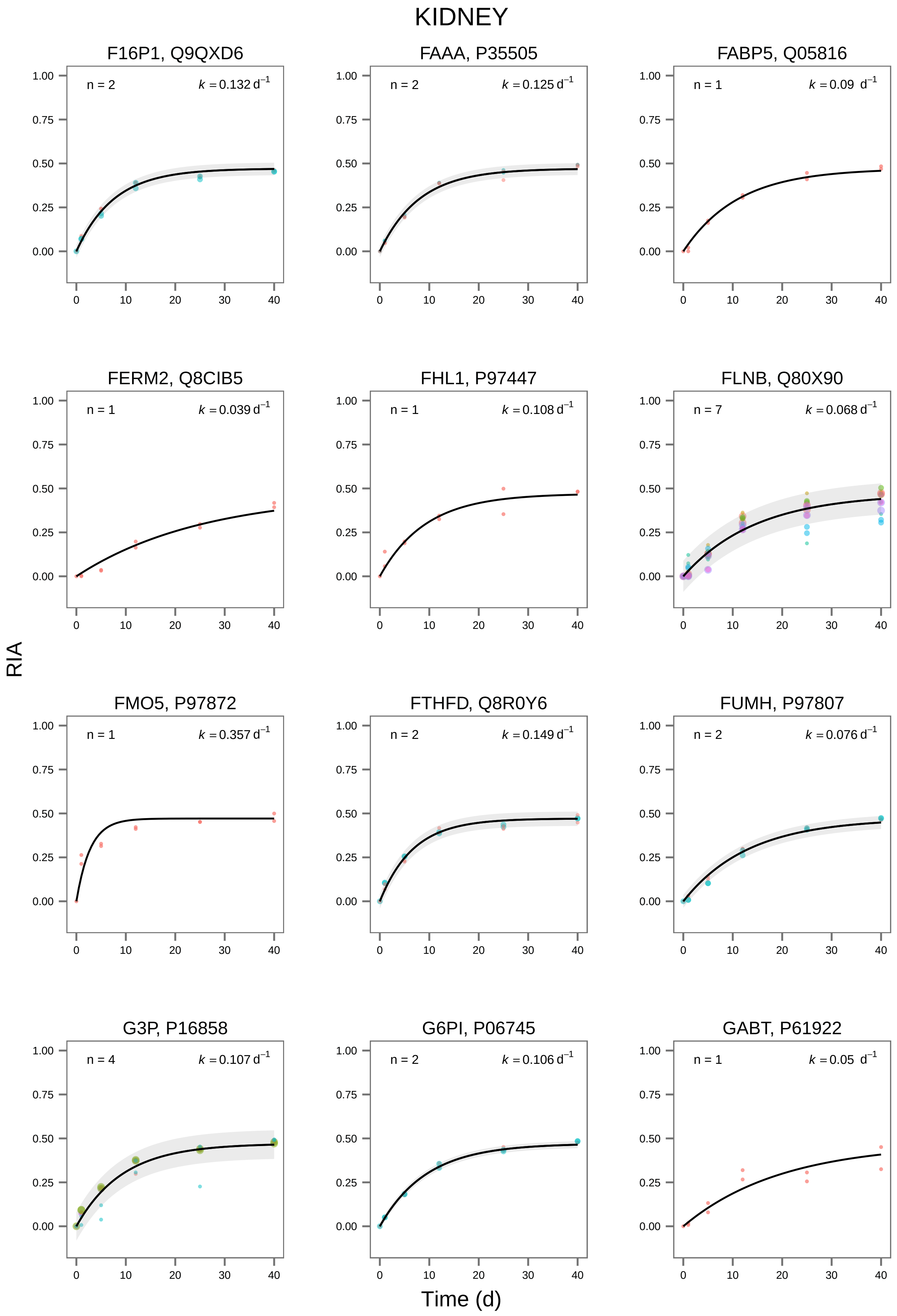
KIDNEY
F16P1, Q9QXD6
n = 2
k = 0.132 d−1
FAAA, P35505
n = 2
k = 0.125 d−1
FABP5, Q05816
n = 1
k = 0.09 d−1
FERM2, Q8CIB5
n = 1
k = 0.039 d−1
FHL1, P97447
n = 1
k = 0.108 d−1
FLNB, Q80X90
n = 7
k = 0.068 d−1
FMO5, P97872
n = 1
k = 0.357 d−1
FTHFD, Q8R0Y6
n = 2
k = 0.149 d−1
FUMH, P97807
n = 2
k = 0.076 d−1
G3P, P16858
n = 4
k = 0.107 d−1
G6PI, P06745
n = 2
k = 0.106 d−1
GABT, P61922
n = 1
k = 0.05 d−1
RIA
Time (d)
0.00
0.25
0.50
0.75
1.00
0
10
20
30
40

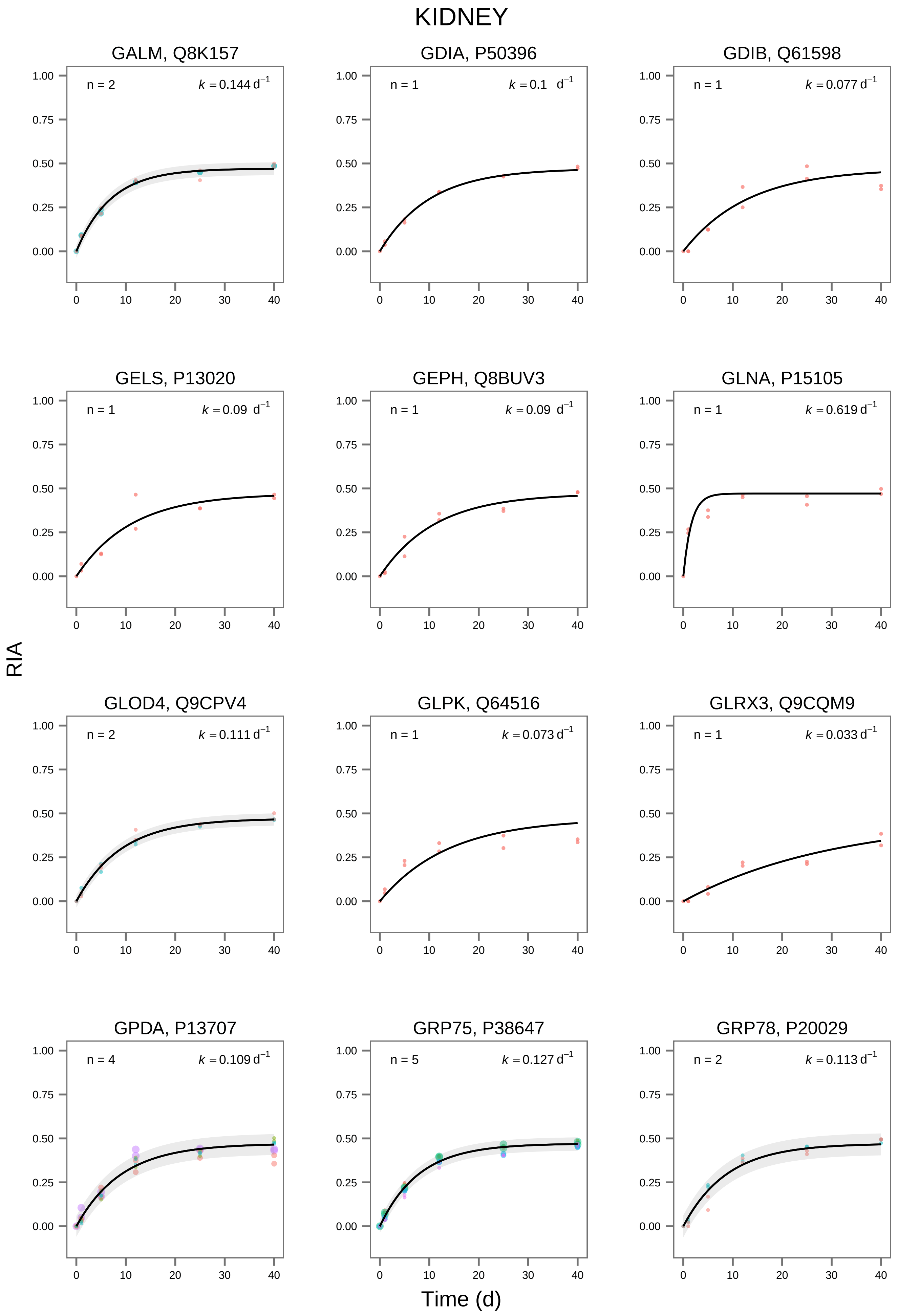
KIDNEY
GALM, Q8K157
n = 2
k = 0.144 d−1
GDIA, P50396
n = 1
k = 0.1 d−1
GDIB, Q61598
n = 1
k = 0.077 d−1
GELS, P13020
n = 1
k = 0.09 d−1
GEPH, Q8BUV3
n = 1
k = 0.09 d−1
GLNA, P15105
n = 1
k = 0.619 d−1
GLOD4, Q9CPV4
n = 2
k = 0.111 d−1
GLPK, Q64516
n = 1
k = 0.073 d−1
GLRX3, Q9CQM9
n = 1
k = 0.033 d−1
GPDA, P13707
n = 4
k = 0.109 d−1
GRP75, P38647
n = 5
k = 0.127 d−1
GRP78, P20029
n = 2
k = 0.113 d−1
RIA
Time (d)

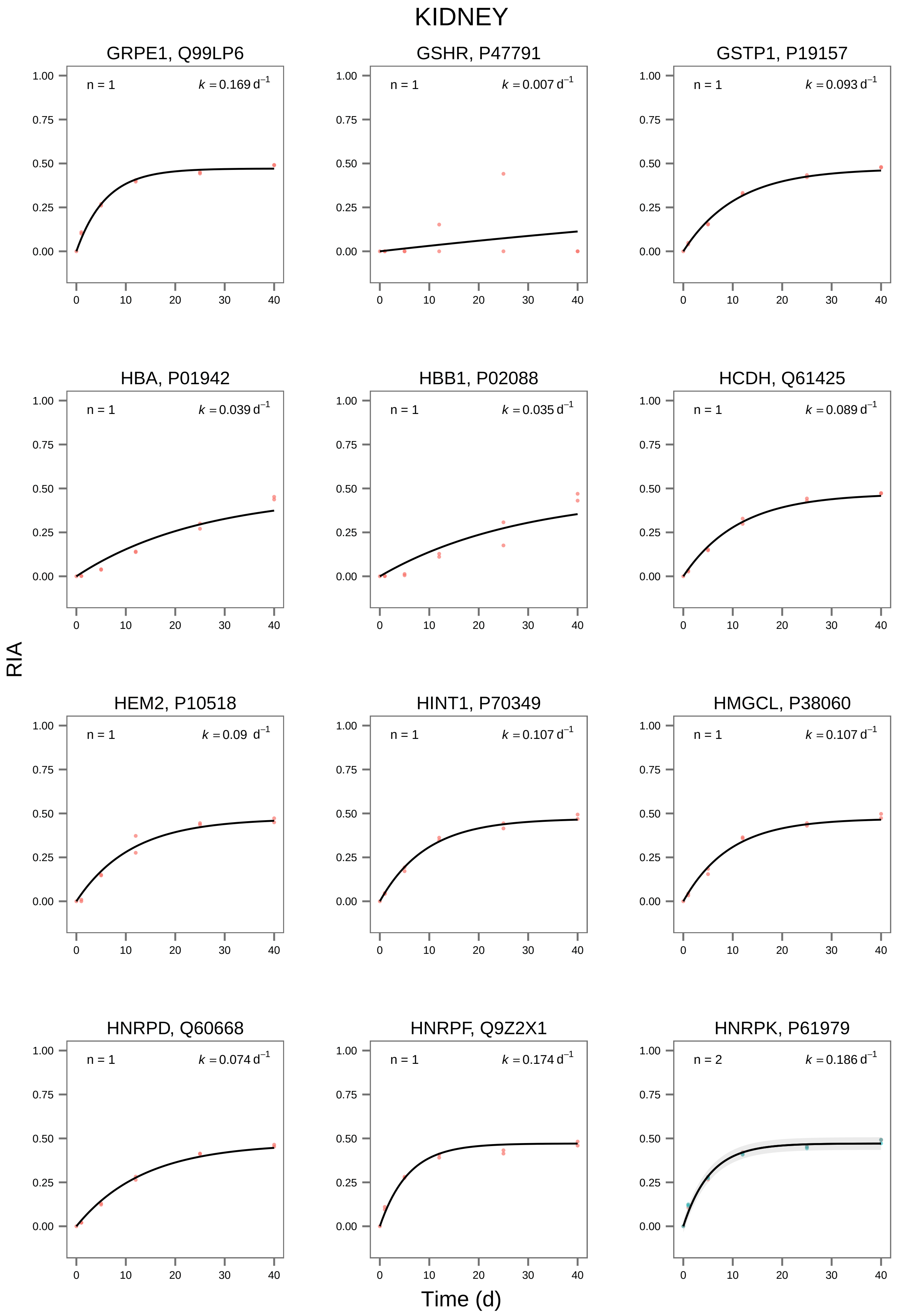
KIDNEY
GRPE1, Q99LP6
n = 1
k = 0.169 d−1
GSHR, P47791
n = 1
k = 0.007 d−1
GSTP1, P19157
n = 1
k = 0.093 d−1
HBA, P01942
n = 1
k = 0.039 d−1
HBB1, P02088
n = 1
k = 0.035 d−1
HCDH, Q61425
n = 1
k = 0.089 d−1
HEM2, P10518
n = 1
k = 0.09 d−1
HINT1, P70349
n = 1
k = 0.107 d−1
HMGCL, P38060
n = 1
k = 0.107 d−1
HNRPD, Q60668
n = 1
k = 0.074 d−1
HNRPF, Q9Z2X1
n = 1
k = 0.174 d−1
HNRPK, P61979
n = 2
k = 0.186 d−1
1.00
0.75
0.50
0.25
0.00
0
10
20
30
40
RIA
Time (d)

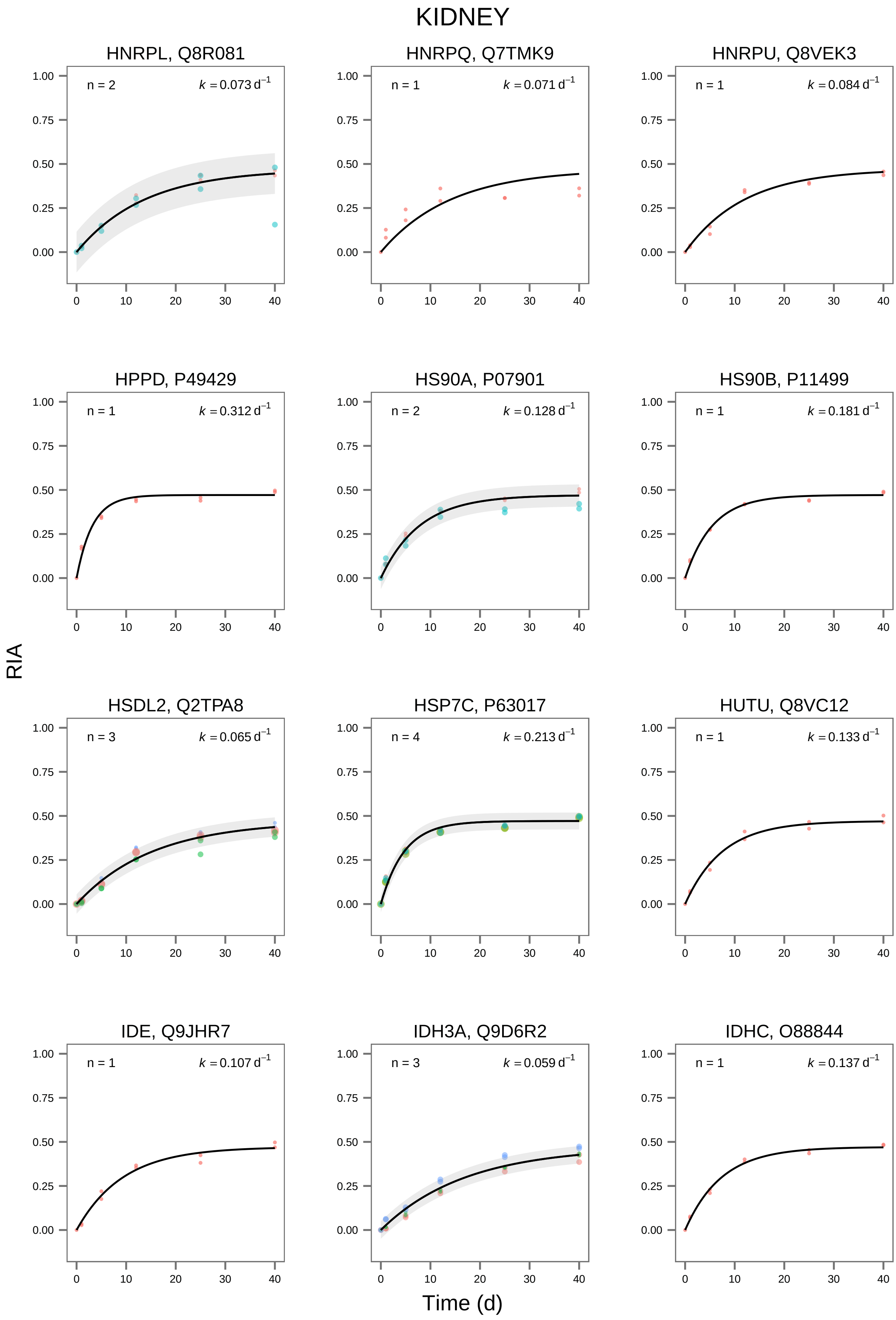
KIDNEY
HNRPL, Q8R081
n = 2
k = 0.073 d−1
HNRPQ, Q7TMK9
n = 1
k = 0.071 d−1
HNRPU, Q8VEK3
n = 1
k = 0.084 d−1
HPPD, P49429
n = 1
k = 0.312 d−1
HS90A, P07901
n = 2
k = 0.128 d−1
HS90B, P11499
n = 1
k = 0.181 d−1
HSDL2, Q2TPA8
n = 3
k = 0.065 d−1
HSP7C, P63017
n = 4
k = 0.213 d−1
HUTU, Q8VC12
n = 1
k = 0.133 d−1
IDE, Q9JHR7
n = 1
k = 0.107 d−1
IDH3A, Q9D6R2
n = 3
k = 0.059 d−1
IDHC, O88844
n = 1
k = 0.137 d−1
RIA
Time (d)

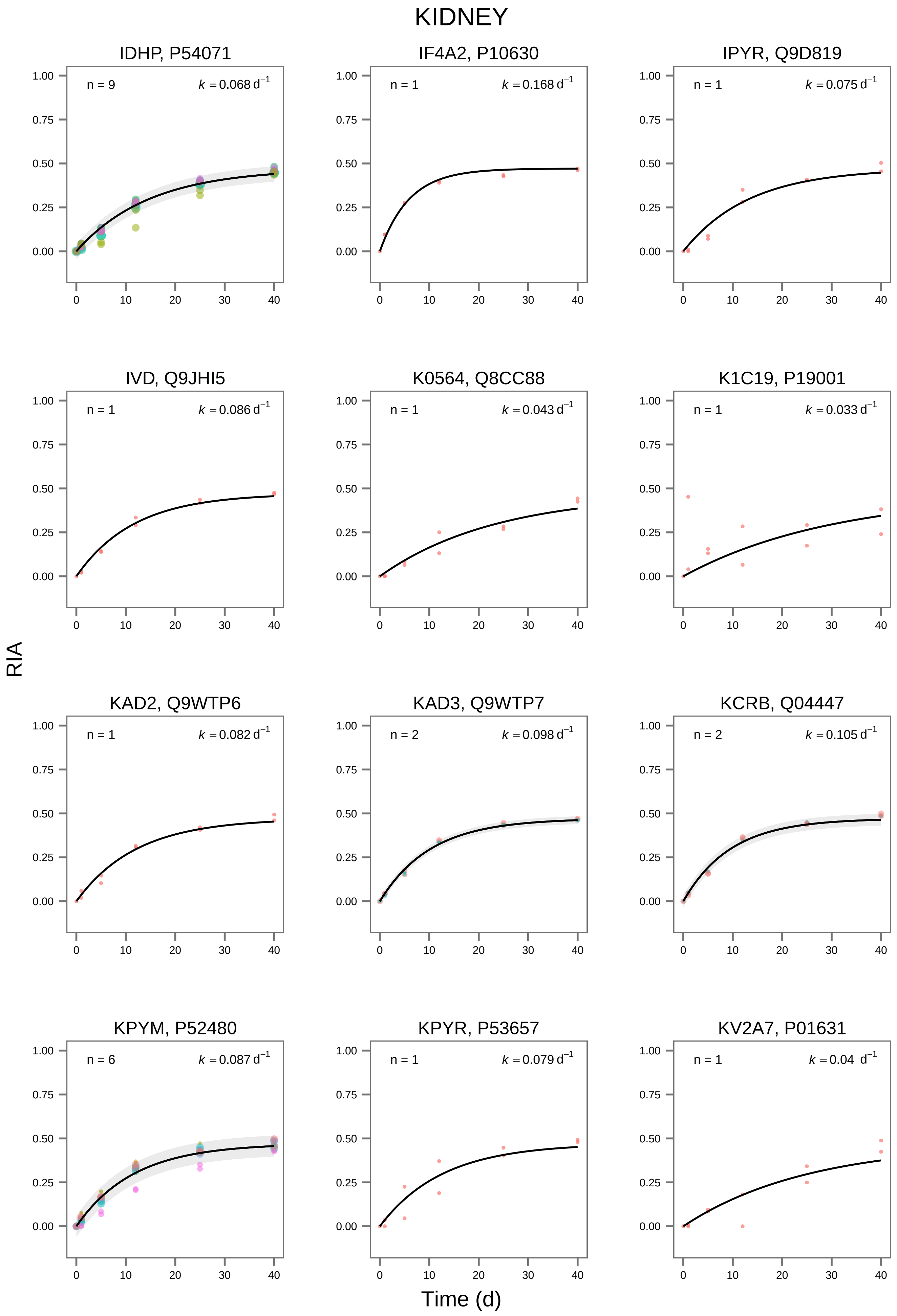
KIDNEY
IDHP, P54071
n = 9
k = 0.068 d−1
IF4A2, P10630
n = 1
k = 0.168 d−1
IPYR, Q9D819
n = 1
k = 0.075 d−1
IVD, Q9JHI5
n = 1
k = 0.086 d−1
K0564, Q8CC88
n = 1
k = 0.043 d−1
K1C19, P19001
n = 1
k = 0.033 d−1
KAD2, Q9WTP6
n = 1
k = 0.082 d−1
KAD3, Q9WTP7
n = 2
k = 0.098 d−1
KCRB, Q04447
n = 2
k = 0.105 d−1
KPYM, P52480
n = 6
k = 0.087 d−1
KPYR, P53657
n = 1
k = 0.079 d−1
KV2A7, P01631
n = 1
k = 0.04 d−1
RIA
Time (d)

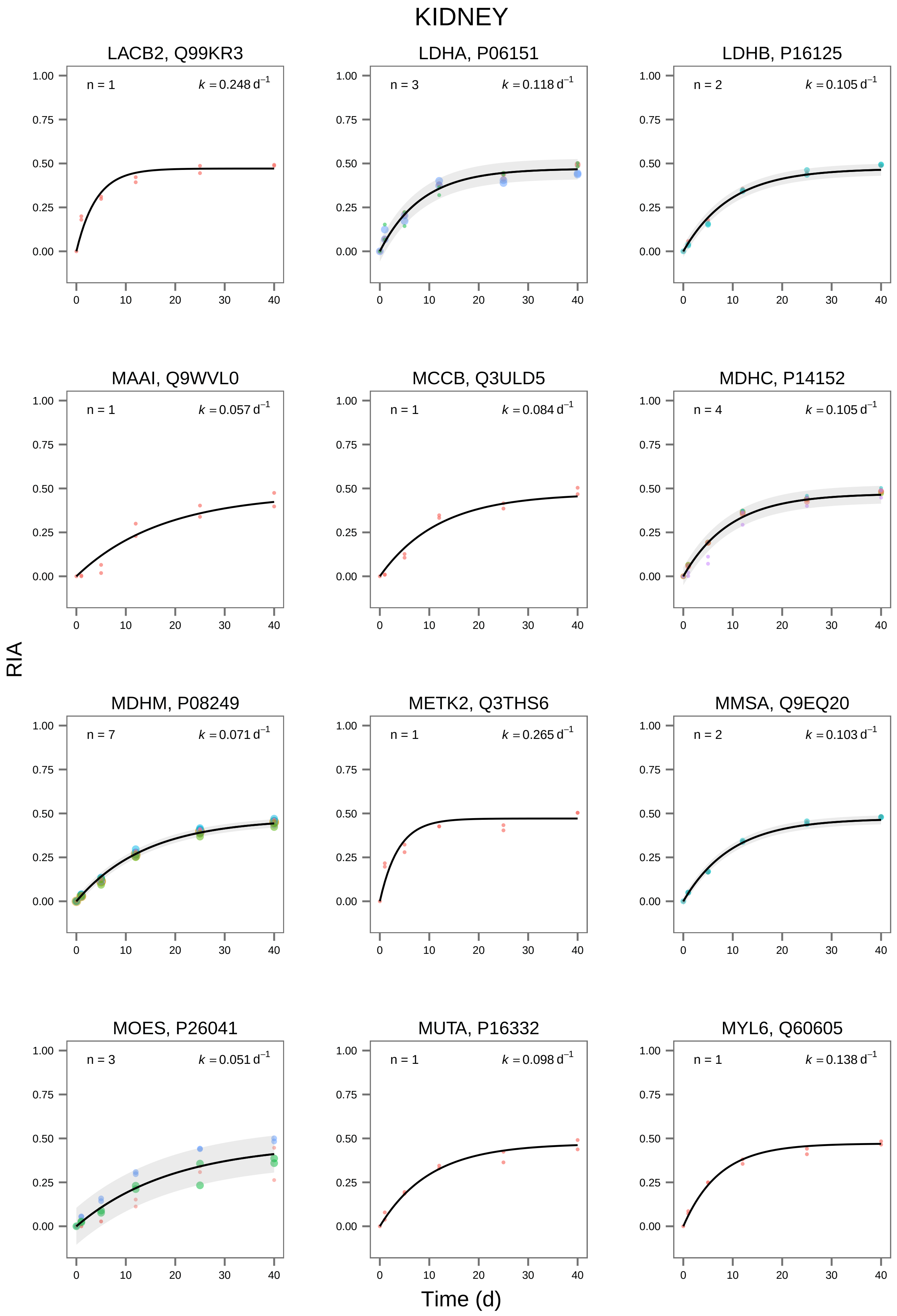
KIDNEY
LACB2, Q99KR3
n = 1
k = 0.248 d^{-1}
LDHA, P06151
n = 3
k = 0.118 d^{-1}
LDHB, P16125
n = 2
k = 0.105 d^{-1}
MAAI, Q9WVL0
n = 1
k = 0.057 d^{-1}
MCCB, Q3ULD5
n = 1
k = 0.084 d^{-1}
MDHC, P14152
n = 4
k = 0.105 d^{-1}
MDHM, P08249
n = 7
k = 0.071 d^{-1}
METK2, Q3THS6
n = 1
k = 0.265 d^{-1}
MMSA, Q9EQ20
n = 2
k = 0.103 d^{-1}
MOES, P26041
n = 3
k = 0.051 d^{-1}
MUTA, P16332
n = 1
k = 0.098 d^{-1}
MYL6, Q60605
n = 1
k = 0.138 d^{-1}
RIA
Time (d)

KIDNEY

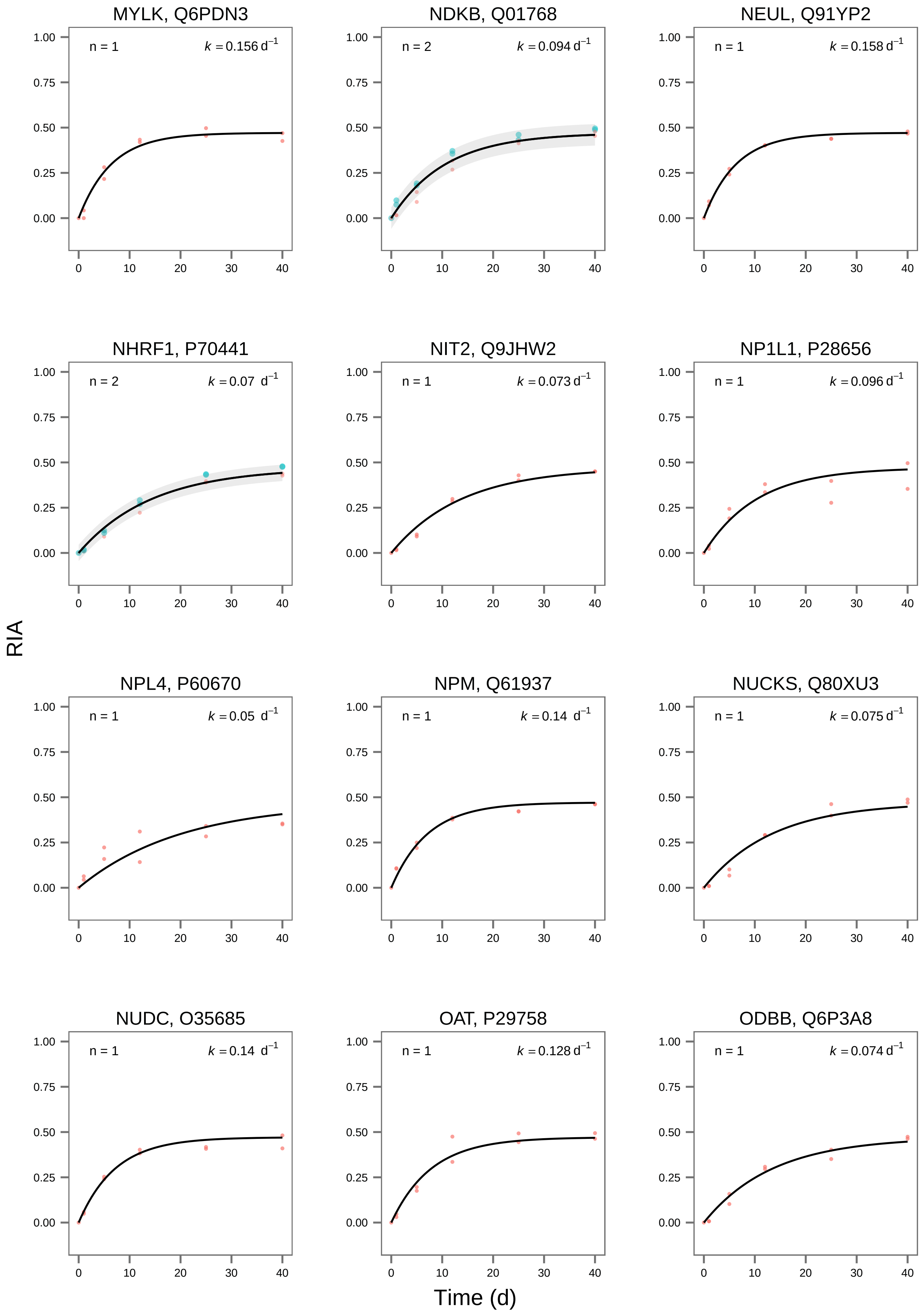
MYLK, Q6PDN3
n = 1
k = 0.156 d^{-1}
NDKB, Q01768
n = 2
k = 0.094 d^{-1}
NEUL, Q91YP2
n = 1
k = 0.158 d^{-1}
NHRF1, P70441
n = 2
k = 0.07 d^{-1}
NIT2, Q9JHW2
n = 1
k = 0.073 d^{-1}
NP1L1, P28656
n = 1
k = 0.096 d^{-1}
NPL4, P60670
n = 1
k = 0.05 d^{-1}
NPM, Q61937
n = 1
k = 0.14 d^{-1}
NUCKS, Q80XU3
n = 1
k = 0.075 d^{-1}
NUDC, O35685
n = 1
k = 0.14 d^{-1}
OAT, P29758
n = 1
k = 0.128 d^{-1}
ODBB, Q6P3A8
n = 1
k = 0.074 d^{-1}
1.00
0.75
0.50
0.25
0.00
0
10
20
30
40
RIA
Time (d)

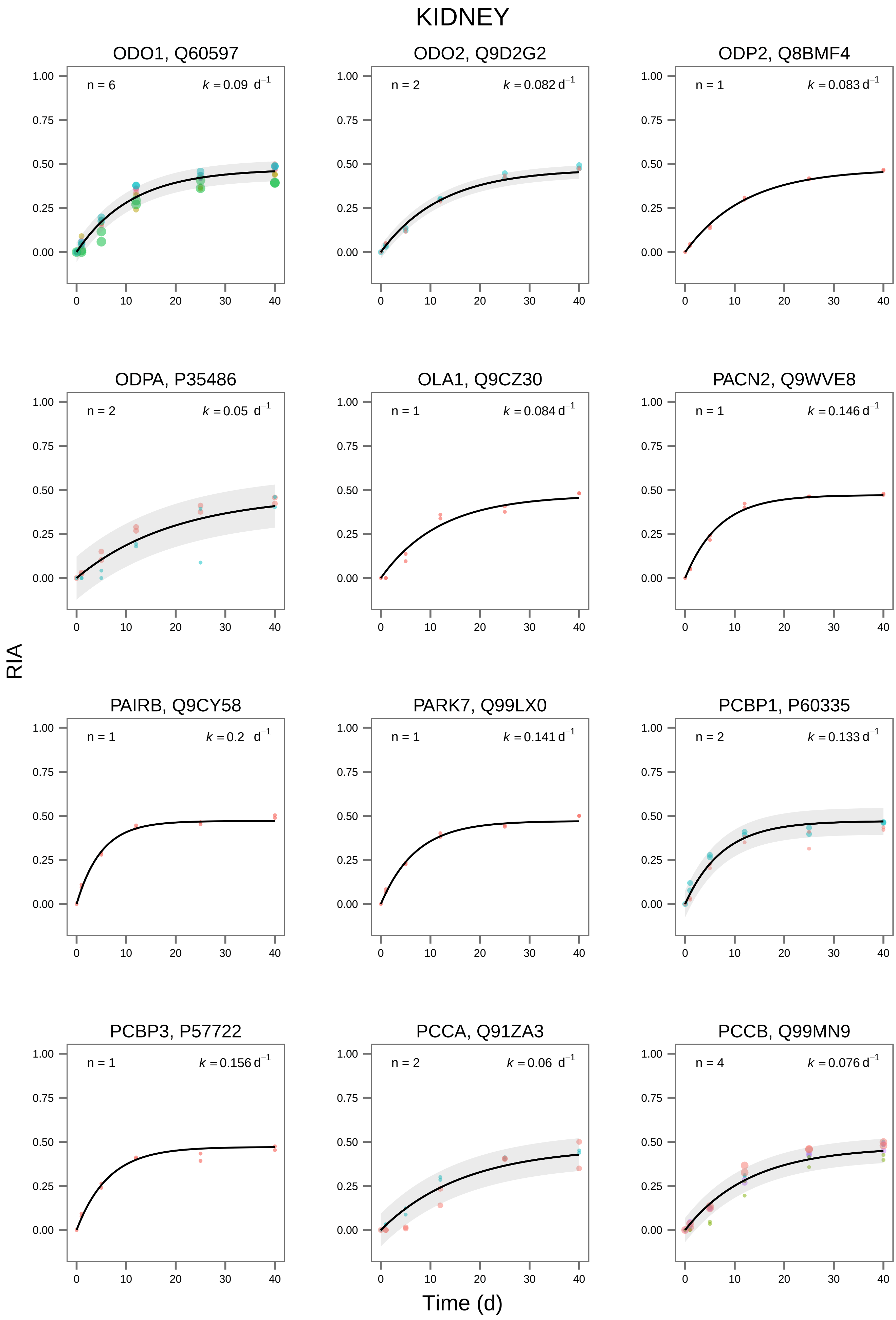
KIDNEY
ODO1, Q60597
n = 6
k = 0.09 d−1
ODO2, Q9D2G2
n = 2
k = 0.082 d−1
ODP2, Q8BMF4
n = 1
k = 0.083 d−1
ODPA, P35486
n = 2
k = 0.05 d−1
OLA1, Q9CZ30
n = 1
k = 0.084 d−1
PACN2, Q9WVE8
n = 1
k = 0.146 d−1
PAIRB, Q9CY58
n = 1
k = 0.2 d−1
PARK7, Q99LX0
n = 1
k = 0.141 d−1
PCBP1, P60335
n = 2
k = 0.133 d−1
PCBP3, P57722
n = 1
k = 0.156 d−1
PCCA, Q91ZA3
n = 2
k = 0.06 d−1
PCCB, Q99MN9
n = 4
k = 0.076 d−1
RIA
Time (d)

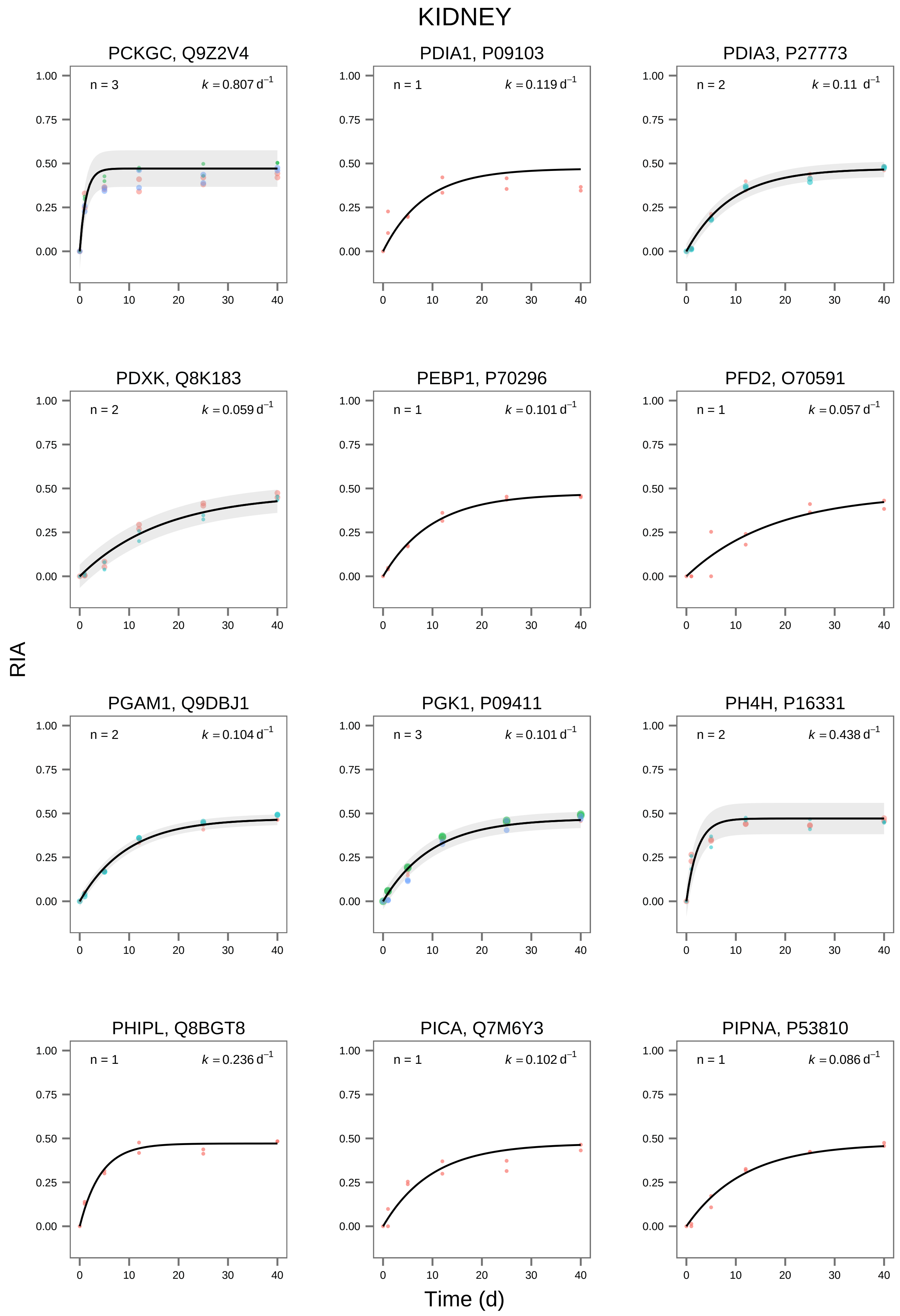
KIDNEY
PCKGC, Q9Z2V4
n = 3
k = 0.807 d^{-1}
PDIA1, P09103
n = 1
k = 0.119 d^{-1}
PDIA3, P27773
n = 2
k = 0.11 d^{-1}
PDXK, Q8K183
n = 2
k = 0.059 d^{-1}
PEBP1, P70296
n = 1
k = 0.101 d^{-1}
PFD2, O70591
n = 1
k = 0.057 d^{-1}
PGAM1, Q9DBJ1
n = 2
k = 0.104 d^{-1}
PGK1, P09411
n = 3
k = 0.101 d^{-1}
PH4H, P16331
n = 2
k = 0.438 d^{-1}
PHIPL, Q8BGT8
n = 1
k = 0.236 d^{-1}
PICA, Q7M6Y3
n = 1
k = 0.102 d^{-1}
PIPNA, P53810
n = 1
k = 0.086 d^{-1}
RIA
Time (d)
0.00
0.25
0.50
0.75
1.00
0
10
20
30
40

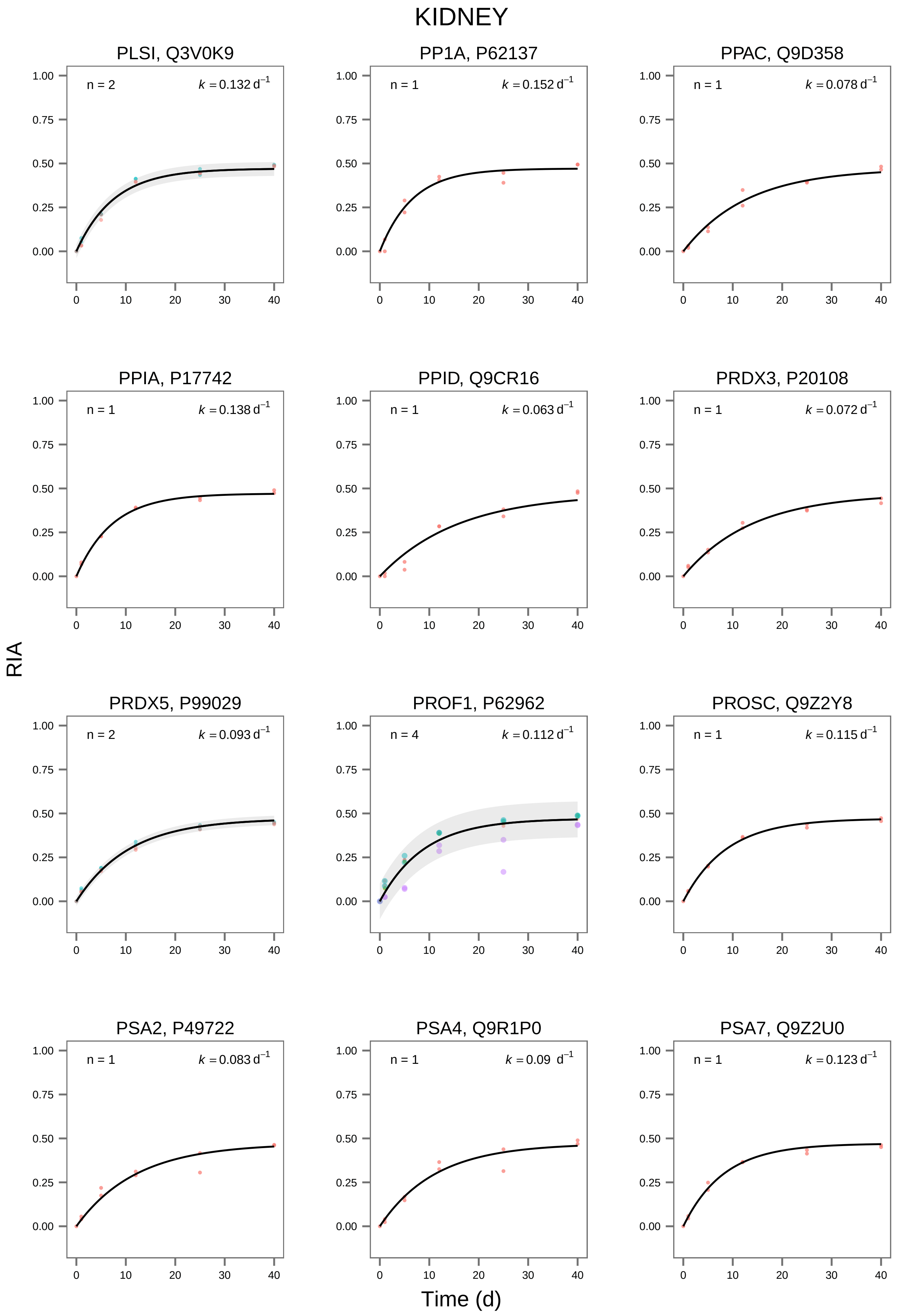
KIDNEY
PLSI, Q3V0K9
n = 2
k = 0.132 d−1
PP1A, P62137
n = 1
k = 0.152 d−1
PPAC, Q9D358
n = 1
k = 0.078 d−1
PPIA, P17742
n = 1
k = 0.138 d−1
PPID, Q9CR16
n = 1
k = 0.063 d−1
PRDX3, P20108
n = 1
k = 0.072 d−1
PRDX5, P99029
n = 2
k = 0.093 d−1
PROF1, P62962
n = 4
k = 0.112 d−1
PROSC, Q9Z2Y8
n = 1
k = 0.115 d−1
PSA2, P49722
n = 1
k = 0.083 d−1
PSA4, Q9R1P0
n = 1
k = 0.09 d−1
PSA7, Q9Z2U0
n = 1
k = 0.123 d−1
1.00
0.75
0.50
0.25
0.00
0
10
20
30
40
RIA
Time (d)

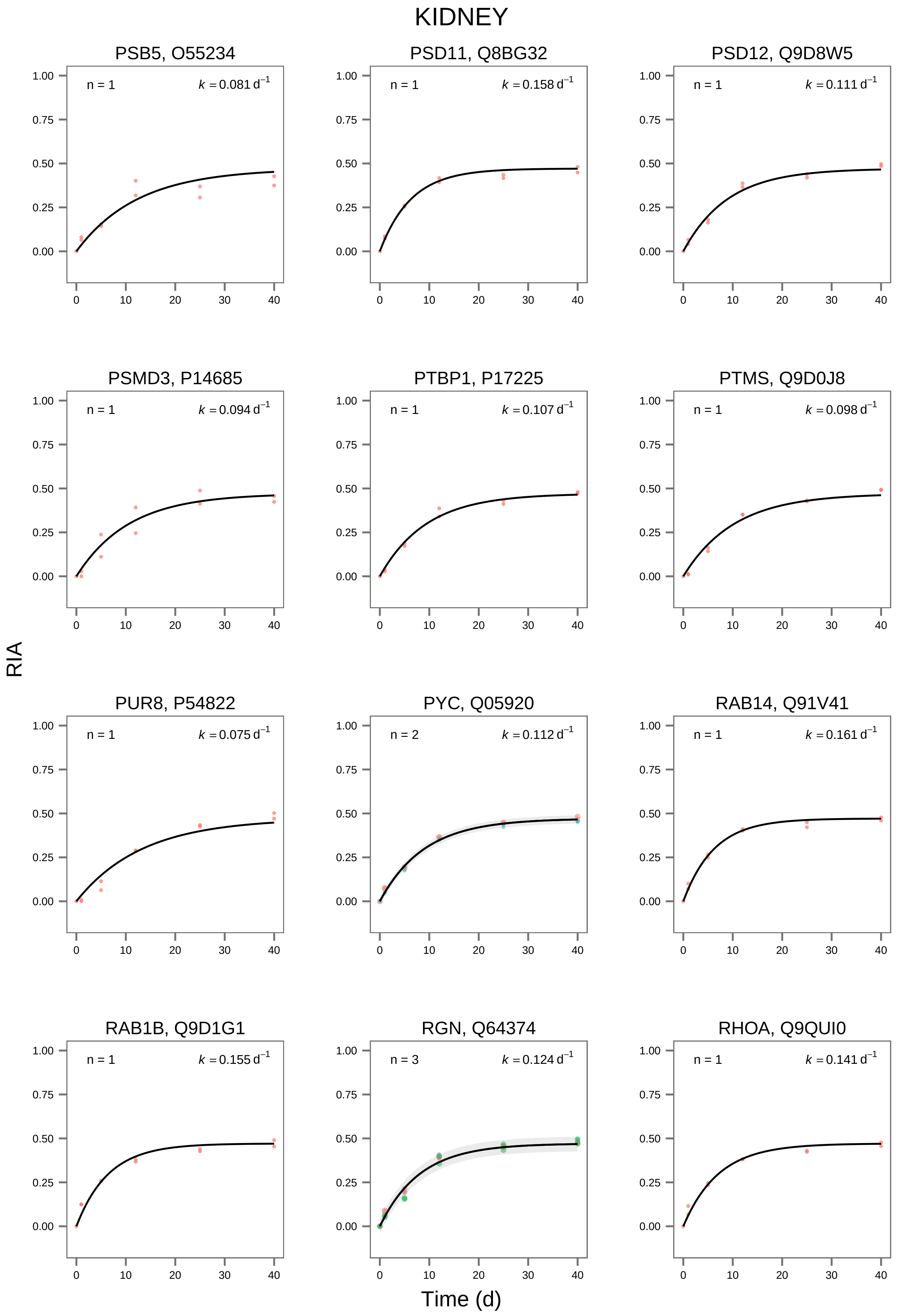
KIDNEY
PSB5, O55234
n = 1
k = 0.081 d−1
PSD11, Q8BG32
n = 1
k = 0.158 d−1
PSD12, Q9D8W5
n = 1
k = 0.111 d−1
PSMD3, P14685
n = 1
k = 0.094 d−1
PTBP1, P17225
n = 1
k = 0.107 d−1
PTMS, Q9D0J8
n = 1
k = 0.098 d−1
PUR8, P54822
n = 1
k = 0.075 d−1
PYC, Q05920
n = 2
k = 0.112 d−1
RAB14, Q91V41
n = 1
k = 0.161 d−1
RAB1B, Q9D1G1
n = 1
k = 0.155 d−1
RGN, Q64374
n = 3
k = 0.124 d−1
RHOA, Q9QUI0
n = 1
k = 0.141 d−1
RIA
Time (d)
0.00
0.25
0.50
0.75
1.00
0
10
20
30
40

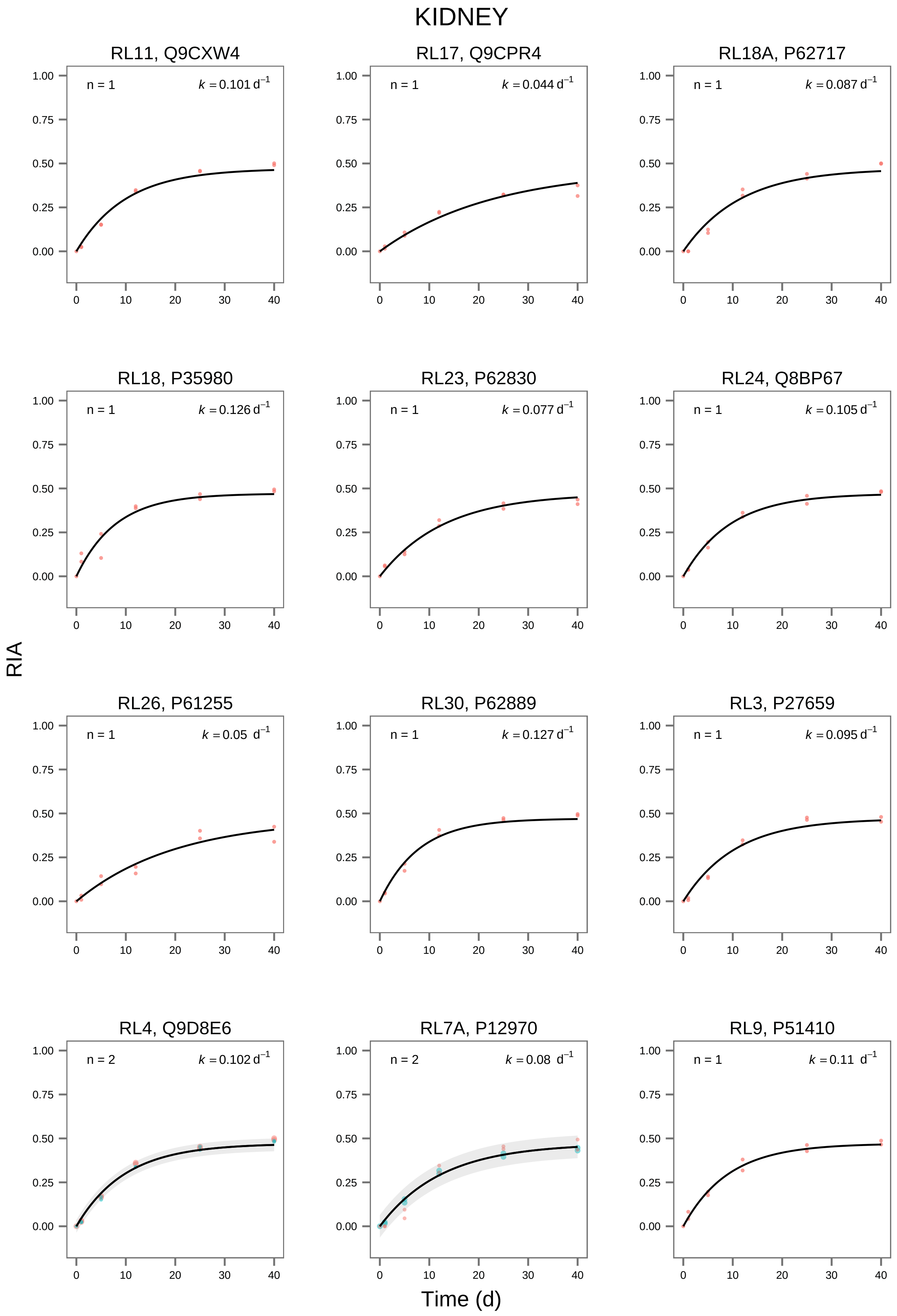
KIDNEY
RL11, Q9CXW4
n = 1
k = 0.101 d−1
RL17, Q9CPR4
n = 1
k = 0.044 d−1
RL18A, P62717
n = 1
k = 0.087 d−1
RL18, P35980
n = 1
k = 0.126 d−1
RL23, P62830
n = 1
k = 0.077 d−1
RL24, Q8BP67
n = 1
k = 0.105 d−1
RL26, P61255
n = 1
k = 0.05 d−1
RL30, P62889
n = 1
k = 0.127 d−1
RL3, P27659
n = 1
k = 0.095 d−1
RL4, Q9D8E6
n = 2
k = 0.102 d−1
RL7A, P12970
n = 2
k = 0.08 d−1
RL9, P51410
n = 1
k = 0.11 d−1
RIA
Time (d)

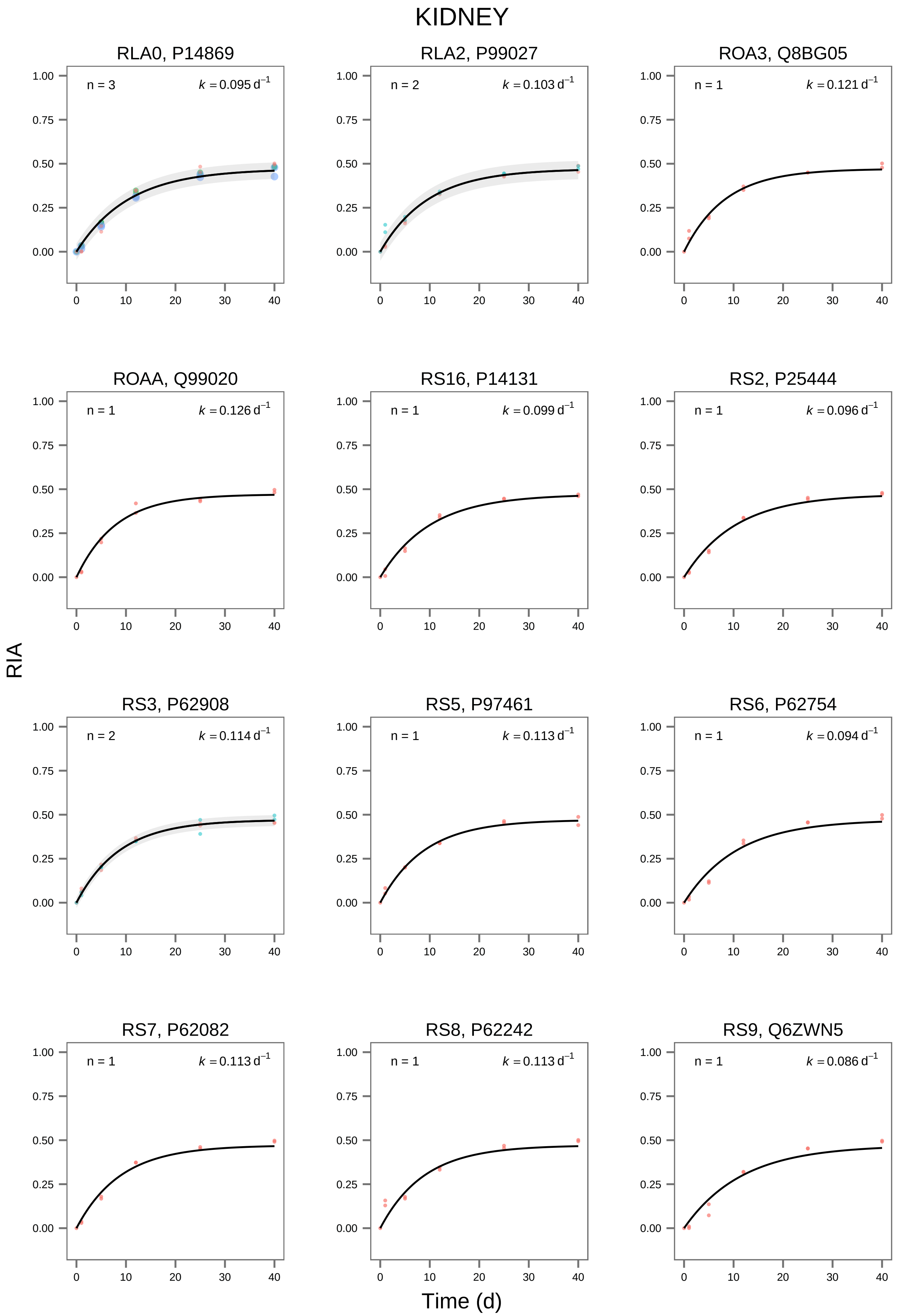
KIDNEY
RLA0, P14869
n = 3
k = 0.095 d−1
RLA2, P99027
n = 2
k = 0.103 d−1
ROA3, Q8BG05
n = 1
k = 0.121 d−1
ROAA, Q99020
n = 1
k = 0.126 d−1
RS16, P14131
n = 1
k = 0.099 d−1
RS2, P25444
n = 1
k = 0.096 d−1
RS3, P62908
n = 2
k = 0.114 d−1
RS5, P97461
n = 1
k = 0.113 d−1
RS6, P62754
n = 1
k = 0.094 d−1
RS7, P62082
n = 1
k = 0.113 d−1
RS8, P62242
n = 1
k = 0.113 d−1
RS9, Q6ZWN5
n = 1
k = 0.086 d−1
RIA
Time (d)
1.00
0.75
0.50
0.25
0.00
0
10
20
30
40

KIDNEY

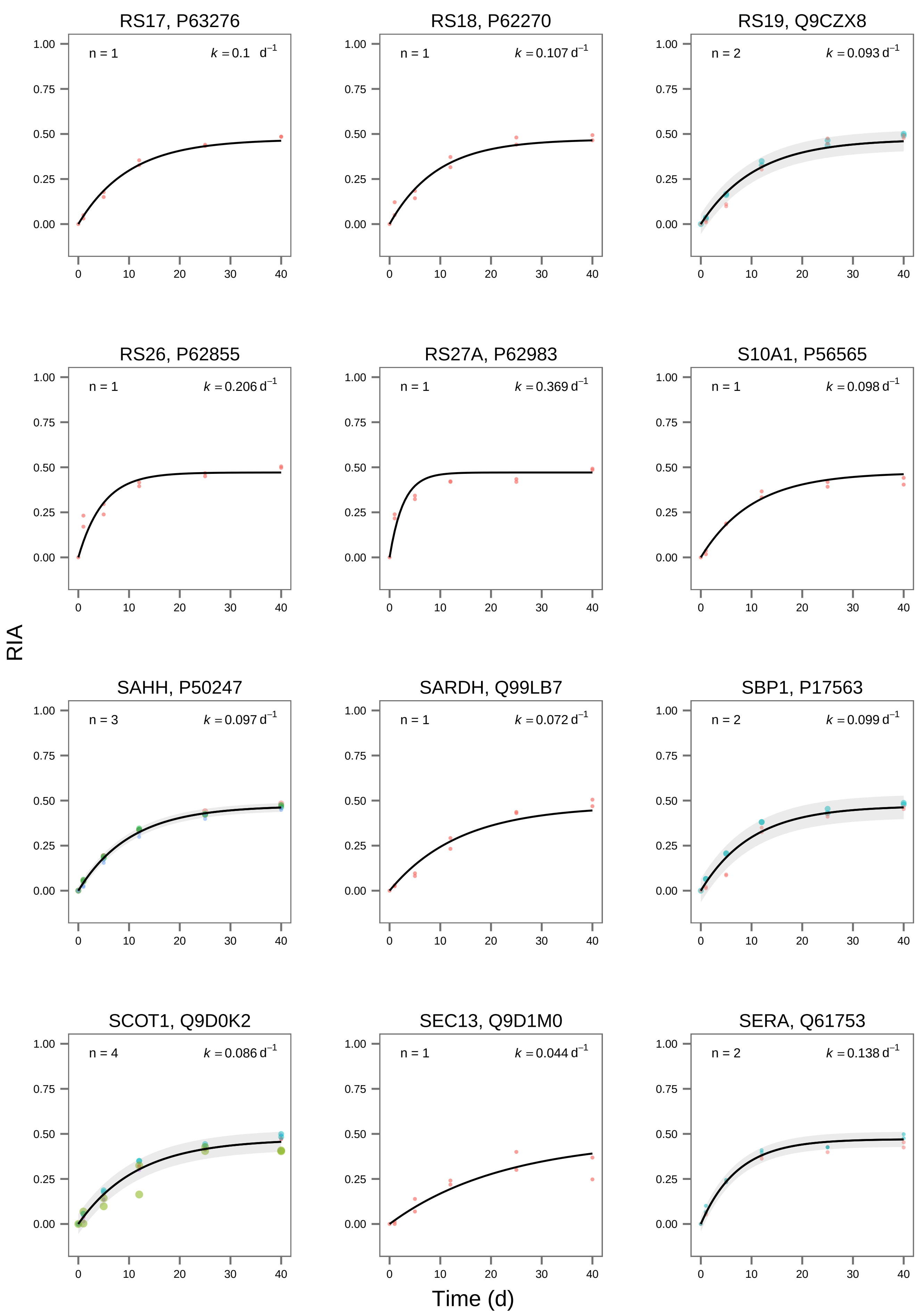

RS17, P63276
n = 1
k = 0.1 d⁻¹
RS18, P62270
n = 1
k = 0.107 d⁻¹
RS19, Q9CZX8
n = 2
k = 0.093 d⁻¹
RS26, P62855
n = 1
k = 0.206 d⁻¹
RS27A, P62983
n = 1
k = 0.369 d⁻¹
S10A1, P56565
n = 1
k = 0.098 d⁻¹
SAHH, P50247
n = 3
k = 0.097 d⁻¹
SARDH, Q99LB7
n = 1
k = 0.072 d⁻¹
SBP1, P17563
n = 2
k = 0.099 d⁻¹
SCOT1, Q9D0K2
n = 4
k = 0.086 d⁻¹
SEC13, Q9D1M0
n = 1
k = 0.044 d⁻¹
SERA, Q61753
n = 2
k = 0.138 d⁻¹
RIA
Time (d)
0.00
0.25
0.50
0.75
1.00
0
10
20
30
40

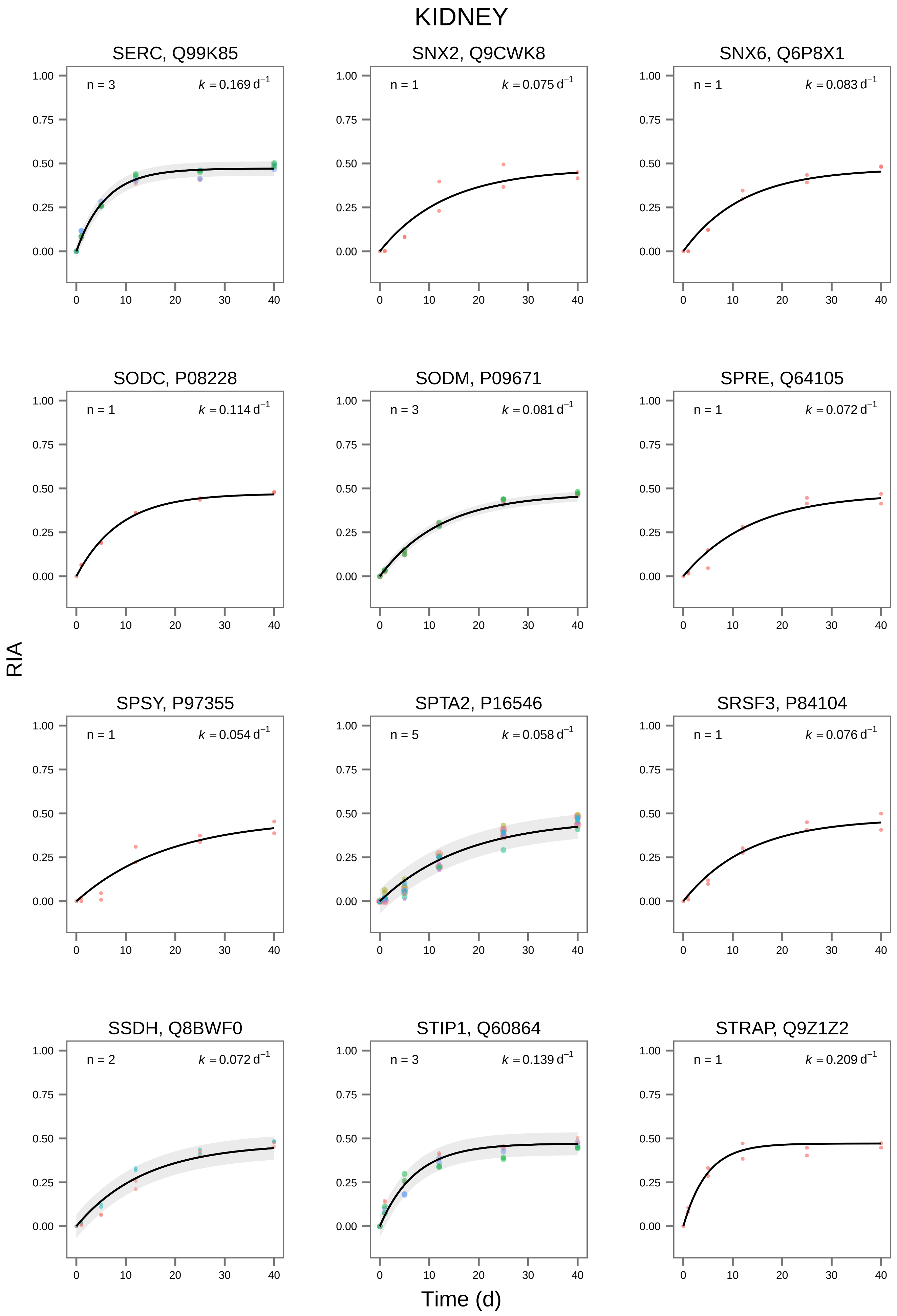
KIDNEY
SERC, Q99K85
n = 3
k = 0.169 d−1
SNX2, Q9CWK8
n = 1
k = 0.075 d−1
SNX6, Q6P8X1
n = 1
k = 0.083 d−1
SODC, P08228
n = 1
k = 0.114 d−1
SODM, P09671
n = 3
k = 0.081 d−1
SPRE, Q64105
n = 1
k = 0.072 d−1
SPSY, P97355
n = 1
k = 0.054 d−1
SPTA2, P16546
n = 5
k = 0.058 d−1
SRSF3, P84104
n = 1
k = 0.076 d−1
SSDH, Q8BWF0
n = 2
k = 0.072 d−1
STIP1, Q60864
n = 3
k = 0.139 d−1
STRAP, Q9Z1Z2
n = 1
k = 0.209 d−1
RIA
Time (d)

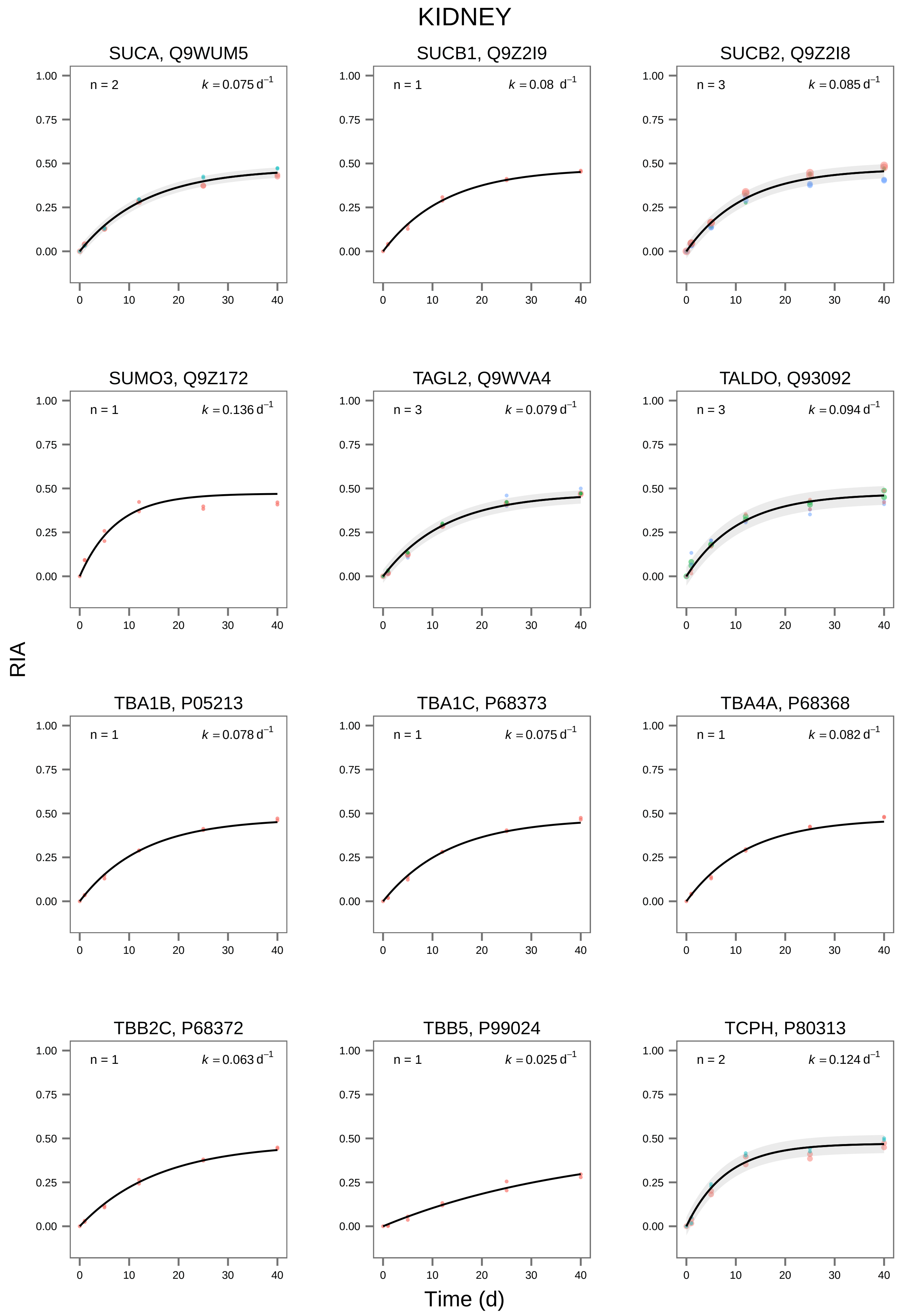
KIDNEY
SUCA, Q9WUM5
n = 2
k = 0.075 d⁻¹
SUCB1, Q9Z2I9
n = 1
k = 0.08 d⁻¹
SUCB2, Q9Z2I8
n = 3
k = 0.085 d⁻¹
SUMO3, Q9Z172
n = 1
k = 0.136 d⁻¹
TAGL2, Q9WVA4
n = 3
k = 0.079 d⁻¹
TALDO, Q93092
n = 3
k = 0.094 d⁻¹
TBA1B, P05213
n = 1
k = 0.078 d⁻¹
TBA1C, P68373
n = 1
k = 0.075 d⁻¹
TBA4A, P68368
n = 1
k = 0.082 d⁻¹
TBB2C, P68372
n = 1
k = 0.063 d⁻¹
TBB5, P99024
n = 1
k = 0.025 d⁻¹
TCPH, P80313
n = 2
k = 0.124 d⁻¹
RIA
Time (d)

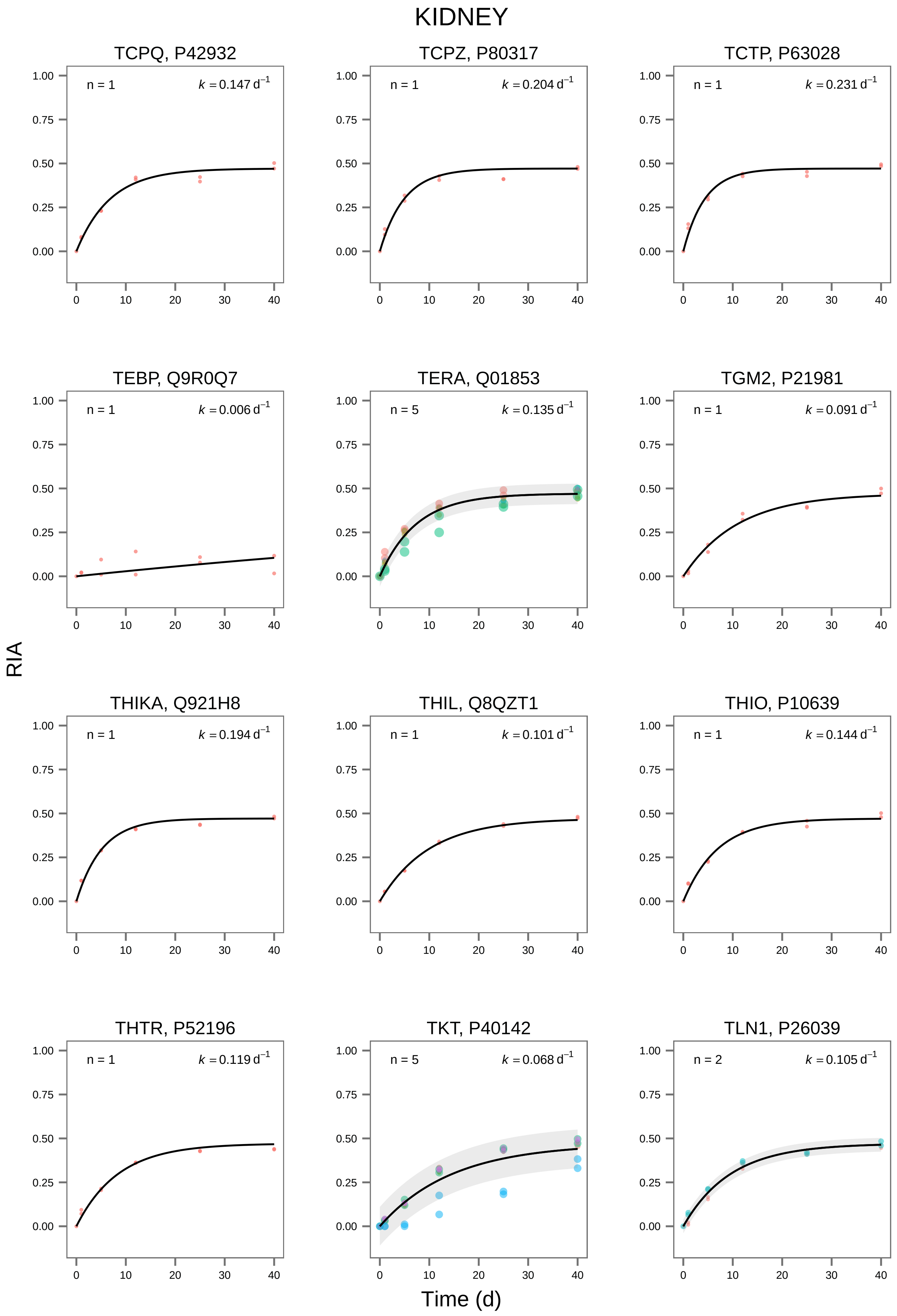
KIDNEY
TCPQ, P42932
n = 1
k = 0.147 d^{-1}
TCPZ, P80317
n = 1
k = 0.204 d^{-1}
TCTP, P63028
n = 1
k = 0.231 d^{-1}
TEBP, Q9R0Q7
n = 1
k = 0.006 d^{-1}
TERA, Q01853
n = 5
k = 0.135 d^{-1}
TGM2, P21981
n = 1
k = 0.091 d^{-1}
THIKA, Q921H8
n = 1
k = 0.194 d^{-1}
THIL, Q8QZT1
n = 1
k = 0.101 d^{-1}
THIO, P10639
n = 1
k = 0.144 d^{-1}
THTR, P52196
n = 1
k = 0.119 d^{-1}
TKT, P40142
n = 5
k = 0.068 d^{-1}
TLN1, P26039
n = 2
k = 0.105 d^{-1}
RIA
Time (d)

KIDNEY

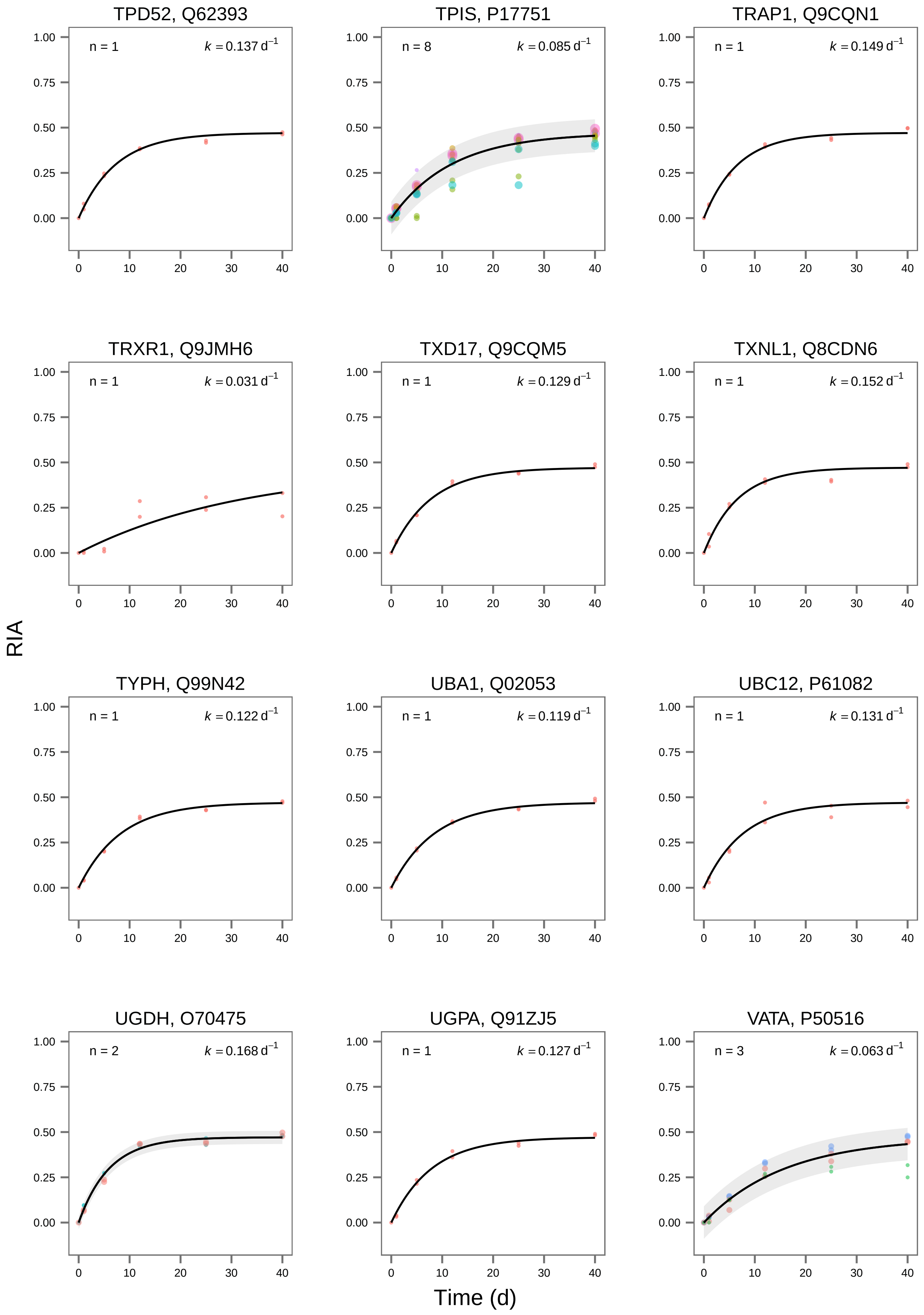
TPD52, Q62393
n = 1
k = 0.137 d−1
TPIS, P17751
n = 8
k = 0.085 d−1
TRAP1, Q9CQN1
n = 1
k = 0.149 d−1
TRXR1, Q9JMH6
n = 1
k = 0.031 d−1
TXD17, Q9CQM5
n = 1
k = 0.129 d−1
TXNL1, Q8CDN6
n = 1
k = 0.152 d−1
TYPH, Q99N42
n = 1
k = 0.122 d−1
UBA1, Q02053
n = 1
k = 0.119 d−1
UBC12, P61082
n = 1
k = 0.131 d−1
UGDH, O70475
n = 2
k = 0.168 d−1
UGPA, Q91ZJ5
n = 1
k = 0.127 d−1
VATA, P50516
n = 3
k = 0.063 d−1
1.00
0.75
0.50
0.25
0.00
0
10
20
30
40
RIA
Time (d)

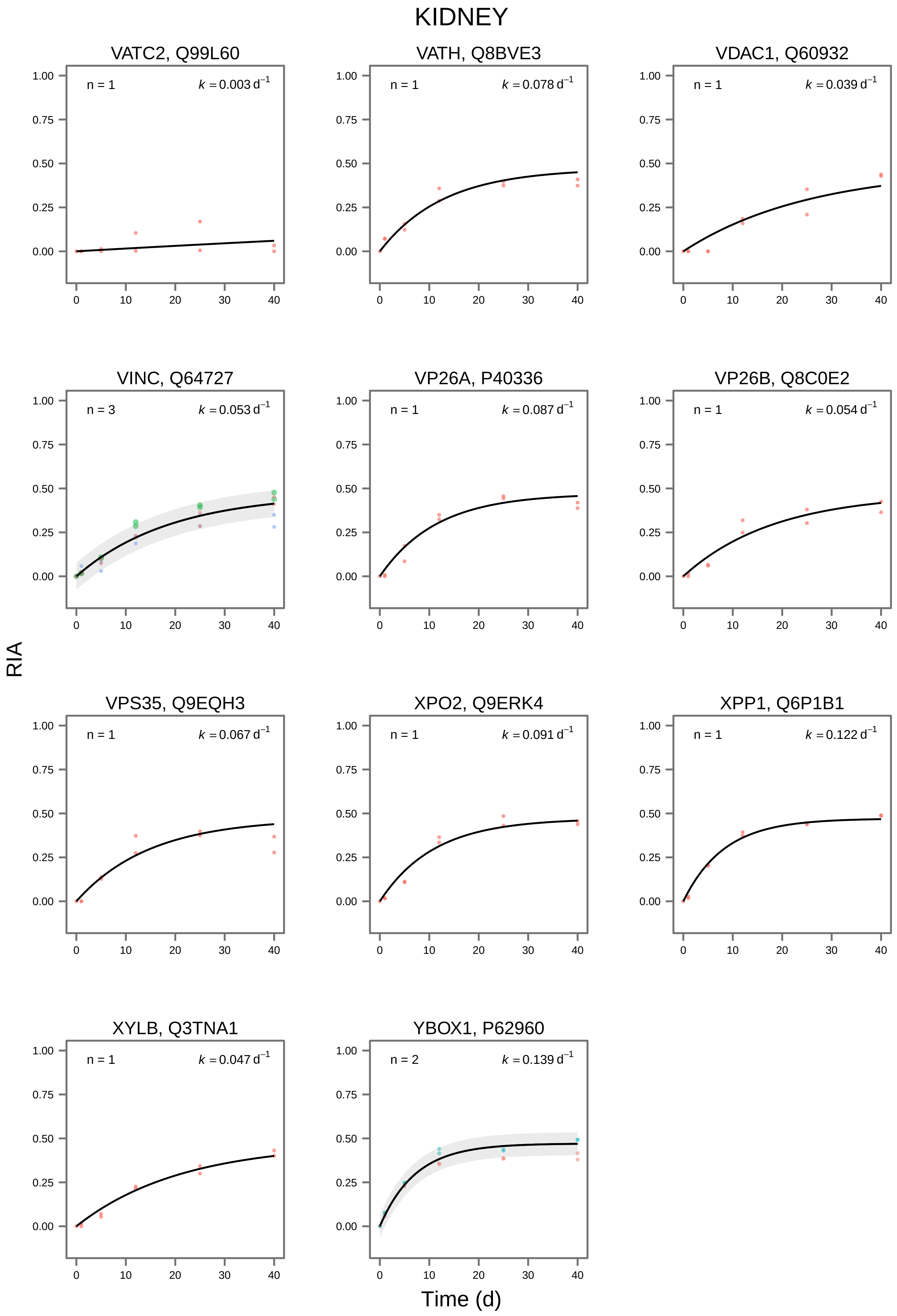
KIDNEY
VATC2, Q99L60
n = 1
k = 0.003 d−1
VATH, Q8BVE3
n = 1
k = 0.078 d−1
VDAC1, Q60932
n = 1
k = 0.039 d−1
VINC, Q64727
n = 3
k = 0.053 d−1
VP26A, P40336
n = 1
k = 0.087 d−1
VP26B, Q8C0E2
n = 1
k = 0.054 d−1
VPS35, Q9EQH3
n = 1
k = 0.067 d−1
XPO2, Q9ERK4
n = 1
k = 0.091 d−1
XPP1, Q6P1B1
n = 1
k = 0.122 d−1
XYLB, Q3TNA1
n = 1
k = 0.047 d−1
YBOX1, P62960
n = 2
k = 0.139 d−1
RIA
Time (d)
0.00
0.25
0.50
0.75
1.00
0
10
20
30
40

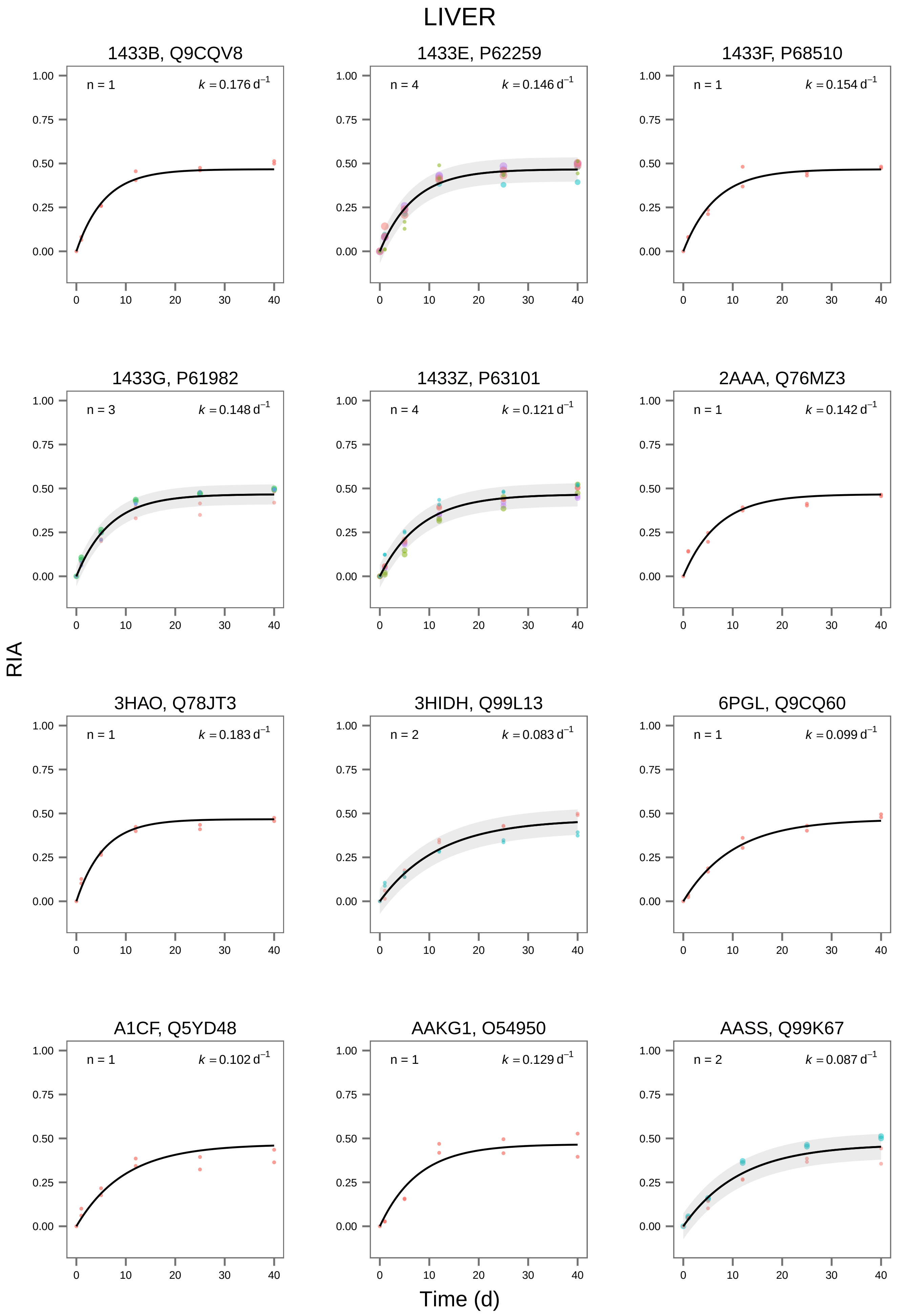
LIVER
1433B, Q9CQV8
n = 1
k = 0.176 d–1
1433E, P62259
n = 4
k = 0.146 d–1
1433F, P68510
n = 1
k = 0.154 d–1
1433G, P61982
n = 3
k = 0.148 d–1
1433Z, P63101
n = 4
k = 0.121 d–1
2AAA, Q76MZ3
n = 1
k = 0.142 d–1
3HAO, Q78JT3
n = 1
k = 0.183 d–1
3HIDH, Q99L13
n = 2
k = 0.083 d–1
6PGL, Q9CQ60
n = 1
k = 0.099 d–1
A1CF, Q5YD48
n = 1
k = 0.102 d–1
AAKG1, O54950
n = 1
k = 0.129 d–1
AASS, Q99K67
n = 2
k = 0.087 d–1
RIA
Time (d)
1.00
0.75
0.50
0.25
0.00
0
10
20
30
40

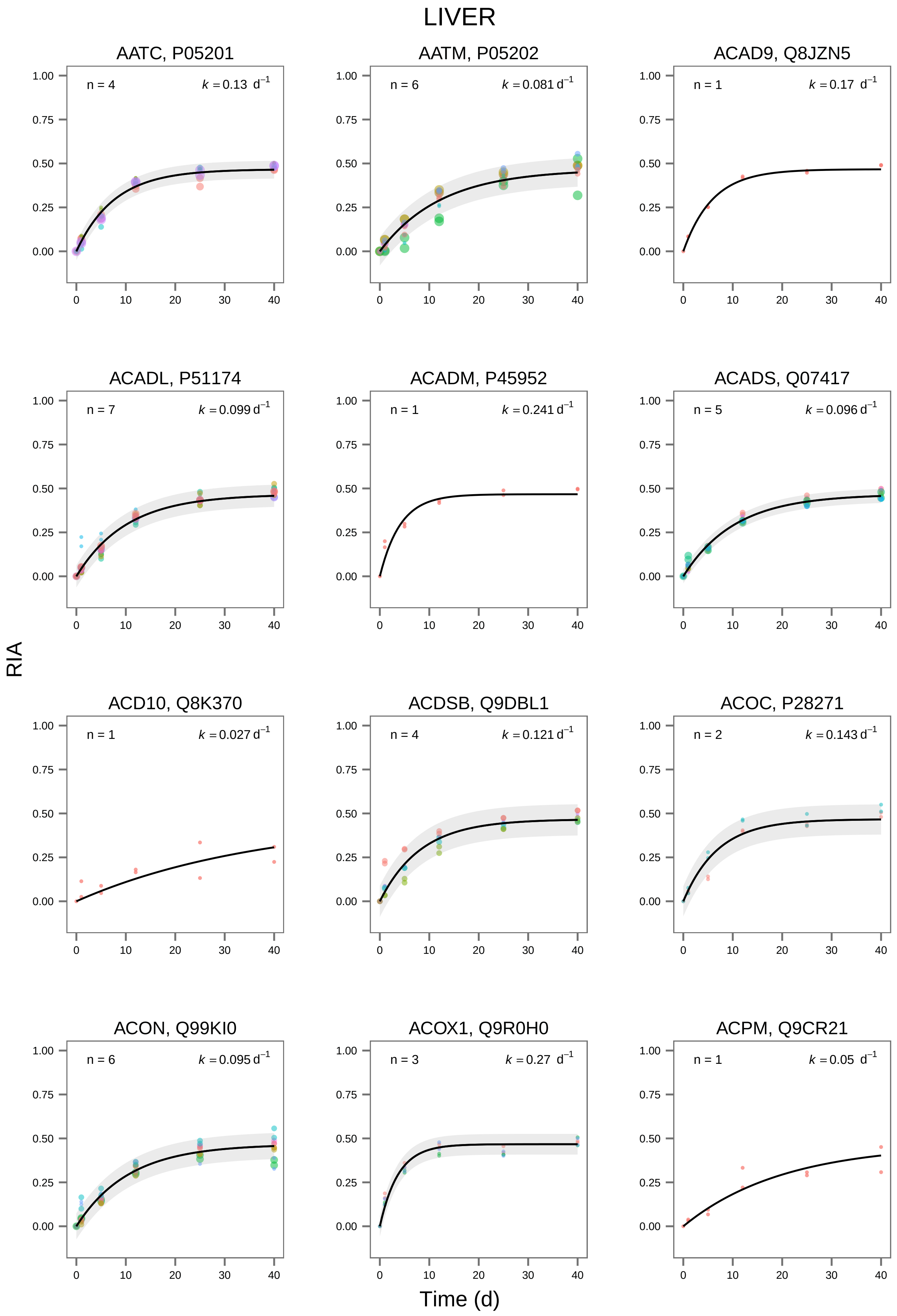
LIVER
AATC, P05201
n = 4
k = 0.13 d−1
AATM, P05202
n = 6
k = 0.081 d−1
ACAD9, Q8JZN5
n = 1
k = 0.17 d−1
ACADL, P51174
n = 7
k = 0.099 d−1
ACADM, P45952
n = 1
k = 0.241 d−1
ACADS, Q07417
n = 5
k = 0.096 d−1
ACD10, Q8K370
n = 1
k = 0.027 d−1
ACDSB, Q9DBL1
n = 4
k = 0.121 d−1
ACOC, P28271
n = 2
k = 0.143 d−1
ACON, Q99KI0
n = 6
k = 0.095 d−1
ACOX1, Q9R0H0
n = 3
k = 0.27 d−1
ACPM, Q9CR21
n = 1
k = 0.05 d−1
RIA
Time (d)
0.00
0.25
0.50
0.75
1.00
0
10
20
30
40

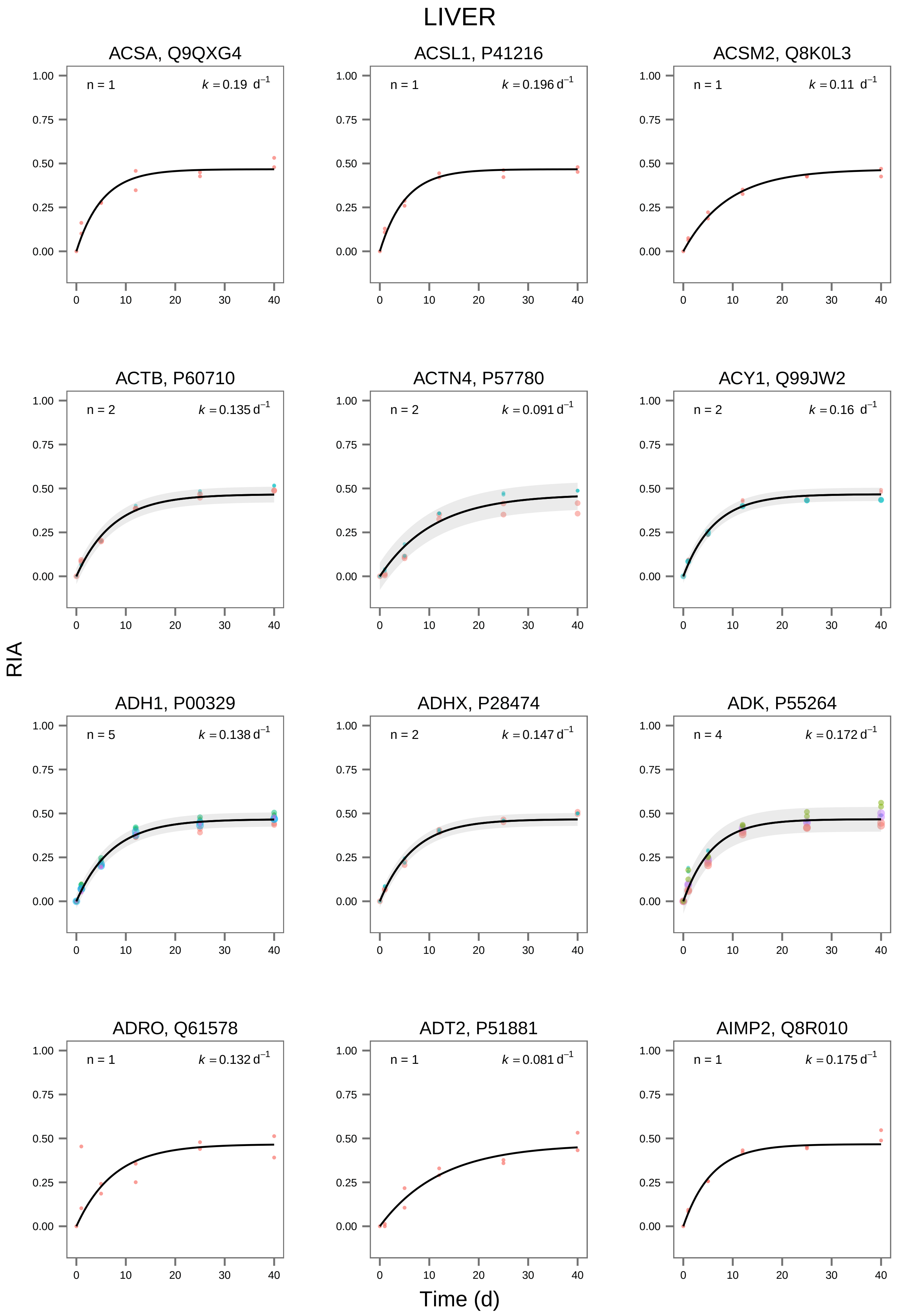
LIVER
ACSA, Q9QXG4
n = 1
k = 0.19 d⁻¹
ACSL1, P41216
n = 1
k = 0.196 d⁻¹
ACSM2, Q8K0L3
n = 1
k = 0.11 d⁻¹
ACTB, P60710
n = 2
k = 0.135 d⁻¹
ACTN4, P57780
n = 2
k = 0.091 d⁻¹
ACY1, Q99JW2
n = 2
k = 0.16 d⁻¹
ADH1, P00329
n = 5
k = 0.138 d⁻¹
ADHX, P28474
n = 2
k = 0.147 d⁻¹
ADK, P55264
n = 4
k = 0.172 d⁻¹
ADRO, Q61578
n = 1
k = 0.132 d⁻¹
ADT2, P51881
n = 1
k = 0.081 d⁻¹
AIMP2, Q8R010
n = 1
k = 0.175 d⁻¹
RIA
Time (d)
1.00
0.75
0.50
0.25
0.00
0
10
20
30
40

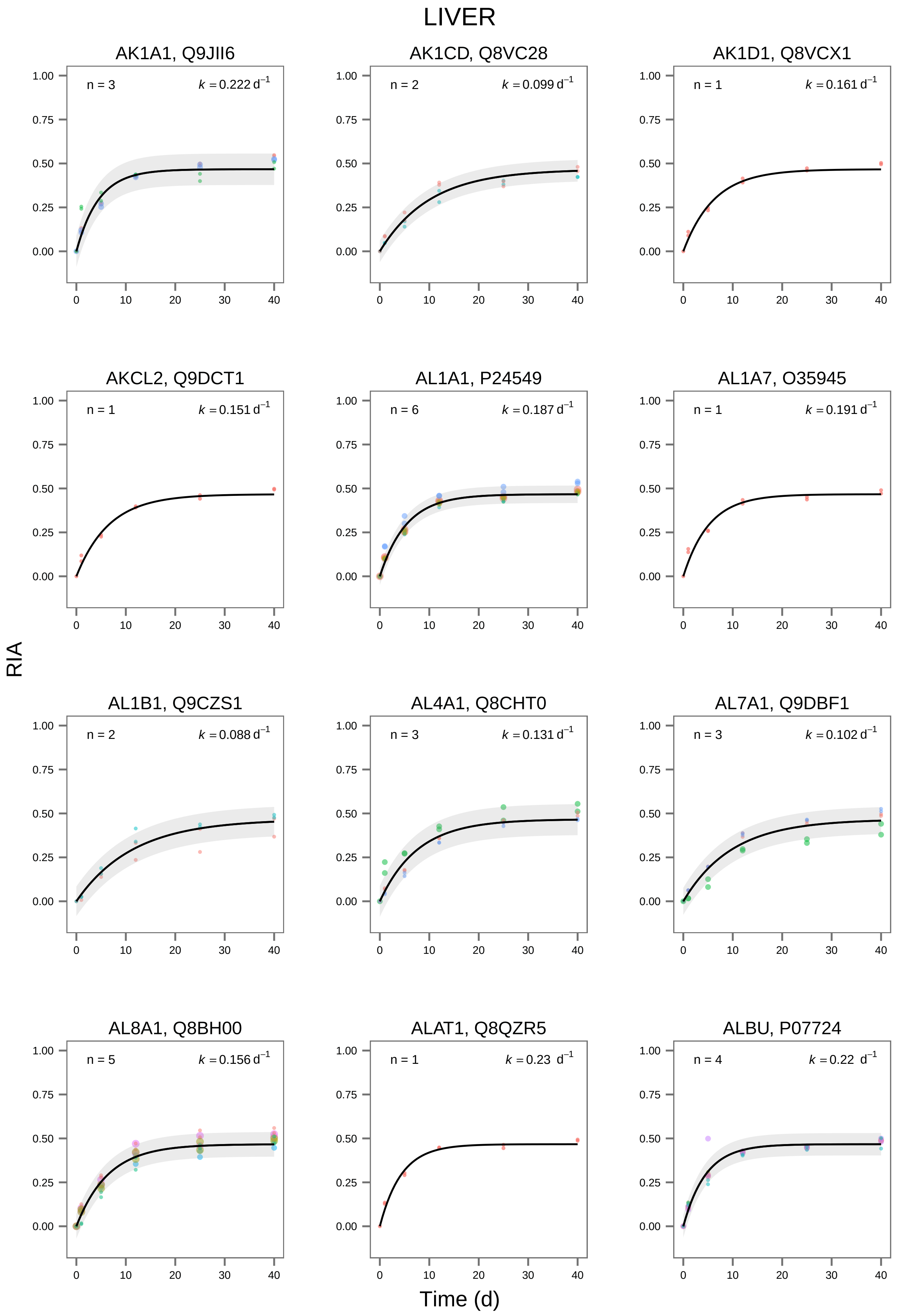
LIVER
AK1A1, Q9JII6
n = 3
k = 0.222 d⁻¹
AK1CD, Q8VC28
n = 2
k = 0.099 d⁻¹
AK1D1, Q8VCX1
n = 1
k = 0.161 d⁻¹
AKCL2, Q9DCT1
n = 1
k = 0.151 d⁻¹
AL1A1, P24549
n = 6
k = 0.187 d⁻¹
AL1A7, O35945
n = 1
k = 0.191 d⁻¹
AL1B1, Q9CZS1
n = 2
k = 0.088 d⁻¹
AL4A1, Q8CHT0
n = 3
k = 0.131 d⁻¹
AL7A1, Q9DBF1
n = 3
k = 0.102 d⁻¹
AL8A1, Q8BH00
n = 5
k = 0.156 d⁻¹
ALAT1, Q8QZR5
n = 1
k = 0.23 d⁻¹
ALBU, P07724
n = 4
k = 0.22 d⁻¹
RIA
Time (d)

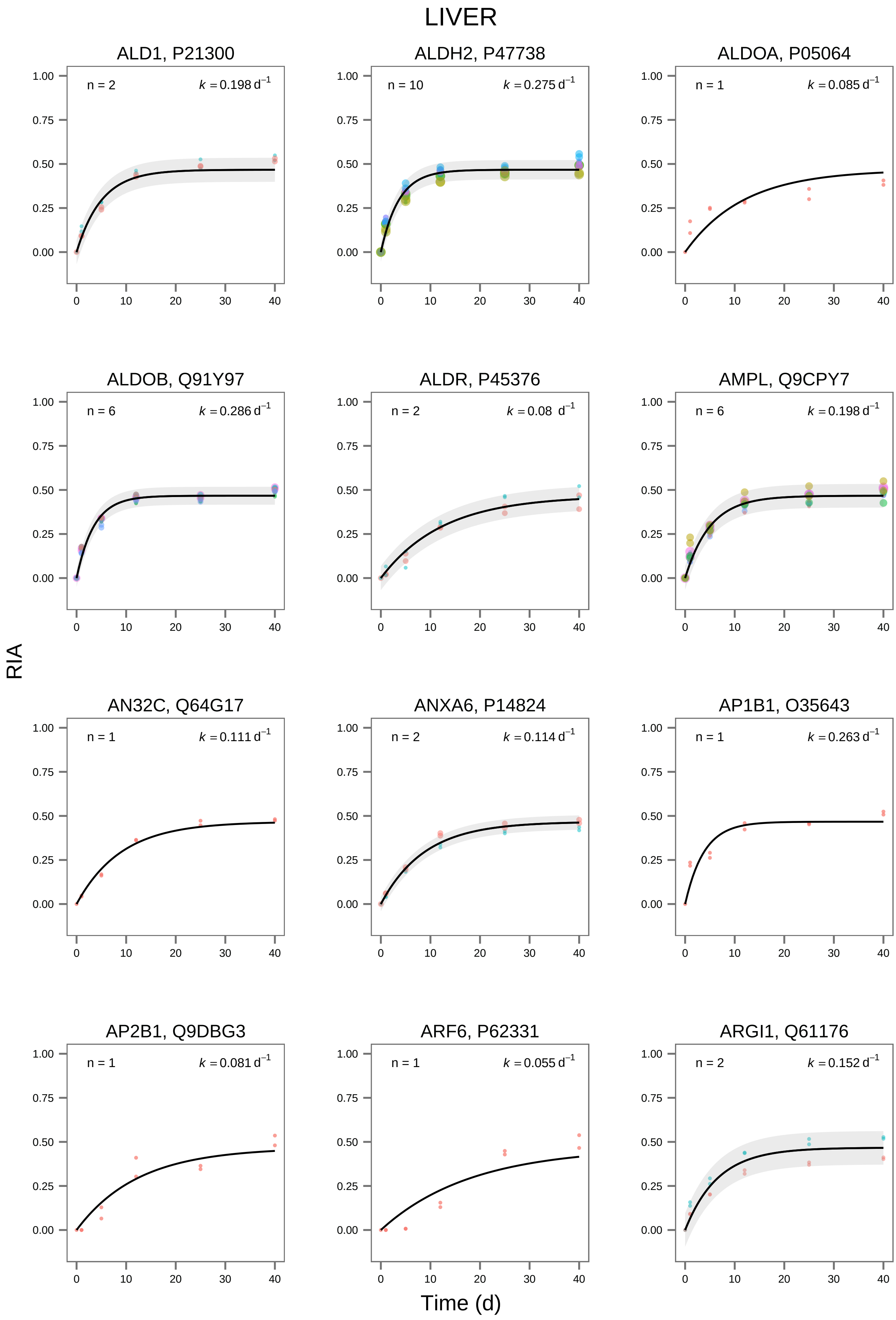
LIVER
ALD1, P21300
n = 2
k = 0.198 d−1
ALDH2, P47738
n = 10
k = 0.275 d−1
ALDOA, P05064
n = 1
k = 0.085 d−1
ALDOB, Q91Y97
n = 6
k = 0.286 d−1
ALDR, P45376
n = 2
k = 0.08 d−1
AMPL, Q9CPY7
n = 6
k = 0.198 d−1
AN32C, Q64G17
n = 1
k = 0.111 d−1
ANXA6, P14824
n = 2
k = 0.114 d−1
AP1B1, O35643
n = 1
k = 0.263 d−1
AP2B1, Q9DBG3
n = 1
k = 0.081 d−1
ARF6, P62331
n = 1
k = 0.055 d−1
ARGI1, Q61176
n = 2
k = 0.152 d−1
RIA
Time (d)
0.00
0.25
0.50
0.75
1.00
0
10
20
30
40

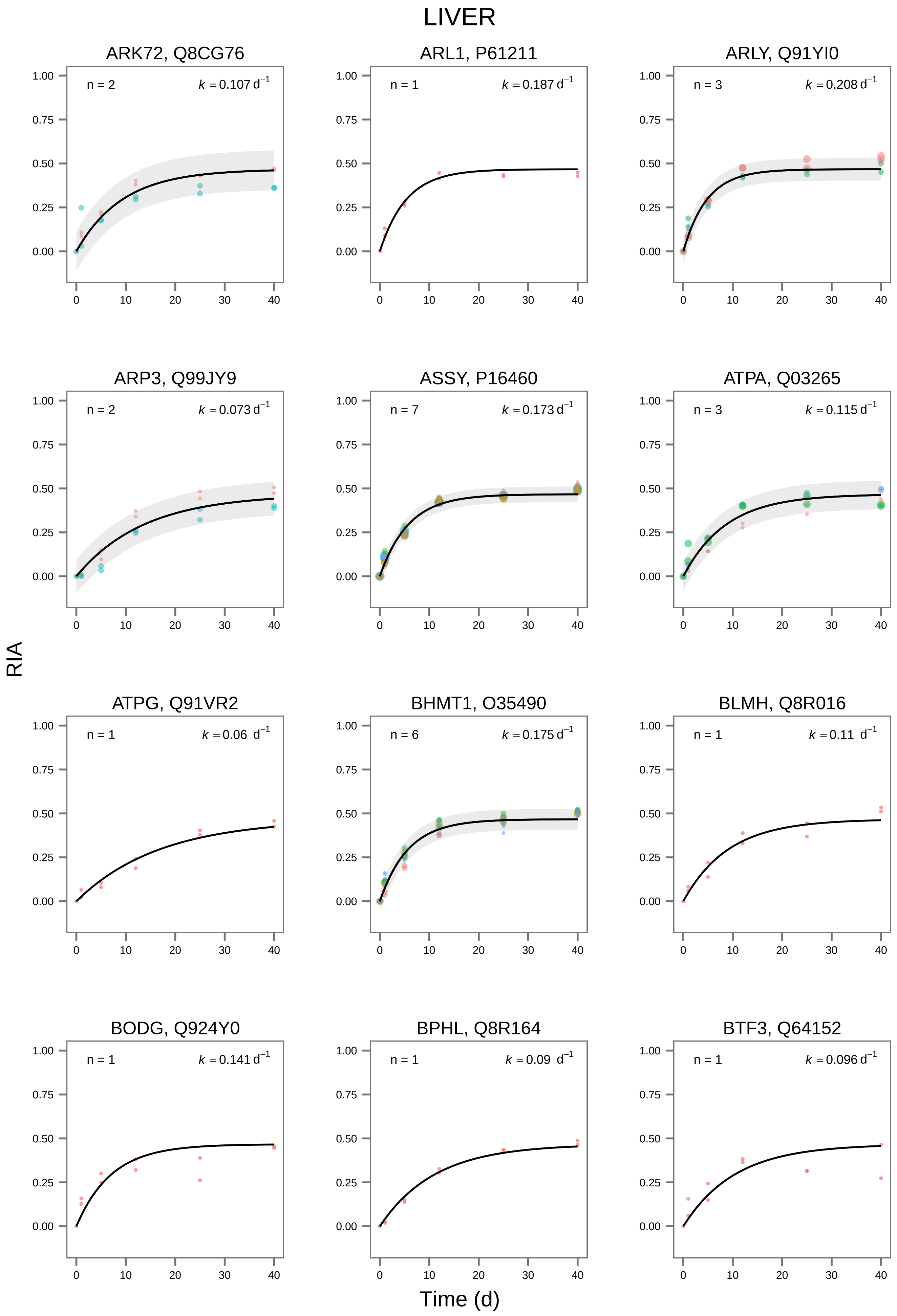
LIVER
ARK72, Q8CG76
n = 2
k = 0.107 d−1
ARL1, P61211
n = 1
k = 0.187 d−1
ARLY, Q91YI0
n = 3
k = 0.208 d−1
ARP3, Q99JY9
n = 2
k = 0.073 d−1
ASSY, P16460
n = 7
k = 0.173 d−1
ATPA, Q03265
n = 3
k = 0.115 d−1
ATPG, Q91VR2
n = 1
k = 0.06 d−1
BHMT1, O35490
n = 6
k = 0.175 d−1
BLMH, Q8R016
n = 1
k = 0.11 d−1
BODG, Q924Y0
n = 1
k = 0.141 d−1
BPHL, Q8R164
n = 1
k = 0.09 d−1
BTF3, Q64152
n = 1
k = 0.096 d−1
RIA
Time (d)

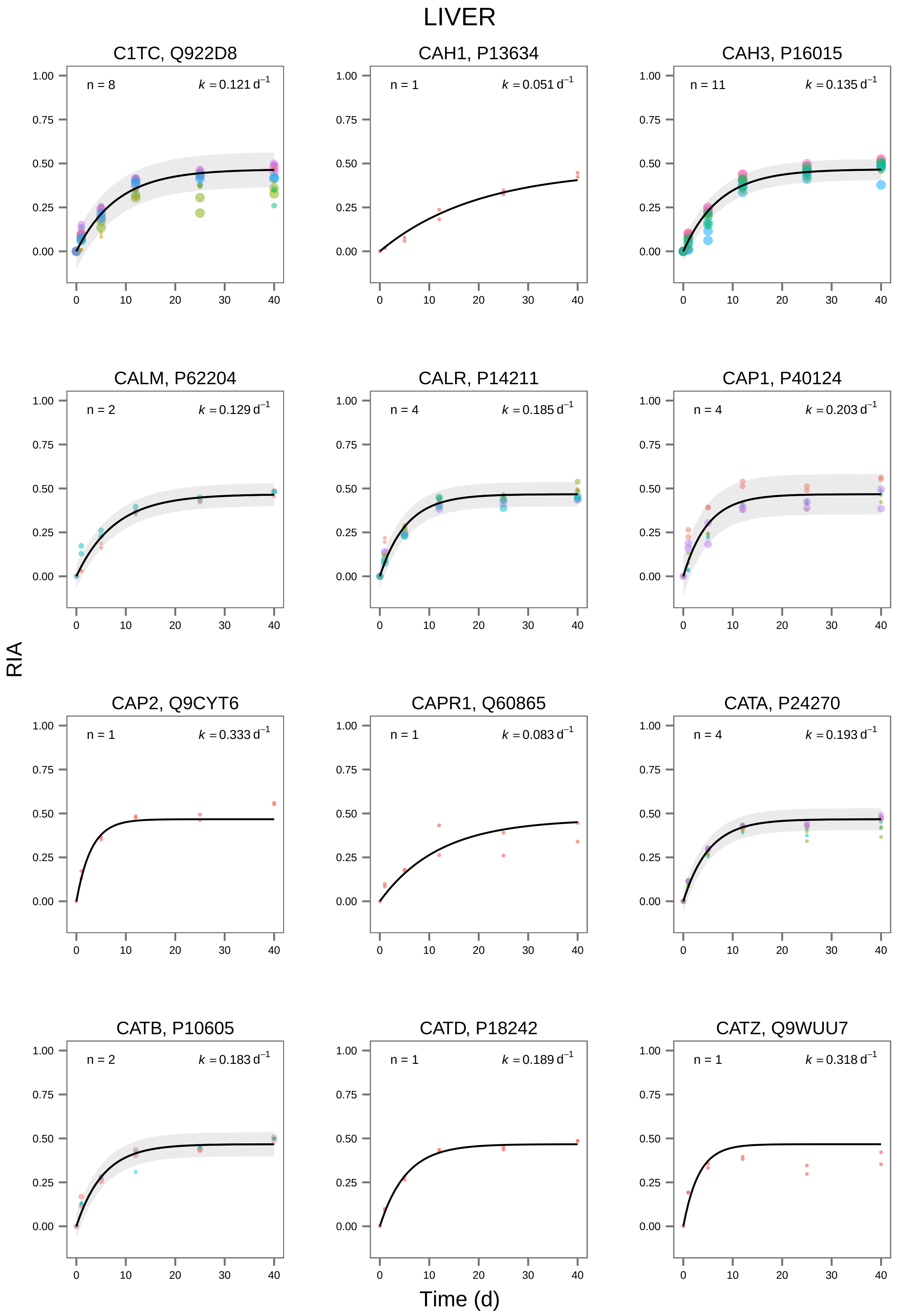
LIVER
C1TC, Q922D8
n = 8
k = 0.121 d−1
CAH1, P13634
n = 1
k = 0.051 d−1
CAH3, P16015
n = 11
k = 0.135 d−1
CALM, P62204
n = 2
k = 0.129 d−1
CALR, P14211
n = 4
k = 0.185 d−1
CAP1, P40124
n = 4
k = 0.203 d−1
CAP2, Q9CYT6
n = 1
k = 0.333 d−1
CAPR1, Q60865
n = 1
k = 0.083 d−1
CATA, P24270
n = 4
k = 0.193 d−1
CATB, P10605
n = 2
k = 0.183 d−1
CATD, P18242
n = 1
k = 0.189 d−1
CATZ, Q9WUU7
n = 1
k = 0.318 d−1
RIA
Time (d)
1.00
0.75
0.50
0.25
0.00
0
10
20
30
40

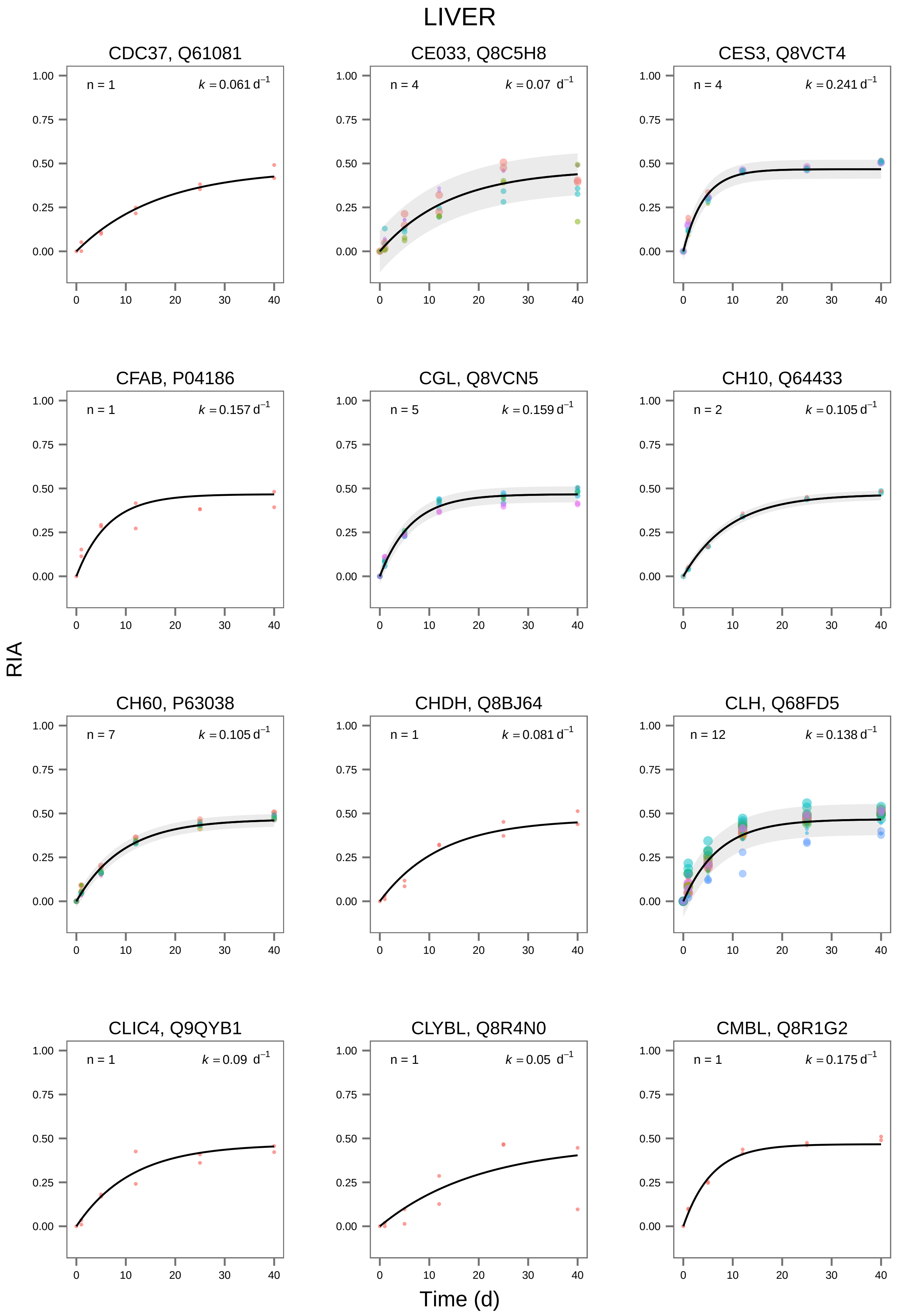
LIVER
CDC37, Q61081
n = 1
k = 0.061 d⁻¹
CE033, Q8C5H8
n = 4
k = 0.07 d⁻¹
CES3, Q8VCT4
n = 4
k = 0.241 d⁻¹
CFAB, P04186
n = 1
k = 0.157 d⁻¹
CGL, Q8VCN5
n = 5
k = 0.159 d⁻¹
CH10, Q64433
n = 2
k = 0.105 d⁻¹
CH60, P63038
n = 7
k = 0.105 d⁻¹
CHDH, Q8BJ64
n = 1
k = 0.081 d⁻¹
CLH, Q68FD5
n = 12
k = 0.138 d⁻¹
CLIC4, Q9QYB1
n = 1
k = 0.09 d⁻¹
CLYBL, Q8R4N0
n = 1
k = 0.05 d⁻¹
CMBL, Q8R1G2
n = 1
k = 0.175 d⁻¹
RIA
Time (d)

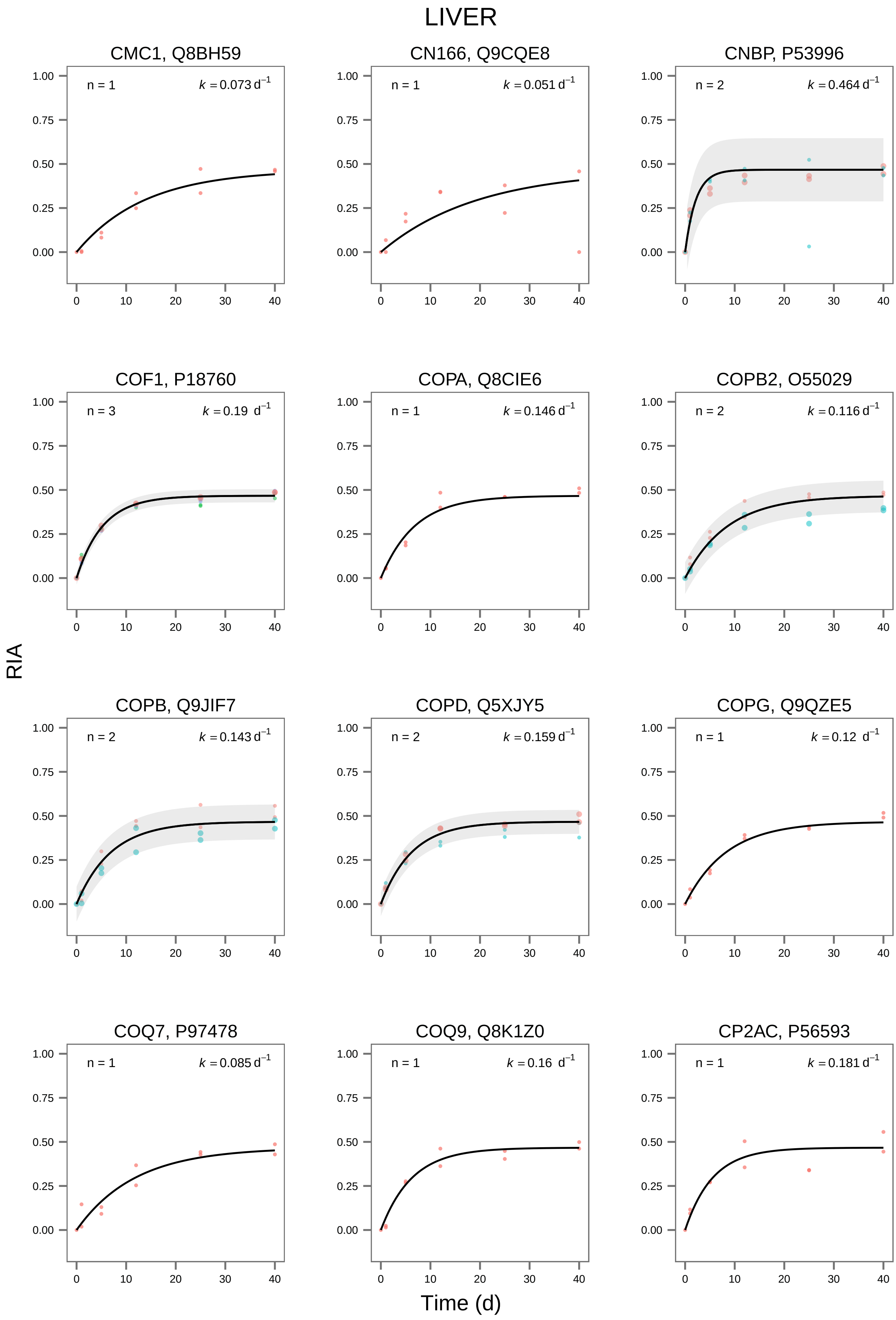
LIVER
CMC1, Q8BH59
n = 1
k = 0.073 d−1
CN166, Q9CQE8
n = 1
k = 0.051 d−1
CNBP, P53996
n = 2
k = 0.464 d−1
COF1, P18760
n = 3
k = 0.19 d−1
COPA, Q8CIE6
n = 1
k = 0.146 d−1
COPB2, O55029
n = 2
k = 0.116 d−1
COPB, Q9JIF7
n = 2
k = 0.143 d−1
COPD, Q5XJY5
n = 2
k = 0.159 d−1
COPG, Q9QZE5
n = 1
k = 0.12 d−1
COQ7, P97478
n = 1
k = 0.085 d−1
COQ9, Q8K1Z0
n = 1
k = 0.16 d−1
CP2AC, P56593
n = 1
k = 0.181 d−1
RIA
Time (d)

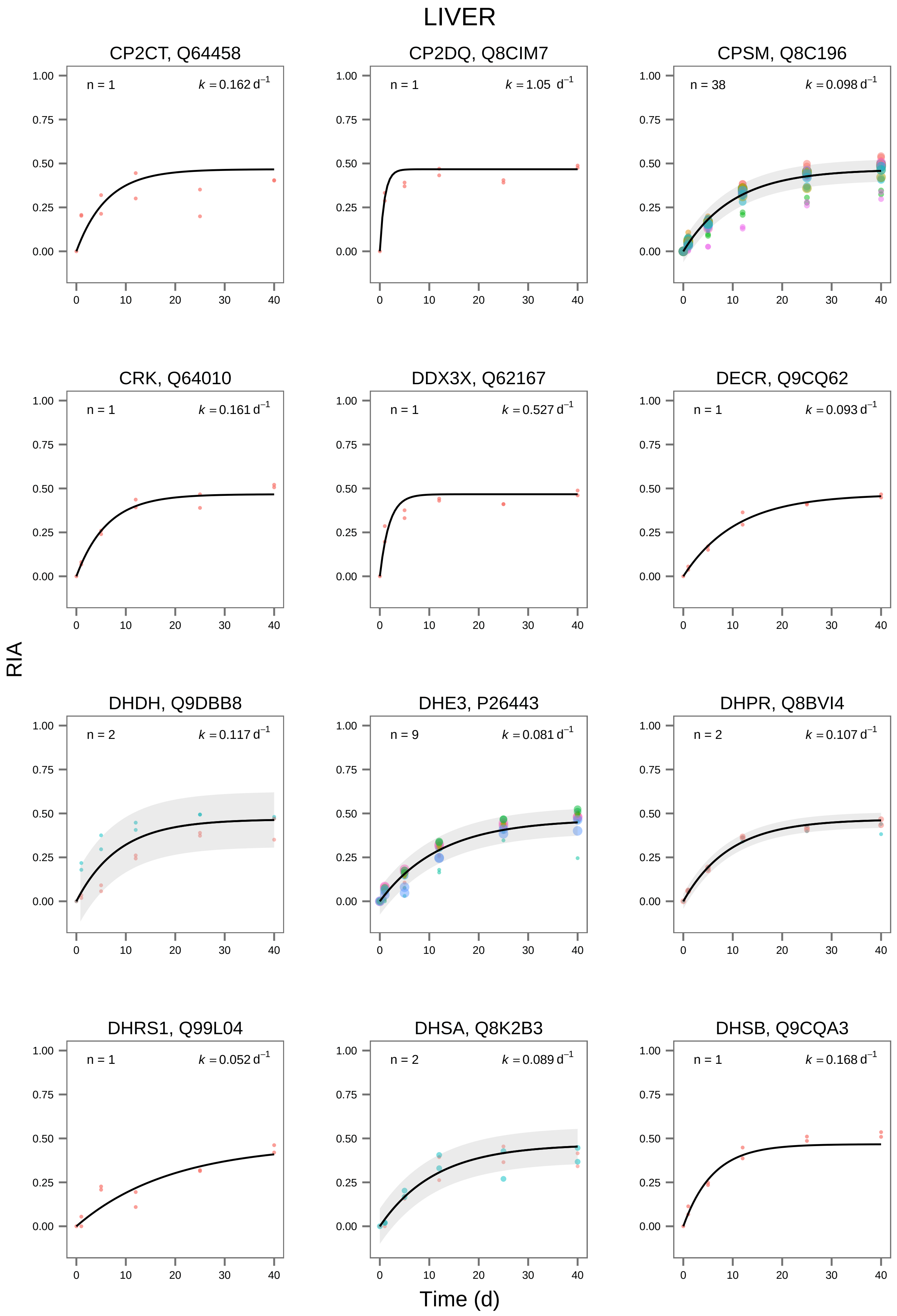
LIVER
CP2CT, Q64458
n = 1
k = 0.162 d⁻¹
CP2DQ, Q8CIM7
n = 1
k = 1.05 d⁻¹
CPSM, Q8C196
n = 38
k = 0.098 d⁻¹
CRK, Q64010
n = 1
k = 0.161 d⁻¹
DDX3X, Q62167
n = 1
k = 0.527 d⁻¹
DECR, Q9CQ62
n = 1
k = 0.093 d⁻¹
DHDH, Q9DBB8
n = 2
k = 0.117 d⁻¹
DHE3, P26443
n = 9
k = 0.081 d⁻¹
DHPR, Q8BVI4
n = 2
k = 0.107 d⁻¹
DHRS1, Q99L04
n = 1
k = 0.052 d⁻¹
DHSA, Q8K2B3
n = 2
k = 0.089 d⁻¹
DHSB, Q9CQA3
n = 1
k = 0.168 d⁻¹
RIA
Time (d)

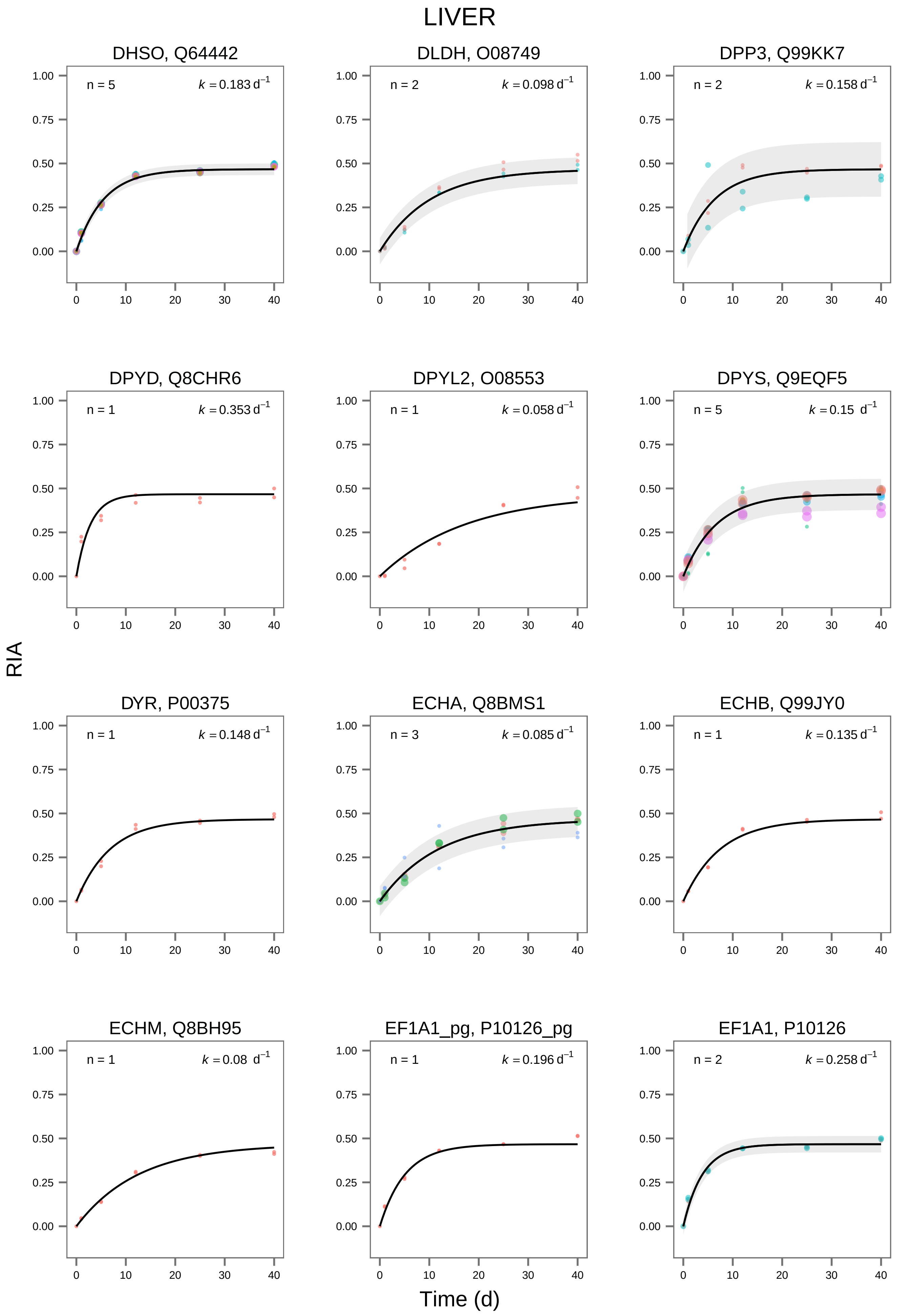

LIVER
DHSO, Q64442
n = 5
k = 0.183 d^-1
DLDH, O08749
n = 2
k = 0.098 d^-1
DPP3, Q99KK7
n = 2
k = 0.158 d^-1
DPYD, Q8CHR6
n = 1
k = 0.353 d^-1
DPYL2, O08553
n = 1
k = 0.058 d^-1
DPYS, Q9EQF5
n = 5
k = 0.15 d^-1
DYR, P00375
n = 1
k = 0.148 d^-1
ECHA, Q8BMS1
n = 3
k = 0.085 d^-1
ECHB, Q99JY0
n = 1
k = 0.135 d^-1
ECHM, Q8BH95
n = 1
k = 0.08 d^-1
EF1A1_pg, P10126_pg
n = 1
k = 0.196 d^-1
EF1A1, P10126
n = 2
k = 0.258 d^-1
RIA
Time (d)

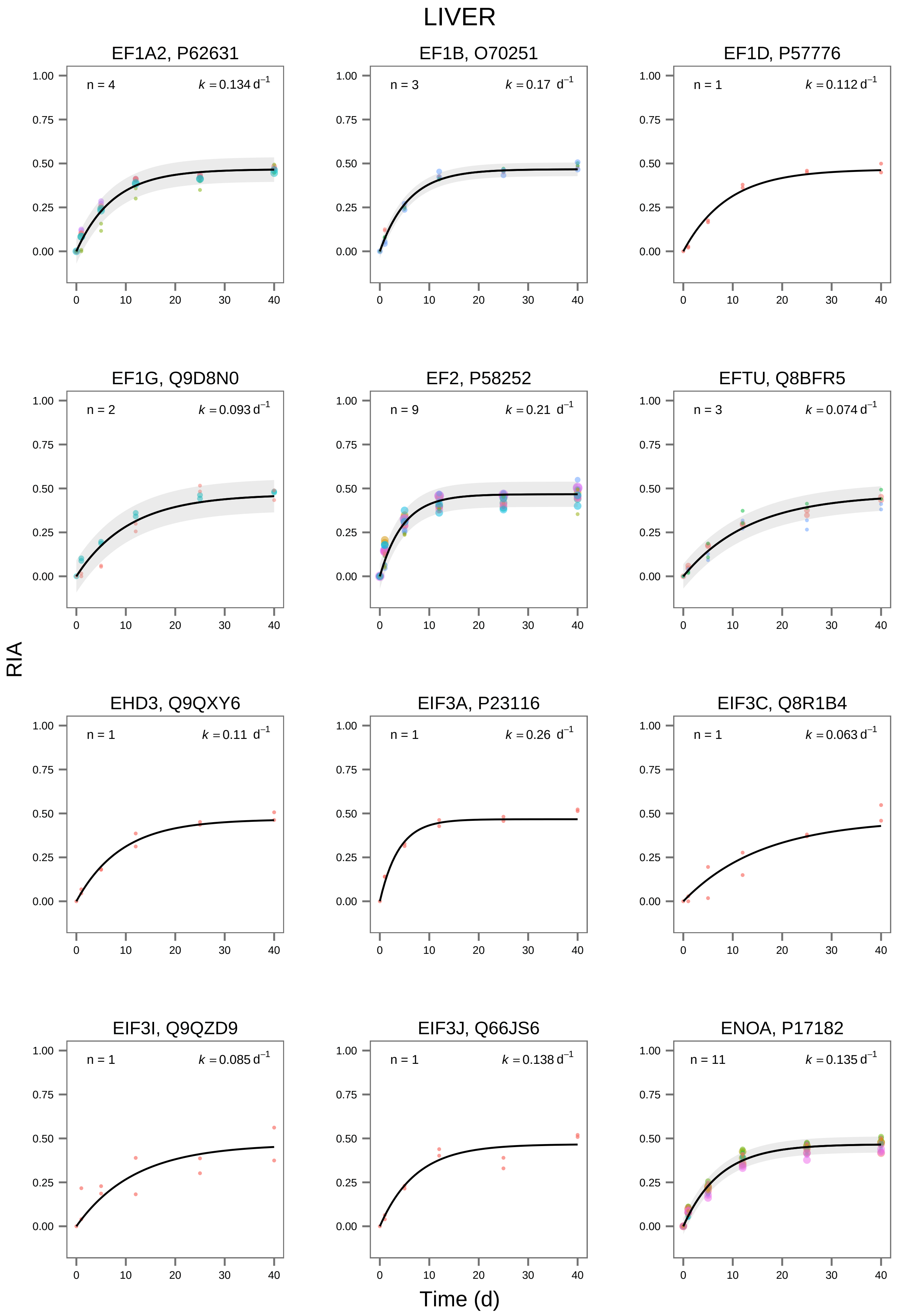
LIVER
EF1A2, P62631
n = 4
k = 0.134 d−1
EF1B, O70251
n = 3
k = 0.17 d−1
EF1D, P57776
n = 1
k = 0.112 d−1
EF1G, Q9D8N0
n = 2
k = 0.093 d−1
EF2, P58252
n = 9
k = 0.21 d−1
EFTU, Q8BFR5
n = 3
k = 0.074 d−1
EHD3, Q9QXY6
n = 1
k = 0.11 d−1
EIF3A, P23116
n = 1
k = 0.26 d−1
EIF3C, Q8R1B4
n = 1
k = 0.063 d−1
EIF3I, Q9QZD9
n = 1
k = 0.085 d−1
EIF3J, Q66JS6
n = 1
k = 0.138 d−1
ENOA, P17182
n = 11
k = 0.135 d−1
RIA
Time (d)
0.00
0.25
0.50
0.75
1.00
0
10
20
30
40

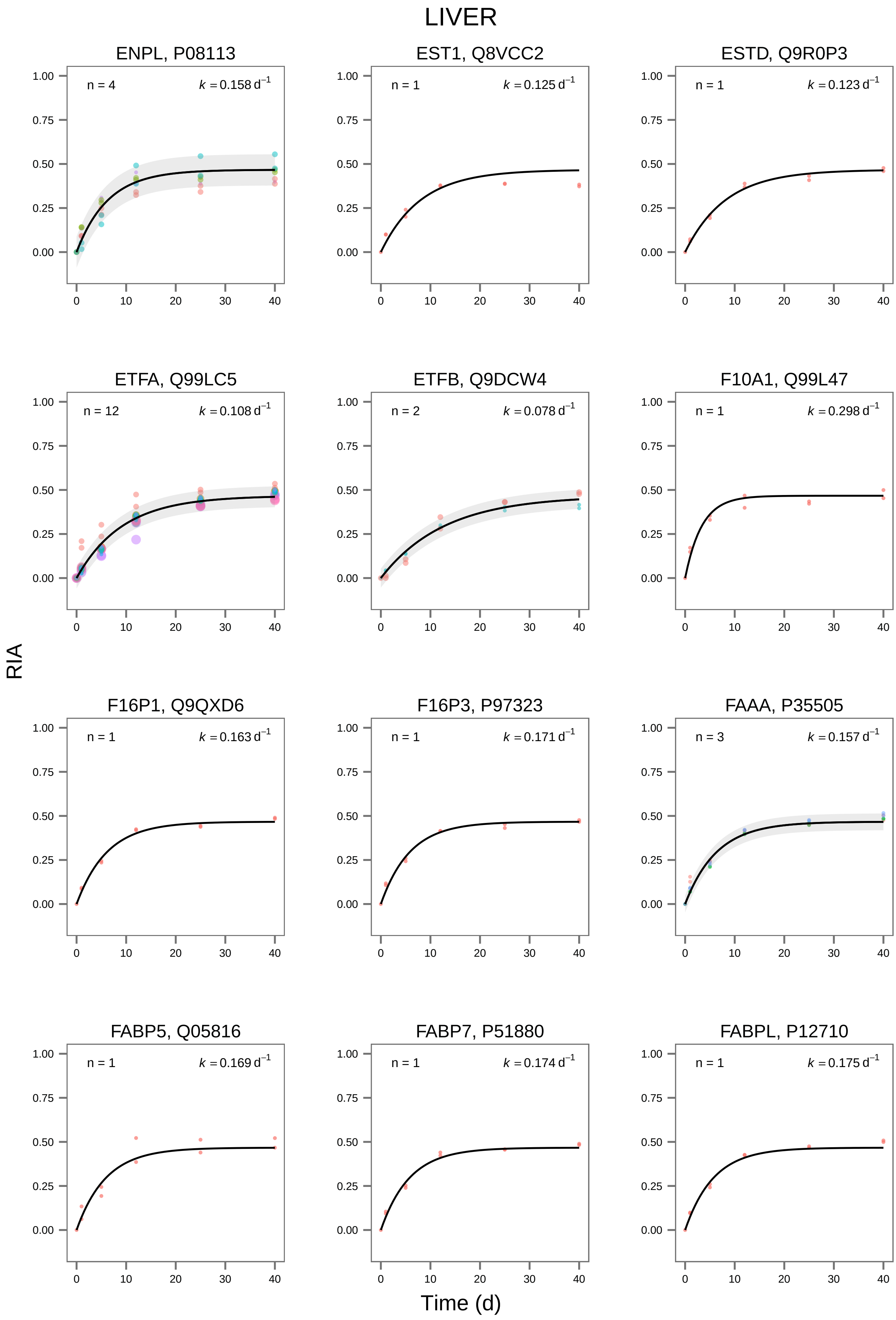
LIVER
ENPL, P08113
n = 4
k = 0.158 d−1
EST1, Q8VCC2
n = 1
k = 0.125 d−1
ESTD, Q9R0P3
n = 1
k = 0.123 d−1
ETFA, Q99LC5
n = 12
k = 0.108 d−1
ETFB, Q9DCW4
n = 2
k = 0.078 d−1
F10A1, Q99L47
n = 1
k = 0.298 d−1
F16P1, Q9QXD6
n = 1
k = 0.163 d−1
F16P3, P97323
n = 1
k = 0.171 d−1
FAAA, P35505
n = 3
k = 0.157 d−1
FABP5, Q05816
n = 1
k = 0.169 d−1
FABP7, P51880
n = 1
k = 0.174 d−1
FABPL, P12710
n = 1
k = 0.175 d−1
RIA
Time (d)

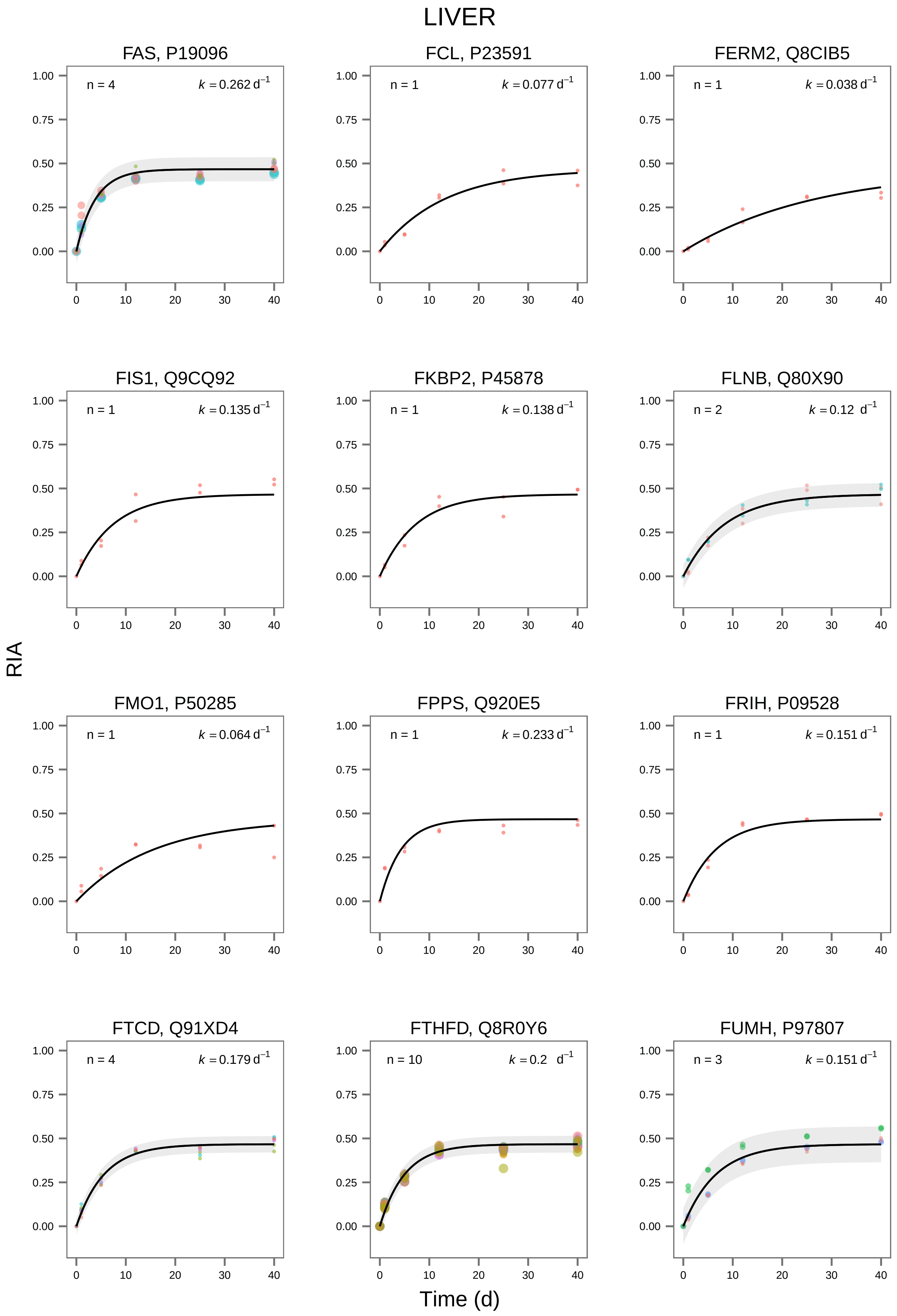
LIVER
FAS, P19096
n = 4
k = 0.262 d^-1
FCL, P23591
n = 1
k = 0.077 d^-1
FERM2, Q8CIB5
n = 1
k = 0.038 d^-1
FIS1, Q9CQ92
n = 1
k = 0.135 d^-1
FKBP2, P45878
n = 1
k = 0.138 d^-1
FLNB, Q80X90
n = 2
k = 0.12 d^-1
FMO1, P50285
n = 1
k = 0.064 d^-1
FPPS, Q920E5
n = 1
k = 0.233 d^-1
FRIH, P09528
n = 1
k = 0.151 d^-1
FTCD, Q91XD4
n = 4
k = 0.179 d^-1
FTHFD, Q8R0Y6
n = 10
k = 0.2 d^-1
FUMH, P97807
n = 3
k = 0.151 d^-1
RIA
Time (d)

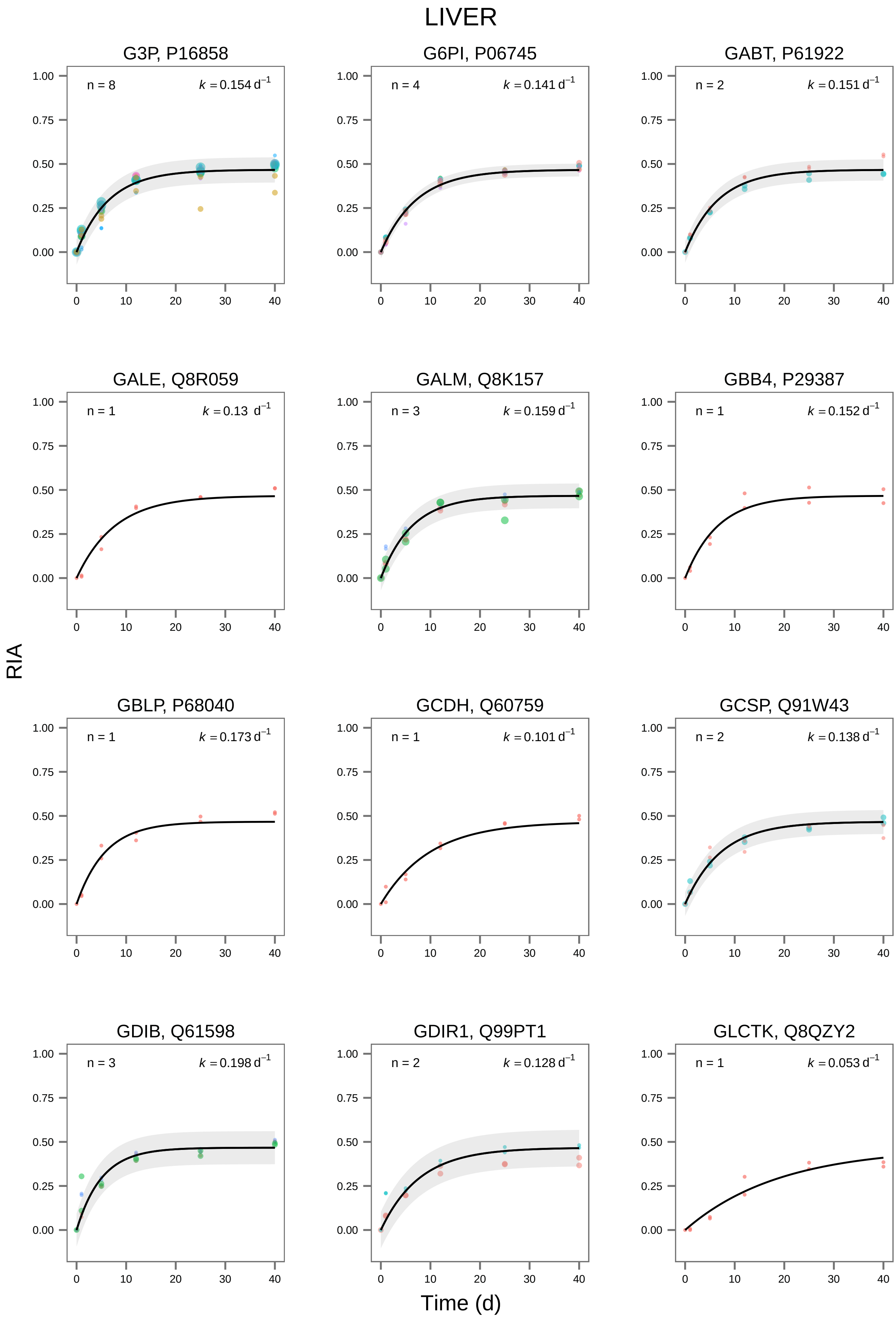

LIVER
G3P, P16858
n = 8
k = 0.154 d⁻¹
G6PI, P06745
n = 4
k = 0.141 d⁻¹
GABT, P61922
n = 2
k = 0.151 d⁻¹
GALE, Q8R059
n = 1
k = 0.13 d⁻¹
GALM, Q8K157
n = 3
k = 0.159 d⁻¹
GBB4, P29387
n = 1
k = 0.152 d⁻¹
GBLP, P68040
n = 1
k = 0.173 d⁻¹
GCDH, Q60759
n = 1
k = 0.101 d⁻¹
GCSP, Q91W43
n = 2
k = 0.138 d⁻¹
GDIB, Q61598
n = 3
k = 0.198 d⁻¹
GDIR1, Q99PT1
n = 2
k = 0.128 d⁻¹
GLCTK, Q8QZY2
n = 1
k = 0.053 d⁻¹
RIA
Time (d)
0.00
0.25
0.50
0.75
1.00
0
10
20
30
40

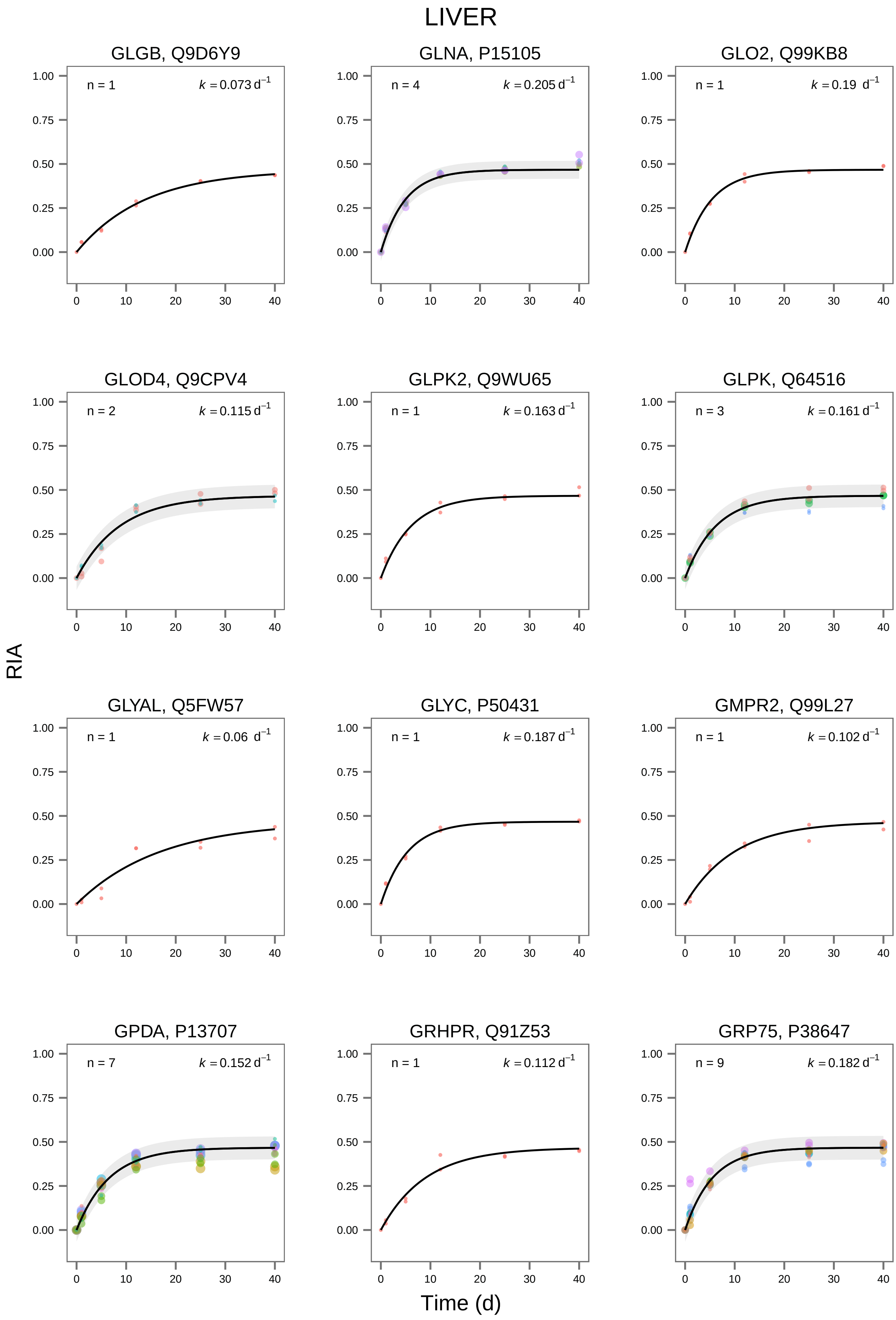
LIVER
GLGB, Q9D6Y9
n = 1
k = 0.073 d−1
GLNA, P15105
n = 4
k = 0.205 d−1
GLO2, Q99KB8
n = 1
k = 0.19 d−1
GLOD4, Q9CPV4
n = 2
k = 0.115 d−1
GLPK2, Q9WU65
n = 1
k = 0.163 d−1
GLPK, Q64516
n = 3
k = 0.161 d−1
GLYAL, Q5FW57
n = 1
k = 0.06 d−1
GLYC, P50431
n = 1
k = 0.187 d−1
GMPR2, Q99L27
n = 1
k = 0.102 d−1
GPDA, P13707
n = 7
k = 0.152 d−1
GRHPR, Q91Z53
n = 1
k = 0.112 d−1
GRP75, P38647
n = 9
k = 0.182 d−1
RIA
Time (d)
0.00
0.25
0.50
0.75
1.00
0
10
20
30
40

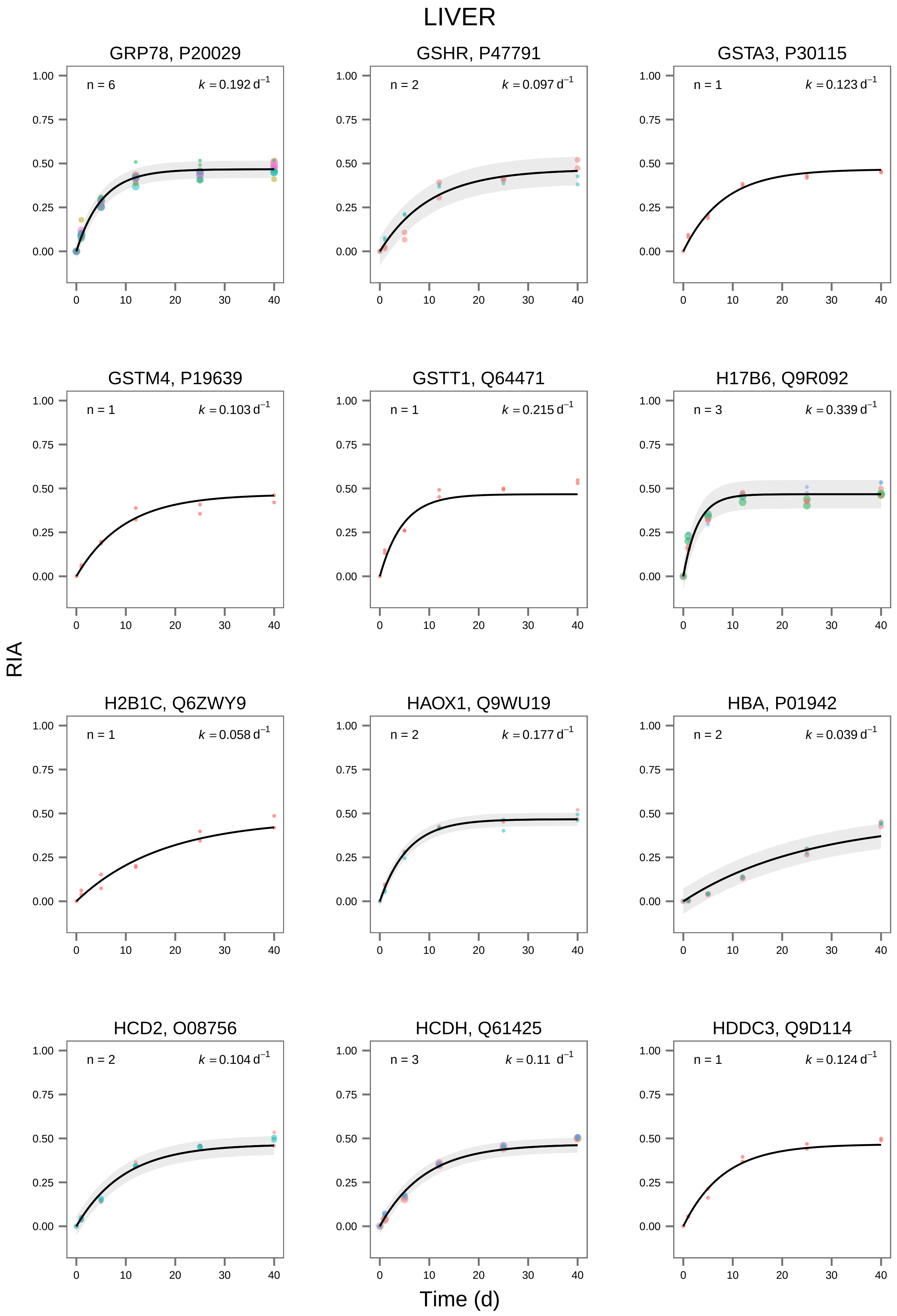
LIVER
GRP78, P20029
n = 6
k = 0.192 d−1
GSHR, P47791
n = 2
k = 0.097 d−1
GSTA3, P30115
n = 1
k = 0.123 d−1
GSTM4, P19639
n = 1
k = 0.103 d−1
GSTT1, Q64471
n = 1
k = 0.215 d−1
H17B6, Q9R092
n = 3
k = 0.339 d−1
H2B1C, Q6ZWY9
n = 1
k = 0.058 d−1
HAOX1, Q9WU19
n = 2
k = 0.177 d−1
HBA, P01942
n = 2
k = 0.039 d−1
HCD2, O08756
n = 2
k = 0.104 d−1
HCDH, Q61425
n = 3
k = 0.11 d−1
HDDC3, Q9D114
n = 1
k = 0.124 d−1
RIA
Time (d)
0.00
0.25
0.50
0.75
1.00
0
10
20
30
40

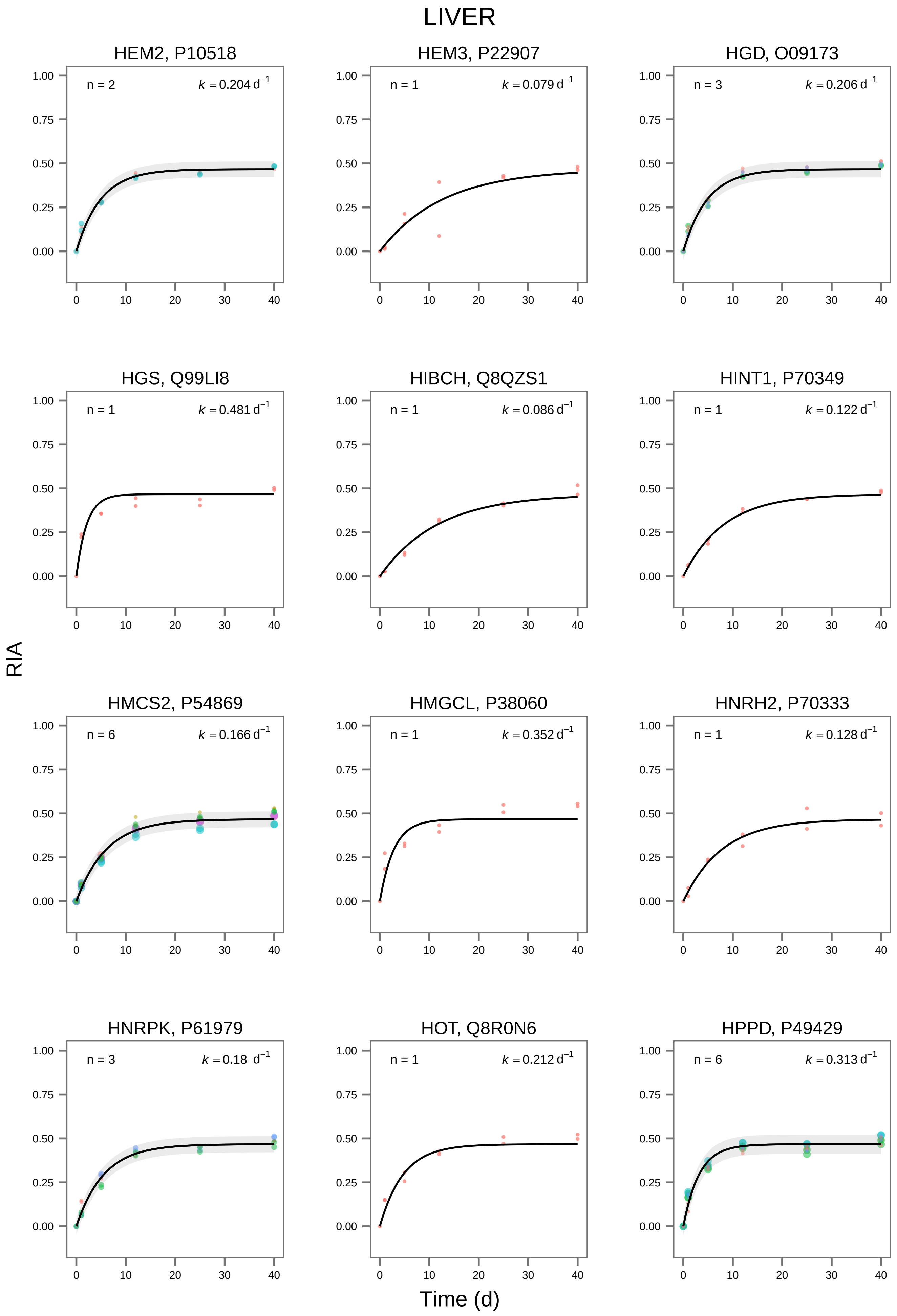
LIVER
HEM2, P10518
n = 2
k = 0.204 d^{-1}
HEM3, P22907
n = 1
k = 0.079 d^{-1}
HGD, O09173
n = 3
k = 0.206 d^{-1}
HGS, Q99LI8
n = 1
k = 0.481 d^{-1}
HIBCH, Q8QZS1
n = 1
k = 0.086 d^{-1}
HINT1, P70349
n = 1
k = 0.122 d^{-1}
HMCS2, P54869
n = 6
k = 0.166 d^{-1}
HMGCL, P38060
n = 1
k = 0.352 d^{-1}
HNRH2, P70333
n = 1
k = 0.128 d^{-1}
HNRPK, P61979
n = 3
k = 0.18 d^{-1}
HOT, Q8R0N6
n = 1
k = 0.212 d^{-1}
HPPD, P49429
n = 6
k = 0.313 d^{-1}
RIA
Time (d)

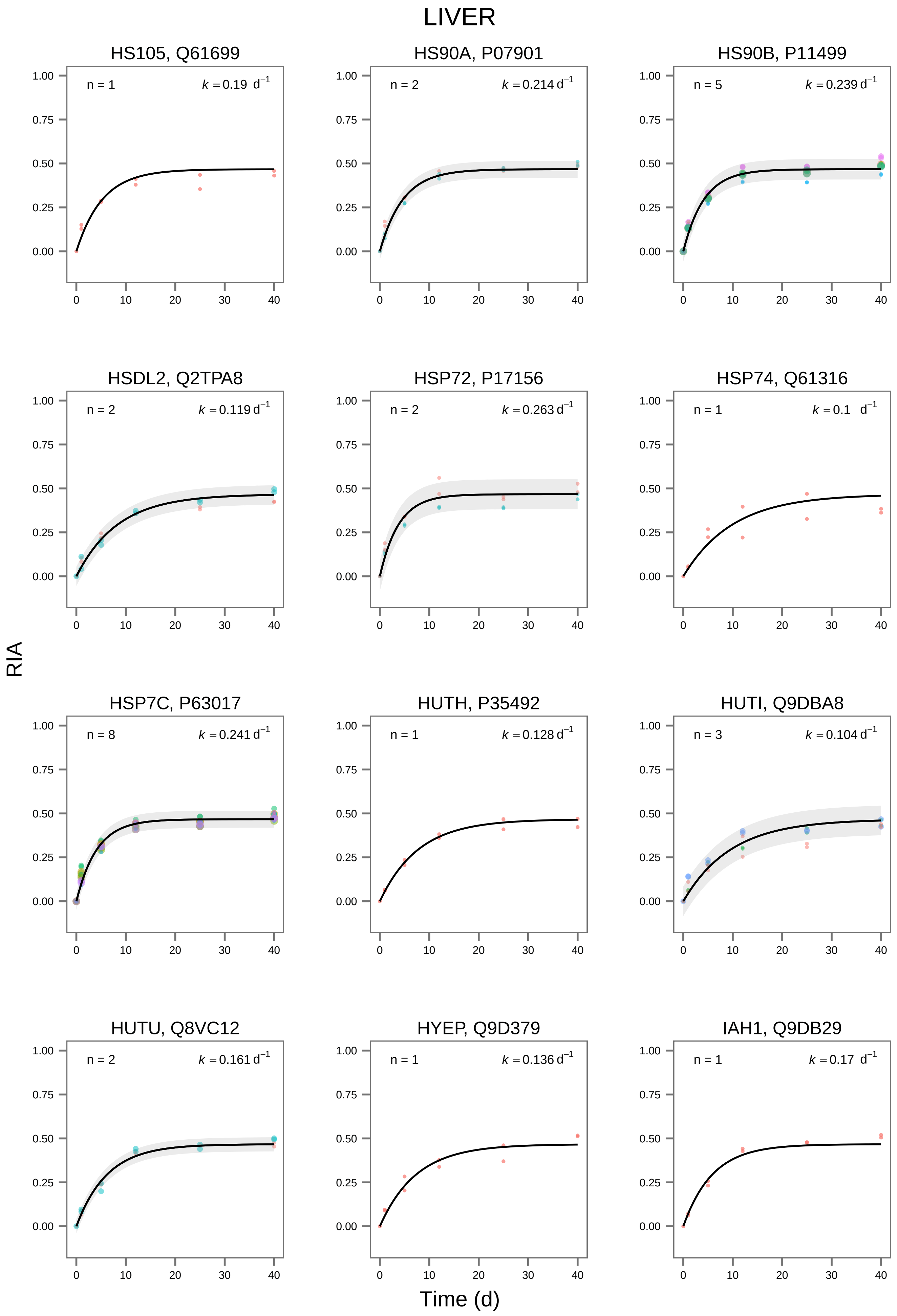

LIVER
HS105, Q61699
n = 1
k = 0.19 d⁻¹
HS90A, P07901
n = 2
k = 0.214 d⁻¹
HS90B, P11499
n = 5
k = 0.239 d⁻¹
HSDL2, Q2TPA8
n = 2
k = 0.119 d⁻¹
HSP72, P17156
n = 2
k = 0.263 d⁻¹
HSP74, Q61316
n = 1
k = 0.1 d⁻¹
HSP7C, P63017
n = 8
k = 0.241 d⁻¹
HUTH, P35492
n = 1
k = 0.128 d⁻¹
HUTI, Q9DBA8
n = 3
k = 0.104 d⁻¹
HUTU, Q8VC12
n = 2
k = 0.161 d⁻¹
HYEP, Q9D379
n = 1
k = 0.136 d⁻¹
IAH1, Q9DB29
n = 1
k = 0.17 d⁻¹
0.00
0.25
0.50
0.75
1.00
0
10
20
30
40
RIA
Time (d)

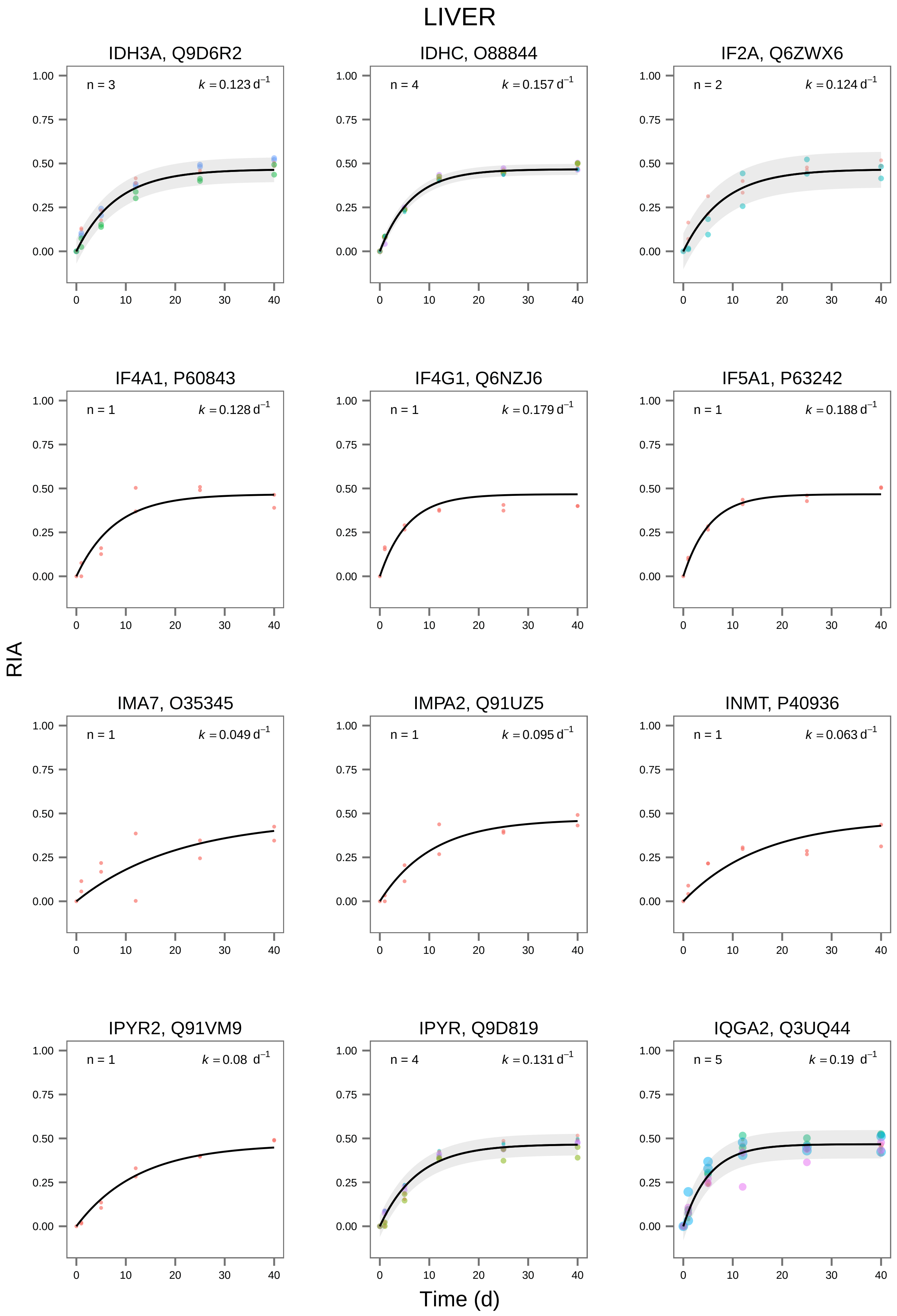
LIVER
IDH3A, Q9D6R2
n = 3
k = 0.123 d−1
IDHC, O88844
n = 4
k = 0.157 d−1
IF2A, Q6ZWX6
n = 2
k = 0.124 d−1
IF4A1, P60843
n = 1
k = 0.128 d−1
IF4G1, Q6NZJ6
n = 1
k = 0.179 d−1
IF5A1, P63242
n = 1
k = 0.188 d−1
IMA7, O35345
n = 1
k = 0.049 d−1
IMPA2, Q91UZ5
n = 1
k = 0.095 d−1
INMT, P40936
n = 1
k = 0.063 d−1
IPYR2, Q91VM9
n = 1
k = 0.08 d−1
IPYR, Q9D819
n = 4
k = 0.131 d−1
IQGA2, Q3UQ44
n = 5
k = 0.19 d−1
1.00
0.75
0.50
0.25
0.00
0
10
20
30
40
RIA
Time (d)

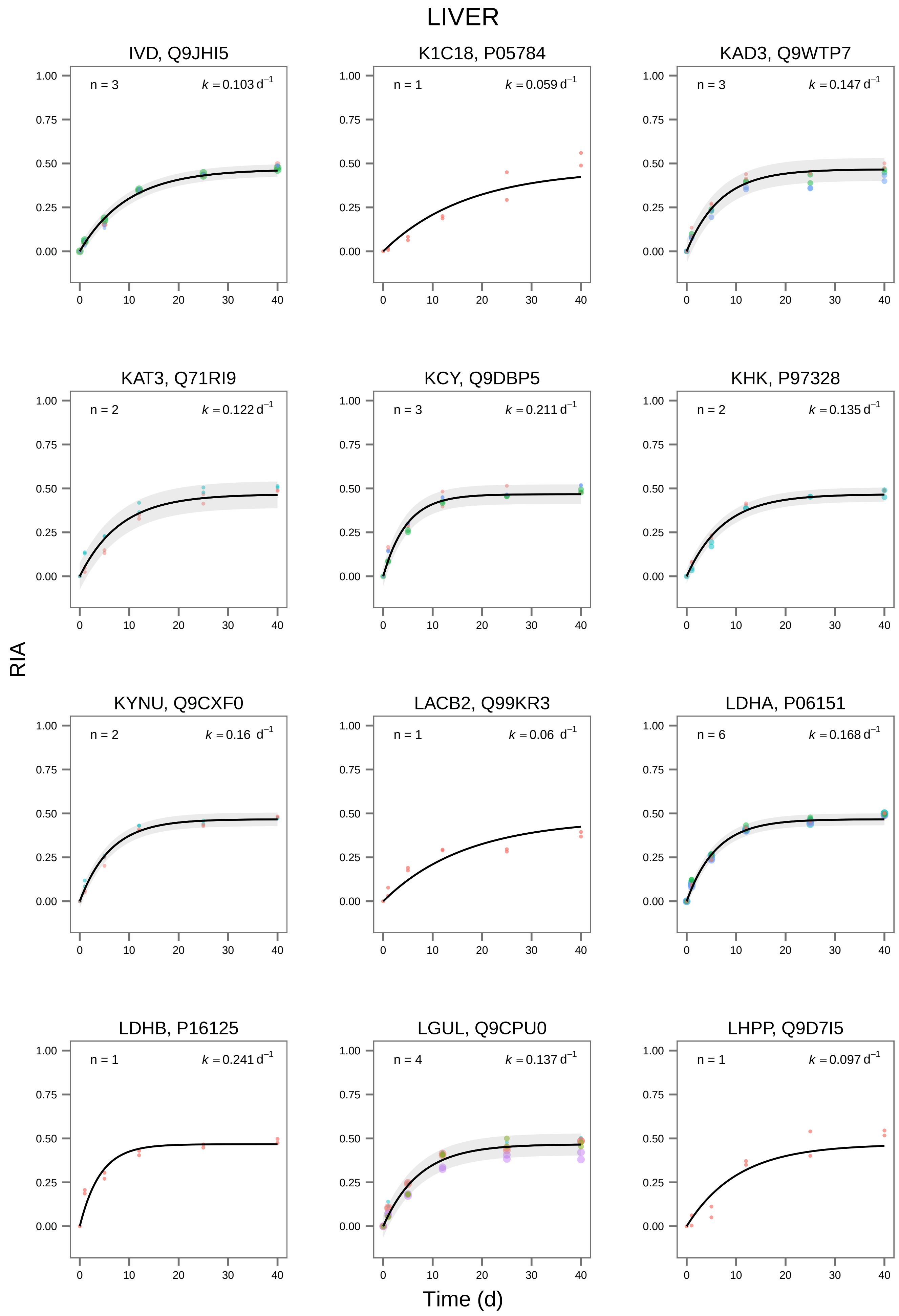
LIVER
IVD, Q9JHI5
n = 3
k = 0.103 d^{-1}
K1C18, P05784
n = 1
k = 0.059 d^{-1}
KAD3, Q9WTP7
n = 3
k = 0.147 d^{-1}
KAT3, Q71RI9
n = 2
k = 0.122 d^{-1}
KCY, Q9DBP5
n = 3
k = 0.211 d^{-1}
KHK, P97328
n = 2
k = 0.135 d^{-1}
KYNU, Q9CXF0
n = 2
k = 0.16 d^{-1}
LACB2, Q99KR3
n = 1
k = 0.06 d^{-1}
LDHA, P06151
n = 6
k = 0.168 d^{-1}
LDHB, P16125
n = 1
k = 0.241 d^{-1}
LGUL, Q9CPU0
n = 4
k = 0.137 d^{-1}
LHPP, Q9D7I5
n = 1
k = 0.097 d^{-1}
RIA
Time (d)

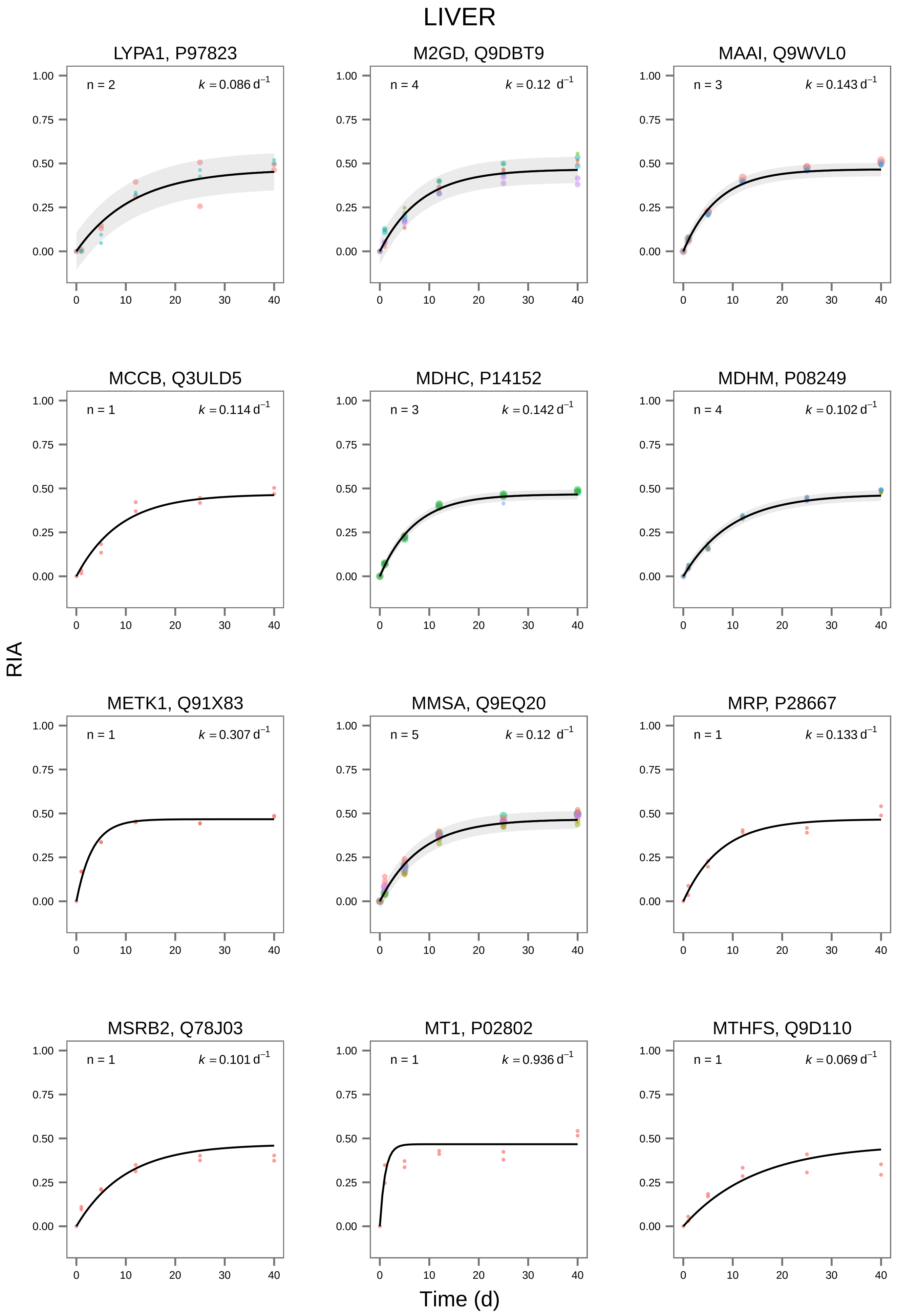

LIVER
LYPA1, P97823
n = 2
k = 0.086 d−1
M2GD, Q9DBT9
n = 4
k = 0.12 d−1
MAAI, Q9WVL0
n = 3
k = 0.143 d−1
MCCB, Q3ULD5
n = 1
k = 0.114 d−1
MDHC, P14152
n = 3
k = 0.142 d−1
MDHM, P08249
n = 4
k = 0.102 d−1
METK1, Q91X83
n = 1
k = 0.307 d−1
MMSA, Q9EQ20
n = 5
k = 0.12 d−1
MRP, P28667
n = 1
k = 0.133 d−1
MSRB2, Q78J03
n = 1
k = 0.101 d−1
MT1, P02802
n = 1
k = 0.936 d−1
MTHFS, Q9D110
n = 1
k = 0.069 d−1
RIA
Time (d)

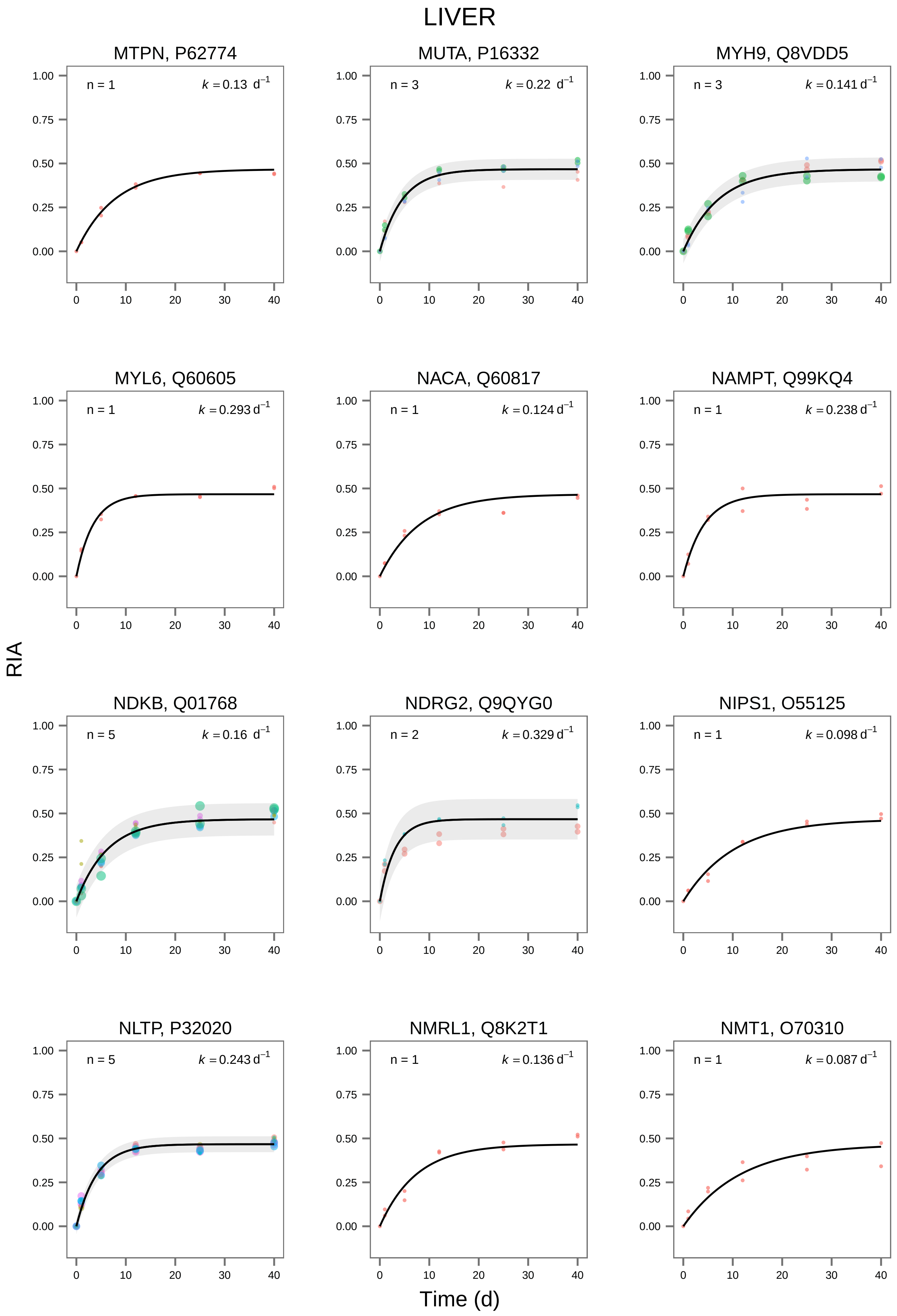
LIVER
MTPN, P62774
n = 1
k = 0.13 d−1
MUTA, P16332
n = 3
k = 0.22 d−1
MYH9, Q8VDD5
n = 3
k = 0.141 d−1
MYL6, Q60605
n = 1
k = 0.293 d−1
NACA, Q60817
n = 1
k = 0.124 d−1
NAMPT, Q99KQ4
n = 1
k = 0.238 d−1
NDKB, Q01768
n = 5
k = 0.16 d−1
NDRG2, Q9QYG0
n = 2
k = 0.329 d−1
NIPS1, O55125
n = 1
k = 0.098 d−1
NLTP, P32020
n = 5
k = 0.243 d−1
NMRL1, Q8K2T1
n = 1
k = 0.136 d−1
NMT1, O70310
n = 1
k = 0.087 d−1
RIA
Time (d)
1.00
0.75
0.50
0.25
0.00
0
10
20
30
40

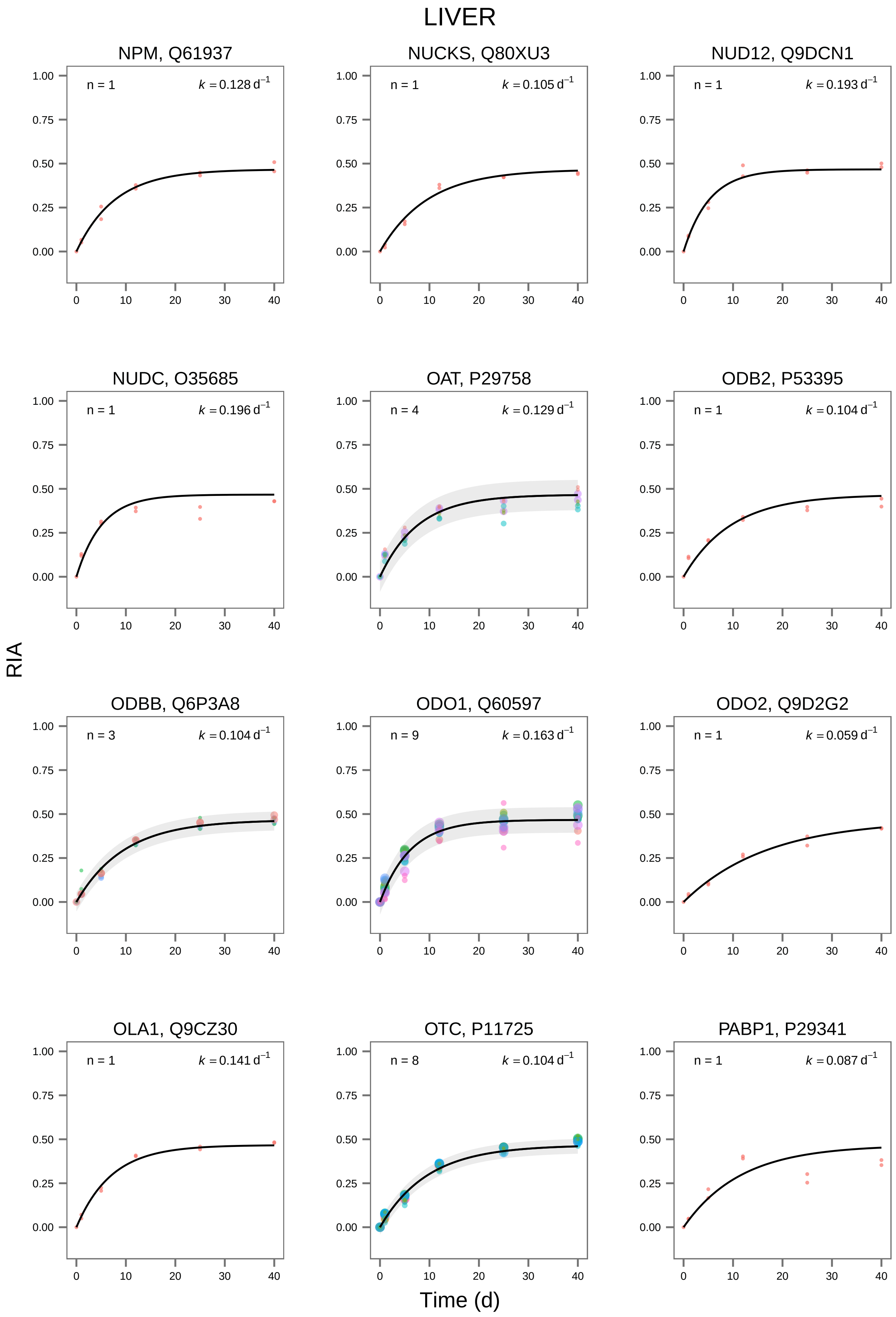

LIVER
NPM, Q61937
n = 1
k = 0.128 d−1
NUCKS, Q80XU3
n = 1
k = 0.105 d−1
NUD12, Q9DCN1
n = 1
k = 0.193 d−1
NUDC, O35685
n = 1
k = 0.196 d−1
OAT, P29758
n = 4
k = 0.129 d−1
ODB2, P53395
n = 1
k = 0.104 d−1
ODBB, Q6P3A8
n = 3
k = 0.104 d−1
ODO1, Q60597
n = 9
k = 0.163 d−1
ODO2, Q9D2G2
n = 1
k = 0.059 d−1
OLA1, Q9CZ30
n = 1
k = 0.141 d−1
OTC, P11725
n = 8
k = 0.104 d−1
PABP1, P29341
n = 1
k = 0.087 d−1
RIA
Time (d)

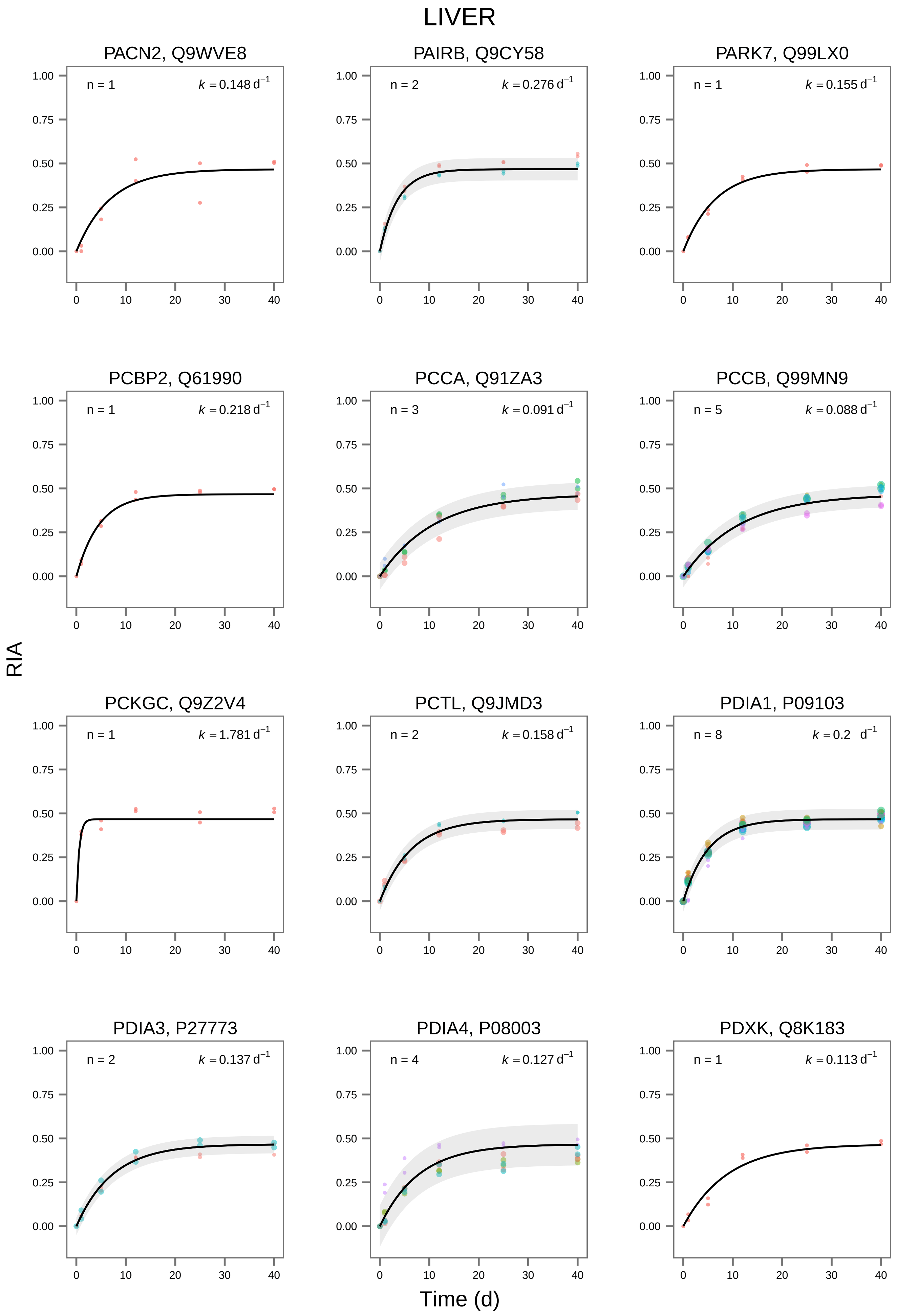
LIVER
PACN2, Q9WVE8
n = 1
k = 0.148 d−1
PAIRB, Q9CY58
n = 2
k = 0.276 d−1
PARK7, Q99LX0
n = 1
k = 0.155 d−1
PCBP2, Q61990
n = 1
k = 0.218 d−1
PCCA, Q91ZA3
n = 3
k = 0.091 d−1
PCCB, Q99MN9
n = 5
k = 0.088 d−1
PCKGC, Q9Z2V4
n = 1
k = 1.781 d−1
PCTL, Q9JMD3
n = 2
k = 0.158 d−1
PDIA1, P09103
n = 8
k = 0.2 d−1
PDIA3, P27773
n = 2
k = 0.137 d−1
PDIA4, P08003
n = 4
k = 0.127 d−1
PDXK, Q8K183
n = 1
k = 0.113 d−1
RIA
Time (d)
1.00
0.75
0.50
0.25
0.00
0
10
20
30
40

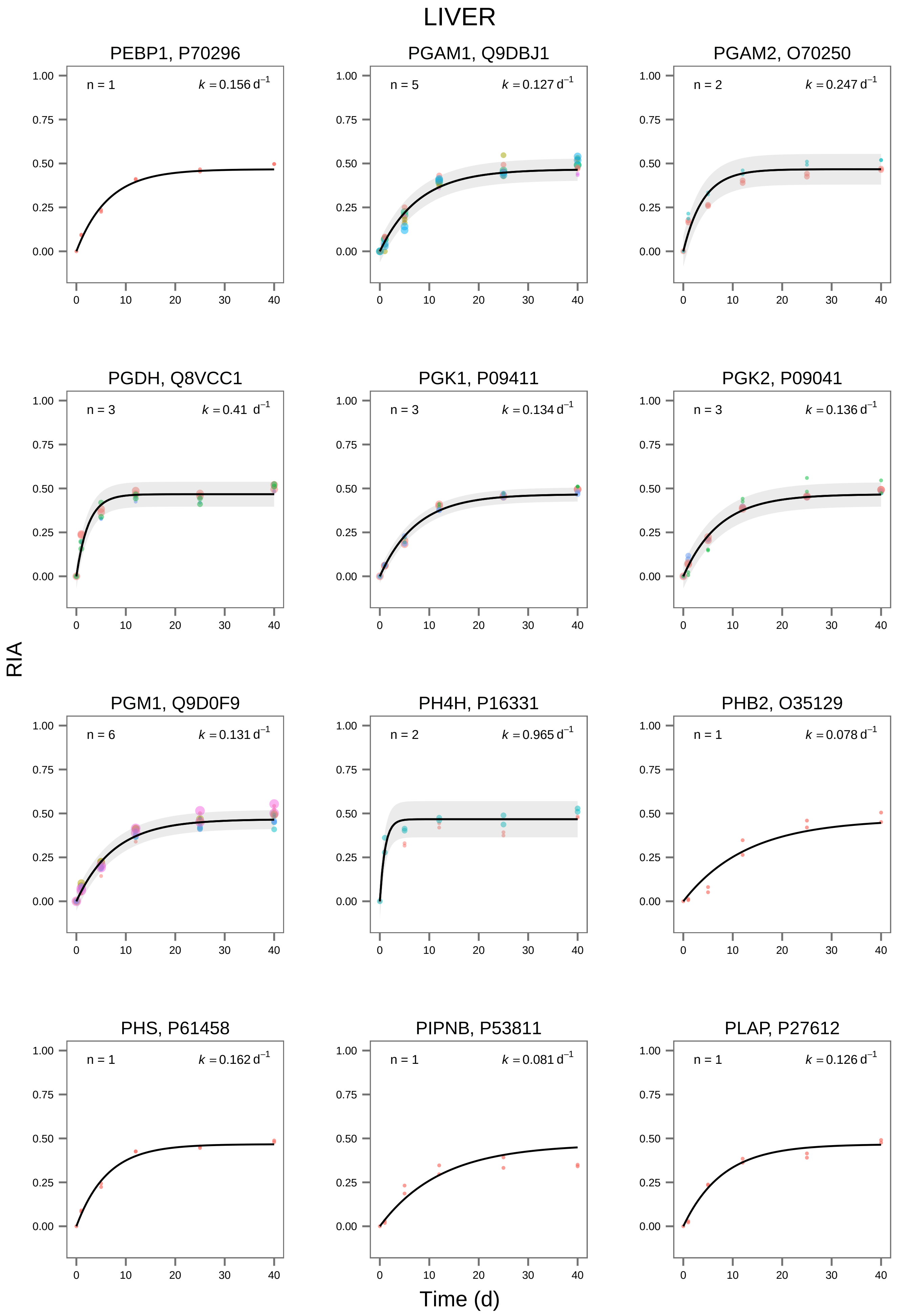

LIVER
PEBP1, P70296
n = 1
k = 0.156 d−1
PGAM1, Q9DBJ1
n = 5
k = 0.127 d−1
PGAM2, O70250
n = 2
k = 0.247 d−1
PGDH, Q8VCC1
n = 3
k = 0.41 d−1
PGK1, P09411
n = 3
k = 0.134 d−1
PGK2, P09041
n = 3
k = 0.136 d−1
PGM1, Q9D0F9
n = 6
k = 0.131 d−1
PH4H, P16331
n = 2
k = 0.965 d−1
PHB2, O35129
n = 1
k = 0.078 d−1
PHS, P61458
n = 1
k = 0.162 d−1
PIPNB, P53811
n = 1
k = 0.081 d−1
PLAP, P27612
n = 1
k = 0.126 d−1
RIA
Time (d)

LIVER

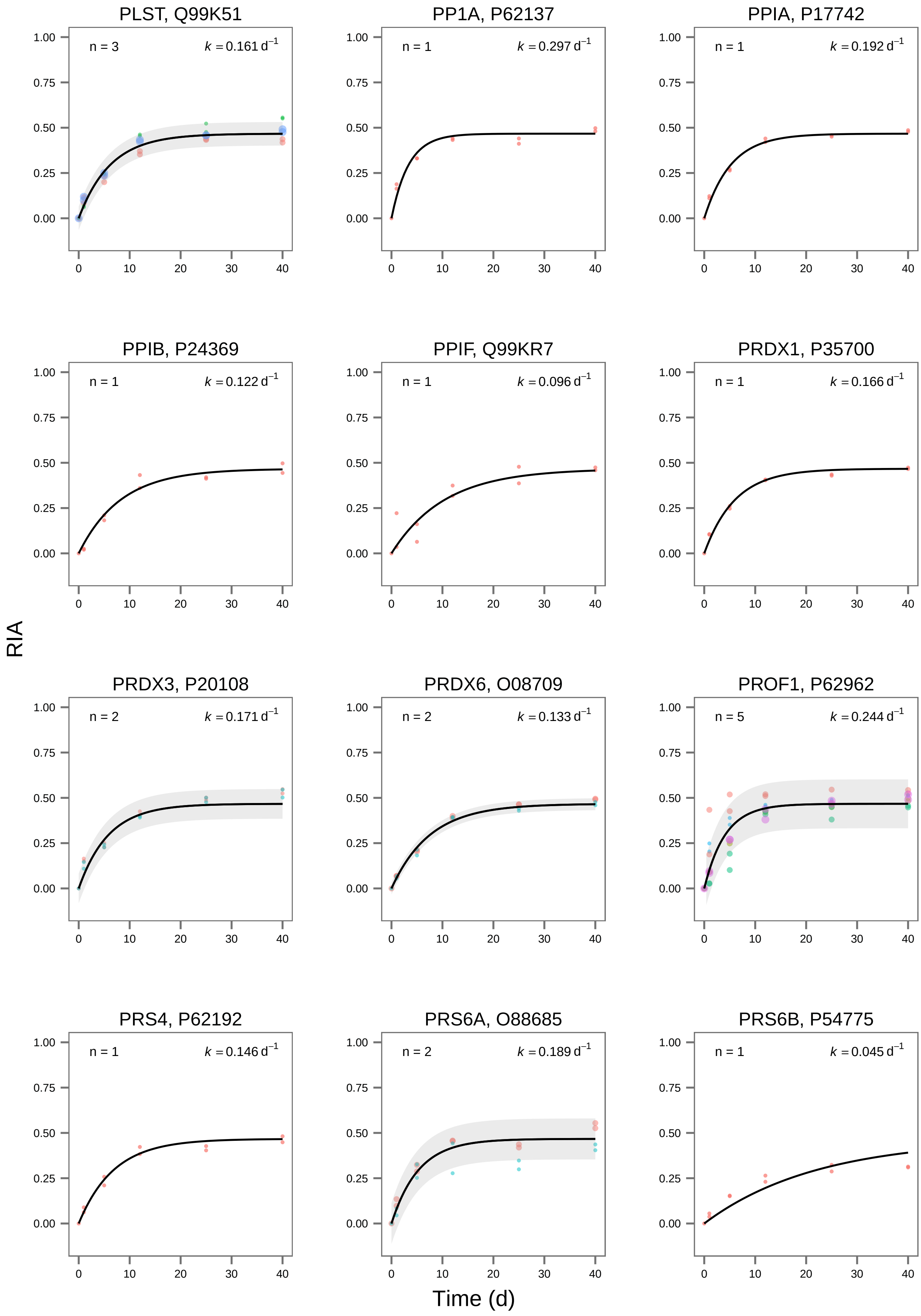

PLST, Q99K51
n = 3
k = 0.161 d⁻¹
PP1A, P62137
n = 1
k = 0.297 d⁻¹
PPIA, P17742
n = 1
k = 0.192 d⁻¹
PPIB, P24369
n = 1
k = 0.122 d⁻¹
PPIF, Q99KR7
n = 1
k = 0.096 d⁻¹
PRDX1, P35700
n = 1
k = 0.166 d⁻¹
PRDX3, P20108
n = 2
k = 0.171 d⁻¹
PRDX6, O08709
n = 2
k = 0.133 d⁻¹
PROF1, P62962
n = 5
k = 0.244 d⁻¹
PRS4, P62192
n = 1
k = 0.146 d⁻¹
PRS6A, O88685
n = 2
k = 0.189 d⁻¹
PRS6B, P54775
n = 1
k = 0.045 d⁻¹
RIA
Time (d)
1.00
0.75
0.50
0.25
0.00
0
10
20
30
40

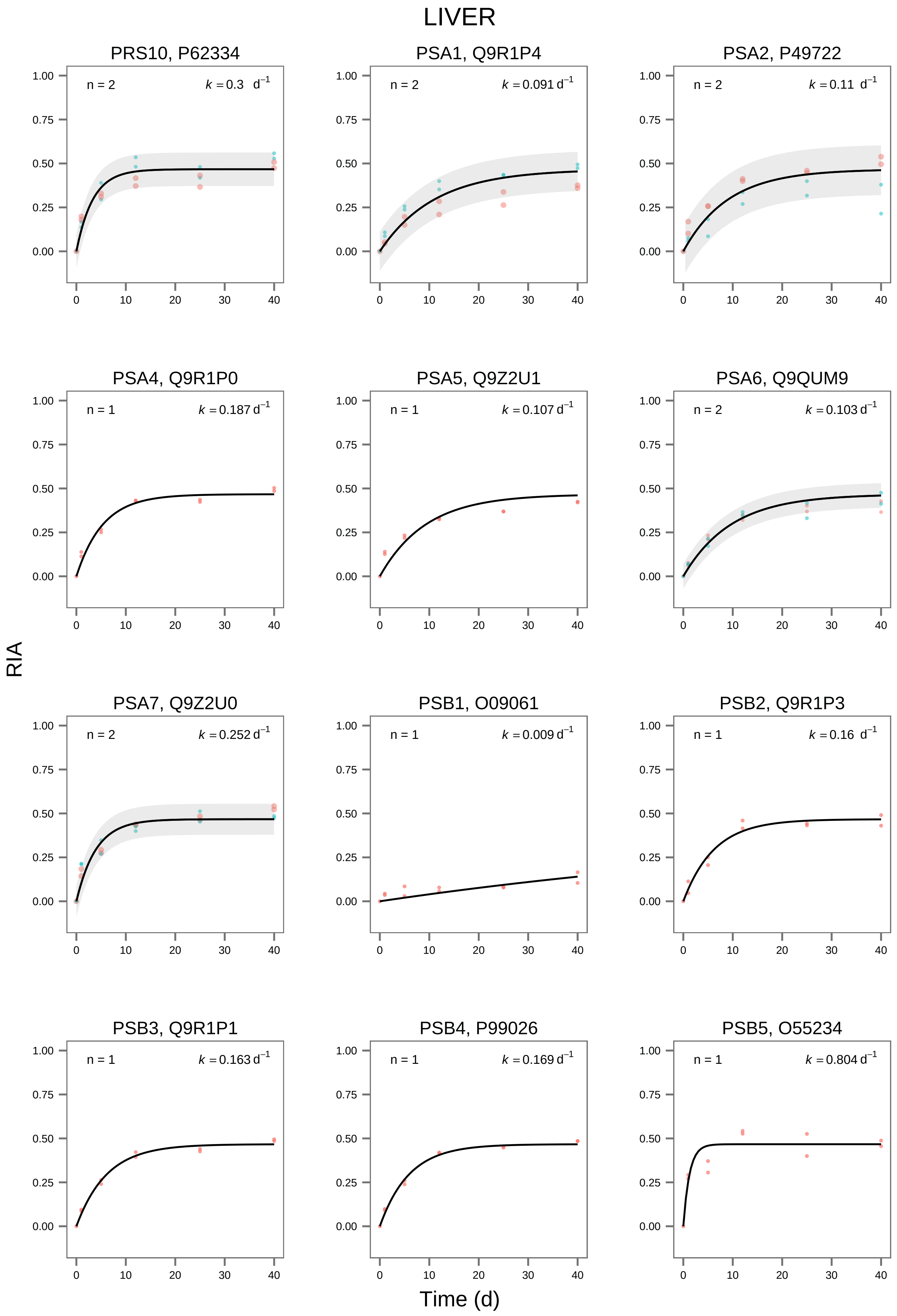
LIVER
PRS10, P62334
n = 2
k = 0.3 d−1
PSA1, Q9R1P4
n = 2
k = 0.091 d−1
PSA2, P49722
n = 2
k = 0.11 d−1
PSA4, Q9R1P0
n = 1
k = 0.187 d−1
PSA5, Q9Z2U1
n = 1
k = 0.107 d−1
PSA6, Q9QUM9
n = 2
k = 0.103 d−1
PSA7, Q9Z2U0
n = 2
k = 0.252 d−1
PSB1, O09061
n = 1
k = 0.009 d−1
PSB2, Q9R1P3
n = 1
k = 0.16 d−1
PSB3, Q9R1P1
n = 1
k = 0.163 d−1
PSB4, P99026
n = 1
k = 0.169 d−1
PSB5, O55234
n = 1
k = 0.804 d−1
RIA
Time (d)

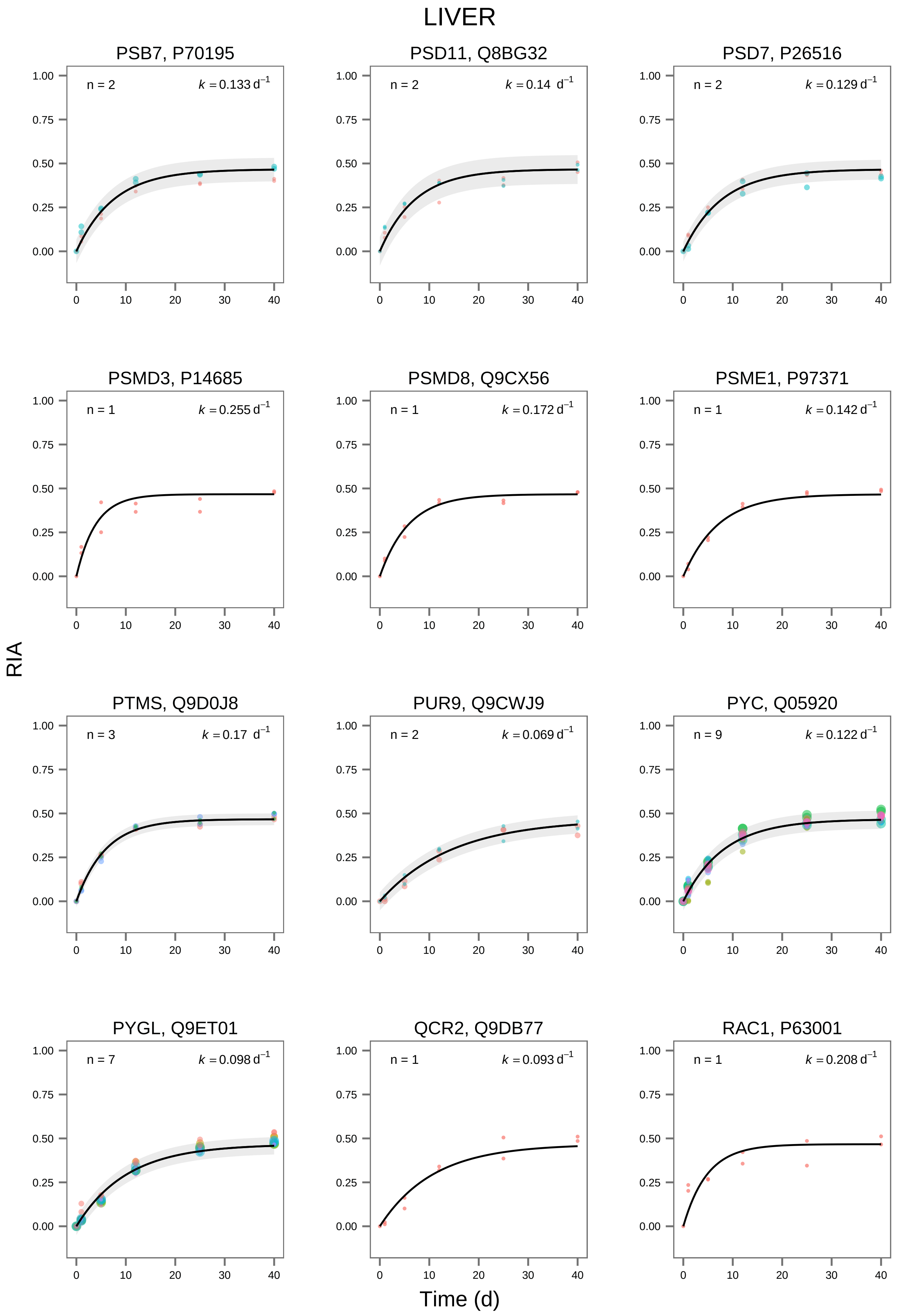
LIVER
PSB7, P70195
n = 2
k = 0.133 d−1
PSD11, Q8BG32
n = 2
k = 0.14 d−1
PSD7, P26516
n = 2
k = 0.129 d−1
PSMD3, P14685
n = 1
k = 0.255 d−1
PSMD8, Q9CX56
n = 1
k = 0.172 d−1
PSME1, P97371
n = 1
k = 0.142 d−1
PTMS, Q9D0J8
n = 3
k = 0.17 d−1
PUR9, Q9CWJ9
n = 2
k = 0.069 d−1
PYC, Q05920
n = 9
k = 0.122 d−1
PYGL, Q9ET01
n = 7
k = 0.098 d−1
QCR2, Q9DB77
n = 1
k = 0.093 d−1
RAC1, P63001
n = 1
k = 0.208 d−1
RIA
Time (d)

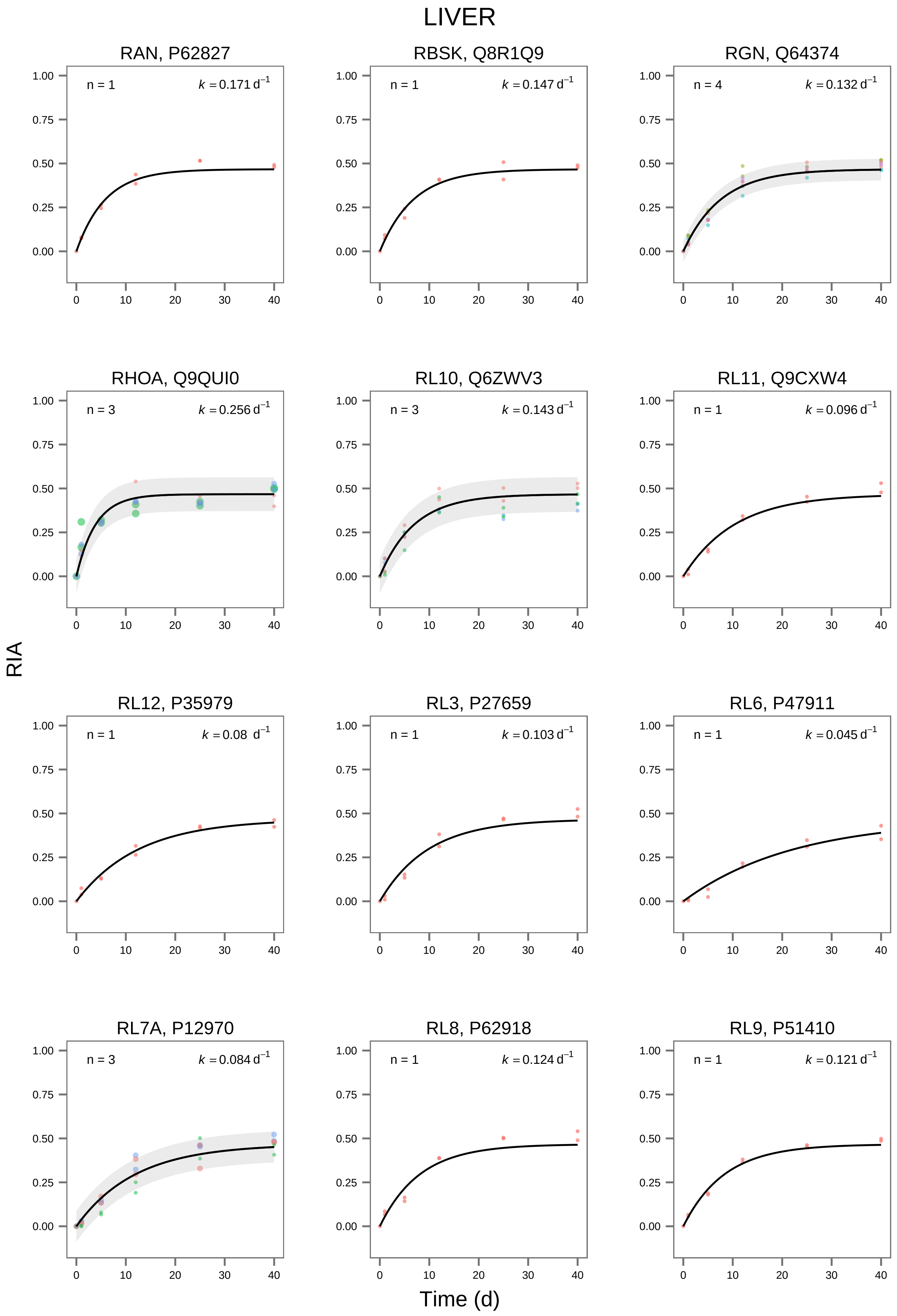
LIVER
RAN, P62827
n = 1
k = 0.171 d−1
RBSK, Q8R1Q9
n = 1
k = 0.147 d−1
RGN, Q64374
n = 4
k = 0.132 d−1
RHOA, Q9QUI0
n = 3
k = 0.256 d−1
RL10, Q6ZWV3
n = 3
k = 0.143 d−1
RL11, Q9CXW4
n = 1
k = 0.096 d−1
RL12, P35979
n = 1
k = 0.08 d−1
RL3, P27659
n = 1
k = 0.103 d−1
RL6, P47911
n = 1
k = 0.045 d−1
RL7A, P12970
n = 3
k = 0.084 d−1
RL8, P62918
n = 1
k = 0.124 d−1
RL9, P51410
n = 1
k = 0.121 d−1
RIA
Time (d)
0.00
0.25
0.50
0.75
1.00
0
10
20
30
40

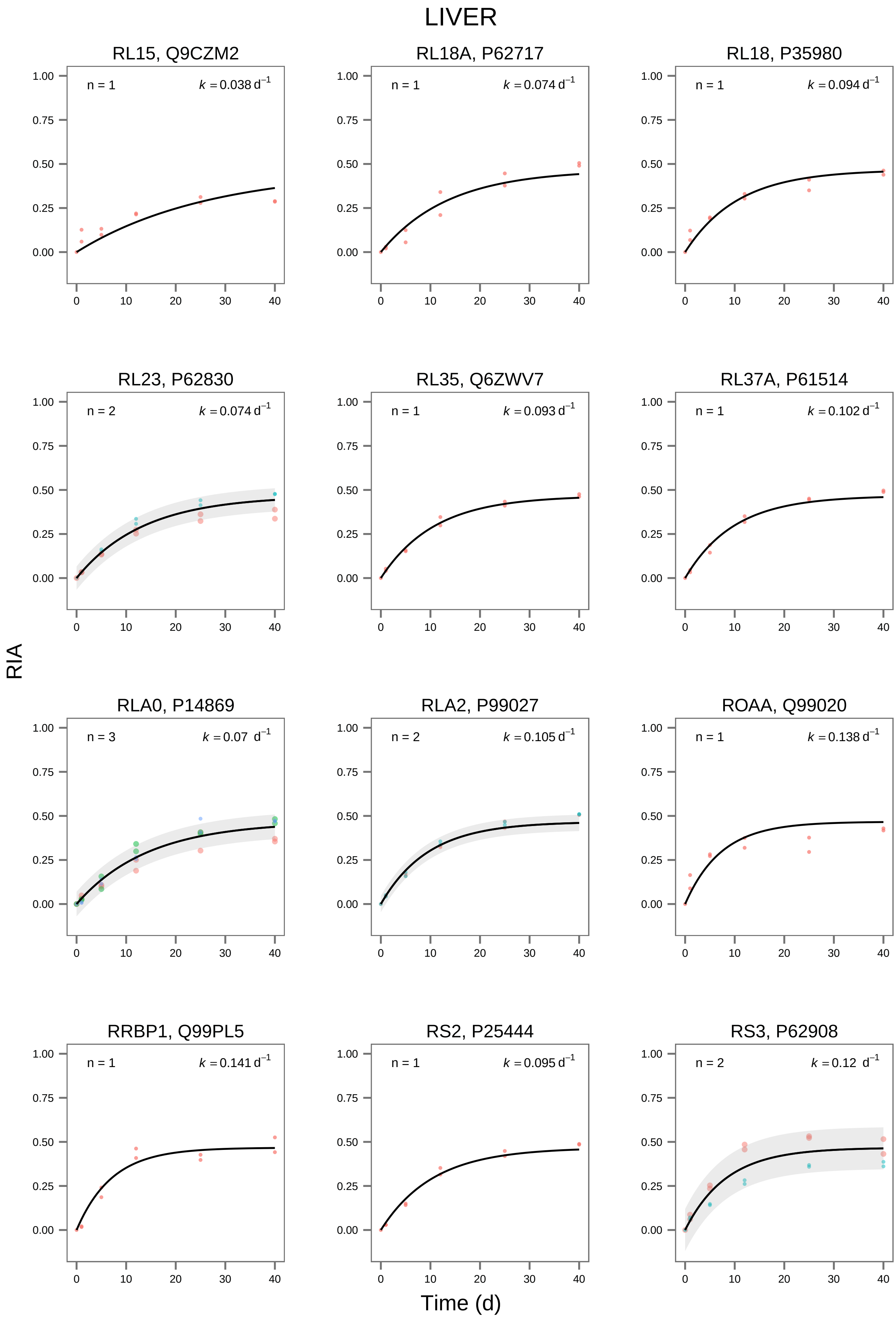
LIVER
RL15, Q9CZM2
n = 1
k = 0.038 d−1
RL18A, P62717
n = 1
k = 0.074 d−1
RL18, P35980
n = 1
k = 0.094 d−1
RL23, P62830
n = 2
k = 0.074 d−1
RL35, Q6ZWV7
n = 1
k = 0.093 d−1
RL37A, P61514
n = 1
k = 0.102 d−1
RLA0, P14869
n = 3
k = 0.07 d−1
RLA2, P99027
n = 2
k = 0.105 d−1
ROAA, Q99020
n = 1
k = 0.138 d−1
RRBP1, Q99PL5
n = 1
k = 0.141 d−1
RS2, P25444
n = 1
k = 0.095 d−1
RS3, P62908
n = 2
k = 0.12 d−1
RIA
Time (d)

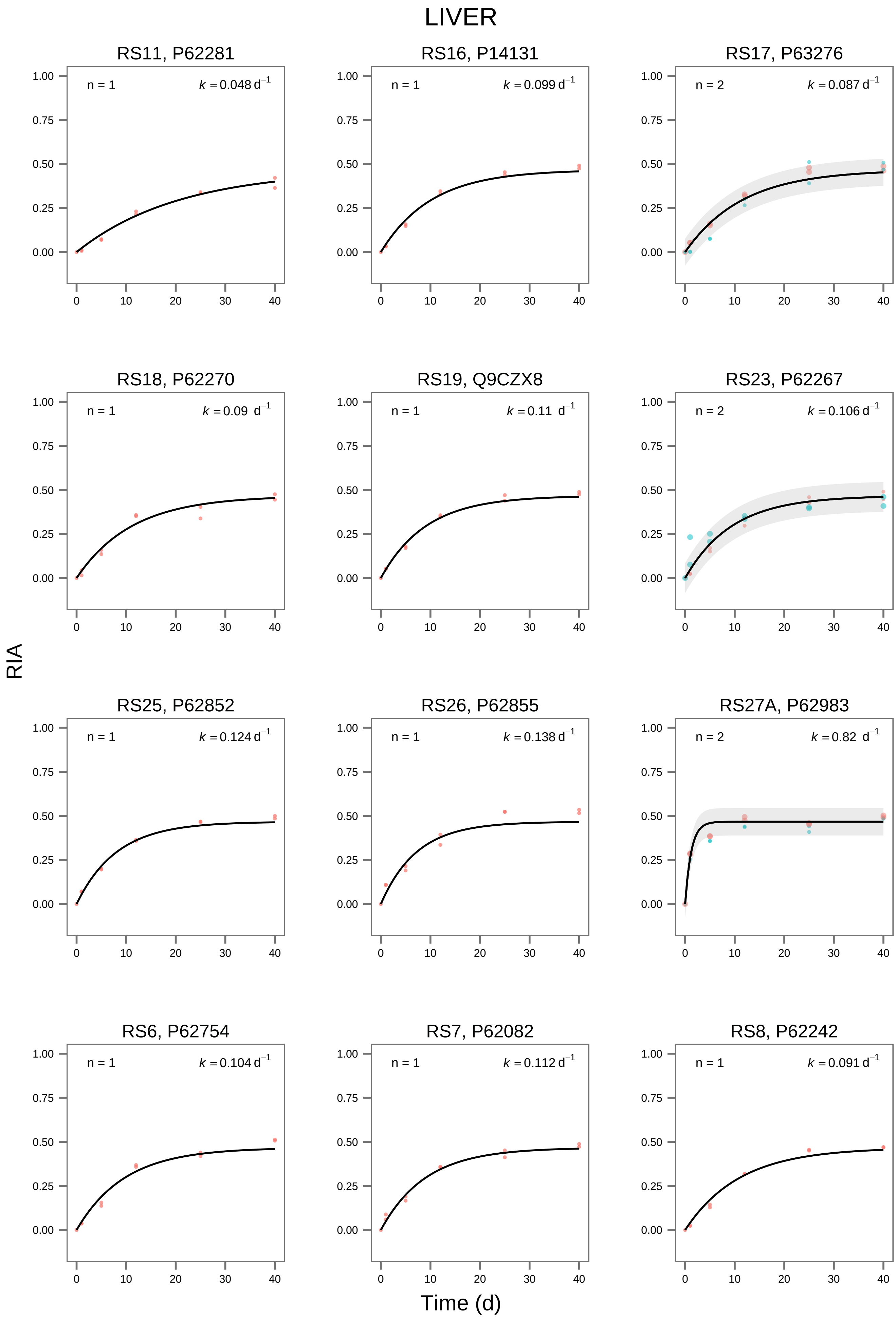
LIVER
RS11, P62281
n = 1
k = 0.048 d⁻¹
RS16, P14131
n = 1
k = 0.099 d⁻¹
RS17, P63276
n = 2
k = 0.087 d⁻¹
RS18, P62270
n = 1
k = 0.09 d⁻¹
RS19, Q9CZX8
n = 1
k = 0.11 d⁻¹
RS23, P62267
n = 2
k = 0.106 d⁻¹
RS25, P62852
n = 1
k = 0.124 d⁻¹
RS26, P62855
n = 1
k = 0.138 d⁻¹
RS27A, P62983
n = 2
k = 0.82 d⁻¹
RS6, P62754
n = 1
k = 0.104 d⁻¹
RS7, P62082
n = 1
k = 0.112 d⁻¹
RS8, P62242
n = 1
k = 0.091 d⁻¹
RIA
Time (d)
1.00
0.75
0.50
0.25
0.00
0
10
20
30
40

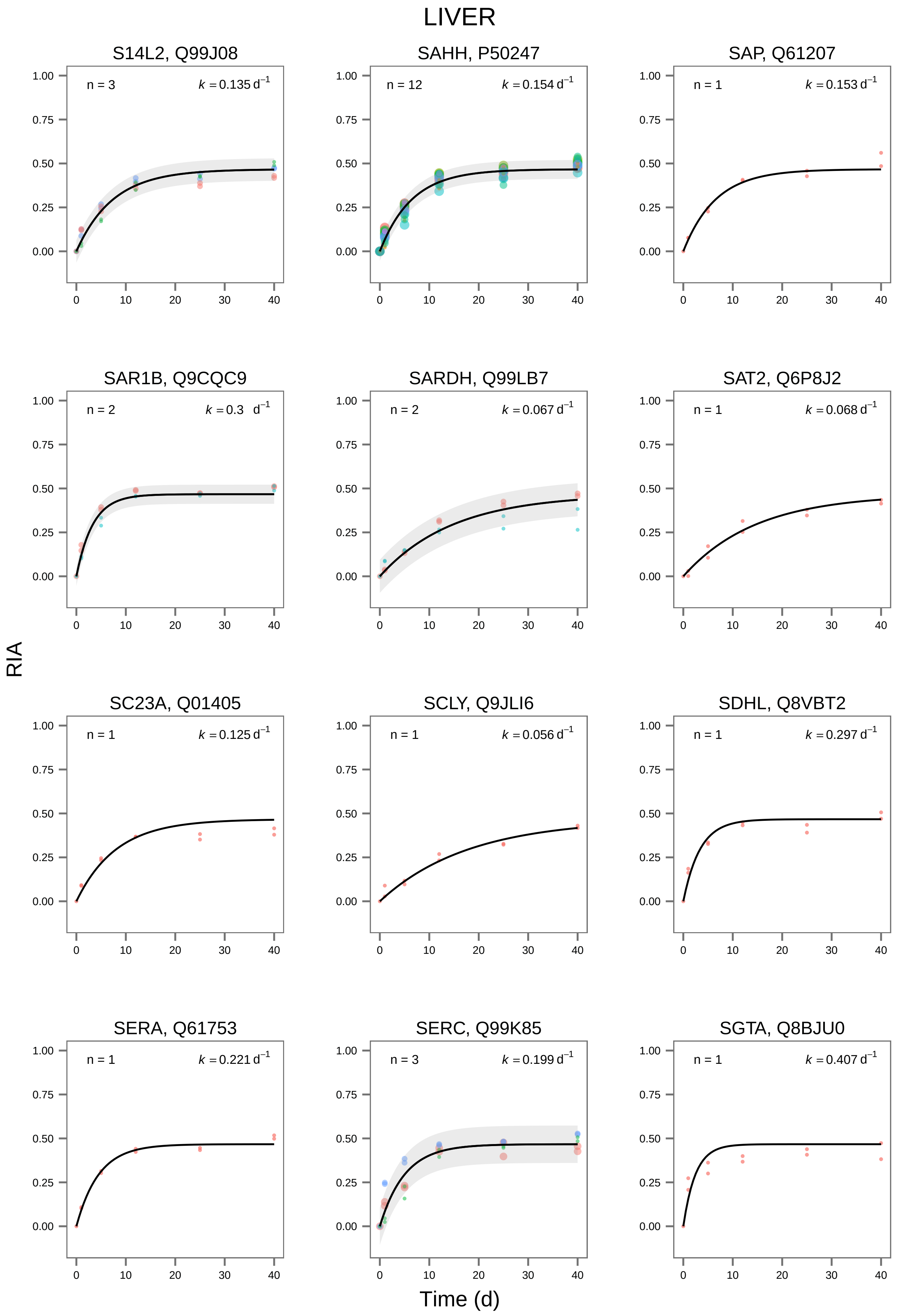
LIVER
S14L2, Q99J08
n = 3
k = 0.135 d−1
SAHH, P50247
n = 12
k = 0.154 d−1
SAP, Q61207
n = 1
k = 0.153 d−1
SAR1B, Q9CQC9
n = 2
k = 0.3 d−1
SARDH, Q99LB7
n = 2
k = 0.067 d−1
SAT2, Q6P8J2
n = 1
k = 0.068 d−1
SC23A, Q01405
n = 1
k = 0.125 d−1
SCLY, Q9JLI6
n = 1
k = 0.056 d−1
SDHL, Q8VBT2
n = 1
k = 0.297 d−1
SERA, Q61753
n = 1
k = 0.221 d−1
SERC, Q99K85
n = 3
k = 0.199 d−1
SGTA, Q8BJU0
n = 1
k = 0.407 d−1
RIA
Time (d)

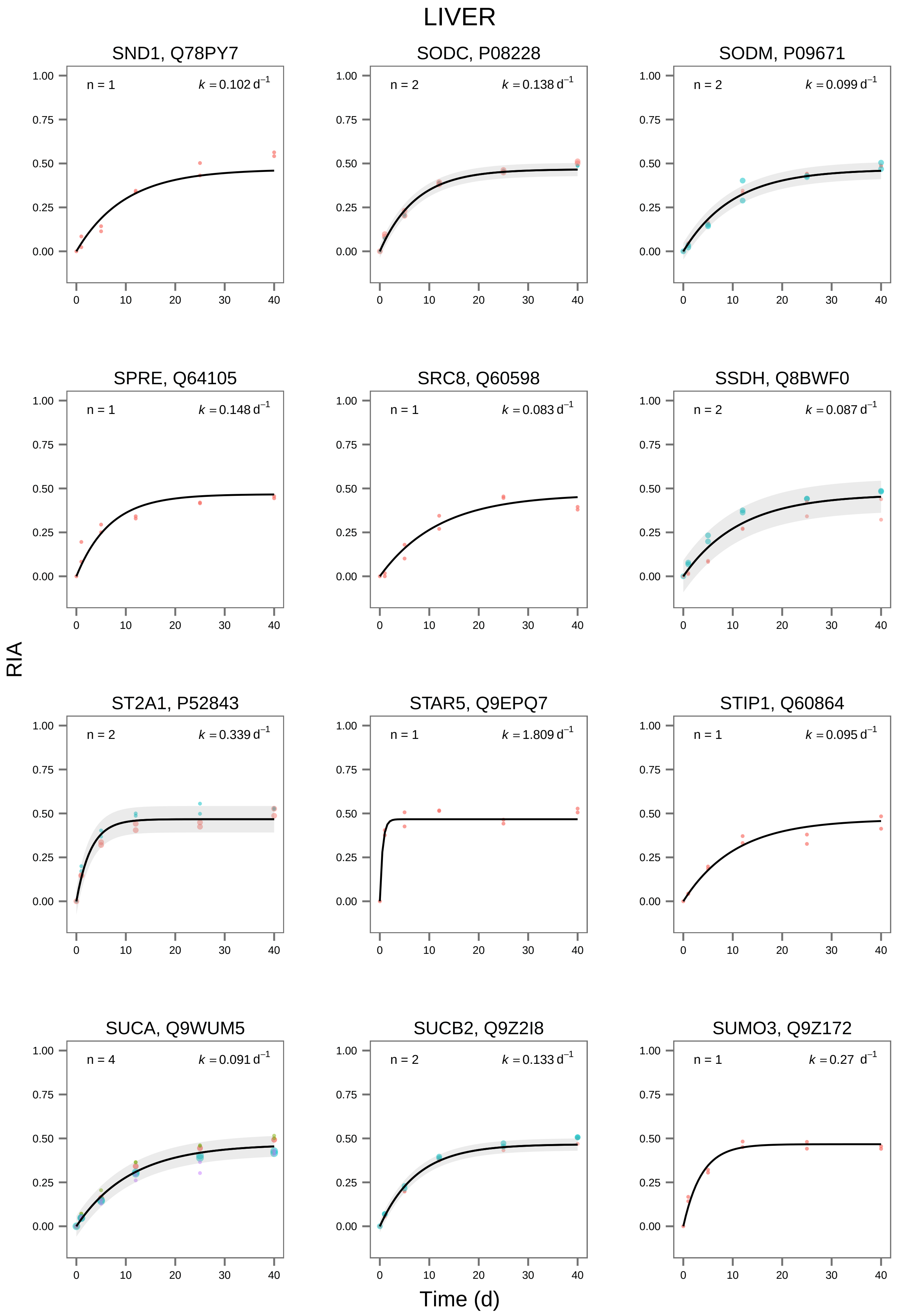
LIVER
SND1, Q78PY7
n = 1
k = 0.102 d−1
SODC, P08228
n = 2
k = 0.138 d−1
SODM, P09671
n = 2
k = 0.099 d−1
SPRE, Q64105
n = 1
k = 0.148 d−1
SRC8, Q60598
n = 1
k = 0.083 d−1
SSDH, Q8BWF0
n = 2
k = 0.087 d−1
ST2A1, P52843
n = 2
k = 0.339 d−1
STAR5, Q9EPQ7
n = 1
k = 1.809 d−1
STIP1, Q60864
n = 1
k = 0.095 d−1
SUCA, Q9WUM5
n = 4
k = 0.091 d−1
SUCB2, Q9Z2I8
n = 2
k = 0.133 d−1
SUMO3, Q9Z172
n = 1
k = 0.27 d−1
RIA
Time (d)
1.00
0.75
0.50
0.25
0.00
0
10
20
30
40

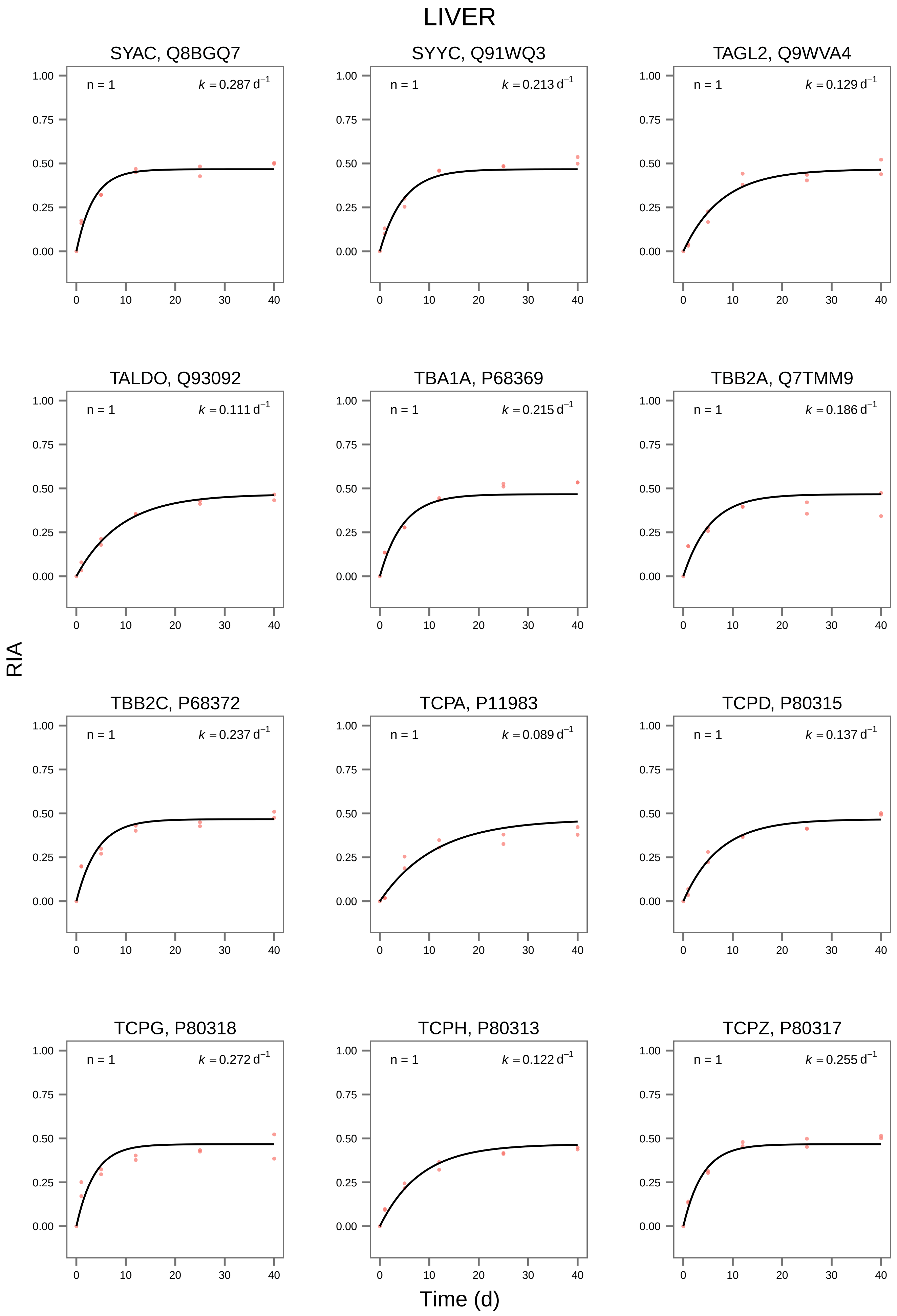
LIVER
SYAC, Q8BGQ7
n = 1
k = 0.287 d^-1
SYYC, Q91WQ3
n = 1
k = 0.213 d^-1
TAGL2, Q9WVA4
n = 1
k = 0.129 d^-1
TALDO, Q93092
n = 1
k = 0.111 d^-1
TBA1A, P68369
n = 1
k = 0.215 d^-1
TBB2A, Q7TMM9
n = 1
k = 0.186 d^-1
TBB2C, P68372
n = 1
k = 0.237 d^-1
TCPA, P11983
n = 1
k = 0.089 d^-1
TCPD, P80315
n = 1
k = 0.137 d^-1
TCPG, P80318
n = 1
k = 0.272 d^-1
TCPH, P80313
n = 1
k = 0.122 d^-1
TCPZ, P80317
n = 1
k = 0.255 d^-1
RIA
Time (d)

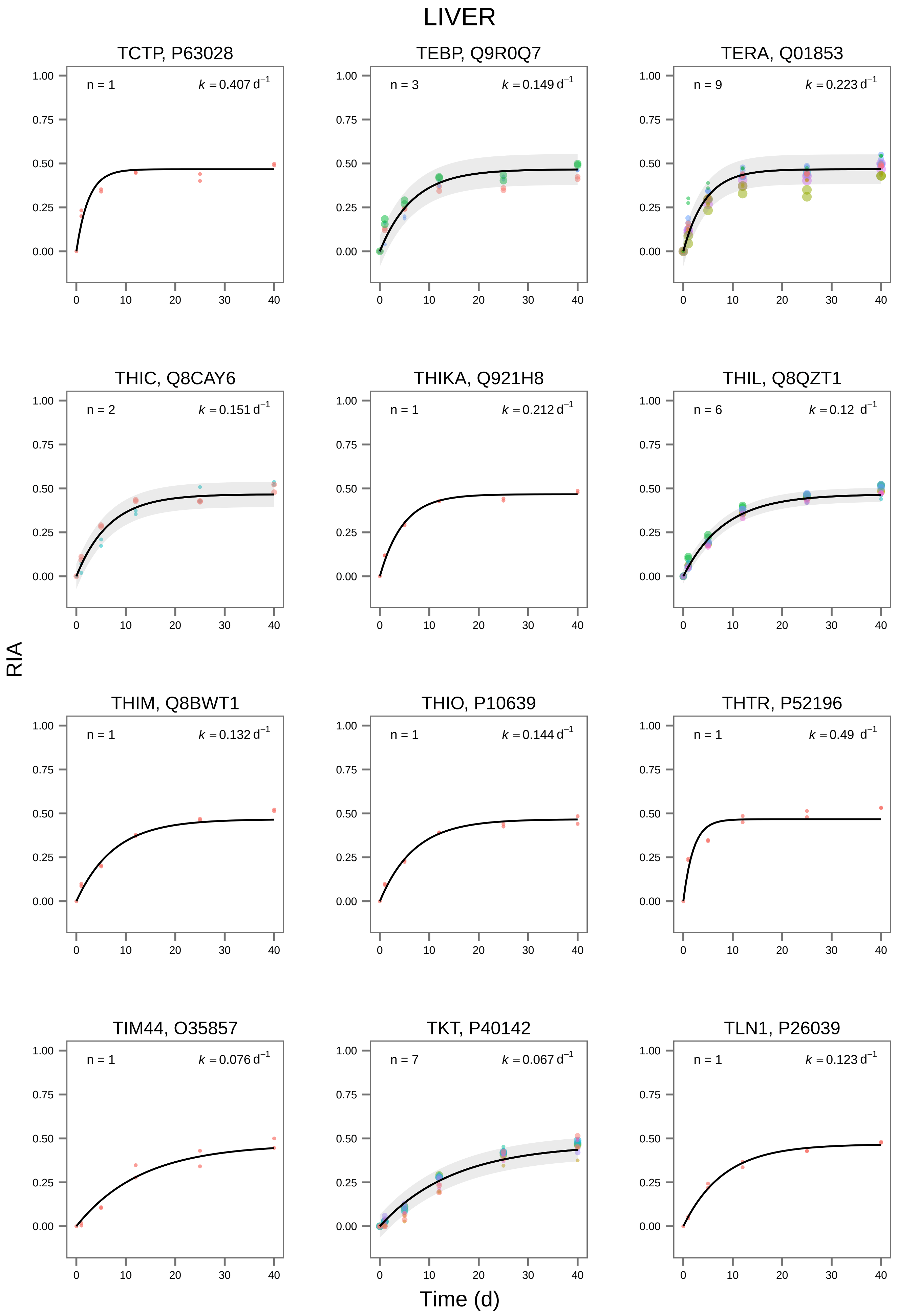
LIVER
TCTP, P63028
n = 1
k = 0.407 d−1
TEBP, Q9R0Q7
n = 3
k = 0.149 d−1
TERA, Q01853
n = 9
k = 0.223 d−1
THIC, Q8CAY6
n = 2
k = 0.151 d−1
THIKA, Q921H8
n = 1
k = 0.212 d−1
THIL, Q8QZT1
n = 6
k = 0.12 d−1
THIM, Q8BWT1
n = 1
k = 0.132 d−1
THIO, P10639
n = 1
k = 0.144 d−1
THTR, P52196
n = 1
k = 0.49 d−1
TIM44, O35857
n = 1
k = 0.076 d−1
TKT, P40142
n = 7
k = 0.067 d−1
TLN1, P26039
n = 1
k = 0.123 d−1
RIA
Time (d)
0.00
0.25
0.50
0.75
1.00
0
10
20
30
40

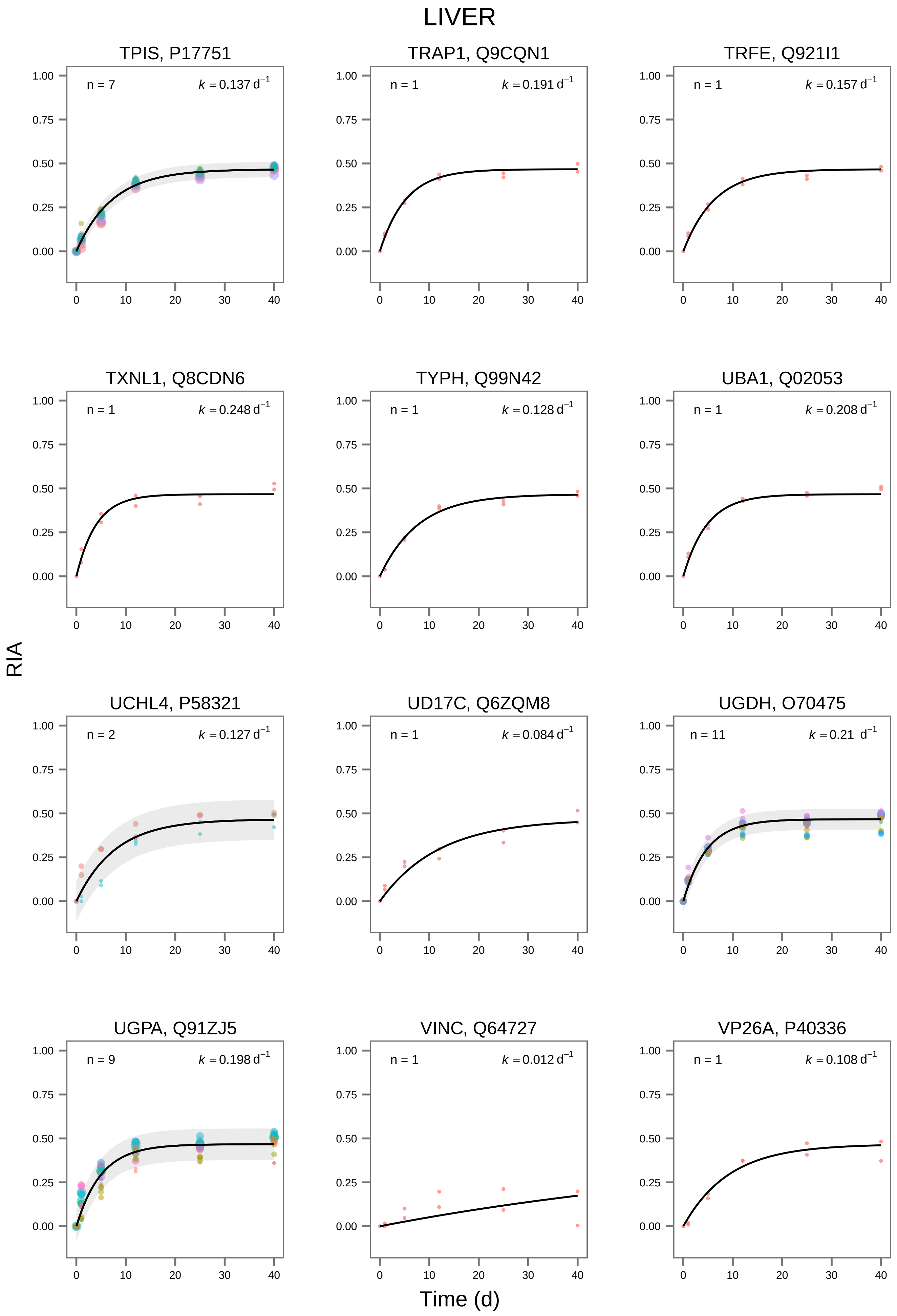
LIVER
TPIS, P17751
n = 7
k = 0.137 d⁻¹
TRAP1, Q9CQN1
n = 1
k = 0.191 d⁻¹
TRFE, Q921I1
n = 1
k = 0.157 d⁻¹
TXNL1, Q8CDN6
n = 1
k = 0.248 d⁻¹
TYPH, Q99N42
n = 1
k = 0.128 d⁻¹
UBA1, Q02053
n = 1
k = 0.208 d⁻¹
UCHL4, P58321
n = 2
k = 0.127 d⁻¹
UD17C, Q6ZQM8
n = 1
k = 0.084 d⁻¹
UGDH, O70475
n = 11
k = 0.21 d⁻¹
UGPA, Q91ZJ5
n = 9
k = 0.198 d⁻¹
VINC, Q64727
n = 1
k = 0.012 d⁻¹
VP26A, P40336
n = 1
k = 0.108 d⁻¹
RIA
Time (d)

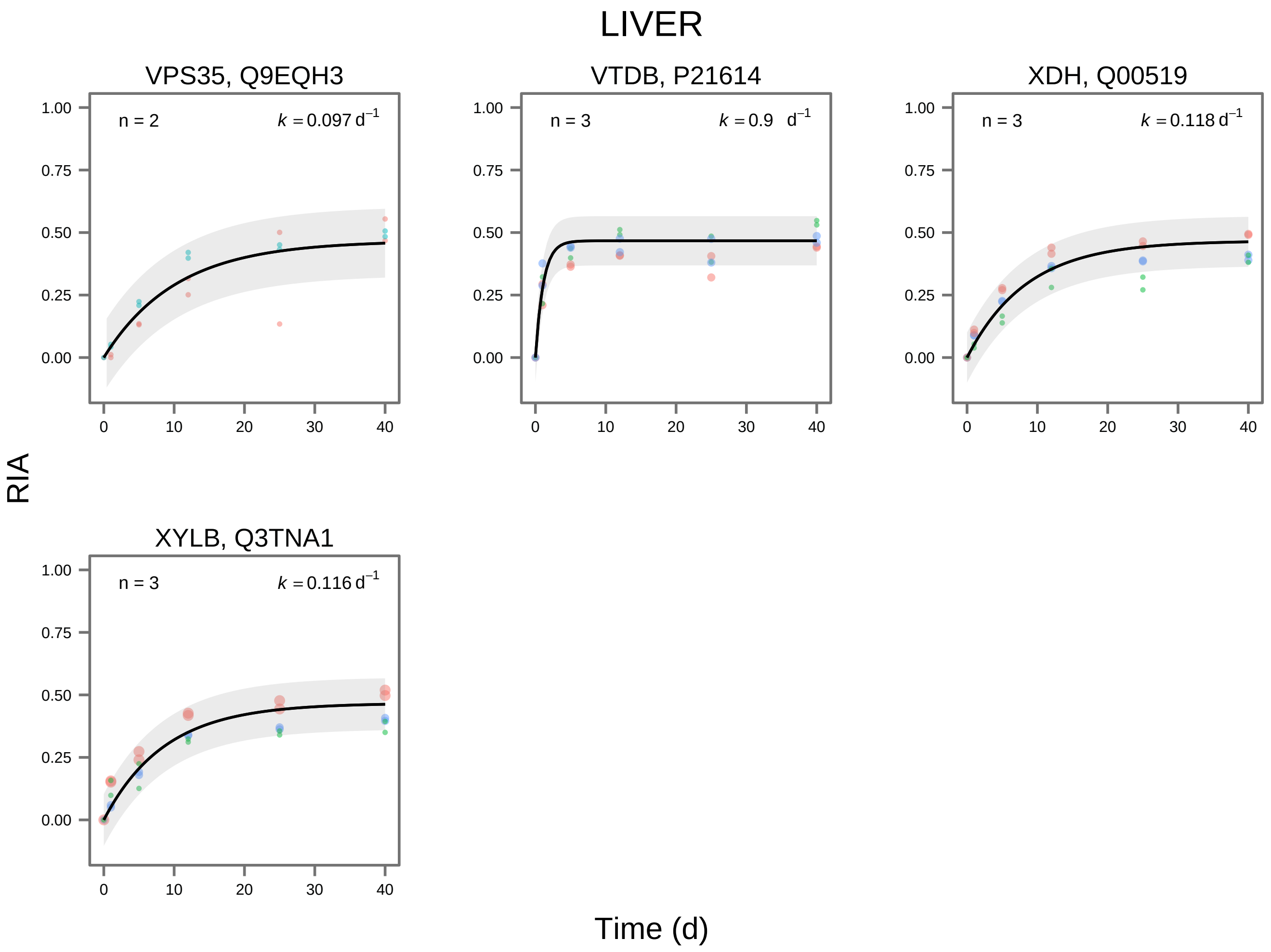
LIVER
VPS35, Q9EQH3
n = 2
k = 0.097 d−1
VTDB, P21614
n = 3
k = 0.9 d−1
XDH, Q00519
n = 3
k = 0.118 d−1
XYLB, Q3TNA1
n = 3
k = 0.116 d−1
RIA
1.00
0.75
0.50
0.25
0.00
0
10
20
30
40
Time (d)

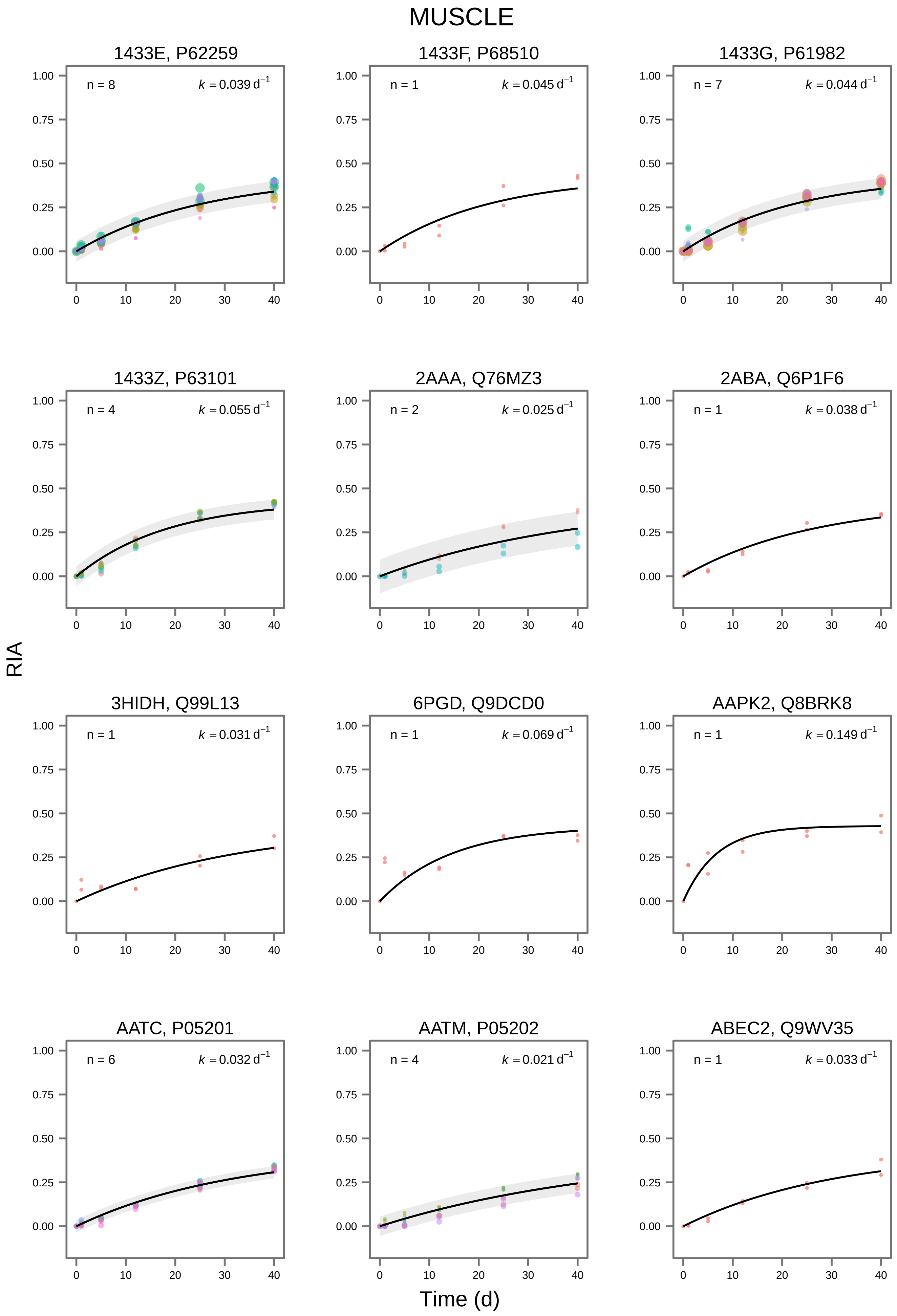
MUSCLE
1433E, P62259
n = 8
k = 0.039 d−1
1433F, P68510
n = 1
k = 0.045 d−1
1433G, P61982
n = 7
k = 0.044 d−1
1433Z, P63101
n = 4
k = 0.055 d−1
2AAA, Q76MZ3
n = 2
k = 0.025 d−1
2ABA, Q6P1F6
n = 1
k = 0.038 d−1
3HIDH, Q99L13
n = 1
k = 0.031 d−1
6PGD, Q9DCD0
n = 1
k = 0.069 d−1
AAPK2, Q8BRK8
n = 1
k = 0.149 d−1
AATC, P05201
n = 6
k = 0.032 d−1
AATM, P05202
n = 4
k = 0.021 d−1
ABEC2, Q9WV35
n = 1
k = 0.033 d−1
RIA
Time (d)

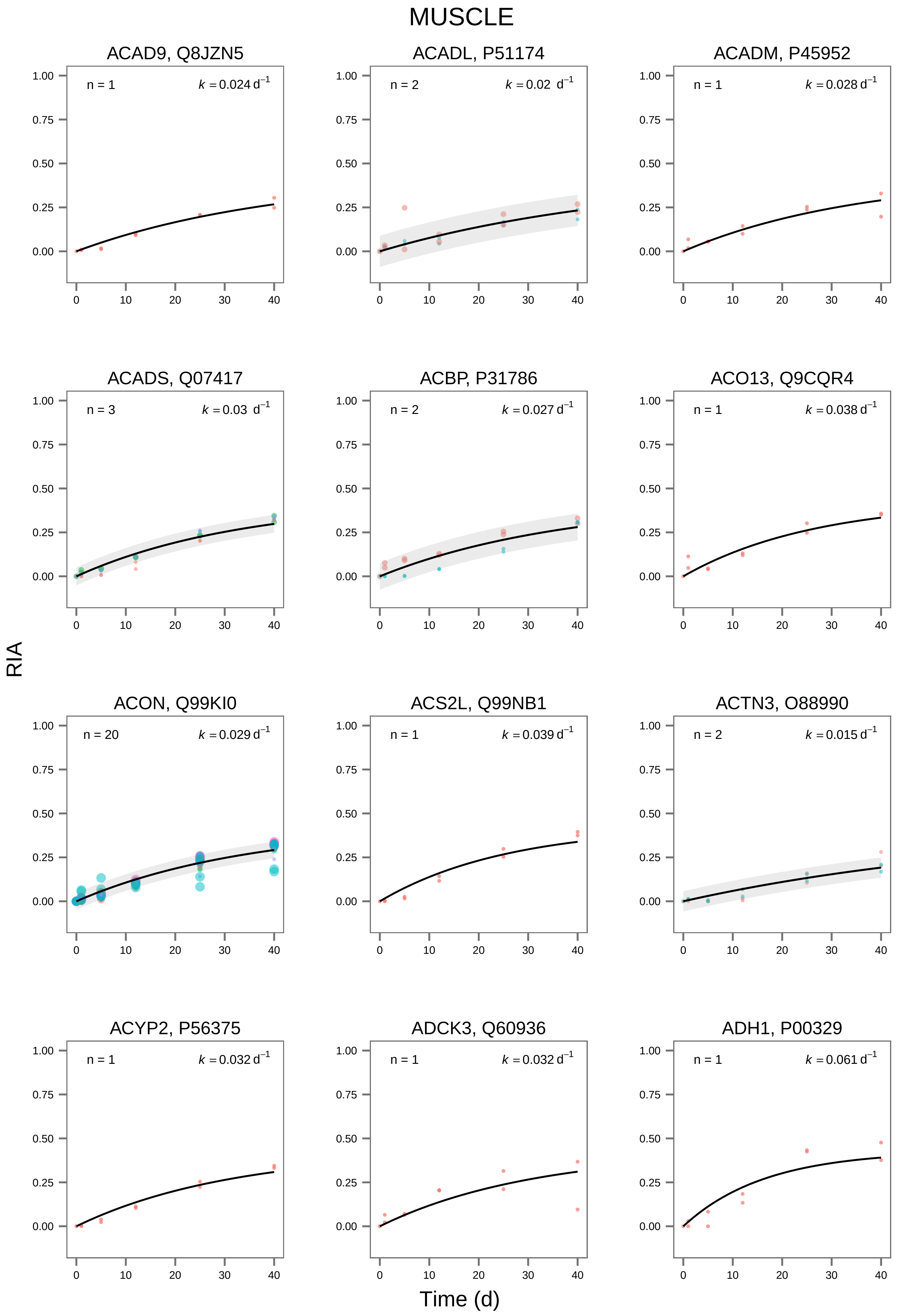
MUSCLE
ACAD9, Q8JZN5
n = 1
k = 0.024 d−1
ACADL, P51174
n = 2
k = 0.02 d−1
ACADM, P45952
n = 1
k = 0.028 d−1
ACADS, Q07417
n = 3
k = 0.03 d−1
ACBP, P31786
n = 2
k = 0.027 d−1
ACO13, Q9CQR4
n = 1
k = 0.038 d−1
ACON, Q99KI0
n = 20
k = 0.029 d−1
ACS2L, Q99NB1
n = 1
k = 0.039 d−1
ACTN3, O88990
n = 2
k = 0.015 d−1
ACYP2, P56375
n = 1
k = 0.032 d−1
ADCK3, Q60936
n = 1
k = 0.032 d−1
ADH1, P00329
n = 1
k = 0.061 d−1
RIA
Time (d)

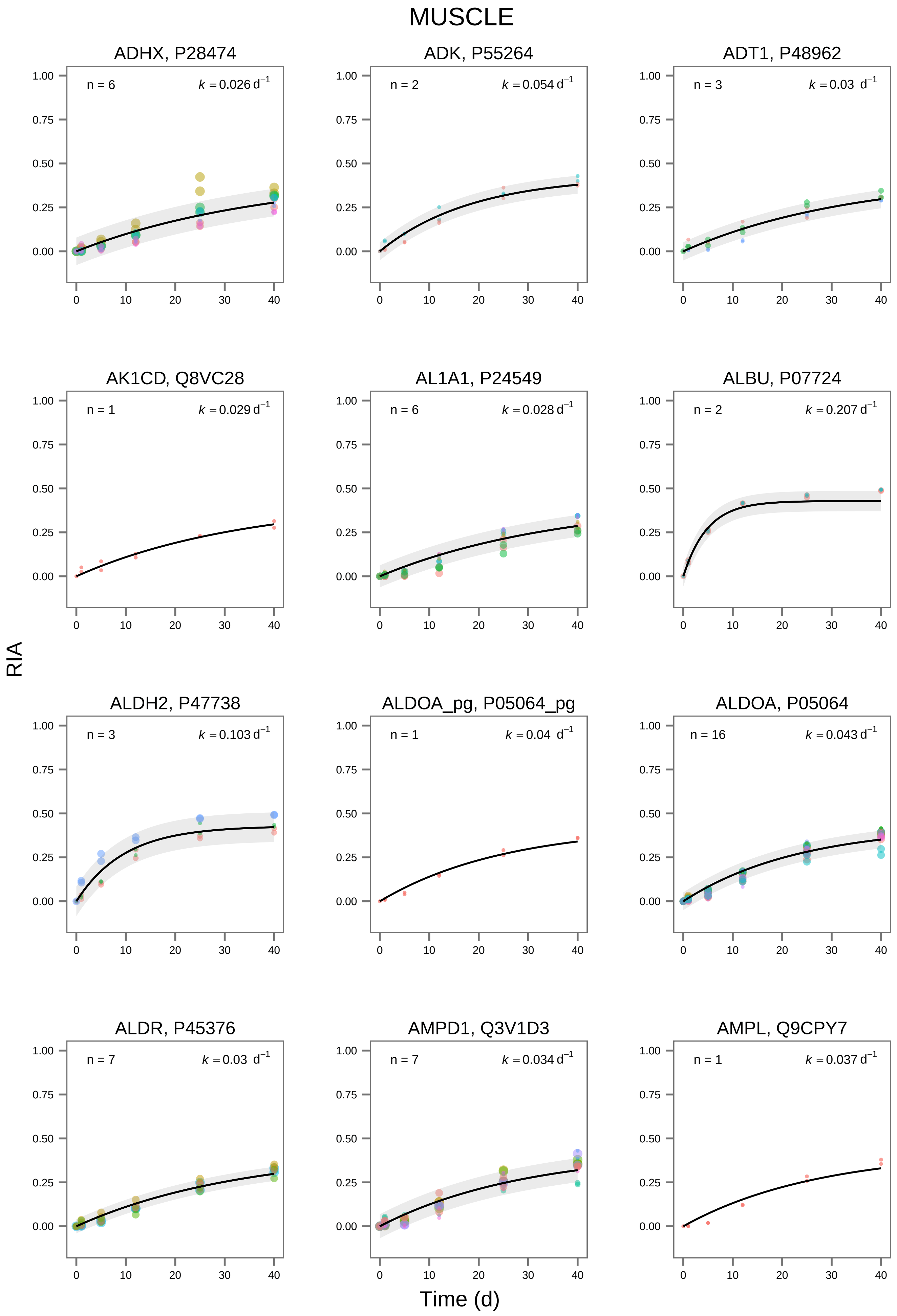
MUSCLE
ADHX, P28474
n = 6
k = 0.026 d−1
ADK, P55264
n = 2
k = 0.054 d−1
ADT1, P48962
n = 3
k = 0.03 d−1
AK1CD, Q8VC28
n = 1
k = 0.029 d−1
AL1A1, P24549
n = 6
k = 0.028 d−1
ALBU, P07724
n = 2
k = 0.207 d−1
ALDH2, P47738
n = 3
k = 0.103 d−1
ALDOA_pg, P05064_pg
n = 1
k = 0.04 d−1
ALDOA, P05064
n = 16
k = 0.043 d−1
ALDR, P45376
n = 7
k = 0.03 d−1
AMPD1, Q3V1D3
n = 7
k = 0.034 d−1
AMPL, Q9CPY7
n = 1
k = 0.037 d−1
RIA
Time (d)

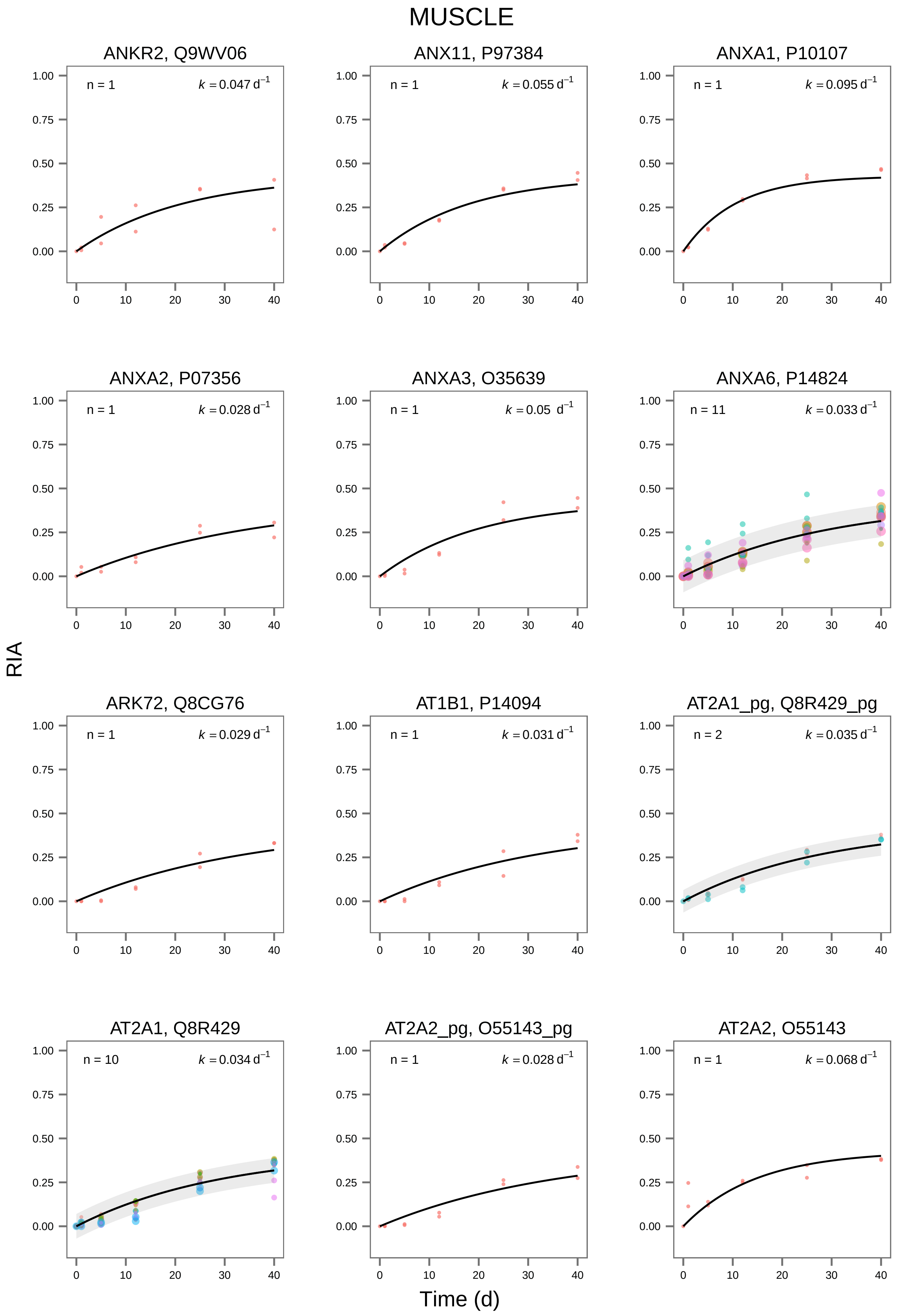
MUSCLE
ANKR2, Q9WV06
n = 1
k = 0.047 d−1
ANX11, P97384
n = 1
k = 0.055 d−1
ANXA1, P10107
n = 1
k = 0.095 d−1
ANXA2, P07356
n = 1
k = 0.028 d−1
ANXA3, O35639
n = 1
k = 0.05 d−1
ANXA6, P14824
n = 11
k = 0.033 d−1
ARK72, Q8CG76
n = 1
k = 0.029 d−1
AT1B1, P14094
n = 1
k = 0.031 d−1
AT2A1_pg, Q8R429_pg
n = 2
k = 0.035 d−1
AT2A1, Q8R429
n = 10
k = 0.034 d−1
AT2A2_pg, O55143_pg
n = 1
k = 0.028 d−1
AT2A2, O55143
n = 1
k = 0.068 d−1
RIA
Time (d)
1.00
0.75
0.50
0.25
0.00
0
10
20
30
40

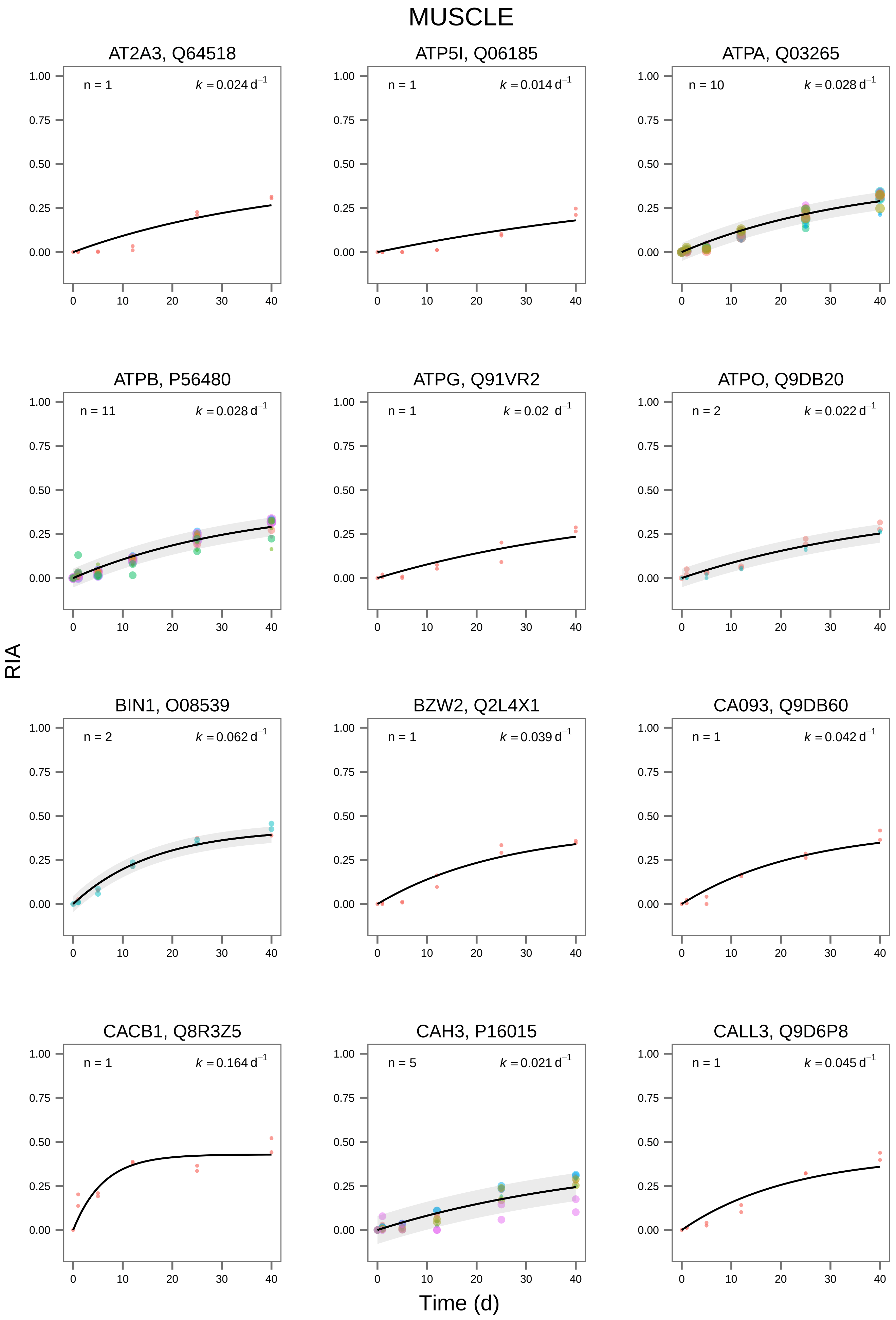
MUSCLE
AT2A3, Q64518
n = 1
k = 0.024 d−1
ATP5I, Q06185
n = 1
k = 0.014 d−1
ATPA, Q03265
n = 10
k = 0.028 d−1
ATPB, P56480
n = 11
k = 0.028 d−1
ATPG, Q91VR2
n = 1
k = 0.02 d−1
ATPO, Q9DB20
n = 2
k = 0.022 d−1
BIN1, O08539
n = 2
k = 0.062 d−1
BZW2, Q2L4X1
n = 1
k = 0.039 d−1
CA093, Q9DB60
n = 1
k = 0.042 d−1
CACB1, Q8R3Z5
n = 1
k = 0.164 d−1
CAH3, P16015
n = 5
k = 0.021 d−1
CALL3, Q9D6P8
n = 1
k = 0.045 d−1
RIA
Time (d)
1.00
0.75
0.50
0.25
0.00
0
10
20
30
40

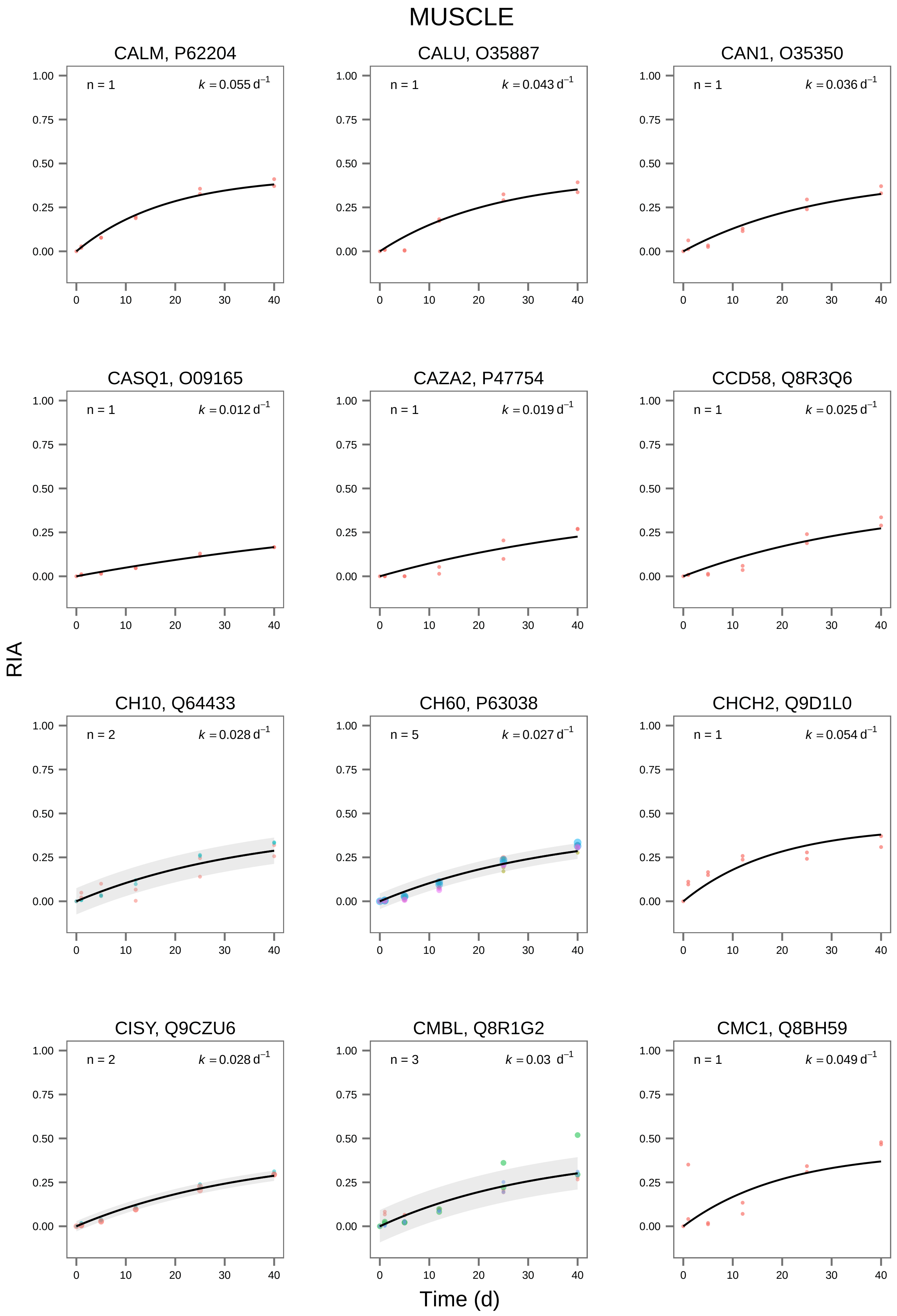
MUSCLE
CALM, P62204
n = 1
k = 0.055 d⁻¹
CALU, O35887
n = 1
k = 0.043 d⁻¹
CAN1, O35350
n = 1
k = 0.036 d⁻¹
CASQ1, O09165
n = 1
k = 0.012 d⁻¹
CAZA2, P47754
n = 1
k = 0.019 d⁻¹
CCD58, Q8R3Q6
n = 1
k = 0.025 d⁻¹
CH10, Q64433
n = 2
k = 0.028 d⁻¹
CH60, P63038
n = 5
k = 0.027 d⁻¹
CHCH2, Q9D1L0
n = 1
k = 0.054 d⁻¹
CISY, Q9CZU6
n = 2
k = 0.028 d⁻¹
CMBL, Q8R1G2
n = 3
k = 0.03 d⁻¹
CMC1, Q8BH59
n = 1
k = 0.049 d⁻¹
RIA
Time (d)
0.00
0.25
0.50
0.75
1.00
0
10
20
30
40

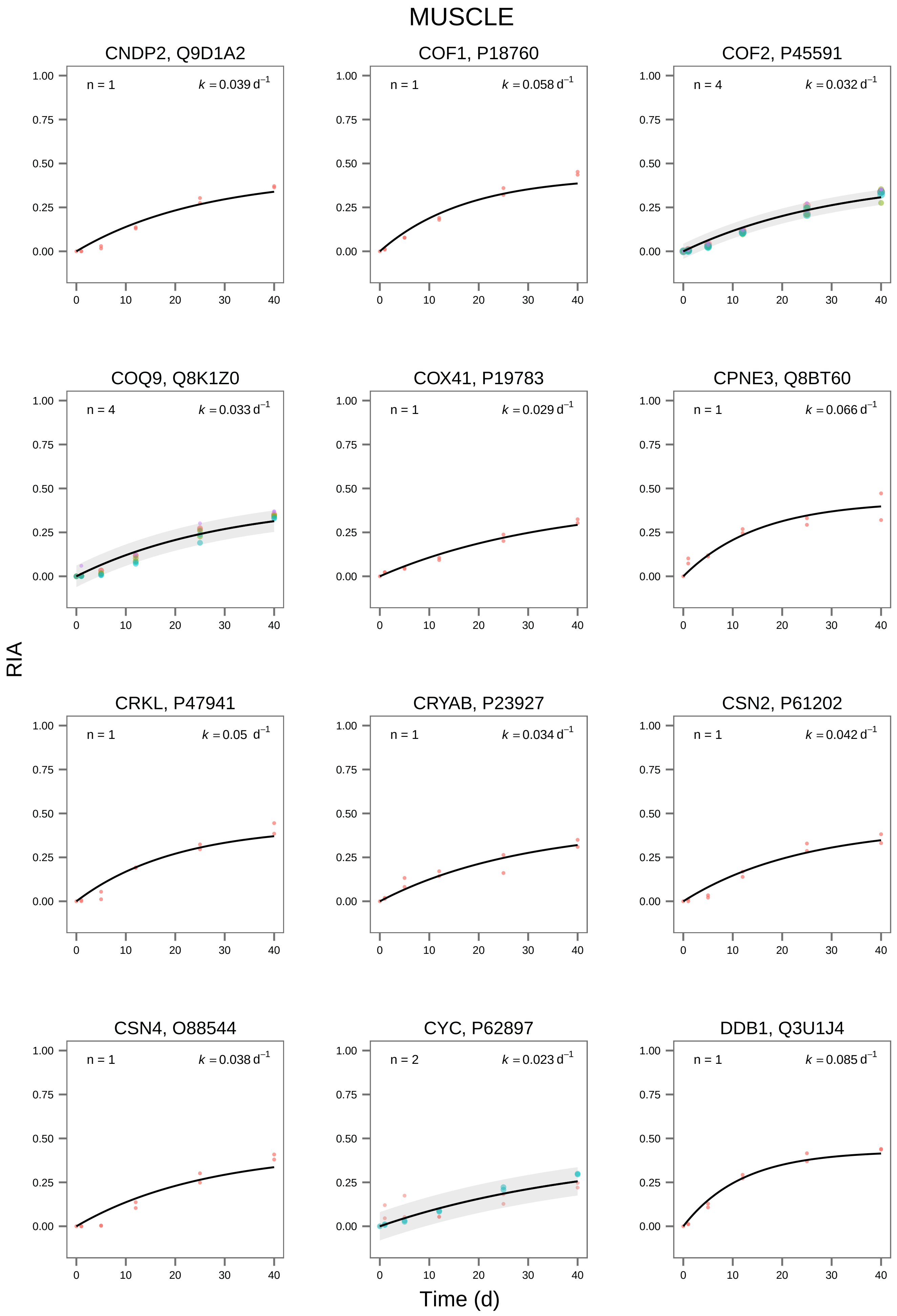

MUSCLE
CNDP2, Q9D1A2
n = 1
k = 0.039 d⁻¹
COF1, P18760
n = 1
k = 0.058 d⁻¹
COF2, P45591
n = 4
k = 0.032 d⁻¹
COQ9, Q8K1Z0
n = 4
k = 0.033 d⁻¹
COX41, P19783
n = 1
k = 0.029 d⁻¹
CPNE3, Q8BT60
n = 1
k = 0.066 d⁻¹
CRKL, P47941
n = 1
k = 0.05 d⁻¹
CRYAB, P23927
n = 1
k = 0.034 d⁻¹
CSN2, P61202
n = 1
k = 0.042 d⁻¹
CSN4, O88544
n = 1
k = 0.038 d⁻¹
CYC, P62897
n = 2
k = 0.023 d⁻¹
DDB1, Q3U1J4
n = 1
k = 0.085 d⁻¹
RIA
Time (d)
1.00
0.75
0.50
0.25
0.00
0
10
20
30
40

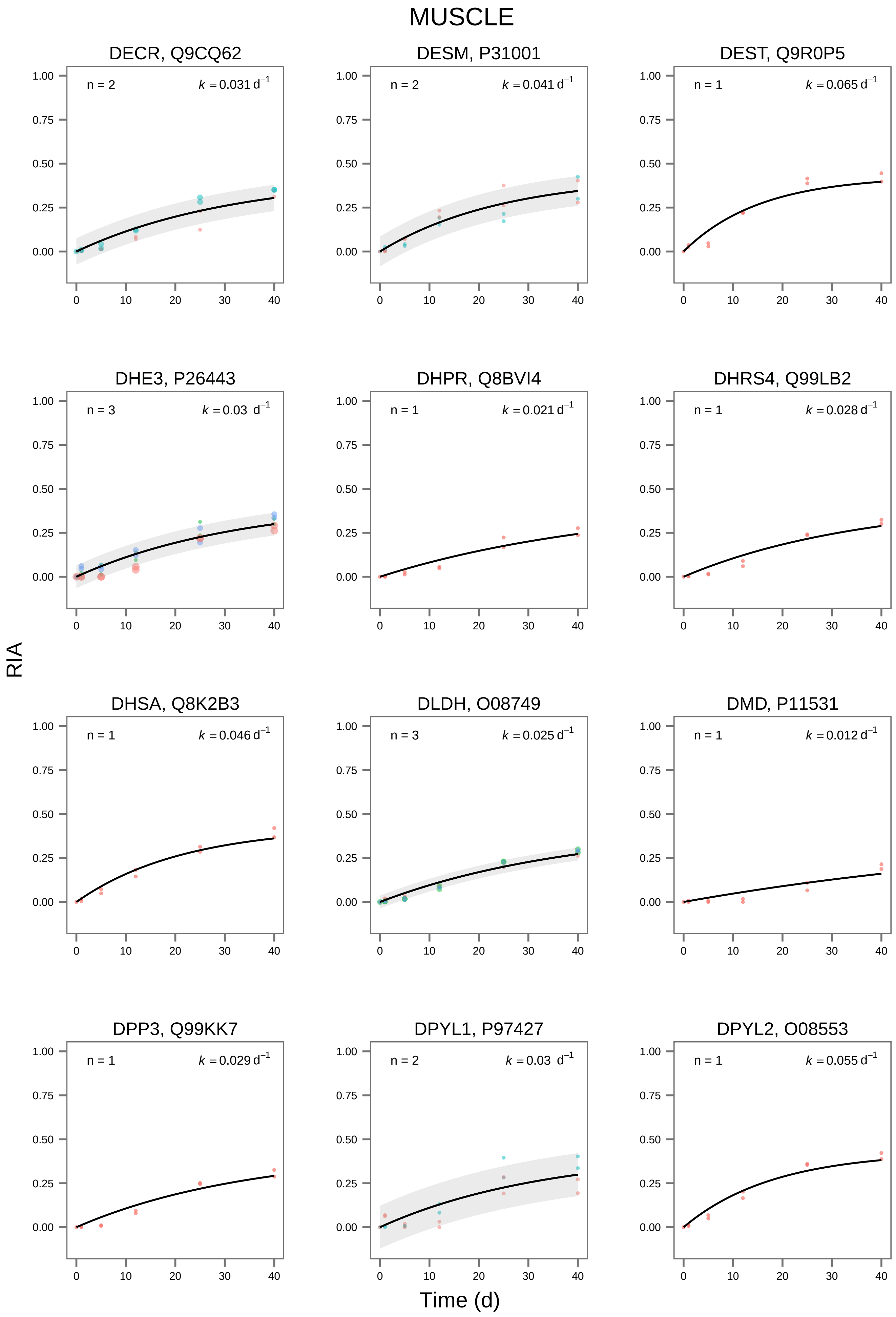
MUSCLE
DECR, Q9CQ62
n = 2
k = 0.031 d^-1
DESM, P31001
n = 2
k = 0.041 d^-1
DEST, Q9R0P5
n = 1
k = 0.065 d^-1
DHE3, P26443
n = 3
k = 0.03 d^-1
DHPR, Q8BVI4
n = 1
k = 0.021 d^-1
DHRS4, Q99LB2
n = 1
k = 0.028 d^-1
DHSA, Q8K2B3
n = 1
k = 0.046 d^-1
DLDH, O08749
n = 3
k = 0.025 d^-1
DMD, P11531
n = 1
k = 0.012 d^-1
DPP3, Q99KK7
n = 1
k = 0.029 d^-1
DPYL1, P97427
n = 2
k = 0.03 d^-1
DPYL2, O08553
n = 1
k = 0.055 d^-1
RIA
Time (d)

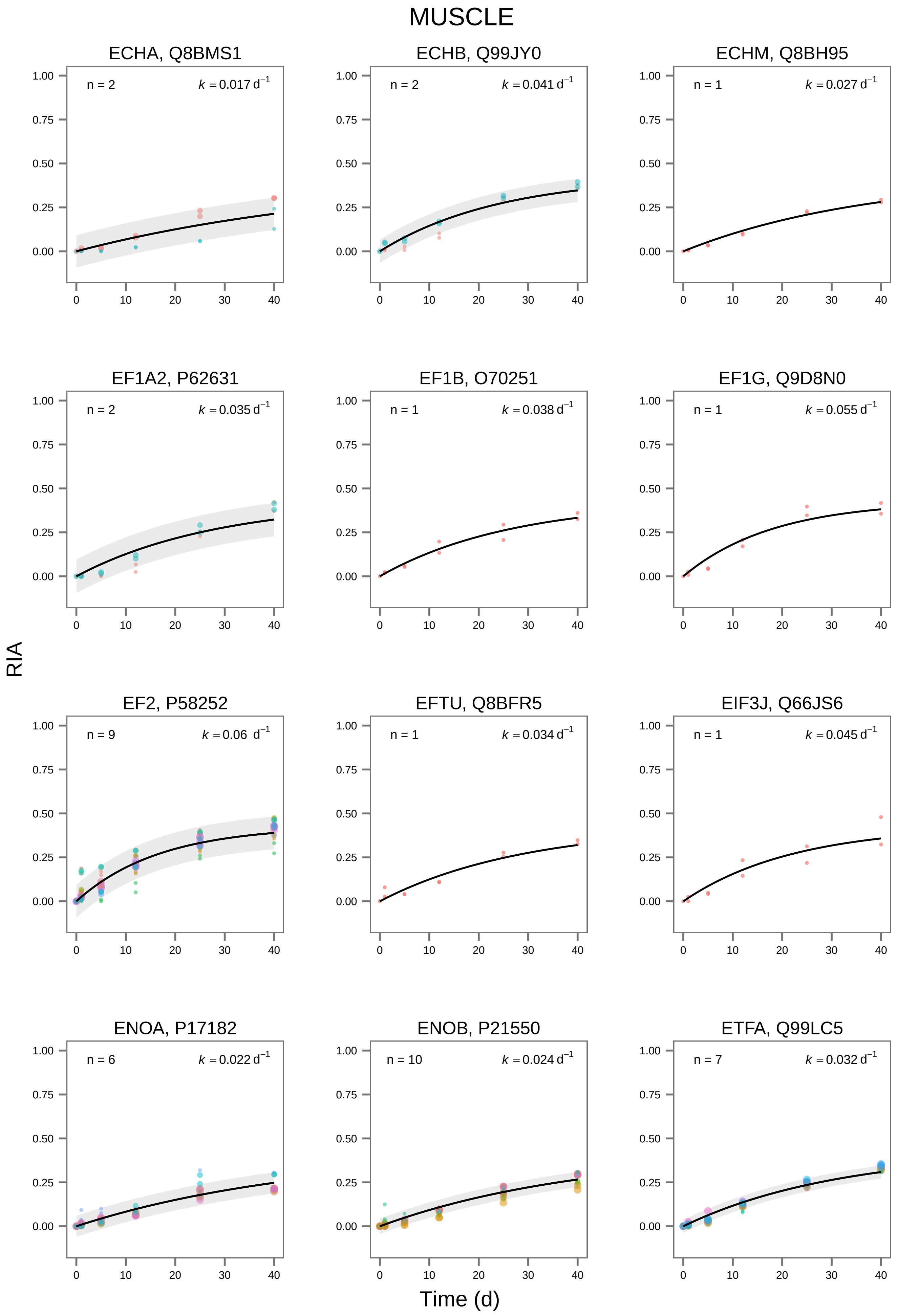
MUSCLE
ECHA, Q8BMS1
n = 2
k = 0.017 d−1
ECHB, Q99JY0
n = 2
k = 0.041 d−1
ECHM, Q8BH95
n = 1
k = 0.027 d−1
EF1A2, P62631
n = 2
k = 0.035 d−1
EF1B, O70251
n = 1
k = 0.038 d−1
EF1G, Q9D8N0
n = 1
k = 0.055 d−1
EF2, P58252
n = 9
k = 0.06 d−1
EFTU, Q8BFR5
n = 1
k = 0.034 d−1
EIF3J, Q66JS6
n = 1
k = 0.045 d−1
ENOA, P17182
n = 6
k = 0.022 d−1
ENOB, P21550
n = 10
k = 0.024 d−1
ETFA, Q99LC5
n = 7
k = 0.032 d−1
RIA
Time (d)

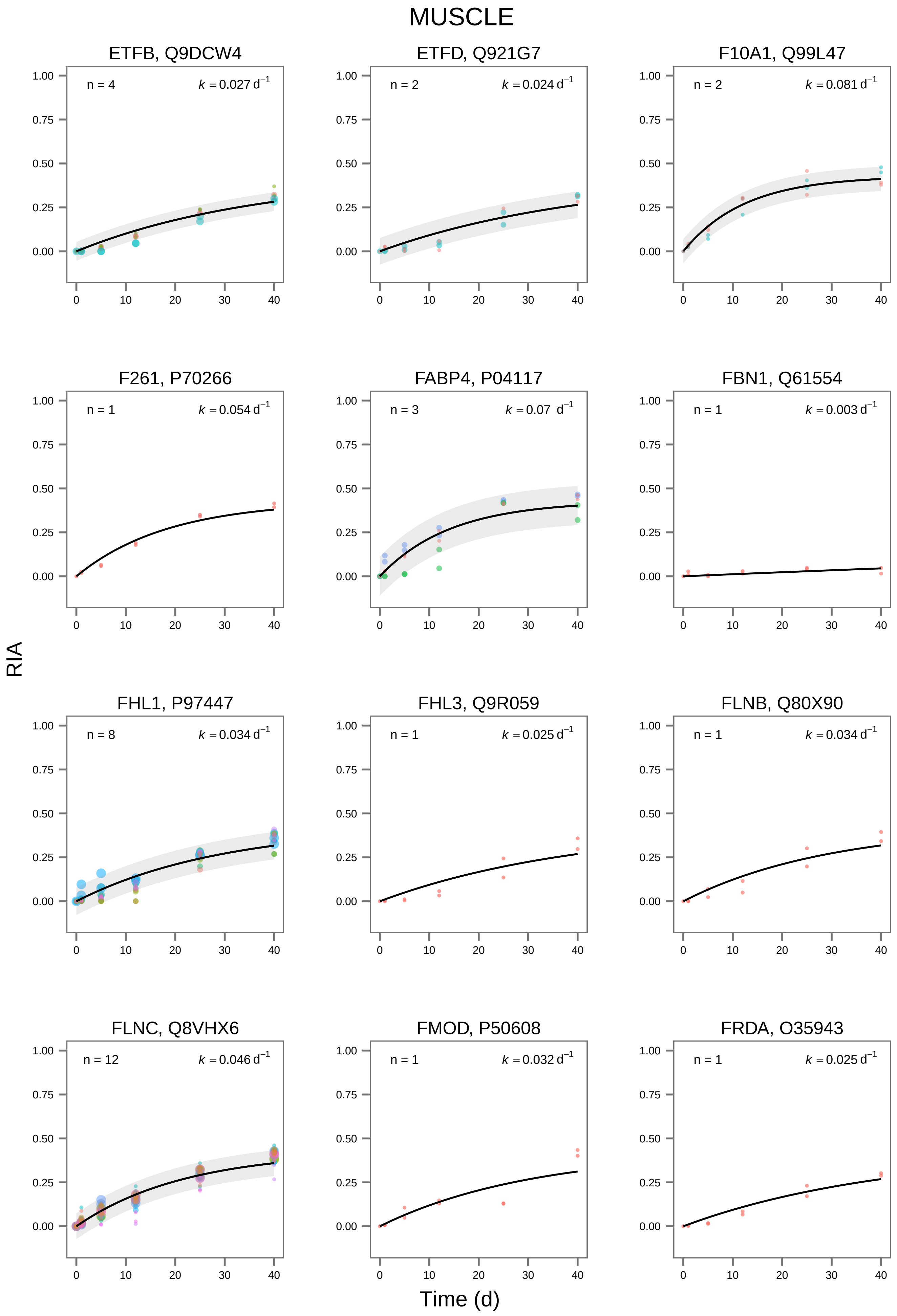
MUSCLE
ETFB, Q9DCW4
n = 4
k = 0.027 d−1
ETFD, Q921G7
n = 2
k = 0.024 d−1
F10A1, Q99L47
n = 2
k = 0.081 d−1
F261, P70266
n = 1
k = 0.054 d−1
FABP4, P04117
n = 3
k = 0.07 d−1
FBN1, Q61554
n = 1
k = 0.003 d−1
FHL1, P97447
n = 8
k = 0.034 d−1
FHL3, Q9R059
n = 1
k = 0.025 d−1
FLNB, Q80X90
n = 1
k = 0.034 d−1
FLNC, Q8VHX6
n = 12
k = 0.046 d−1
FMOD, P50608
n = 1
k = 0.032 d−1
FRDA, O35943
n = 1
k = 0.025 d−1
RIA
Time (d)

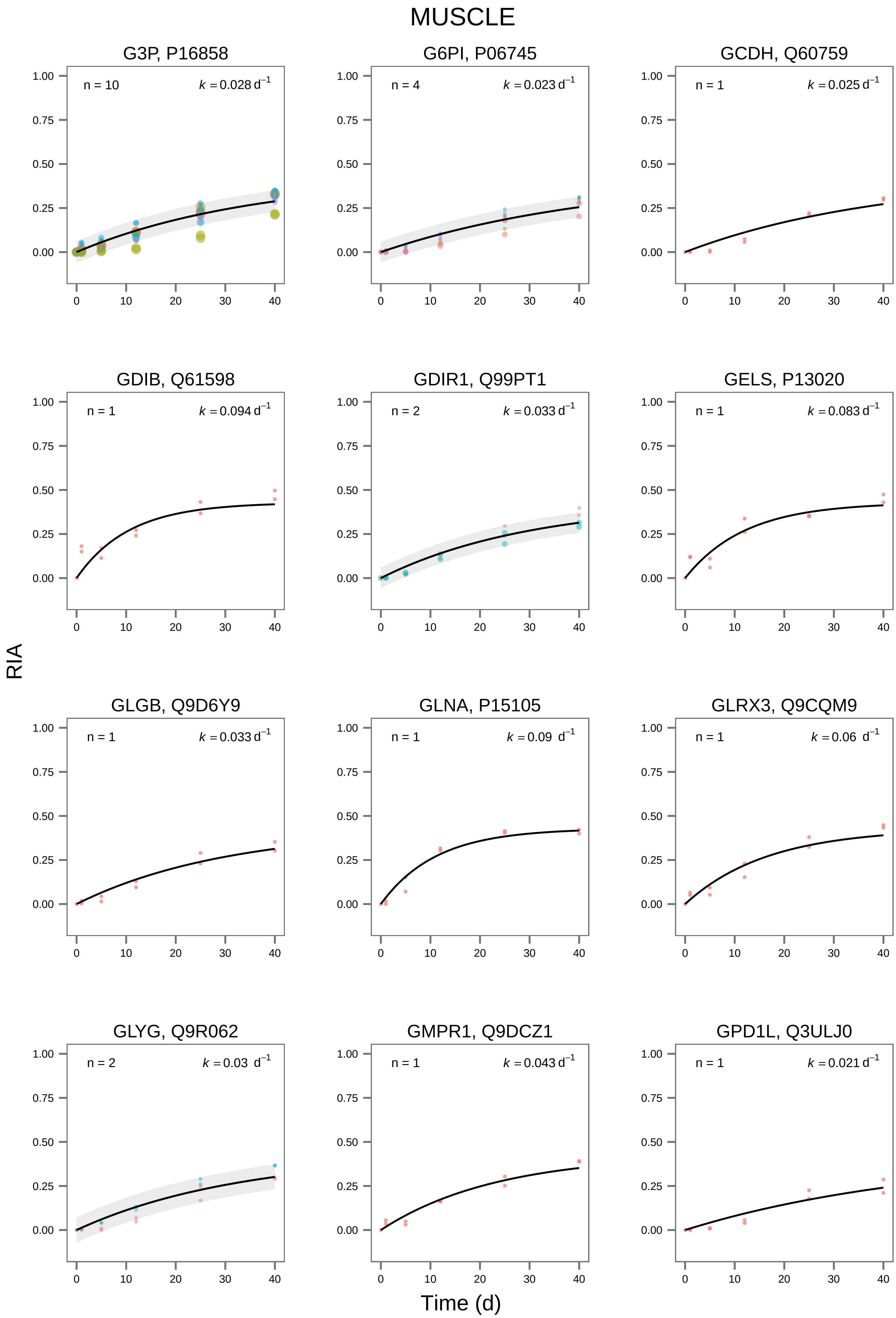

MUSCLE
G3P, P16858
n = 10
k = 0.028 d−1
G6PI, P06745
n = 4
k = 0.023 d−1
GCDH, Q60759
n = 1
k = 0.025 d−1
GDIB, Q61598
n = 1
k = 0.094 d−1
GDIR1, Q99PT1
n = 2
k = 0.033 d−1
GELS, P13020
n = 1
k = 0.083 d−1
GLGB, Q9D6Y9
n = 1
k = 0.033 d−1
GLNA, P15105
n = 1
k = 0.09 d−1
GLRX3, Q9CQM9
n = 1
k = 0.06 d−1
GLYG, Q9R062
n = 2
k = 0.03 d−1
GMPR1, Q9DCZ1
n = 1
k = 0.043 d−1
GPD1L, Q3ULJ0
n = 1
k = 0.021 d−1
RIA
Time (d)

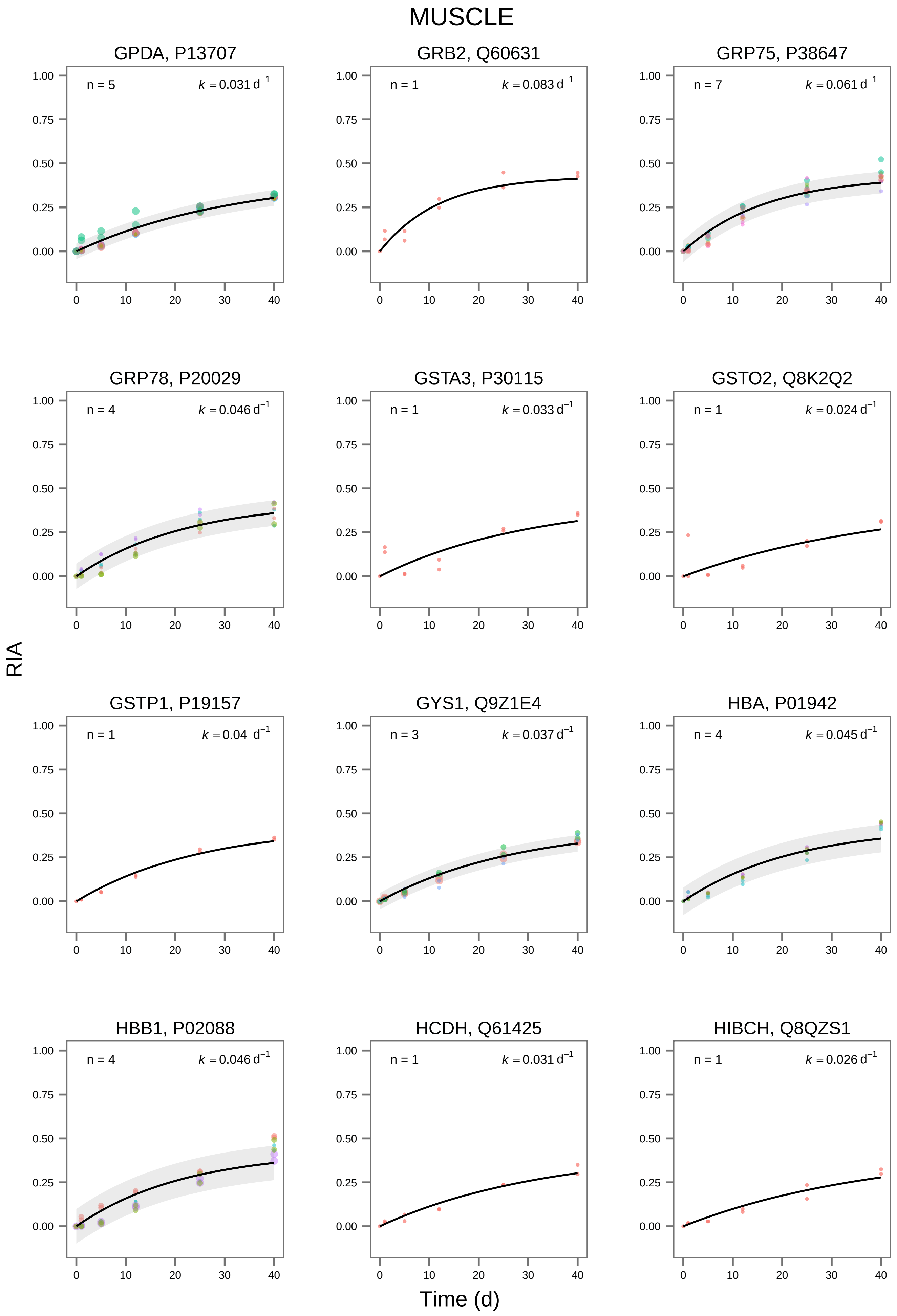
MUSCLE
GPDA, P13707
n = 5
k = 0.031 d⁻¹
GRB2, Q60631
n = 1
k = 0.083 d⁻¹
GRP75, P38647
n = 7
k = 0.061 d⁻¹
GRP78, P20029
n = 4
k = 0.046 d⁻¹
GSTA3, P30115
n = 1
k = 0.033 d⁻¹
GSTO2, Q8K2Q2
n = 1
k = 0.024 d⁻¹
GSTP1, P19157
n = 1
k = 0.04 d⁻¹
GYS1, Q9Z1E4
n = 3
k = 0.037 d⁻¹
HBA, P01942
n = 4
k = 0.045 d⁻¹
HBB1, P02088
n = 4
k = 0.046 d⁻¹
HCDH, Q61425
n = 1
k = 0.031 d⁻¹
HIBCH, Q8QZS1
n = 1
k = 0.026 d⁻¹
RIA
Time (d)

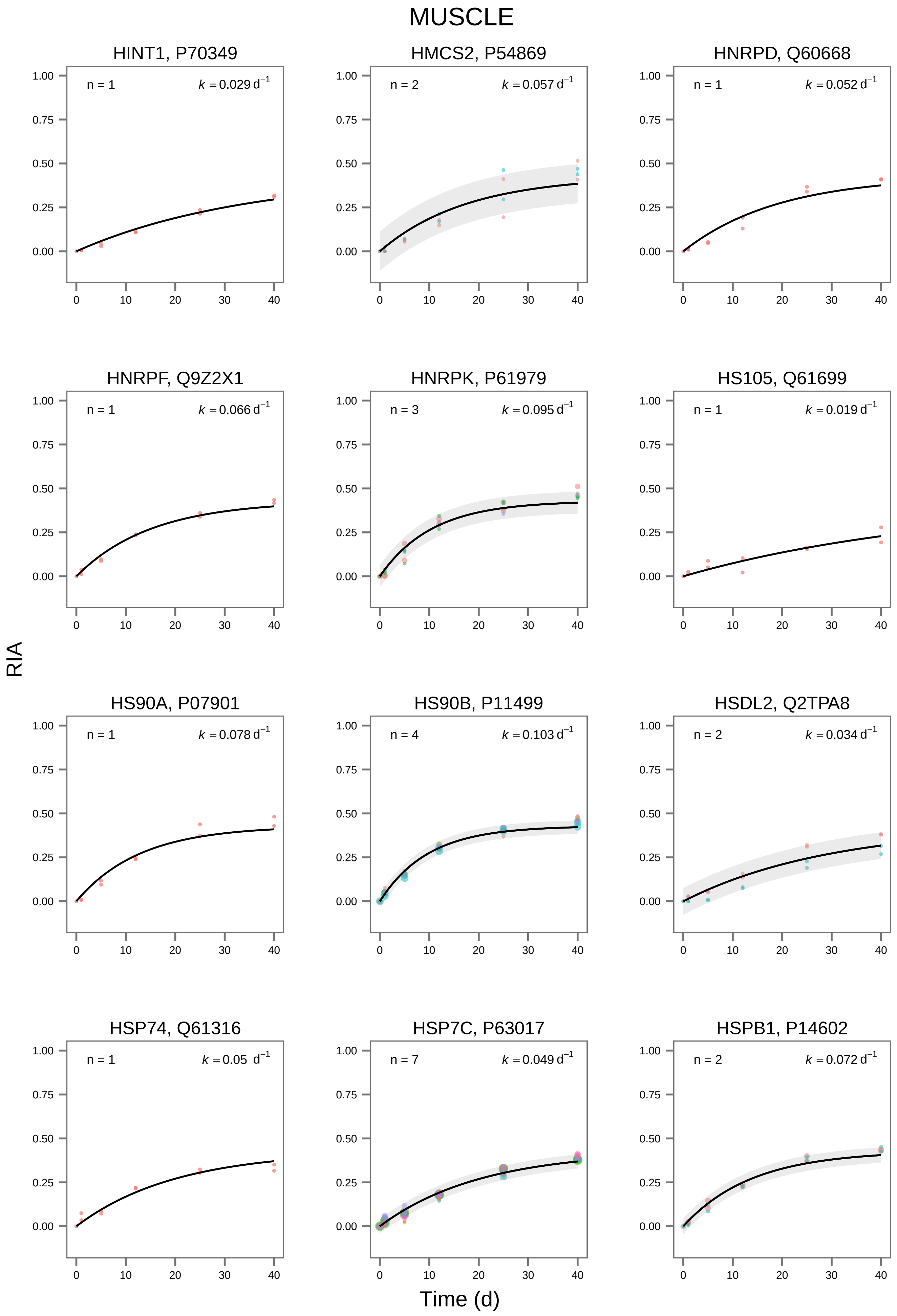
MUSCLE
HINT1, P70349
n = 1
k = 0.029 d−1
HMCS2, P54869
n = 2
k = 0.057 d−1
HNRPD, Q60668
n = 1
k = 0.052 d−1
HNRPF, Q9Z2X1
n = 1
k = 0.066 d−1
HNRPK, P61979
n = 3
k = 0.095 d−1
HS105, Q61699
n = 1
k = 0.019 d−1
HS90A, P07901
n = 1
k = 0.078 d−1
HS90B, P11499
n = 4
k = 0.103 d−1
HSDL2, Q2TPA8
n = 2
k = 0.034 d−1
HSP74, Q61316
n = 1
k = 0.05 d−1
HSP7C, P63017
n = 7
k = 0.049 d−1
HSPB1, P14602
n = 2
k = 0.072 d−1
RIA
Time (d)

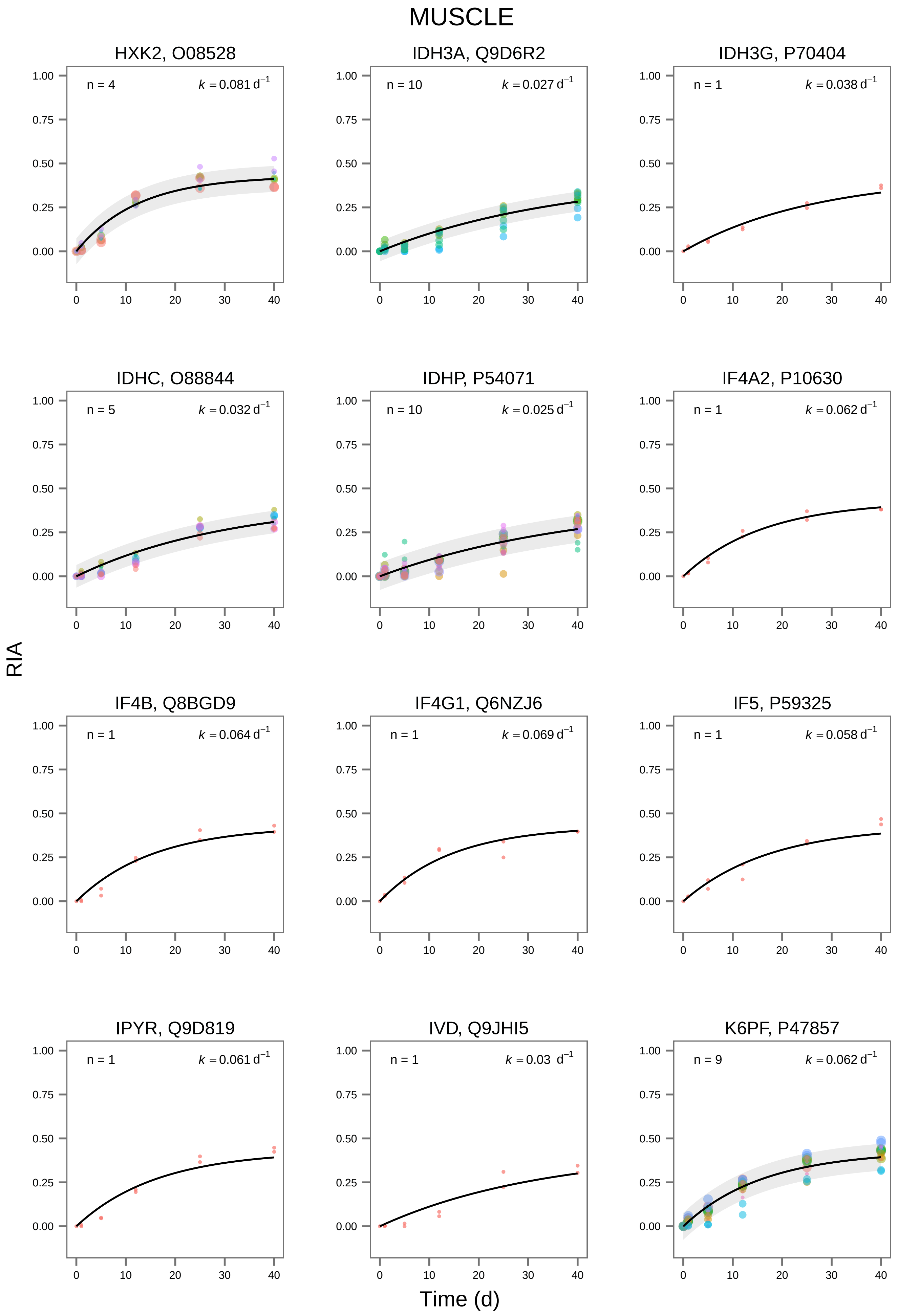
MUSCLE
HXK2, O08528
n = 4
k = 0.081 d−1
IDH3A, Q9D6R2
n = 10
k = 0.027 d−1
IDH3G, P70404
n = 1
k = 0.038 d−1
IDHC, O88844
n = 5
k = 0.032 d−1
IDHP, P54071
n = 10
k = 0.025 d−1
IF4A2, P10630
n = 1
k = 0.062 d−1
IF4B, Q8BGD9
n = 1
k = 0.064 d−1
IF4G1, Q6NZJ6
n = 1
k = 0.069 d−1
IF5, P59325
n = 1
k = 0.058 d−1
IPYR, Q9D819
n = 1
k = 0.061 d−1
IVD, Q9JHI5
n = 1
k = 0.03 d−1
K6PF, P47857
n = 9
k = 0.062 d−1
RIA
Time (d)

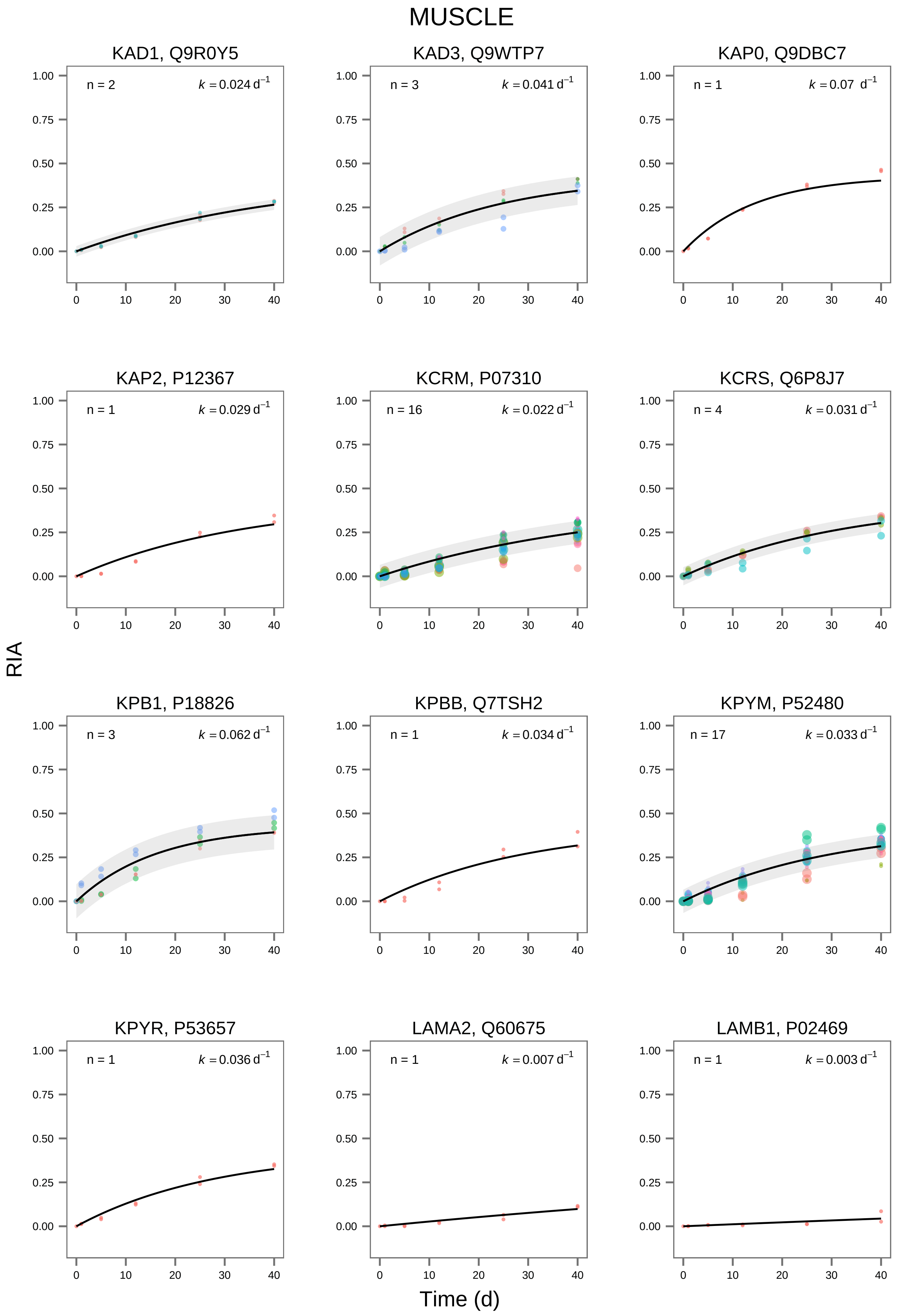
MUSCLE
KAD1, Q9R0Y5
n = 2
k = 0.024 d−1
KAD3, Q9WTP7
n = 3
k = 0.041 d−1
KAP0, Q9DBC7
n = 1
k = 0.07 d−1
KAP2, P12367
n = 1
k = 0.029 d−1
KCRM, P07310
n = 16
k = 0.022 d−1
KCRS, Q6P8J7
n = 4
k = 0.031 d−1
KPB1, P18826
n = 3
k = 0.062 d−1
KPBB, Q7TSH2
n = 1
k = 0.034 d−1
KPYM, P52480
n = 17
k = 0.033 d−1
KPYR, P53657
n = 1
k = 0.036 d−1
LAMA2, Q60675
n = 1
k = 0.007 d−1
LAMB1, P02469
n = 1
k = 0.003 d−1
RIA
Time (d)

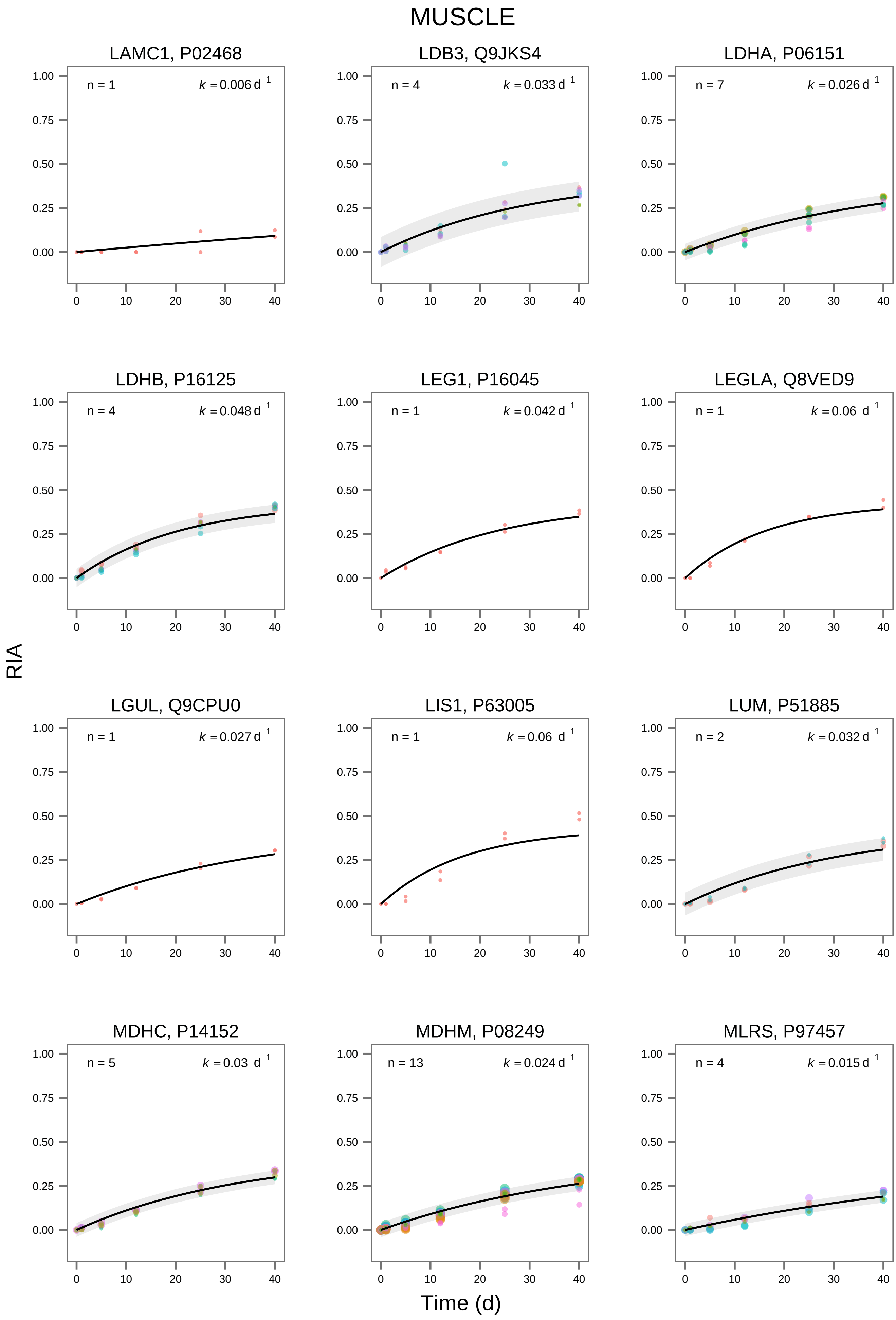
MUSCLE
LAMC1, P02468
n = 1
k = 0.006 d−1
LDB3, Q9JKS4
n = 4
k = 0.033 d−1
LDHA, P06151
n = 7
k = 0.026 d−1
LDHB, P16125
n = 4
k = 0.048 d−1
LEG1, P16045
n = 1
k = 0.042 d−1
LEGLA, Q8VED9
n = 1
k = 0.06 d−1
LGUL, Q9CPU0
n = 1
k = 0.027 d−1
LIS1, P63005
n = 1
k = 0.06 d−1
LUM, P51885
n = 2
k = 0.032 d−1
MDHC, P14152
n = 5
k = 0.03 d−1
MDHM, P08249
n = 13
k = 0.024 d−1
MLRS, P97457
n = 4
k = 0.015 d−1
RIA
Time (d)

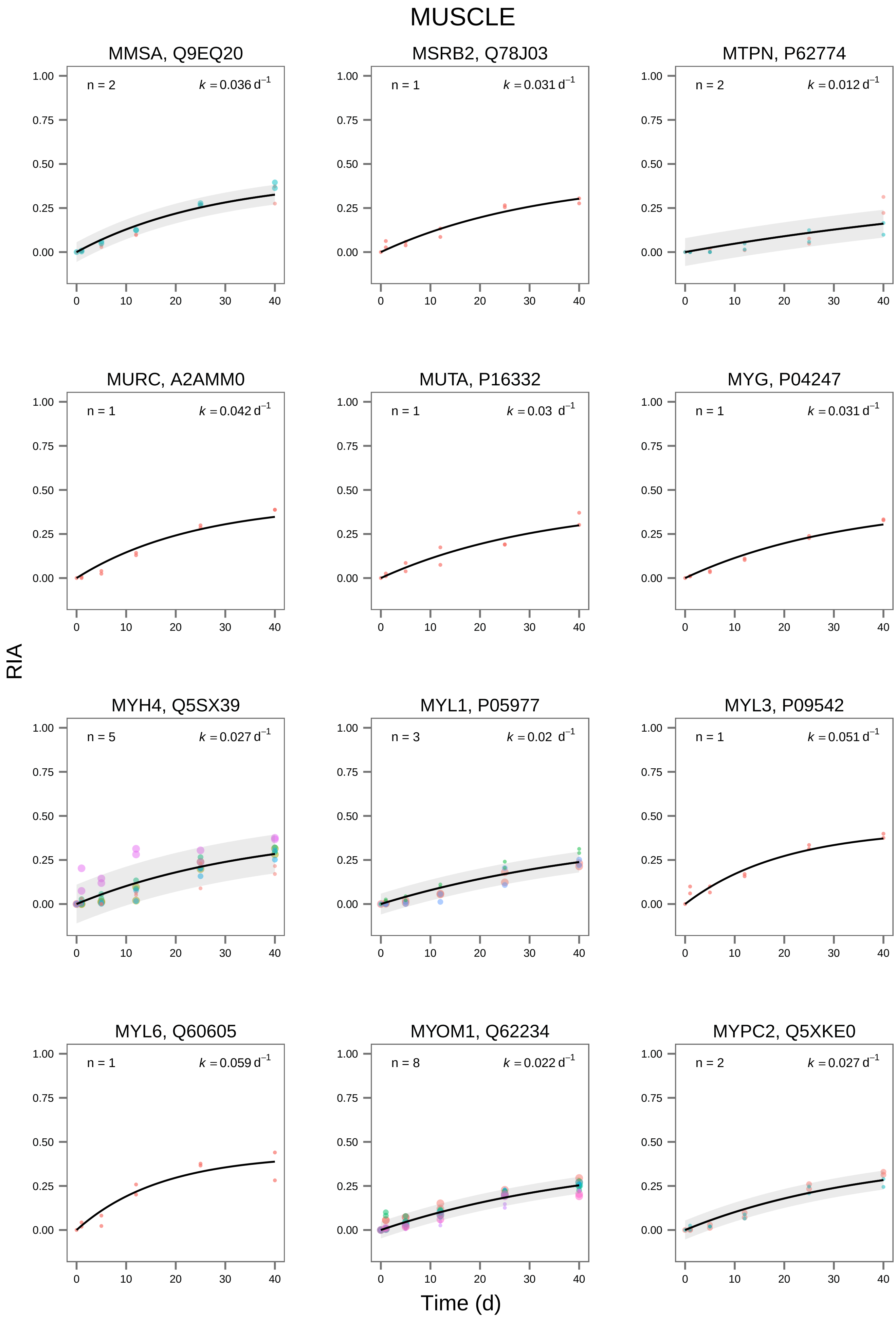
MUSCLE
MMSA, Q9EQ20
n = 2
k = 0.036 d⁻¹
MSRB2, Q78J03
n = 1
k = 0.031 d⁻¹
MTPN, P62774
n = 2
k = 0.012 d⁻¹
MURC, A2AMM0
n = 1
k = 0.042 d⁻¹
MUTA, P16332
n = 1
k = 0.03 d⁻¹
MYG, P04247
n = 1
k = 0.031 d⁻¹
MYH4, Q5SX39
n = 5
k = 0.027 d⁻¹
MYL1, P05977
n = 3
k = 0.02 d⁻¹
MYL3, P09542
n = 1
k = 0.051 d⁻¹
MYL6, Q60605
n = 1
k = 0.059 d⁻¹
MYOM1, Q62234
n = 8
k = 0.022 d⁻¹
MYPC2, Q5XKE0
n = 2
k = 0.027 d⁻¹
RIA
Time (d)
0.00
0.25
0.50
0.75
1.00
0
10
20
30
40

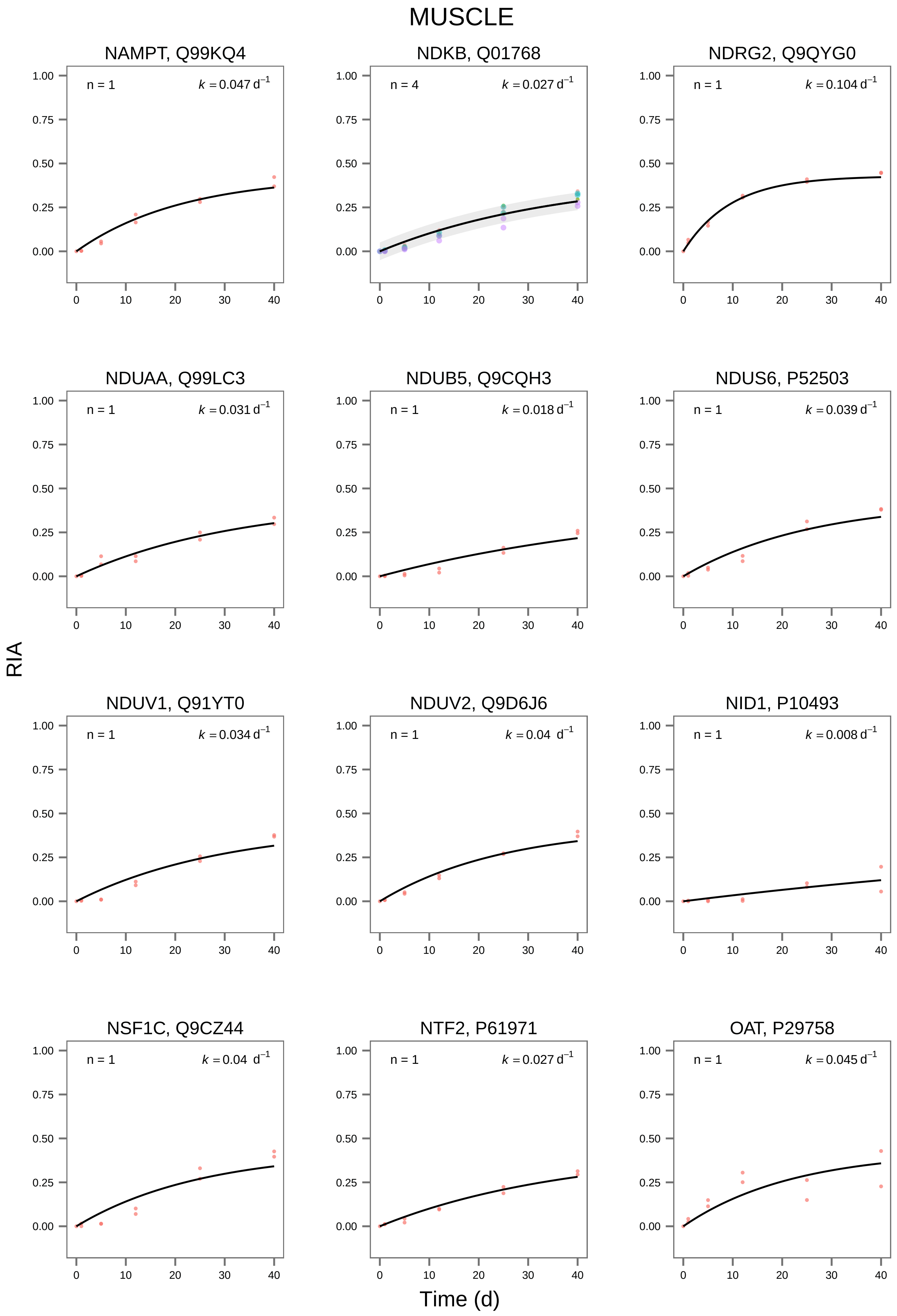
MUSCLE
NAMPT, Q99KQ4
n = 1
k = 0.047 d^-1
NDKB, Q01768
n = 4
k = 0.027 d^-1
NDRG2, Q9QYG0
n = 1
k = 0.104 d^-1
NDUAA, Q99LC3
n = 1
k = 0.031 d^-1
NDUB5, Q9CQH3
n = 1
k = 0.018 d^-1
NDUS6, P52503
n = 1
k = 0.039 d^-1
NDUV1, Q91YT0
n = 1
k = 0.034 d^-1
NDUV2, Q9D6J6
n = 1
k = 0.04 d^-1
NID1, P10493
n = 1
k = 0.008 d^-1
NSF1C, Q9CZ44
n = 1
k = 0.04 d^-1
NTF2, P61971
n = 1
k = 0.027 d^-1
OAT, P29758
n = 1
k = 0.045 d^-1
1.00
0.75
0.50
0.25
0.00
0
10
20
30
40
RIA
Time (d)

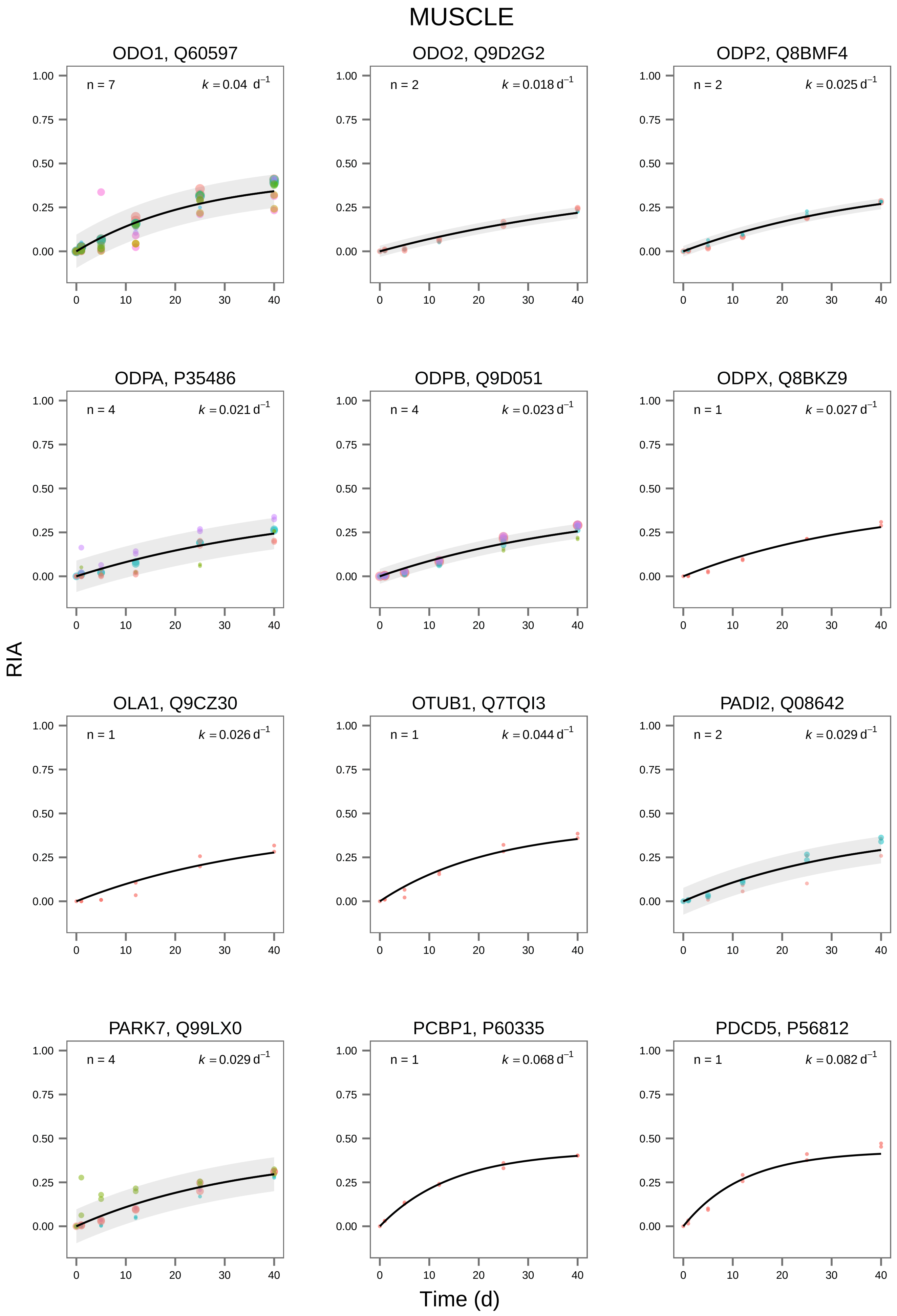
MUSCLE
ODO1, Q60597
n = 7
k = 0.04 d−1
ODO2, Q9D2G2
n = 2
k = 0.018 d−1
ODP2, Q8BMF4
n = 2
k = 0.025 d−1
ODPA, P35486
n = 4
k = 0.021 d−1
ODPB, Q9D051
n = 4
k = 0.023 d−1
ODPX, Q8BKZ9
n = 1
k = 0.027 d−1
OLA1, Q9CZ30
n = 1
k = 0.026 d−1
OTUB1, Q7TQI3
n = 1
k = 0.044 d−1
PADI2, Q08642
n = 2
k = 0.029 d−1
PARK7, Q99LX0
n = 4
k = 0.029 d−1
PCBP1, P60335
n = 1
k = 0.068 d−1
PDCD5, P56812
n = 1
k = 0.082 d−1
RIA
Time (d)

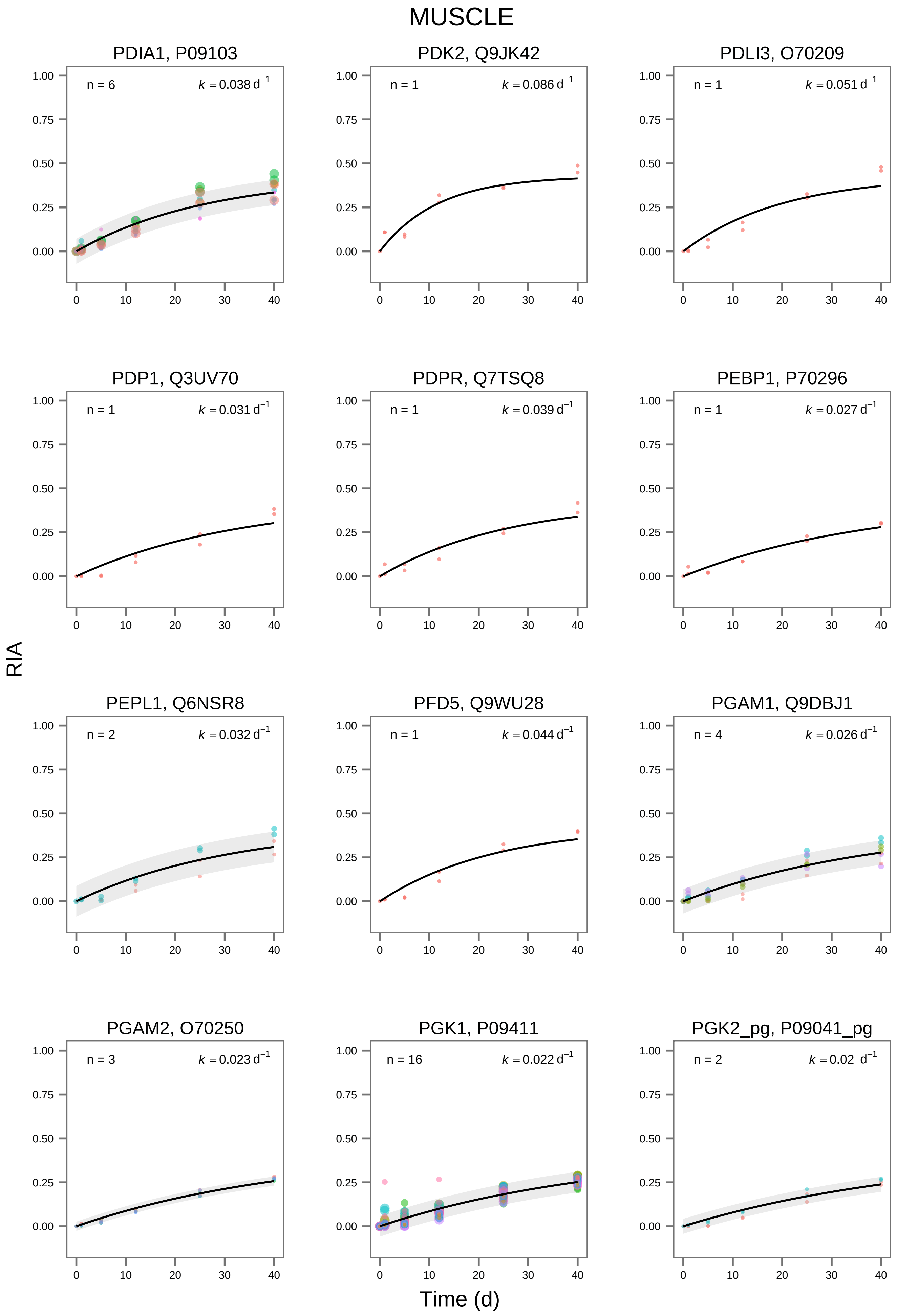
MUSCLE
PDIA1, P09103
n = 6
k = 0.038 d−1
PDK2, Q9JK42
n = 1
k = 0.086 d−1
PDLI3, O70209
n = 1
k = 0.051 d−1
PDP1, Q3UV70
n = 1
k = 0.031 d−1
PDPR, Q7TSQ8
n = 1
k = 0.039 d−1
PEBP1, P70296
n = 1
k = 0.027 d−1
PEPL1, Q6NSR8
n = 2
k = 0.032 d−1
PFD5, Q9WU28
n = 1
k = 0.044 d−1
PGAM1, Q9DBJ1
n = 4
k = 0.026 d−1
PGAM2, O70250
n = 3
k = 0.023 d−1
PGK1, P09411
n = 16
k = 0.022 d−1
PGK2_pg, P09041_pg
n = 2
k = 0.02 d−1
RIA
Time (d)

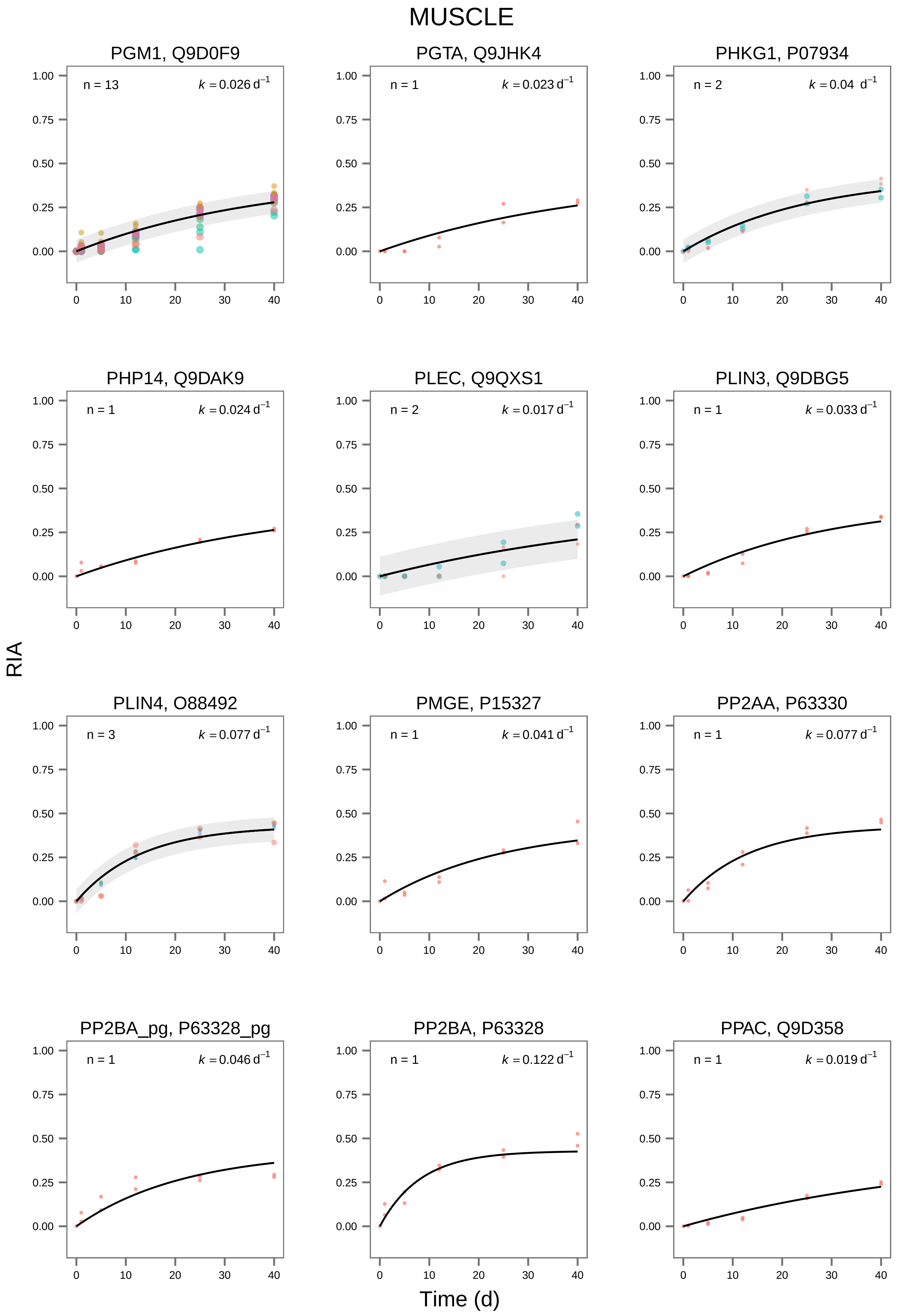
MUSCLE
PGM1, Q9D0F9
n = 13
k = 0.026 d−1
PGTA, Q9JHK4
n = 1
k = 0.023 d−1
PHKG1, P07934
n = 2
k = 0.04 d−1
PHP14, Q9DAK9
n = 1
k = 0.024 d−1
PLEC, Q9QXS1
n = 2
k = 0.017 d−1
PLIN3, Q9DBG5
n = 1
k = 0.033 d−1
PLIN4, O88492
n = 3
k = 0.077 d−1
PMGE, P15327
n = 1
k = 0.041 d−1
PP2AA, P63330
n = 1
k = 0.077 d−1
PP2BA_pg, P63328_pg
n = 1
k = 0.046 d−1
PP2BA, P63328
n = 1
k = 0.122 d−1
PPAC, Q9D358
n = 1
k = 0.019 d−1
RIA
Time (d)

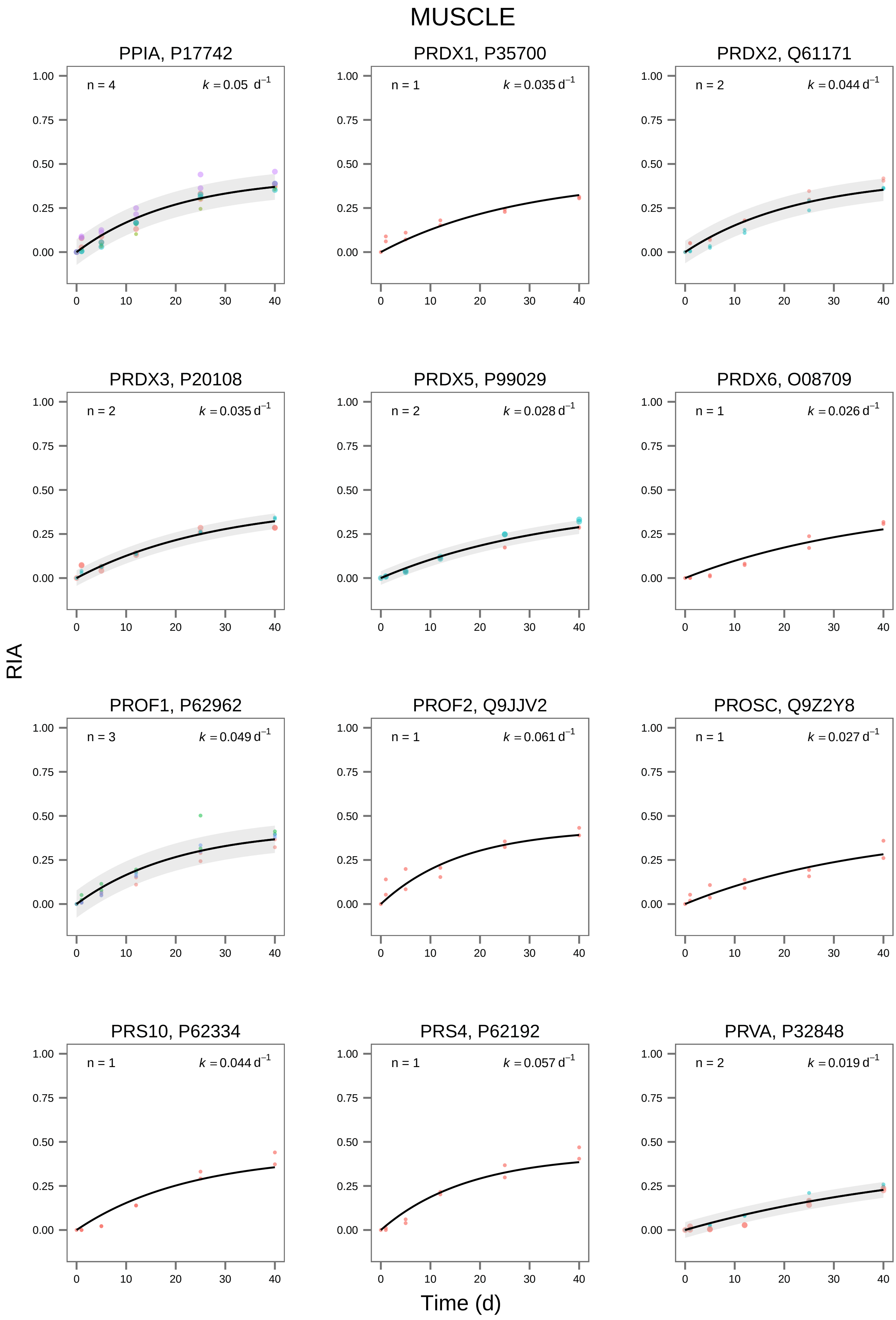
MUSCLE
PPIA, P17742
n = 4
k = 0.05 d−1
PRDX1, P35700
n = 1
k = 0.035 d−1
PRDX2, Q61171
n = 2
k = 0.044 d−1
PRDX3, P20108
n = 2
k = 0.035 d−1
PRDX5, P99029
n = 2
k = 0.028 d−1
PRDX6, O08709
n = 1
k = 0.026 d−1
PROF1, P62962
n = 3
k = 0.049 d−1
PROF2, Q9JJV2
n = 1
k = 0.061 d−1
PROSC, Q9Z2Y8
n = 1
k = 0.027 d−1
PRS10, P62334
n = 1
k = 0.044 d−1
PRS4, P62192
n = 1
k = 0.057 d−1
PRVA, P32848
n = 2
k = 0.019 d−1
RIA
Time (d)

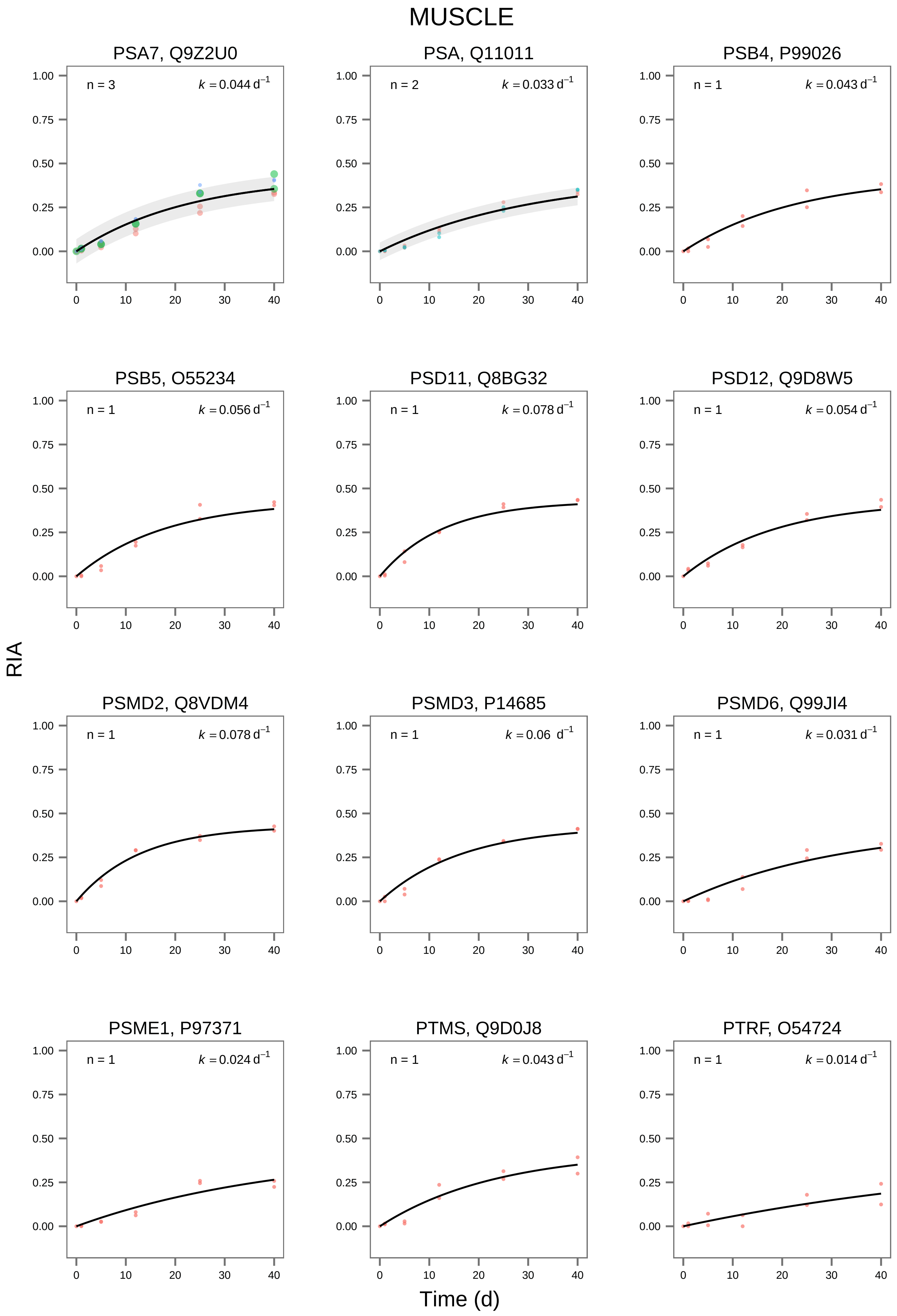
MUSCLE
PSA7, Q9Z2U0
n = 3
k = 0.044 d⁻¹
PSA, Q11011
n = 2
k = 0.033 d⁻¹
PSB4, P99026
n = 1
k = 0.043 d⁻¹
PSB5, O55234
n = 1
k = 0.056 d⁻¹
PSD11, Q8BG32
n = 1
k = 0.078 d⁻¹
PSD12, Q9D8W5
n = 1
k = 0.054 d⁻¹
PSMD2, Q8VDM4
n = 1
k = 0.078 d⁻¹
PSMD3, P14685
n = 1
k = 0.06 d⁻¹
PSMD6, Q99JI4
n = 1
k = 0.031 d⁻¹
PSME1, P97371
n = 1
k = 0.024 d⁻¹
PTMS, Q9D0J8
n = 1
k = 0.043 d⁻¹
PTRF, O54724
n = 1
k = 0.014 d⁻¹
RIA
Time (d)
1.00
0.75
0.50
0.25
0.00
0
10
20
30
40

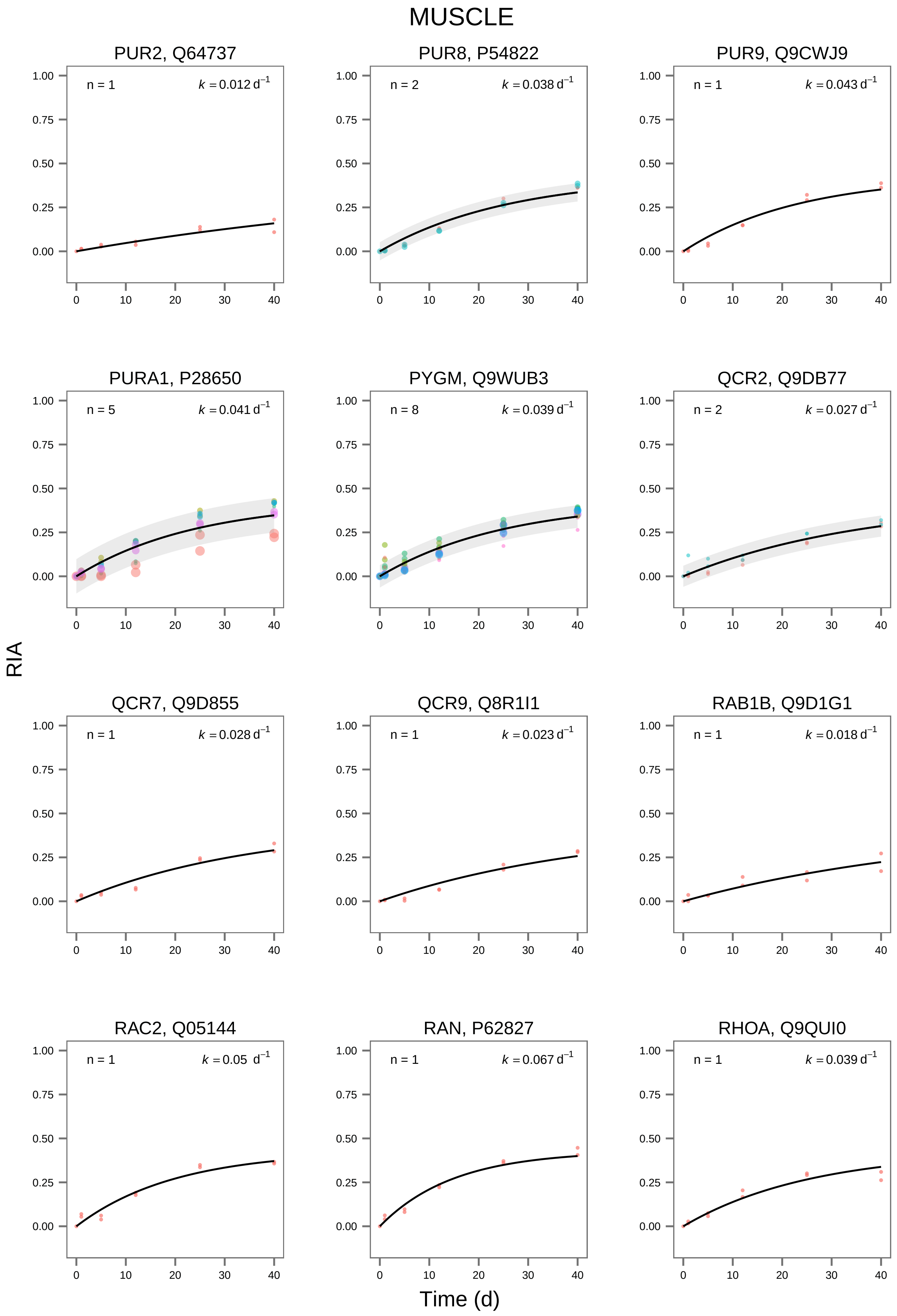
MUSCLE
PUR2, Q64737
n = 1
k = 0.012 d−1
PUR8, P54822
n = 2
k = 0.038 d−1
PUR9, Q9CWJ9
n = 1
k = 0.043 d−1
PURA1, P28650
n = 5
k = 0.041 d−1
PYGM, Q9WUB3
n = 8
k = 0.039 d−1
QCR2, Q9DB77
n = 2
k = 0.027 d−1
QCR7, Q9D855
n = 1
k = 0.028 d−1
QCR9, Q8R1I1
n = 1
k = 0.023 d−1
RAB1B, Q9D1G1
n = 1
k = 0.018 d−1
RAC2, Q05144
n = 1
k = 0.05 d−1
RAN, P62827
n = 1
k = 0.067 d−1
RHOA, Q9QUI0
n = 1
k = 0.039 d−1
RIA
Time (d)

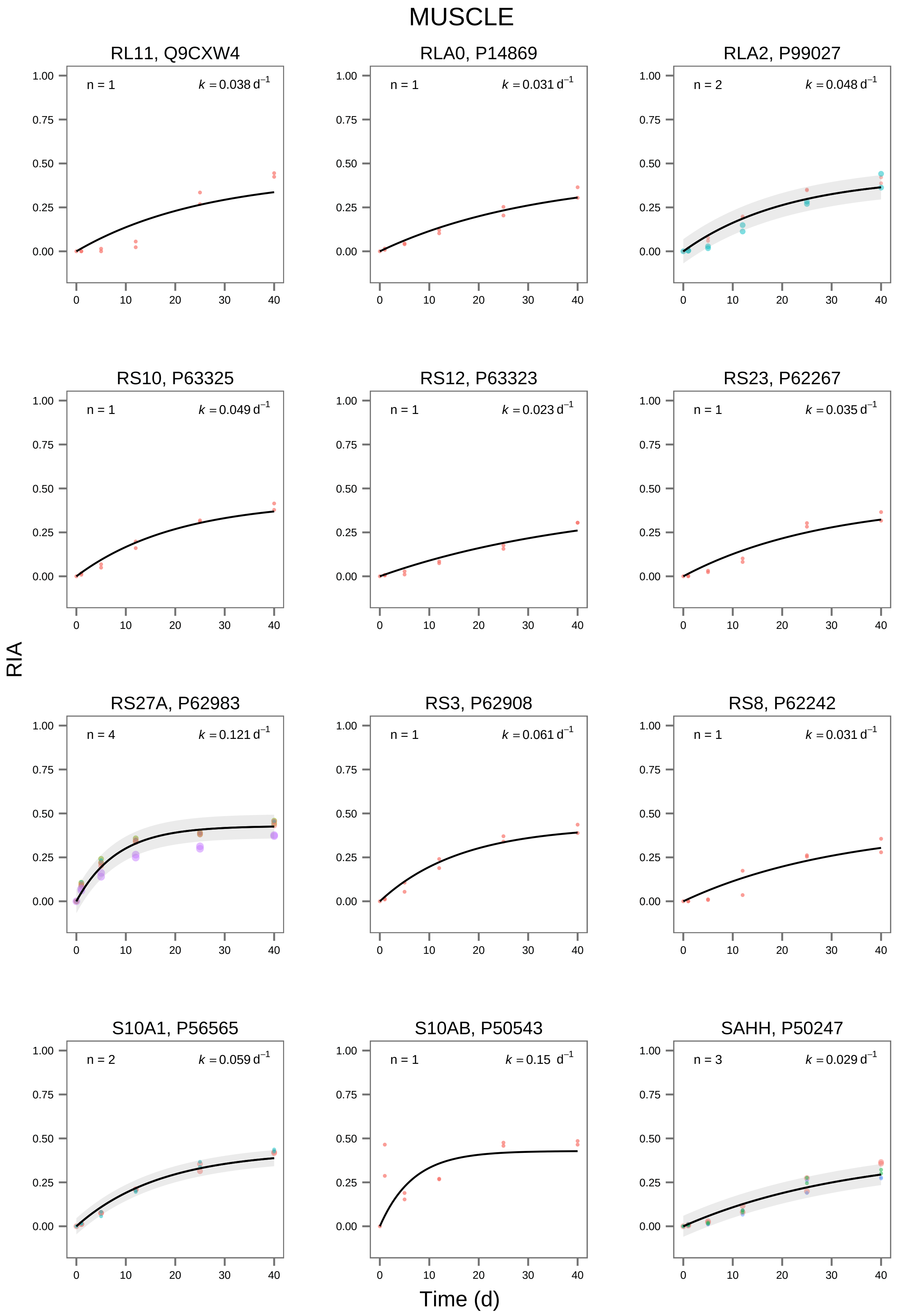
MUSCLE
RL11, Q9CXW4
n = 1
k = 0.038 d−1
RLA0, P14869
n = 1
k = 0.031 d−1
RLA2, P99027
n = 2
k = 0.048 d−1
RS10, P63325
n = 1
k = 0.049 d−1
RS12, P63323
n = 1
k = 0.023 d−1
RS23, P62267
n = 1
k = 0.035 d−1
RS27A, P62983
n = 4
k = 0.121 d−1
RS3, P62908
n = 1
k = 0.061 d−1
RS8, P62242
n = 1
k = 0.031 d−1
S10A1, P56565
n = 2
k = 0.059 d−1
S10AB, P50543
n = 1
k = 0.15 d−1
SAHH, P50247
n = 3
k = 0.029 d−1
RIA
Time (d)
0.00
0.25
0.50
0.75
1.00
0
10
20
30
40

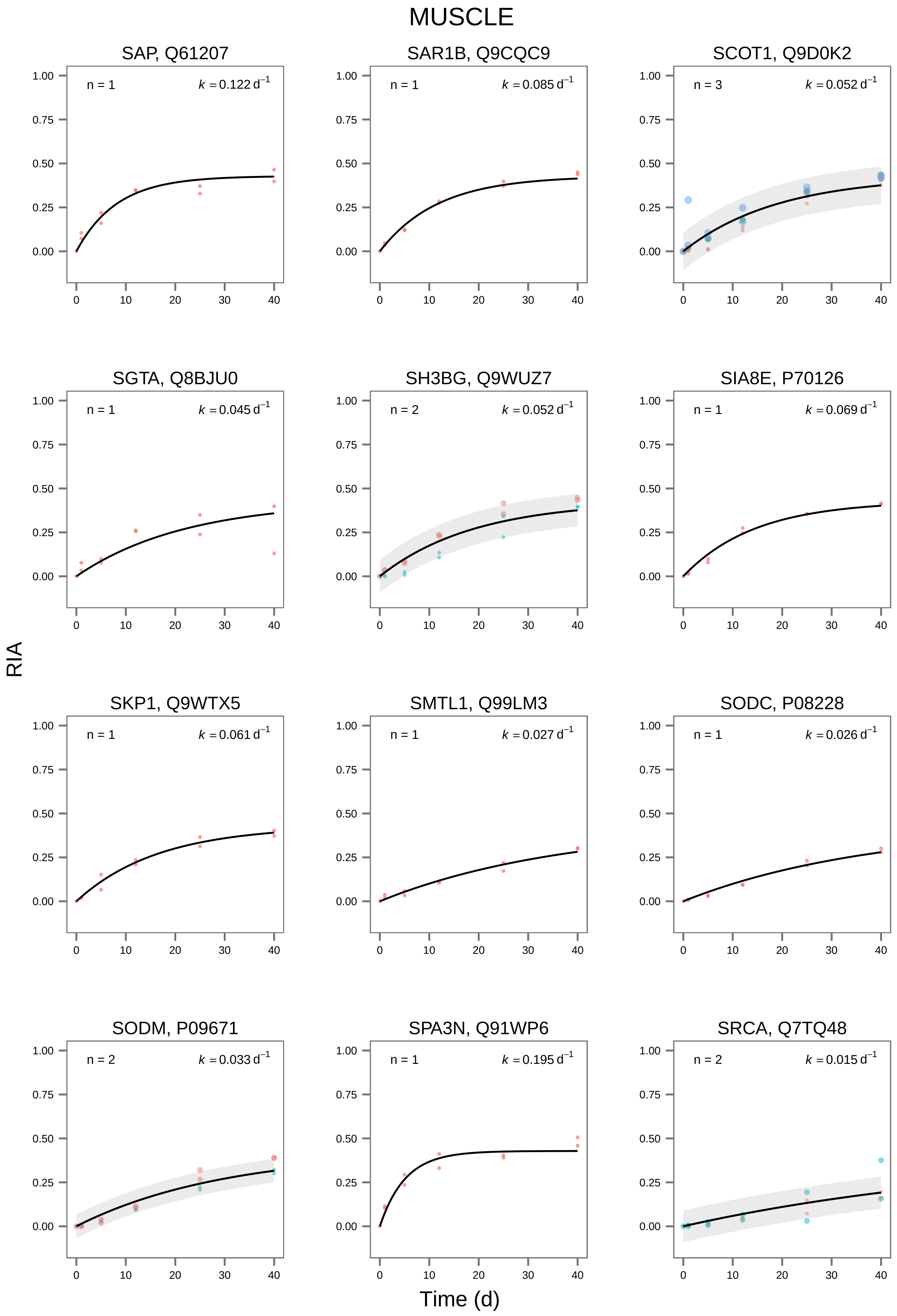
MUSCLE
SAP, Q61207
n = 1
k = 0.122 d−1
SAR1B, Q9CQC9
n = 1
k = 0.085 d−1
SCOT1, Q9D0K2
n = 3
k = 0.052 d−1
SGTA, Q8BJU0
n = 1
k = 0.045 d−1
SH3BG, Q9WUZ7
n = 2
k = 0.052 d−1
SIA8E, P70126
n = 1
k = 0.069 d−1
SKP1, Q9WTX5
n = 1
k = 0.061 d−1
SMTL1, Q99LM3
n = 1
k = 0.027 d−1
SODC, P08228
n = 1
k = 0.026 d−1
SODM, P09671
n = 2
k = 0.033 d−1
SPA3N, Q91WP6
n = 1
k = 0.195 d−1
SRCA, Q7TQ48
n = 2
k = 0.015 d−1
RIA
Time (d)

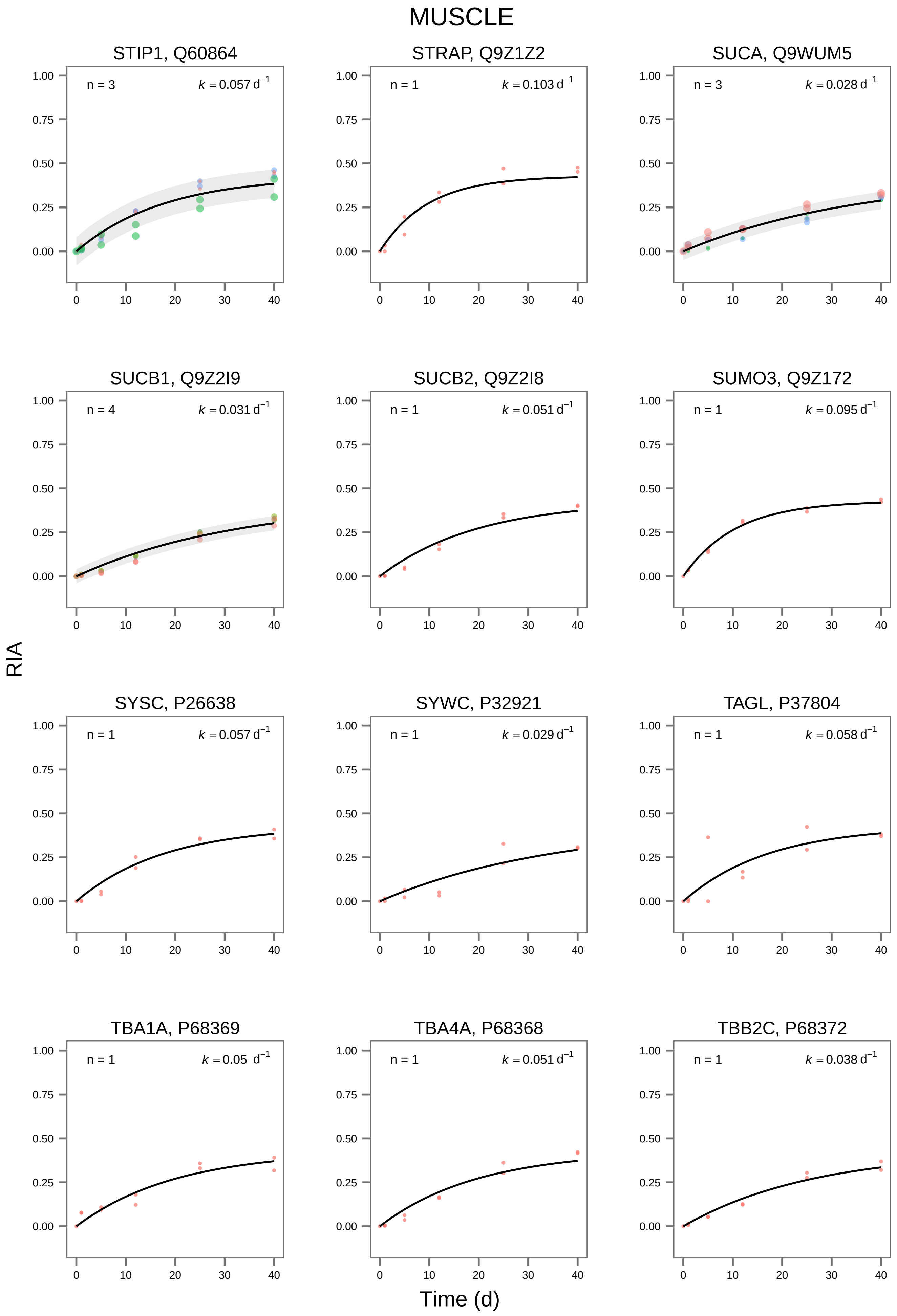
MUSCLE
STIP1, Q60864
n = 3
$k = 0.057\ d^{-1}$
STRAP, Q9Z1Z2
n = 1
$k = 0.103\ d^{-1}$
SUCA, Q9WUM5
n = 3
$k = 0.028\ d^{-1}$
SUCB1, Q9Z2I9
n = 4
$k = 0.031\ d^{-1}$
SUCB2, Q9Z2I8
n = 1
$k = 0.051\ d^{-1}$
SUMO3, Q9Z172
n = 1
$k = 0.095\ d^{-1}$
SYSC, P26638
n = 1
$k = 0.057\ d^{-1}$
SYWC, P32921
n = 1
$k = 0.029\ d^{-1}$
TAGL, P37804
n = 1
$k = 0.058\ d^{-1}$
TBA1A, P68369
n = 1
$k = 0.05\ d^{-1}$
TBA4A, P68368
n = 1
$k = 0.051\ d^{-1}$
TBB2C, P68372
n = 1
$k = 0.038\ d^{-1}$
RIA
Time (d)

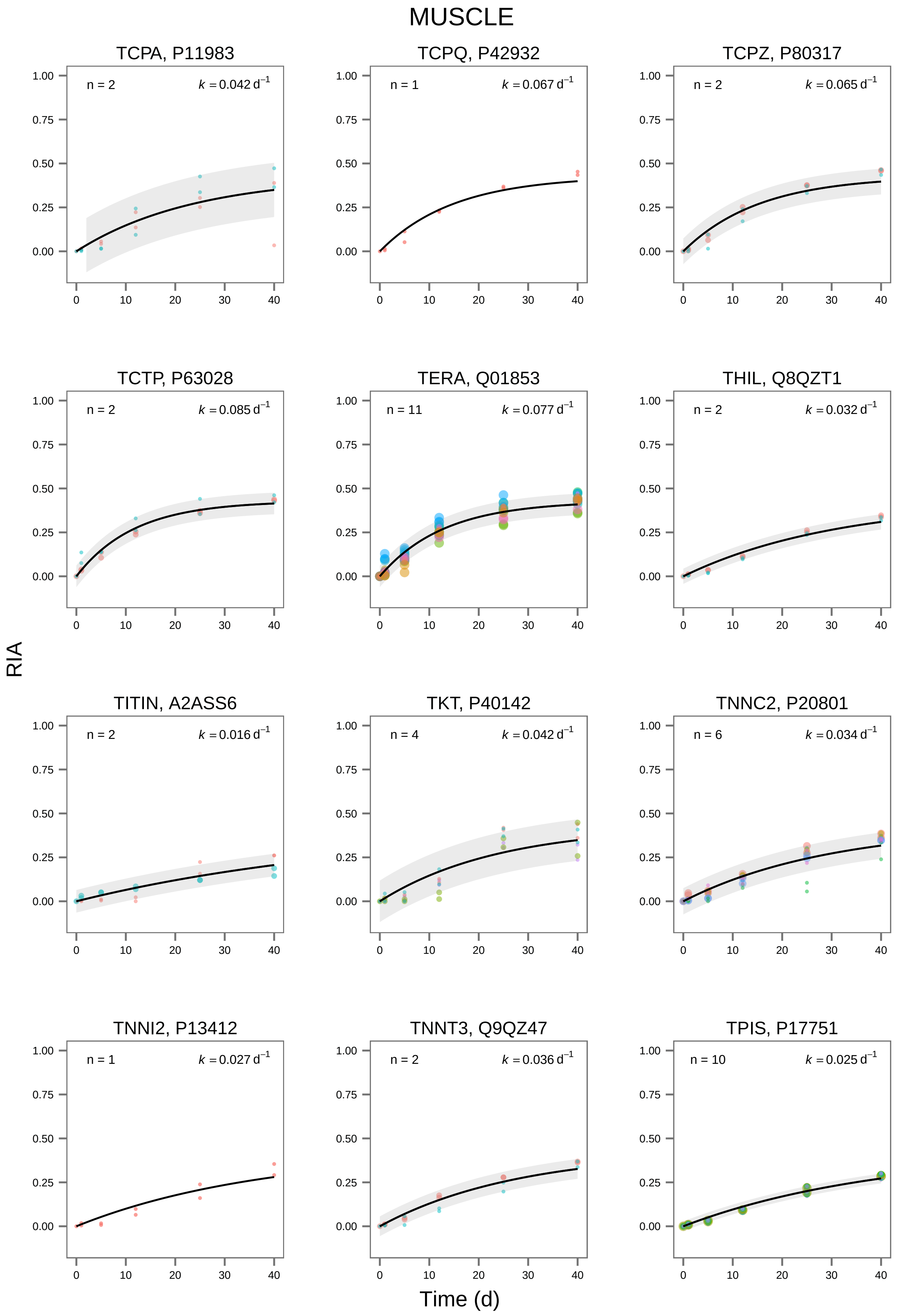
MUSCLE
TCPA, P11983
n = 2
k = 0.042 d−1
TCPQ, P42932
n = 1
k = 0.067 d−1
TCPZ, P80317
n = 2
k = 0.065 d−1
TCTP, P63028
n = 2
k = 0.085 d−1
TERA, Q01853
n = 11
k = 0.077 d−1
THIL, Q8QZT1
n = 2
k = 0.032 d−1
TITIN, A2ASS6
n = 2
k = 0.016 d−1
TKT, P40142
n = 4
k = 0.042 d−1
TNNC2, P20801
n = 6
k = 0.034 d−1
TNNI2, P13412
n = 1
k = 0.027 d−1
TNNT3, Q9QZ47
n = 2
k = 0.036 d−1
TPIS, P17751
n = 10
k = 0.025 d−1
RIA
Time (d)
1.00
0.75
0.50
0.25
0.00
0
10
20
30
40

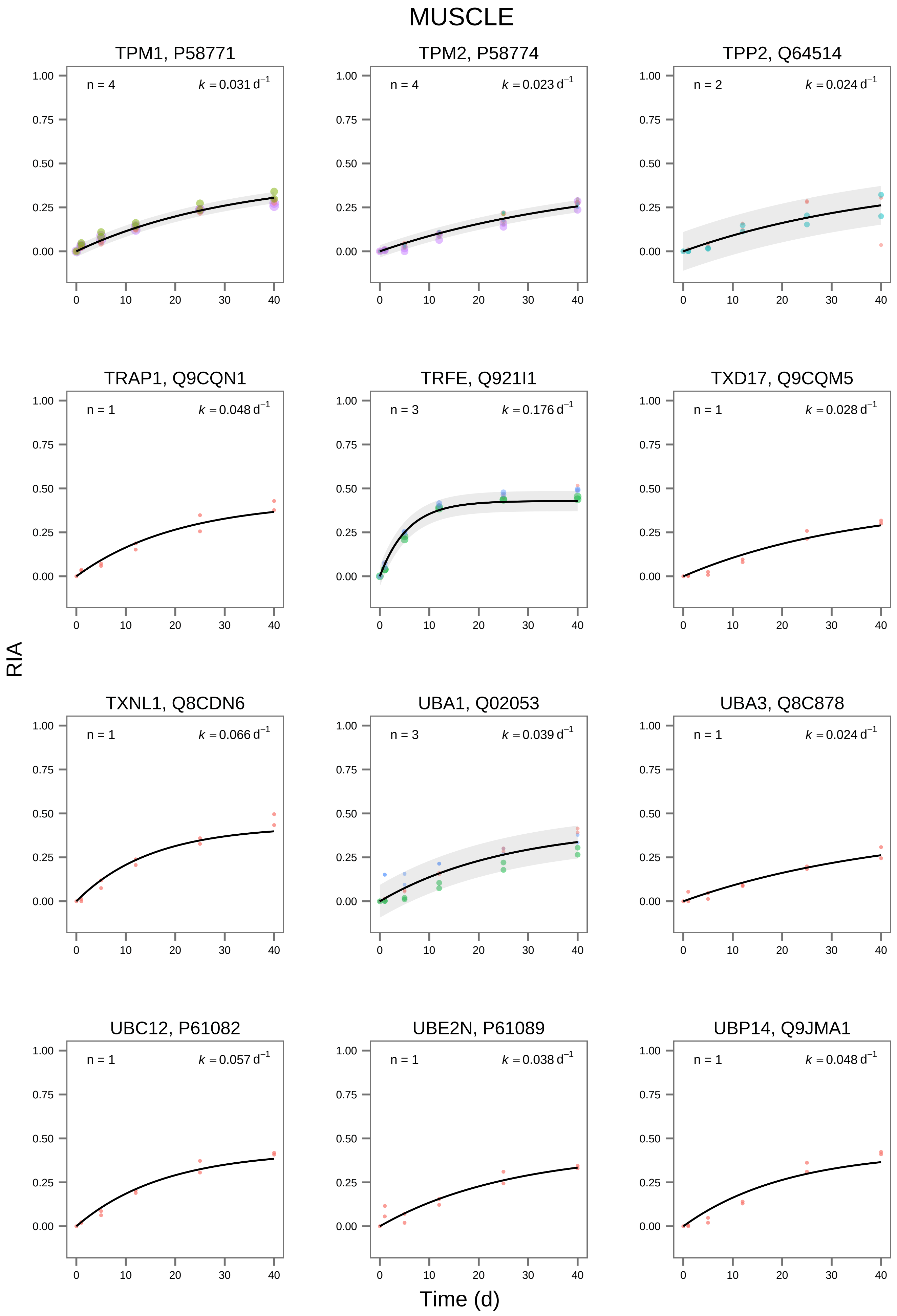
MUSCLE
TPM1, P58771
n = 4
k = 0.031 d−1
TPM2, P58774
n = 4
k = 0.023 d−1
TPP2, Q64514
n = 2
k = 0.024 d−1
TRAP1, Q9CQN1
n = 1
k = 0.048 d−1
TRFE, Q921I1
n = 3
k = 0.176 d−1
TXD17, Q9CQM5
n = 1
k = 0.028 d−1
TXNL1, Q8CDN6
n = 1
k = 0.066 d−1
UBA1, Q02053
n = 3
k = 0.039 d−1
UBA3, Q8C878
n = 1
k = 0.024 d−1
UBC12, P61082
n = 1
k = 0.057 d−1
UBE2N, P61089
n = 1
k = 0.038 d−1
UBP14, Q9JMA1
n = 1
k = 0.048 d−1
RIA
Time (d)

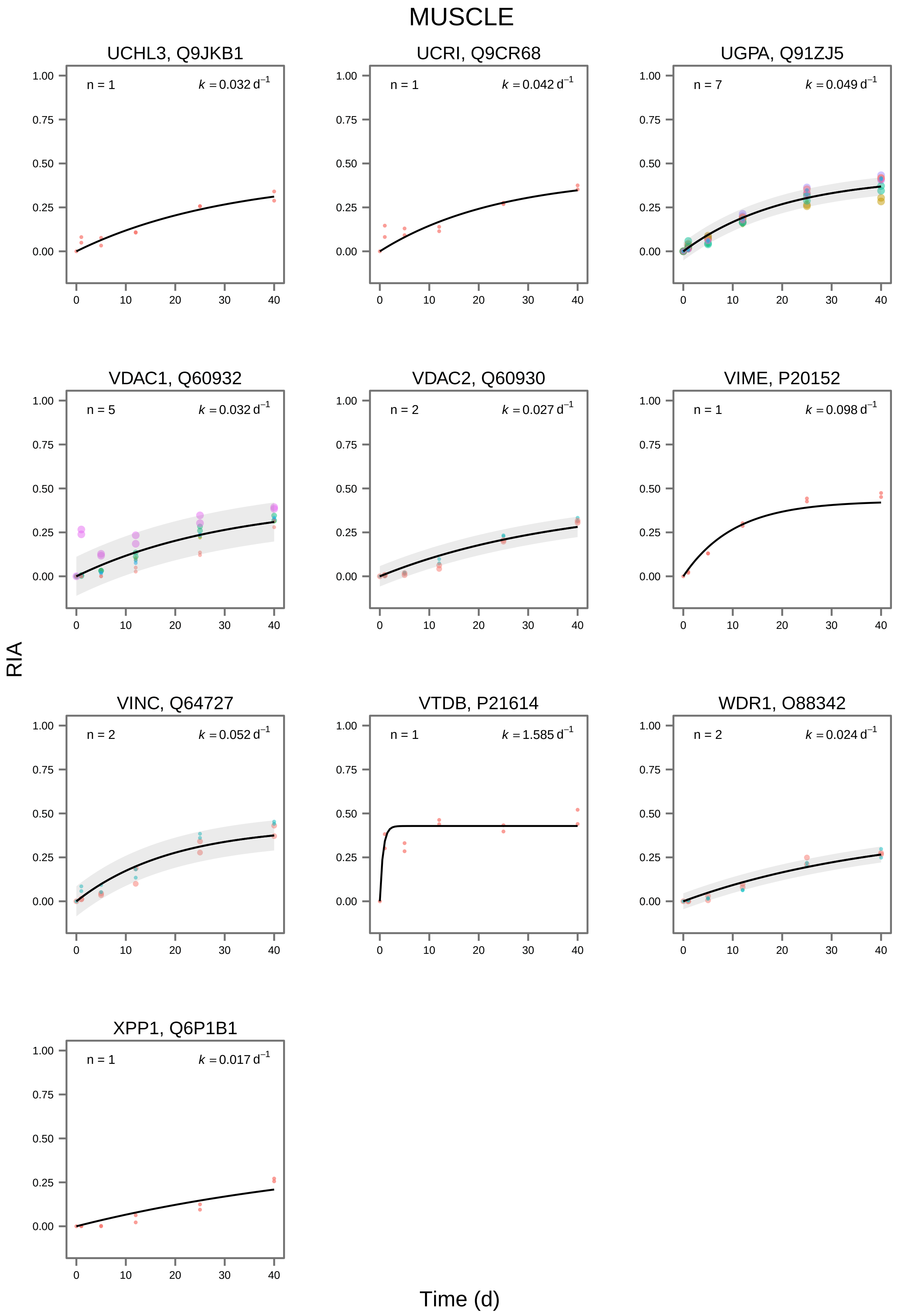
MUSCLE
UCHL3, Q9JKB1
n = 1
k = 0.032 d−1
UCRI, Q9CR68
n = 1
k = 0.042 d−1
UGPA, Q91ZJ5
n = 7
k = 0.049 d−1
VDAC1, Q60932
n = 5
k = 0.032 d−1
VDAC2, Q60930
n = 2
k = 0.027 d−1
VIME, P20152
n = 1
k = 0.098 d−1
VINC, Q64727
n = 2
k = 0.052 d−1
VTDB, P21614
n = 1
k = 1.585 d−1
WDR1, O88342
n = 2
k = 0.024 d−1
XPP1, Q6P1B1
n = 1
k = 0.017 d−1
RIA
Time (d)
