# Supplementary material for: Proteome Dynamics: Tissue Variation in the Kinetics of Proteostasis in Intact Animals
Source: Mol Cell Proteomics. 2016 Feb 1;15(4):1204–19. doi: 10.1074/mcp.M115.053488 (PMC4824850; doi:10.1074/mcp.M115.053488)
Supplement: Supplemental Data [file 10.1074_M115.053488_mcp.M115.053488-5.pdf]

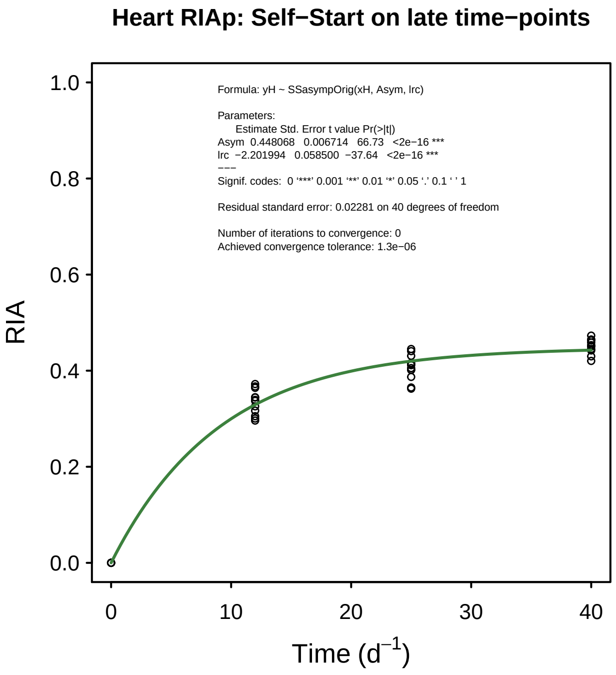
Heart RIAp: Self−Start on late time−points
Formula: yH ~ SSasympOrig(xH, Asym, lrc)
Parameters:
Estimate Std. Error t value Pr(>|t|)
Asym 0.448068 0.006714 66.73 <2e−16 ***
lrc −2.201994 0.058500 −37.64 <2e−16 ***

Signif. codes: 0 '***' 0.001 '**' 0.01 '*' 0.05 '.' 0.1 ' ' 1
Residual standard error: 0.02281 on 40 degrees of freedom
Number of iterations to convergence: 0
Achieved convergence tolerance: 1.3e−06
RIA
Time (d−1)
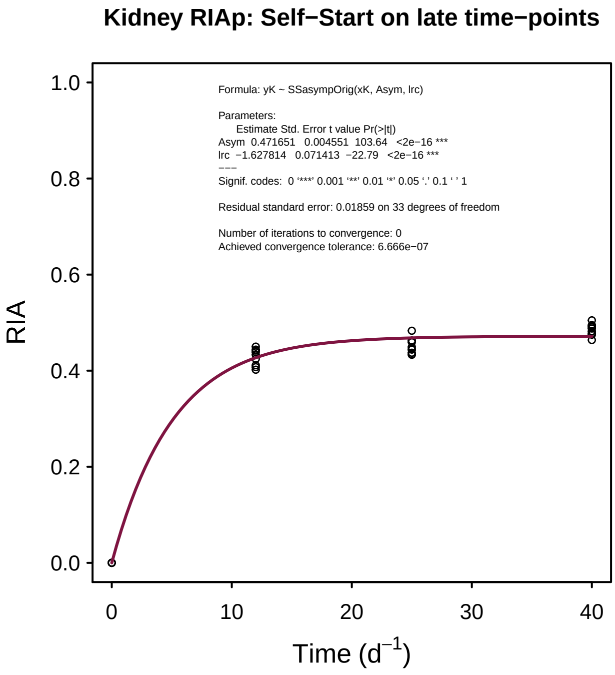
Kidney RIAp: Self−Start on late time−points
Formula: yK ~ SSasympOrig(xK, Asym, lrc)
Parameters:
Estimate Std. Error t value Pr(>|t|)
Asym 0.471651 0.004551 103.64 <2e−16 ***
lrc −1.627814 0.071413 −22.79 <2e−16 ***

Signif. codes: 0 '***' 0.001 '**' 0.01 '*' 0.05 '.' 0.1 ' ' 1
Residual standard error: 0.01859 on 33 degrees of freedom
Number of iterations to convergence: 0
Achieved convergence tolerance: 6.666e−07
RIA
Time (d−1)
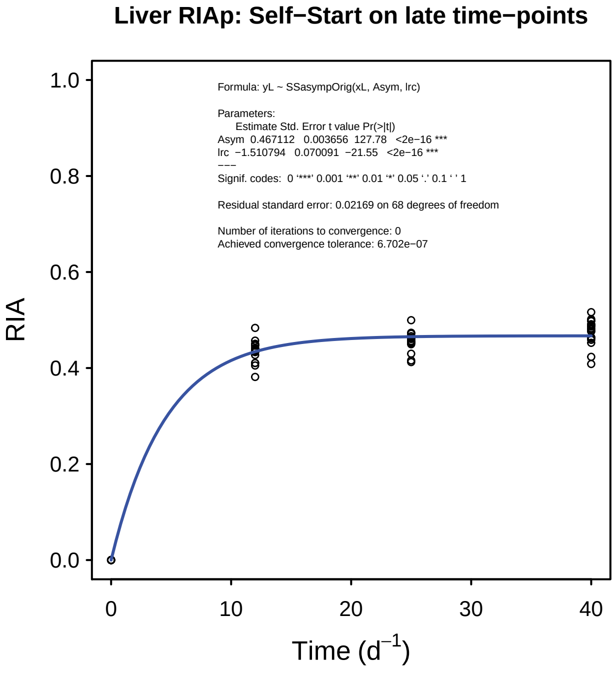
Liver RIAp: Self−Start on late time−points
Formula: yL ~ SSasympOrig(xL, Asym, lrc)
Parameters:
Estimate Std. Error t value Pr(>|t|)
Asym 0.467112 0.003656 127.78 <2e−16 ***
lrc −1.510794 0.070091 −21.55 <2e−16 ***

Signif. codes: 0 '***' 0.001 '**' 0.01 '*' 0.05 '.' 0.1 ' ' 1
Residual standard error: 0.02169 on 68 degrees of freedom
Number of iterations to convergence: 0
Achieved convergence tolerance: 6.702e−07
RIA
Time (d−1)
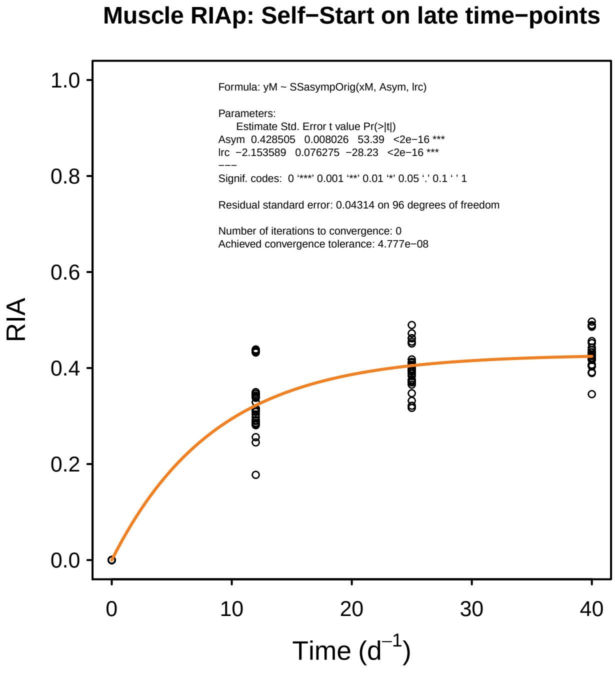
Muscle RIAp: Self−Start on late time−points
Formula: yM ~ SSasympOrig(xM, Asym, lrc)
Parameters:
Estimate Std. Error t value Pr(>|t|)
Asym 0.428505 0.008026 53.39 <2e−16 ***
lrc −2.153589 0.076275 −28.23 <2e−16 ***

Signif. codes: 0 '***' 0.001 '**' 0.01 '*' 0.05 '.' 0.1 ' ' 1
Residual standard error: 0.04314 on 96 degrees of freedom
Number of iterations to convergence: 0
Achieved convergence tolerance: 4.777e−08
RIA
Time (d−1)
